# Supplementary material for: Stereodivergent dehydrative allylation of β-keto esters using a Ru/Pd synergistic catalyst
Source: Nat Commun. 2022 Oct 12;13:5876. doi: 10.1038/s41467-022-33432-4 (PMC9556617; doi:10.1038/s41467-022-33432-4)
Supplement: Supplementary file 1 — Supplementary Information [file 41467_2022_33432_MOESM1_ESM.pdf]

Supplementary Information for
Stereodivergent Dehydrative Allylation of β-Keto Esters Using a Ru/Pd Synergistic Catalyst

Thien Phuc Le[1], Shinji Tanaka[2], Masahiro Yoshimura[3],
Kazuhiko Sato[2], and Masato Kitamura[1]

E-mail: tanaka-sh@aist.go.jp

[1]Graduate School of Pharmaceutical Sciences and Research Center for Materials Science, Nagoya University, Chikusa, Nagoya 464-8601, Japan
[2]Interdisciplinary Research Center for Catalytic Chemistry, National Institute of Advanced Industrial Science and Technology (AIST), Tsukuba, Ibaraki 305-8565, Japan
[3]Division of Liberal Arts and Sciences, Aichi Gakuin University, Iwasaki, Nisshin, Aichi 470-0195, Japan

Table of Contents

Supplementary Method
1. Instruments 1
2. Materials 1
3. General operation 3
4. Dehydrative allylation of β-keto esters 3
5. Generality 4
6. Diastereoselective reduction of **3aa** 28
7. Condition optimization 34
8. Formal synthesis of (+)-pancratistatin 34

Supplementary Figures 38
Supplementary Tables 181
Supplementary References 195

Supplementary Method

1. Instruments.

Nuclear magnetic resonance (NMR) spectra were recorded on JEOL JNM-ECA-600 system (600 MHz for ^{1}H, 152 MHz for ^{13}C), and the chemical shifts are expressed in parts per million (ppm) downfield from $Si(CH_3)_4$ or in ppm relative to $CHCl_3$ (δ 7.26 in ^{1}H NMR; δ 77.16 in ^{13}C NMR, respectively) or CH_3OH (δ 3.34 in ^{1}H NMR; δ 49.86 in ^{13}C NMR, respectively). The signal coupling patterns are indicated as follows: s, singlet; d, doublet; t, triplet; q, quartet; sept, septet; m, multiplet; and br, broad signal. The resolutions of ^{1}H- and ^{13}C-NMR spectra are 0.689 Hz and 1.44 Hz, respectively. Unless otherwise specified, NMR spectra were recorded at 25 °C using $CDCl_3$ containing 0.03 wt% $Si(CH_3)_4$. X-ray crystallographic analyses were conducted on Rigaku Rapid-DW (Cu) system, and the structures were solved by direct methods using "CrystalStructure 4.0" or "CrystalStructure 4.2.2" crystallographic software. ORTEP diagrams were drawn at 50% probability level. High-resolution mass spectra (HRMS) were measured by electrospray ionization (ESI) method on a Bruker Daltonics compact system. High performance liquid chromatography (HPLC) analyses were performed on a Shimadzu LC-10A system. Optical rotations were measured on a JASCO P-1010-GT system. Melting points (mp) were measured on a Yanaco MP-J3 system. Glove box MBraun MB150B-G was used. Weight of reagents, ligands and metal precursors were measured on a Sartorius ME235S system within an accuracy of 0.01 mg.

2. Materials.

2.1. Gases.

Ar gas (99.998%) obtained from Taiyo Nippon Sanso Corporation was purified by passage through a column of BASF R3-11 catalyst at 80 °C and then through a column of granular calcium sulfate.

2.2. Solvents.

Solvents for the metal complexes preparation and catalytic reaction were dried and degassed at the reflux temperature in the presence of appropriate drying agents (2.5 g/L) under Ar for 6 h and distilled into Schlenk flasks before use: cyclopentyl methyl ether (CPME), 1,4-dioxane, tetrahydrofuran (THF), and toluene ($C_6H_5CH_3$) from sodium benzophenone ketyl; *tert*-butyl alcohol (*tert*-C_4H_9OH), chloroform ($CHCl_3$), dichloromethane (CH_2Cl_2), *N*,*N*-dimethylacetamide (DMA) from CaH_2; acetone (CH_3COCH_3) from activated molecular sieves (MS) 4A; methanol (CH_3OH) from activated molecular sieves (MS) 3A. Distilled water was purchased from Wako Pure Chemical and used without further purification. These were degassed by three freeze–thaw cycles before use. One freeze-thaw cycle consists of i) freezing a liquid mixture, ii) evacuation of the system at the freezing stage, iii) closing the system, iv) thawing the frozen liquid, and v) releasing the negative pressure to atmospheric pressure by filling Ar gas.

For the preparation of substrates, extraction, column chromatography, and HPLC analysis, first grade solvents were used without purification: CH_2Cl_2, ether, ethyl acetate (EtOAc), hexane (Hex), toluene and 2-propanol (2-PrOH).

2.3. Reagents and chemicals.

All of reagents which were purchased from companies were used without further purification. These are listed below. **Aldrich:** 1.0 M K-selectride in THF, lanthanum(III) chloride hexahydrate ($LaCl_3 \cdot 6H_2O$), sodium borohydride ($NaBH_4$), potassium methoxide (CH_3OK), 5.0–6.0 M *tert*-butyl hydroperoxide (TBHP) in decane, vanadyl(IV)

acetoacetonate ($VO(acac)_2$) and Grubb's 2nd catalyst. **Nacalai Tesque:** sodium sulfate (Na_2SO_4). **Tokyo Chemical Industry (TCI):** diphenyldiselenide (PhSeSePh), 35% hydrogen peroxide (H_2O_2) in water and triethyl amine (Et_3N). **Wako Pure Chemical:** sodium chloride (NaCl), ammonium chloride (NH_4Cl), sodium hydroxide (NaOH).

Zinc(II) tetrahydroborate ($Zn(BH_4)_2$) was prepared according to the procedures reported[1].

2.4. Substrates.

All of substrates which were purchased from companies were used without further purification. **Tokyo Chemical Industry (TCI):** *tert*-butyl acetoacetate (**1a**), cinnamyl alcohol (**2a**). **Aldrich:** *tert*-butyl 3-oxopentanoate (**1c**), *tert*-butyl 2-methyl-3-oxobutanoate (**1j**).

The next compounds were prepared according to the procedures reported: *tert*-butyl 3-oxopropanoate (**1b**)[2], *tert*-butyl 4-methyl-3-oxopentanoate (**1d**)[3], *tert*-butyl pivaloylacetate (**1e**)[4], *tert*-butyl 4-methoxy-3-oxobutanoate (**1f**)[5], *tert*-butyl 3-oxo-3-phenylpropanoate (**1g**)[6], *tert*-butyl 3-(4-methoxyphenyl)-3-oxopropanoate (**1h**)[7], *tert*-butyl 3-(4-chlorophenyl)-3-oxopropanoate (**1i**)[8], *tert*-butyl 2-benzyl-3-oxobutanoate (**1k**)[9], *tert*-butyl 2-fluoro-3-oxobutanoate (**1l**)[10], *tert*-butyl 2-oxocyclopentane-1-carboxylate (**1m**)[12], *tert*-butyl (*E*)-3-oxohex-4-enoate (**1n**)[13]. (*E*)-3-(2-fluorophenyl)prop-2-en-1-ol (**2b**)[13], (*E*)-3-(*o*-tolyl)prop-2-en-1-ol (**2c**)[14], (*E*)-3-(2-methoxyphenyl)prop-2-en-1-ol (**2d**)[15], (*E*)-3-(3-fluorophenyl)prop-2-en-1-ol (**2e**)[16], (*E*)-3-(*m*-tolyl)prop-2-en-1-ol (**2f**)[16], (*E*)-3-(3-methoxyphenyl)prop-2-en-1-ol (**2g**)[12], (*E*)-3-(4-fluorophenyl)prop-2-en-1-ol (**2h**)[12], (*E*)-3-(4-chlorophenyl)prop-2-en-1-ol (**2i**)[12], (*E*)-3-(4-(trifluoromethyl)phenyl)prop-2-en-1-ol (**2j**)[12], (*E*)-3-(*p*-tolyl)prop-2-en-1-ol (**2k**)[17], (*E*)-3-(4-methoxyphenyl)prop-2-en-1-ol (**2l**)[12], (*E*)-3-(naphthalen-2-yl)prop-2-en-1-ol (**2m**)[12], *tert*-butyl (*E*)-3-(3-hydroxyprop-1-en-1-yl)-1*H*-indole-1-carboxylate (**2n**)[18], *tert*-butyl (*E*)-3-(3-hydroxyprop-1-en-1-yl)-1*H*-pyrrole-1-carboxylate(**2o**)[18], (*E*)-5-phenylpent-2-en-1-ol (**2p**)[19], (*E*)-3-(benzo[*d*][1,3]dioxol-5-yl)prop-2-en-1-ol (**2q**)[20].

2.5. Metal precursors.

All of metal precursors were purchased from companies and were used without further purification. These are listed below in the alphabetical order neglecting number suffix. **Aldrich:** bis(acetonitrile)palladium(II) dichloride ($PdCl_2(CH_3CN)_2$), copper(II) trifluoromethanesulfonate ($Cu(OTf)_2$), nickel(II) trifluoromethanesulfonate ($Ni(OTf)_2$), silver trifluoromethanesulfonate (AgOTf). **TCI:** tris(acetonitrile)cyclopentadienylruthenium(II) hexafluorophosphate ($[CpRu(CH_3CN)_3]PF_6$).

2.6. Ligands.

Ligands which were purchased from companies were used without further purification. These are listed below in the alphabetical order neglecting number suffix. **Aldrich:** 1,10-Phenanthroline (1,10-Phen). **TCI:** (*R*)-SEGPHOS, (*R*)-TolBINAP, (R)-XylBINAP, (*R*)-*t*BuBOX. (*R*)- and (*S*)-BINAP ($\mathbf{L^1}_{\boldsymbol{R}}$ and $\mathbf{L^1}_{\boldsymbol{S}}$) were provided by Takasago International Corpoation.

(*R*,*R*)-Naph-diPIM-dioxo-*i*Pr ($\mathbf{L^2}_{\boldsymbol{R}}$), (*S*,*S*)-Naph-diPIM-dioxo-*i*Pr ($\mathbf{L^2}_{\boldsymbol{S}}$) and achiral Naph-diPIM were synthesized according to the procedures reported[21].

2.7. Synthesis of catalysts.

$[Pd(\mathit{rac}\text{-binap})(H_2O)_2](OTf)_2$, $[Pd((R)\text{-binap})(H_2O)_2](OTf)_2$ (Pd$\mathbf{L^1}_{\boldsymbol{R}}$(OTf)), [Pd((S)-binap)$(H_2O)_2](OTf)_2$ (Pd$\mathbf{L^1}_{\boldsymbol{S}}$(OTf)), $[Pd((R)\text{-tolbinap})(H_2O)_2](OTf)_2$, [Pd((*R*)-

xylbinap)(H_2O)2](OTf)2 and [Pd((*R*)-segphos)(H_2O)2](OTf)2 were prepared according to the procedures reported by Sodeoka *et al*[22].

[CpRu((*R*,*R*)-Naph-diPIM-dioxo-*i*Pr)]PF_6 (Ru**L**^2_R), [CpRu((*S*,*S*)-Naph-diPIM-dioxo-*i*Pr)]PF_6 (Ru**L**^2_S), [CpRu(Naph-diPIM)]PF_6 were prepared according to the procedures reported by Kitamura *et al*[21].

2.8. Authentic products.

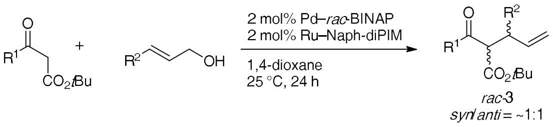

Racemic products, (±)-**3** was prepared by the dehydrative allylation of the corresponding β-keto ester (**1a–n**) and allylic alcohol (**2a–q**) (2 mol% of [Pd(*rac*-binap)(H_2O)2](OTf)2, 2 mol% of [CpRu(Naph-diPIM)]PF_6, 1,4-dioxane, 25 °C, 24 h), otherwise specified. Detail procedures were same as that described in section 4.1. The ^{1}H-NMR spectra of the authentic samples were consistent with those of the synthetic products obtained by Pd**L**^1_R(OTf)/Ru**L**^2_R or Pd**L**^1_S(OTf)/Ru**L**^2_R catalyzed dehydrative allylation (see section 4 and 5).

Racemic products, (±)-*syn*-**3ia**, (±)-*syn*-**3ja**, (±)-*syn*-**3aj**, (±)-*syn*-**3an** and (±)-*syn*-**3ao** were prepared by mixing product and its enantiomer in 1:1 ratio.

2.9. Silica gels, Celite, and MS.

Analytical thin-layer chromatography (TLC) was performed using Merck 5715 plates precoated with silica gel 60 F254 (layer thickness, 0.25 mm). The product spots were visualized with a solution of cerium(IV) sulfate-phosphomolybdic acid (CePMP). Flash silica-gel column chromatography (SiO_2-chromatography) was performed using AP 300. MS 3A and 4A were purchased from Nacalai Tesque and were activated at 250 °C under vacuum before use.

3. General operation.

A Teflon-coated magnetic bar was used for stirring a reaction mixture. Room temperature (rt) ranges from 25 °C to 28 °C. Temperatures were controlled using Eyela Chemistation PPS-CTRL 1 for reaction screening and Eyela PSL-2500B for organic synthesis. Solvents after general workup process were removed by means of a rotary evaporator. Concentration of a reaction mixture in a Schlenk tube was performed by connecting to a vacuum-Ar line via a cold trap. All metal-catalyzed reactions were carried out under Ar atmosphere by use of a general Schlenk technique unless otherwise specified. A Schlenk with Teflon J. Young valve was specified by "Young-type Schlenk." Schlenks were dried at ca. 250 °C by use of a heat gun under a reduced pressure. Liquid reagents were introduced by use of a syringe via a septum rubber. After introduction, the septum was replaced with a glass stopper or with a Young valve. Heating in a closed system was carried out after reducing the pressure of the whole system or after raising the temperature followed by closing the system. Degassed solvents and degassed solutions of reagents, catalysts, and substrates were transferred to another Schlenk by use of a gas-tight syringe or cannulation method. Cannulation was performed by use of a Teflon or stainless tube through a septum rubber under a slightly positive pressure of Ar.

4. Dehydrative Allylation of β-keto esters.

Product number in stereodivergent dehydrative allylation of β-keto esters is defined as **3xy**, in which the alphabet symbols, **x** and **y,** represent those of β-keto esters

1x and allylic alcohol **2y**: e.g., the product in the reaction of **1a** and **2b** is expressed as **3ab**.

4.1. General procedure.

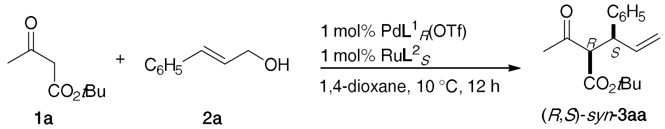

The general procedure was given in the Method section of the manuscript: ^{1}H NMR ($CDCl_3$) δ 1.14 (s, 9H, $C(CH_3)_3$), 2.30 (s, 3H, CH_3), 3.90 (d, *J* = 11.4 Hz, 2H, CH_2), 4.07 (dd, *J* = 7.20, 11.40 Hz, 1H, PhC*H*CH=CH_2), 5.02 (d, *J* = 4.20 Hz, 1H, PhCHCH=CH*H*), 5.05 (s, 1H, PhCHCH=CH*H*), 5.85–5.91 (m, 1H, PhCHC*H*=CH_2), 7.19–7.23 (m, 3H, ArH), 7.28–7.30 (m, 2H, ArH); ^{13}C NMR ($CDCl_3$) δ 27.59, 29.55, 49.37, 66.46, 82.12, 116.37, 127.08, 128.43, 128.63, 138.62, 140.43, 166.82, 202.16; HRMS (ESI) calcd for $C_{17}H_{22}NaO_3$ [M+Na$^+$] 297.1467, found 297.1468; $[\alpha]_D^{20}$ +31.894 (*c* 0.697, $CHCl_3$). **Supplementary Figure 1** showed the ^{1}H- and ^{13}C-NMR spectra.

4. 2. Determination of conversion, ^{1}H-NMR yield, diastereomer and enantiomer ratio.

The reaction mixture obtained under the above conditions in a different batch was used for the purpose. The reaction mixture was concentrated in vacuo. To the resulting pale-yellow solid was added $CDCl_3$ (ca. 4.00 mL) and mesitylene (69.5 μL, 60.0 mg, 0.500 mmol, 1.00 mol amt) as an internal standard. This solution was subjected to ^{1}H-NMR analysis with 10-sec repetition time so that the integrations of the signal areas become accurate as much as possible. The 99.58:0.41:33.30 ratio of the signal intensities of *syn*-**3aa** (δ 1.14, 9H, $C(CH_3)_3$), *anti*-**3aa** (δ 1.46, 9H, $C(CH_3)_3$), and mesitylene (δ 6.79, 3H, 3 x ArH) determined the yield to be >99%. The 99.58:0.41 ratio of the signal intensities of *syn*-**3aa** (δ 1.37, 9H, $C(CH_3)_3$) and *anti*-**3aa** (δ 1.46, 9H, $C(CH_3)_3$) determined the diastereomer ratio (*syn*:*anti*) to be 99.6:0.4 (**Supplementary Figure 2**).

The enantiomer ratio (er) of **3aa** was determined by HPLC analysis to be 99.9:0.1 (4.60 mmϕ x 250 mm DAICEL CHIRALPAK ID-3 column; 1.0:99.0 2-PrOH–Hex eluent; 0.50 mL/min flow rate; 254-nm detection; 25 °C; retention time (t_R), 13.2 min (2*R*,3*S*, major), 15.2 min (2*S*,3*R*, minor)). **Supplementary Figure 3** showed the HPLC chart.

The absolute configuration (abs confign) of the major stereoisomer was determined by the X-ray crystallographic analysis of the product to be (2*R*,3*S*)-*syn*-**3aa**. White solid *syn*-**3aa** (10.0 mg, 99.6:0.4 dr, 99.9:0.1 er) was dissolved in Hex (2.00 mL) in a 5-mL glass tube with a screw cap and the solution was kept at –20 °C for 2 h, giving colorless block crystals (ca. 70% yield, mp 53.0 °C). The crystal was subjected to X-ray crystallographic analysis and the abs confign of *syn*-**3aa** derived from the Pd$\mathbf{L^1}_R$(OTf) and Ru$\mathbf{L^2}_S$-catalyzed allylation was determined to be 2*R*,3*S* (Flack parameter, 0.09(6) for (2*R*,3*S*)-*syn*-**3aa**). **Supplementary Table 1** summarized the crystallographic data, and the molecular structure in a crystal was shown in **Supplementary Figure 4**. Crystallographic data have been deposited with Cambridge Crystallographic Data Centre as supplementary publication no. CCDC-2193030

5. Generality.

5.1. Matched system.

According to the general procedure described for dehydrative allylation of β-keto ester **1a** and allylic alcohol **2a**, all reactions were carried out basically under the

conditions of 0.500-mmol scale of substrates, $[RuCp(CH_3CN)_3]PF_6$ (5.00 μmol), $\mathbf{L^2}_S$ (5.00 μmol), Pd$\mathbf{L^1}_R$(OTf) (5.00 μmol), 1,4-dioxane (1.00 mL). Listed below are the reaction conditions (β-keto ester (mg, mmol), allylic alcohol (mg, mmol, mol amt), reaction time and reaction temperature), isolated yield, physical properties of the products. The chemical shifts and retention times of synthetic products with high ers were sometimes slightly different from those of racemic samples. In these cases, the data of synthetic ones were reported. Relative configurations and abs configns of **3aa**, **3ba**, **3ia**, **3ab**, **3ae**, **3ah**, **3am**, and **3ao** were determined by X-ray crystallographic analysis as described below. Relative configurations of other products were assigned by comparison a chemical shift of *tert*-butyl ester moiety in ^{1}H-NMR analysis (*syn*: 1.05–1.30, *anti*: 1.35–1.65) with those of structure-confirmed products. Abs configns of them were not determined, and the structures in schemes and figures were drawn as estimated ones from other structure-confirmed products.

tert-Butyl (2*R*,3*S*)-2-(hydroxymethyl)-3-phenylpent-4-enoate (*syn*-**4ba**)

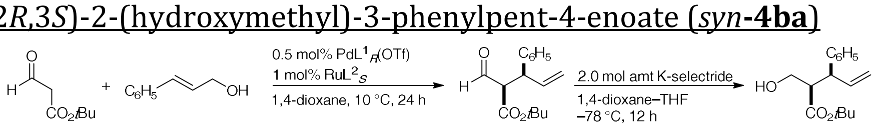

Reaction conditions (**Table 2**, entry 1): β-keto ester **1b** (72.0 mg, 0.500 mmol); allylic alcohol **2a** (0.750 M in 1,4-dioxane, 1.00 mL, 0.750 mmol, 1.50 mol amt); $[RuCp(CH_3CN)_3]PF_6$ (2.17 mg, 4.99 μmol, 1.00 mol%); $\mathbf{L^2}_S$ (2.74 mg, 5.03 μmol, 1.00 mol%); Pd$\mathbf{L^1}_R$(OTf) (2.66 mg, 2.50 μmol, 0.50 mol%); 10 °C; 24 h. After 24 h: >99% conversion, branch/linear =>99.9:<0.1, *syn*:*anti* = 99.6:0.4. **Supplementary Figure 5** showed a ^{1}H-NMR spectrum of the reaction mixture. The epimerization of **3ba** underwent very quickly under SiO_2-chromatography purification. Therefore, the product of this reaction has been transformed and isolated as a β-hydroxy ester after one-pot reduction using K-selectride in –78 °C: The reaction mixture was diluted with dry THF (4.00 mL) and cooled to –78 °C. To this mixture, 1.0 M solution of K-selectride in THF (1.00 mL, 1.00 mmol, 2.00 mol amt) was added dropwise and the reaction mixture was stirred at this temperature for 12 h. After 12 h, 35% H_2O_2 aq. (1.00 mL) and 3.0 M NaOH aq. (1.00 mL) were added, and the reaction mixture was slowly warmed to rt. The resulting solution was extracted with ether (3 x 15 mL). The organic layers were combined, washed with brine (20 mL), filtered through a pad of Celite, dried over Na_2SO_4, filtered, and concentrated. After work-up: single diastereomer. The residue was purified by SiO_2-chromatography (40 g; 1:5 EtOAc–Hex eluent) to give the product as a white solid (107 mg, 81.6%). ^{1}H NMR ($CDCl_3$) δ 1.15 (s, 9H, $C(CH_3)_3$), 2.38–2.40 (m, 1H, CH_2OH), 2.84–2.86 (m, 1H, C*H*$CO_2C(CH_3)_3$), 3.65 (dd, *J* = 9.60, 9.60 Hz, 1H, PhC*H*CH=CH_2), 3.84–3.88 (m, 2H, C*H2*OH), 5.10 (d, *J* = 9.60 Hz, 1H, PhCHCH=CH*H*), 5.17 (d, *J* = 17.4 Hz, 1H, PhCHCH=CH*H*), 5.94 (ddd, *J* = 9.60, 9.60, 17.4 Hz, 1H, PhCHC*H*=CH_2), 7.19–7.30 (m, 5H, ArH); ^{13}C NMR ($CDCl_3$) δ 27.77, 49.99, 52.77, 62.20, 81.23, 116.78, 126.86, 128.43, 128.54, 138.82, 141.50, 173.55; HRMS (ESI) calcd for $C_{16}H_{22}NaO_3$ $[M+Na^+]$ 285.1467, found 285.1466; $[\alpha]_D^{20}$ –40.759 (*c* 0.630, $CHCl_3$).

Enantiomer ratio: 99.9:0.1 (4.60 mmϕ x 250 mm DAICEL CHIRALPAK IA and 4.60 mmϕ x 250 mm DAICEL CHIRALPAK IA-3 columns; 1.0:99.0 2-PrOH–Hex eluent; 0.50 mL/min flow rate; 220-nm detection; 25 °C; t_R, 45.1min (2*S*,3*R*, minor), 47.9 min (2*R*,3*S*, major)). **Supplementary Figures 6** and **7** showed the ^{1}H- and ^{13}C-NMR spectra and HPLC charts, respectively.

The abs confign was determined by the X-ray crystallographic analysis of (2*R*,3*S*)-2-(hydroxymethyl)-3-phenylpentanoic acid ((2*R*,3*S*)-*syn*-**11ba**), which was

prepared by hydrolysis of (2*R*,3*S*)-*syn*-**4ba** under acidic condition. To a solution of (2*R*,3*S*)-*syn*-**4ba** (52.5 mg, 0.200 mmol) in THF (1.00 mL) was added 4 M HCl aq. (4.00 mL) at rt. After 12 h stirring at rt, the mixture was extracted with $CHCl_3$ (10 mL x 3). The organic extracts were dried over Na_2SO_4, filtered, and concentrated under vacuum to afford (2*R*,3*S*)-**11ba** (37.9 mg, 91.9% yield) as a white solid. ^{1}H NMR ($CDCl_3$) δ 2.98–3.01 (m, 1H, C*H*COOH), 3.70 (dd, *J* = 9.60 Hz, 1H, PhC*H*CH=CH$_2$), 3.88 (d, *J* = 4.80 Hz, 2H, C*H$_2$*OH), 5.12 (d, *J* = 10.8 Hz, 1H, PhCHCH=CH*H*), 5.18 (d, *J* = 17.4 Hz, 1H, PhCHCH=CH*H*), 5.96 (ddd, *J* = 9.60, 10.8, 17.4 Hz, 1H, PhCHC*H*=CH$_2$), 7.19–7.30 (m, 5H, ArH); ^{13}C NMR ($CDCl_3$) δ 49.50, 52.39, 61.99, 117.33, 127.12, 127.84, 138.02, 141.10, 177.83; HRMS (ESI) calcd for $C_{12}H_{13}O_3$ [M–H$^-$] 205.0870, found 205.0878; $[\alpha]_D^{20}$ –86.923 (*c* 0.587, $CHCl_3$). **Supplementary Figure 8** showed the ^{1}H- and ^{13}C-NMR spectra of (2*R*,3*S*)-**11ba**.

Recrystallization: (2*R*,3*S*)-**11ba** (10.0 mg); overlay of Hex (2 mL) on $CHCl_3$ solution of (2*R*,3*S*)-**11ba** (0.5 mL); rt/12 h; crystallization yield, ca. 60%; mp 122 °C; Flack, 0.05(5) for (2*R*,3*S*)-**11ba**. **Supplementary Table 2** summarized the crystallographic data, and the molecular structure in a crystal was shown in **Supplementary Figure 9**. Crystallographic data have been deposited with Cambridge Crystallographic Data Centre as supplementary publication no. CCDC-2193052.

tert-Butyl (2*R**,3*S**)-3-phenyl-2-propionylpent-4-enoate (*syn*-**3ca**)

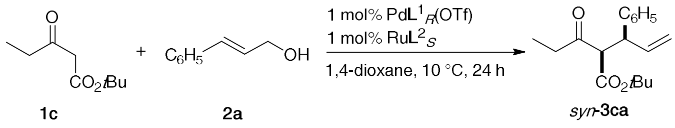

Reaction conditions (**Table 2**, entry 3): β-keto ester **1c** (86.3 mg, 0.500 mmol); allylic alcohol **2a** (0.600 M in 1,4-dioxane, 1.00 mL, 0.600 mmol, 1.20 mol amt); [RuCp(CH_3CN)$_3$]PF$_6$ (2.17 mg, 4.99 μmol, 1.00 mol%); **L**^{2}$_S$ (2.74 mg, 5.03 μmol, 1.00 mol%); Pd**L**^{1}$_R$(OTf) (5.31 mg, 5.00 μmol, 1.00 mol%); 10 °C; 24 h. After 24 h: >99% conversion, branch/linear = >99.9:<0.1, *syn*:*anti* = 99.4:0.6. **Supplementary Figure 10** showed a ^{1}H-NMR spectrum of the reaction mixture. Work up: filtration through short pad of silica. Purification: neutralized SiO$_2$-chromatography (20 g; toluene, within 5 min). Results: **3ca**, 141 mg, 97.8% yield. ^{1}H NMR ($CDCl_3$) δ 1.08 (t, *J* = 7.20 Hz, 3H, CH$_2$C*H$_3$*), 1.13 (s, 9H, C(CH$_3$)$_3$), 2.53–2.68 (m, 2H, C*H$_2$*CH$_3$), 3.94 (d, *J* = 11.4 Hz, 1H, COCH), 4.10 (dd, *J* = 7.80, 11.4 Hz, 1H, PhC*H*CH=CH$_2$), 5.00 (d, *J* = 6.00 Hz, 1H, PhCHCH=CH*H*), 5.02 (d, *J* = 1.20 Hz, 1H, PhCHCH=CH*H*), 5.88 (ddd, *J* =1.20, 6.00, 7.80 Hz, 1H, PhCHC*H*=CH$_2$), 7.19–7.30 (m, 5H, ArH); ^{13}C NMR ($CDCl_3$) δ 7.73, 27.61, 36.21, 49.37, 65.38, 81.99, 116.31, 127.00, 128.42, 128.60, 138.75, 140.64, 166.85, 204.62; HRMS (ESI) calcd for $C_{18}H_{24}NaO_3$ [M+Na$^+$] 311.1623, found 311.1625; $[\alpha]_D^{21}$ +30.269 (*c* 0.529, $CHCl_3$).

Enantiomer ratio: 99.7:0.3 (4.60 mmϕ x 250 mm DAICEL CHIRALPAK IG; 0.5:99.5 2-PrOH–Hex eluent; 0.50 mL/min flow rate; 220-nm detection; 25 °C; t_R, 24.1min (major), 32.3 min (minor)). **Supplementary Figures 11** and **12** showed the ^{1}H- and ^{13}C-NMR spectra and HPLC charts, respectively.

tert-Butyl (2*R**,3*S**)-2-isobutyryl-3-phenylpent-4-enoate (*syn*-**3da**)

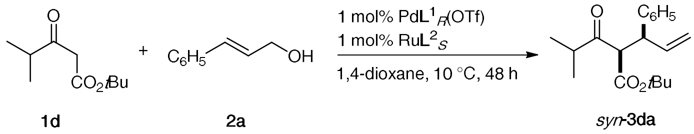

Reaction conditions (**Table 2**, entry 4): β-keto ester **1d** (93.4 mg, 0.501 mmol); allylic alcohol **2a** (0.600 M in 1,4-dioxane, 1.00 mL, 0.600 mmol, 1.20 mol amt); [RuCp(CH_3CN)$_3$]PF$_6$ (2.14 mg, 4.98 μmol, 1.00 mol%); **L**^{2}$_S$ (2.75 mg, 5.04 μmol, 1.00

mol%); Pd**L¹*R***(OTf) (5.29 mg, 4.99 μmol, 1.00 mol%); 10 °C; 48 h. After 48 h: >99% conversion, branch/linear = 98.1:1.9, *syn*:*anti* = 96.1:3.9. **Supplementary Figure 13** showed a ¹H-NMR spectrum of the reaction mixture. Work up: filtration through short pad of silica. Purification: neutralized SiO_2-chromatography (20 g; toluene, within 5 min). Results: **3da**, 138 mg, 91.3% yield. ¹H NMR ($CDCl_3$) δ 1.11 (d, *J* = 7.20 Hz, 3H, $CH_3CHC\mathit{H_3}$), 1.12 (s, 9H, $C(CH_3)_3$), 1.14 (d, *J* = 7.20 Hz, 3H, $C\mathit{H_3}CHCH_3$), 2.80 (sep, *J* = 7.20 Hz, 1H, $C\mathit{H}(CH_3)_2$), 4.08–4.14 (m, 2H, COCH and $PhC\mathit{H}CH{=}CH_2$), 4.97–5.00 (m, 2H, $PhCHCH{=}C\mathit{H_2}$), 5.85 (m, 1H, $PhCHC\mathit{H}{=}CH_2$), 7.19–7.30 (m, 5H, ArH); ¹³C NMR ($CDCl_3$) δ 18.00, 18.18, 27.59, 41.77, 49.46, 63.62, 81.91, 116.48, 126.95, 128.54, 138.85, 140.67, 166.55, 207.37; HRMS (ESI) calcd for $C_{19}H_{26}NaO_3$ [$M+Na^+$] 325.1780, found 325.1798; $[\alpha]_D^{22}$ +38.971 (*c* 0.506, $CHCl_3$).

Enantiomer ratio: 99.9:0.1 (4.60 mmϕ x 250 mm DAICEL CHIRALPAK IA-3 column; 1.0:99.0 2-PrOH–Hex eluent; 0.50 mL/min flow rate; 220-nm detection; 25 °C; t_R, 12.0 min (major), 15.1 min (minor)). **Supplementary Figures 14** and **15** showed the ¹H- and ¹³C-NMR spectra and HPLC charts, respectively.

<u>*tert*-Butyl 3-phenyl-2-pivaloylpent-4-enoate (*syn*-**3ea**)</u>

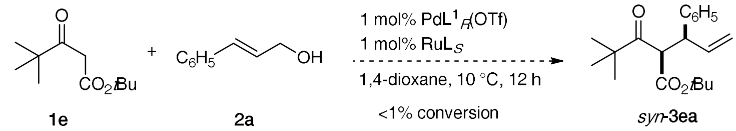

Reaction conditions (**Table 2**, entry 5): β-keto ester **1e** (93.4 mg, 0.501 mmol); allylic alcohol **2a** (0.600 M in 1,4-dioxane, 1.00 mL, 0.600 mmol, 1.20 mol amt); $[RuCp(CH_3CN)_3]PF_6$ (2.14 mg, 4.98 μmol, 1.00 mol%); **L²*S*** (2.73 mg, 5.01 μmol, 1.00 mol%); Pd**L¹*R***(OTf) (5.29 mg, 4.99 μmol, 1.00 mol%); 10 °C; 48 h. Work up: filtration through short pad of silica. Results: <1% conversion.

<u>*tert*-Butyl (2*R**,3*S**)-2-(2-methoxyacetyl)-3-phenylpent-4-enoate (*syn*-**3fa**)</u>

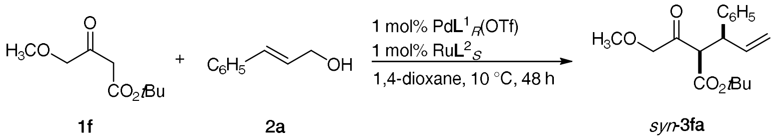

Reaction conditions (**Table 2**, entry 6): β-keto ester **1f** (94.1 mg, 0.500 mmol); allylic alcohol **2a** (0.600 M in 1,4-dioxane, 1.00 mL, 0.600 mmol, 1.20 mol amt); $[RuCp(CH_3CN)_3]PF_6$ (2.15 mg, 4.95 μmol, 0.99 mol%); **L²*S*** (2.76 mg, 5.06 μmol, 1.01 mol%); Pd**L¹*R***(OTf) (5.30 mg, 4.99 μmol, 1.00 mol%); 10 °C; 48 h. After 48 h: >99% conversion, branch/linear = >99.9:<0.1, *syn*:*anti* = 97.6:2.4. **Supplementary Figure 16** showed a ¹H-NMR spectrum of the reaction mixture. Work up: filtration through short pad of silica. Purification: neutralized SiO_2-chromatography (20 g; toluene, within 5 min). Results: *syn*-**3fa**, 147 mg, 96.4% yield. ¹H NMR ($CDCl_3$) δ 1.15 (s, 9H, $C(CH_3)_3$), 3.43 (s, 3H, OCH_3), 4.05–4.21 (m, 4H, $CH_3OC\mathit{H_2}$, COCH and $PhC\mathit{H}CH{=}CH_2$), 5.04 (d, *J* = 4.80 Hz, 1H, $PhCHCH{=}CH\mathit{H}$), 5.02 (d, *J* = 11.40 Hz, 1H, $PhCHCH{=}CH\mathit{H}$), 5.89 (ddd, *J* = 4.80, 7.80, 11.4 Hz, 1H, $PhCHC\mathit{H}{=}CH_2$), 7.19–7.30 (m, 5H, ArH); ¹³C NMR ($CDCl_3$) δ 27.61, 49.15, 59.48, 61.22, 77.69, 82.28, 116.76, 127.09, 128.39, 128.64, 138.31, 140.43, 166.25, 201.78; HRMS (ESI) calcd for $C_{18}H_{24}ClNaO_4$ [$M+Na^+$] 327.1572, found 327.1569; $[\alpha]_D^{22}$ +26.592 (*c* 0.621, $CHCl_3$).

Enantiomer ratio: 99.9:0.1 (4.60 mmϕ x 250 mm DAICEL CHIRALPAK IG column; 1.0:99.0 2-PrOH–Hex eluent; 1.00 mL/min flow rate; 220-nm detection; 25 °C; 24.0min (major), 29.9 min (minor)). **Supplementary Figures 17** and **18** showed the ¹H- and ¹³C-NMR spectra and HPLC charts, respectively.

tert-Butyl (2R*,3S*)-2-benzoyl-3-phenylpent-4-enoate (*syn*-**3ga**)

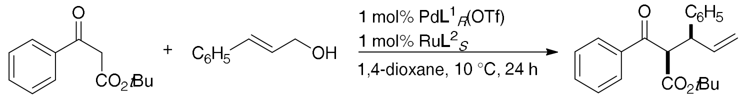

1g **2a** *syn*-**3ga**

Reaction conditions (**Table 2**, entry 7): β-keto ester **1g** (110 mg, 0.499 mmol); allylic alcohol **2a** (0.600 M in 1,4-dioxane, 1.00 mL, 0.600 mmol, 1.20 mol amt); $[RuCp(CH_3CN)_3]PF_6$ (2.16 mg, 4.99 μmol, 1.00 mol%); $\mathbf{L^2}_{S}$ (2.74 mg, 5.02 μmol, 1.00 mol%); Pd$\mathbf{L^1}_{R}$(OTf) (5.30 mg, 4.99 μmol, 1.00 mol%); 10 °C; 24 h. After 24 h: >99% conversion, branch/linear = >99.9:<0.1, *syn*:*anti* = 99.5:0.5. **Supplementary Figure 19** showed a ^{1}H-NMR spectrum of the reaction mixture. Work up: filtration through short pad of silica. Purification: neutralized SiO_2-chromatography (20 g; toluene, within 5 min). Results: *syn*-**3ga**, 163 mg, 96.9% yield. ^{1}H NMR ($CDCl_3$) δ 1.06 (s, 9H, $C(CH_3)_3$), 4.41 (dd, *J* = 7.20, 11.4 Hz, 2H, CH_2), 4.80 (d, *J* = 11.4 Hz, 1H, COCH), 4.95 (d, *J* = 1.20 Hz, 1H, PhCHCH=CH*H*), 4.97 (d, *J* = 4.80 Hz, 1H, PhCHCH=CH*H*), 5.90 (ddd, *J* = 1.20, 4.80, 7.20 Hz, 1H, PhCHC*H*=CH_2), 7.23–7.33 (m, 5H, ArH), 7.48–7.51 (m, 2H, ArH), 7.59–7.60 (m, 1H, ArH), 8.11–8.12 (m, 2H, ArH); ^{13}C NMR ($CDCl_3$) δ 27.55, 49.24, 60.48, 82.09, 116.11, 127.07, 128.55, 128.81, 128.90, 128.96, 133.57, 137.24, 139.01, 140.55, 166.71, 193.41. HRMS (ESI) calcd for $C_{22}H_{24}NaO_3$ [M+Na$^+$] 359.1623, found 359.1623; $[\alpha]_D^{22}$ –11.88 (*c* 0.476, $CHCl_3$).

Enantiomer ratio: 99.4:0.6 (4.60 mmϕ x 250 mm DAICEL CHIRALPAK IB and 4.60 mmϕ x 250 mm DAICEL CHIRALPAK IB-3 columns; 0.2:99.8 2-PrOH–Hex eluent; 1.00 mL/min flow rate; 220-nm detection; 25 °C; t_R, 23.4 min (major), 26.9 min (minor)). **Supplementary Figures 20** and **21** showed the ^{1}H- and ^{13}C-NMR spectra and HPLC charts, respectively.

tert-Butyl (2R*,3S*)-2-(4-methoxybenzoyl)-3-phenylpent-4-enoate (*syn*-**3ha**)

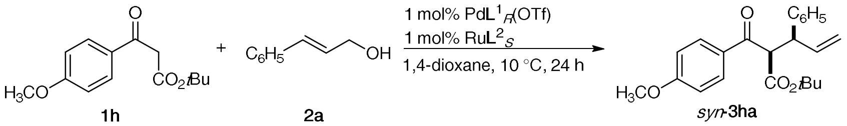

1h **2a** *syn*-**3ha**

Reaction conditions (**Table 2**, entry 8): β-keto ester **1h** (126 mg, 0.503 mmol); allylic alcohol **2a** (0.600 M in 1,4-dioxane, 1.00 mL, 0.600 mmol, 1.20 mol amt); $[RuCp(CH_3CN)_3]PF_6$ (2.17 mg, 5.00 μmol, 1.00 mol%); $\mathbf{L^2}_{S}$ (2.74 mg, 5.02 μmol, 1.00 mol%); Pd$\mathbf{L^1}_{R}$(OTf) (5.30 mg, 4.99 μmol, 1.00 mol%); 10 °C; 24 h. After 24 h: >99% conversion, branch/linear = 95.8:4.2, *syn*:*anti* = 99.4:0.6. **Supplementary Figure 22** showed a ^{1}H-NMR spectrum of the reaction mixture. Work up: filtration through short pad of silica. Purification: neutralized SiO_2-chromatography (20 g; toluene, within 5 min). Results: *syn*-**3ha**, 174 mg, 94.5% yield. ^{1}H NMR ($CDCl_3$) δ 1.06 (s, 9H, $C(CH_3)_3$), 3.88 (s, 3H, OCH_3), 4.41 (dd, *J* = 7.20, 11.4 Hz, 1H, PhC*H*CH=CH_2), 4.75 (d, *J* = 11.4 Hz, 1H, COCH), 4.94 (d, *J* = 4.80 Hz, 1H, PhCHCH=CH*H*), 4.96 (s, 1H, PhCHCH=C*H*H) , 5.89 (ddd, *J* = 3.60, 4.80, 7.20 Hz, 1H, PhCHC*H*=CH_2), 6.96 (d, *J* = 9.00 Hz, 2H, ArH), 7.22–7.32 (m, 5H, ArH), 8.11 (d, *J* = 9.00 Hz, 2H, ArH); ^{13}C NMR ($CDCl_3$) δ 27.56, 49.11, 55.65, 60.09, 81.91, 114.00, 115.93, 127.00, 128.52, 128.98, 130.23, 131.32, 139.15, 140.69, 164.03, 166.94, 191.66; HRMS (ESI) calcd for $C_{23}H_{26}NaO_4$ [M+Na$^+$] 389.1729, found 389.1726; $[\alpha]_D^{23}$ –41.0534 (*c* 0.468, $CHCl_3$).

Enantiomer ratio: 99.8:0.2 (4.60 mmϕ x 250 mm DAICEL CHIRALPAK IB and 4.60 mmϕ x 250 mm DAICEL CHIRALPAK IB-3 columns; 0.5:99.5 2-PrOH–Hex eluent; 1.00 mL/min flow rate; 220-nm detection; 25 °C; t_R, 26.3 min (major), 31.3 min (minor)). **Supplementary Figures 23** and **24** showed the ^{1}H- and ^{13}C-NMR spectra and HPLC charts, respectively.

<u>*tert*-Butyl (2*R*,3*S*)-2-(4-chlorobenzoyl)-3-phenylpent-4-enoate (*syn*-**3ia**)</u>

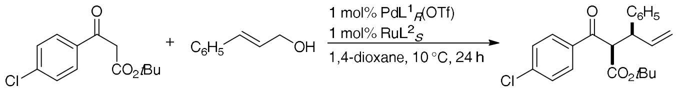

Reaction conditions (**Table 2**, entry 9): β-keto ester **1i** (127 mg, 0.500 mmol); allylic alcohol **2a** (0.600 M in 1,4-dioxane, 1.00 mL, 0.600 mmol, 1.20 mol amt); [RuCp(CH_3CN)$_3$]PF_6 (2.16 mg, 4.99 μmol, 1.00 mol%); **L**^2_S (2.73 mg, 5.01 μmol, 1.00 mol%); Pd**L**^1_R(OTf) (5.31 mg, 5.00 μmol, 1.00 mol%); 10 °C; 24 h. After 24 h: >99% conversion, branch/linear = 98.9:1.1, *syn*:*anti* = 99.7:0.3. **Supplementary Figure 25** showed a ^{1}H-NMR spectrum of the reaction mixture. Work up: filtration through short pad of silica. Purification: neutralized SiO_2-chromatography (20 g; toluene, within 5 min). Results: *syn*-**3ia**, 170 mg, 91.7% yield. ^{1}H NMR ($CDCl_3$) δ 1.06 (s, 9H, C(CH_3)$_3$), 4.39 (dd, *J* = 7.20, 10.8 Hz, 1H, PhC*H*CH=CH_2), 4.73 (d, *J* = 10.8 Hz, 1H, COCH), 4.95 (d, *J* = 10.8 Hz, 1H, PhCHCH=CH*H*), 4.98 (d, *J* = 4.80 Hz, 1H, PhCHCH=C*H*H) , 5.88 (ddd, *J* = 4.80, 7.20 Hz, 10.8 Hz, 1H, PhCHC*H*=CH_2), 7.23–7.32 (m, 5H, ArH), 7.47 (d, *J* = 8.40 Hz, 2H, ArH), 8.06 (d, *J* = 8.40 Hz, 2H, ArH); ^{13}C NMR ($CDCl_3$) δ 27.51, 49.13, 60.46, 82.35, 116.19, 127.16, 128.59, 128.89, 129.18, 130.30, 135.33, 138.80, 140.24, 166.49, 192.17; HRMS (ESI) calcd for $C_{22}H_{23}ClNaO_3$ [M+Na$^+$] 393.1233, found 393.1228; $[\alpha]_D^{23}$ –31.668 (*c* 0.482, $CHCl_3$).

Enantiomer ratio: 99.7:0.3 (4.60 mmϕ x 250 mm DAICEL CHIRALPAK IG column; 1.0:99.0 2-PrOH–Hex eluent; 1.00 mL/min flow rate; 220-nm detection; 25 °C; t_R, 16.5 min (2*R*,3*S*, major), 30.1 min (2*S*,3*R*, minor)). **Supplementary Figures 26** and **27** showed the ^{1}H- and ^{13}C-NMR spectra and HPLC charts, respectively.

The abs confign was determined by the X-ray crystallographic analysis of the product (2*R*,3*S*)-*syn*-**3ia**. White solid *syn*-**3ia** (10.0 mg, 99.0:1.0 dr, 99.7:0.3 er) was dissolved in a 1:5 $CHCl_3$/hexane mixture (2.00 mL) in a 5-mL glass tube with a screw cap and the solution was kept at –20 °C for 2 h, giving colorless block crystals (ca. 50% yield, mp 129 °C). The crystal was subjected to X-ray crystallographic analysis and the abs confign of *syn*-**3ia** derived from the Pd**L**^1_R(OTf) and Ru**L**^2_S-catalyzed allylation was determined to be 2*R*,3*S* (Flack parameter, 0.060(8) for (2*R*,3*S*)-*syn*-**3ia**). **Supplementary Table 3** summarized the crystallographic data, and the molecular structure in a crystal was shown in **Supplementary Figure 28**. Crystallographic data have been deposited with Cambridge Crystallographic Data Centre as supplementary publication no. CCDC-2193053.

<u>*tert*-Butyl (2*R**,3*R**)-2-acetyl-2-methyl-3-phenylpent-4-enoate (*syn*-**3ja**)</u>

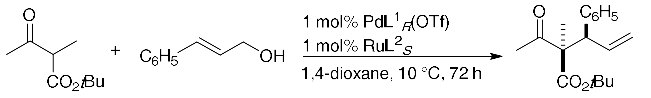

Reaction conditions (**Table 2**, entry 10): β-keto ester **1j** (86.2 mg, 0.500 mmol); allylic alcohol **2a** (0.500 M in 1,4-dioxane, 2.00 mL,1.00 mmol, 2.00 mol amt); [RuCp(CH_3CN)$_3$]PF_6 (2.17 mg, 5.00 μmol, 1.00 mol%); **L**^2_S (2.73 mg, 5.00 μmol, 1.00 mol%); Pd**L**^1_R(OTf) (5.31 mg, 5.00 μmol, 1.00 mol%); 10 °C; 72 h. After 72 h: 92.5% conversion, branch/linear = >99.9:<0.1, *syn*:*anti* = 99.1:0.90. **Supplementary Figure 29** showed a ^{1}H-NMR spectrum of the reaction mixture. Work up: filtration through short pad of silica. Purification: neutralized SiO_2-chromatography (20 g; toluene). Results: **3ja**, 131 mg, 90.8% yield. ^{1}H NMR ($CDCl_3$) δ 1.29 (s, 9H, C(CH_3)$_3$), 1.38 (s, 3H, CH_3), 2.18 (s, 3H, CH_3CO), 4.28 (d, *J* = 8.40 Hz, 1H, PhC*H*CH=CH_2), 5.04 (d, *J* = 17.2 Hz, 1H, PhCHCH=CH*H*), 5.12 (d, *J* = 10.2 Hz, 1H, PhCHCH=C*H*H), 6.13 (ddd, 1H, *J* = 8.40, 10.2,

17.2 Hz, 1H, PhCHC*H*=CH_2), 7.20–7.28 (m, 5H, ArH); ^{13}C NMR ($CDCl_3$) δ 17.06, 27.33, 27.77, 52.85, 64.92, 82.19, 117.90, 127.03, 128.22, 129.97, 137.37, 139.88, 170.47, 204.96; HRMS (ESI) calcd for $C_{18}H_{24}NaO_3$ [M+Na$^+$] 311.1623, found 311.1620; $[\alpha]_D^{23}$ –22.316 (*c* 0.484, $CHCl_3$).

Enantiomer ratio: 99.9:0.1 (4.60 mmϕ x 250 mm DAICEL CHIRALPAK IE-3 column; 0.2:99.8 2-PrOH–Hex eluent; 1.00 mL/min flow rate; 220-nm detection; 25 °C; t_R, 19.7 min (major), 20.3 min (minor)). **Supplementary Figures 30** and **31** showed the ^{1}H- and ^{13}C-NMR spectra and HPLC charts, respectively.

tert-Butyl 2-acetyl-2-benzyl-3-phenylpent-4-enoate (*syn*-**3ka**)

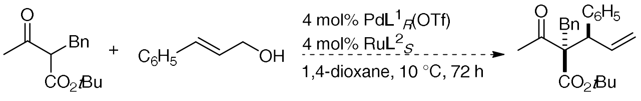

1k **2a** *syn*-**3ka**

Reaction conditions (**Table 2**, entry 11): β-keto ester **1k** (124 mg, 0.500 mmol); allylic alcohol **2a** (0.600 M in 1,4-dioxane, 1.00 mL, 0.600 mmol, 1.20 mol amt); [RuCp(CH_3CN)$_3$]PF_6 (8.67 mg, 20.0 μmol, 4.00 mol%); $\mathbf{L^2}_S$ (10.90 mg, 20.0 μmol, 4.00 mol%); Pd$\mathbf{L^1}_R$(OTf) (21.2 mg, 20.0 μmol, 4.00 mol%); 10 °C; 24 h. Work up: filtration through short pad of silica. Results: <1% conversion.

tert-Butyl (2*S**,3*R**)-2-acetyl-2-fluoro-3-phenylpent-4-enoate (*syn*-**3la**)

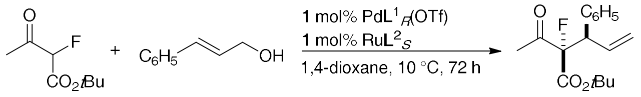

1l **2a** *syn*-**3la**

Reaction conditions (**Table 2**, entry 12): β-keto ester **1l** (88.1 mg, 0.500 mmol); allylic alcohol **2a** (0.500 M in 1,4-dioxane, 2.00 mL,1.00 mmol, 2.00 mol amt); [RuCp(CH_3CN)$_3$]PF_6 (2.17 mg, 5.00 μmol, 1.00 mol%); $\mathbf{L^2}_S$ (2.73 mg, 5.00 μmol, 1.00 mol%); Pd$\mathbf{L^1}_R$(OTf) (5.31 mg, 5.00 μmol, 1.00 mol%); 10 °C; 72 h. After 72 h: 92.4% conversion, branch/linear = >99.9:<0.1, *syn*:*anti* = 97.6:2.4. **Supplementary Figure 32** showed a ^{1}H-NMR spectrum of the reaction mixture. Work up: filtration through short pad of silica. Purification: neutralized SiO_2-chromatography (20 g; toluene, within 5 min). Results: *syn*-**3la**, 130 mg, 88.7% yield. ^{1}H NMR ($CDCl_3$) δ 1.17 (s, 9H, C(CH_3)$_3$), 2.32 (d, *J* = 5.40 Hz, 3H, CH_3), 4.28 (dd, *J* = 9.00, 33.0 Hz, 1H, PhC*H*CH=CH_2), 5.10 (d, *J* = 18.6 Hz, 1H, PhCHCH=CH*H*), 5.14 (d, *J* = 10.8 Hz, 1H, PhCHCH=C*H*H), 6.13 (ddd, *J* = 9.0, 10.8, 18.6 Hz, 1H, PhCHC*H*=CH_2), 7.23–7.36 (m, 5H, ArH); ^{13}C NMR ($CDCl_3$) δ 26.85, 27.52, 54.06 (d, *J* = 15.5 Hz), 84.16, 102.98 (d, *J* = 204 Hz), 119.25, 127.58, 128.63, 129.26, 134.50 (d, *J* = 4.4 Hz), 137.71, 163.56 (d, *J* = 25.8 Hz), 201.85 (d, *J* = 30.2 Hz); HRMS (ESI) calcd for $C_{17}H_{21}FNaO_3$ [M+Na$^+$] 315.1372, found 315.1373; $[\alpha]_D^{21}$ –1.911 (*c* 0.605, $CHCl_3$).

Enantiomer ratio: 98.9:1.1 er (20.0 mmϕ x 250 mm DAICEL Chiralcel OD-H column; 0.5:99.5 2-PrOH–Hex eluent; 3.00 mL/min flow rate; detection, 220-nm light; t_R, 33.2 min (major), 36.6 min (minor)). **Supplementary Figures 33** and **34** showed the ^{1}H- and ^{13}C-NMR spectra and HPLC charts, respectively.

tert-Butyl (*S**)-2-oxo-1-((*R**)-1-phenylallyl)cyclopentane-1-carboxylate (*syn*-**3ma**)

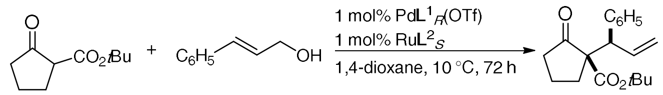

1m **2a** *syn*-**3ma**

Reaction conditions (**Table 2**, entry 13): β-keto ester **1m** (92.1 mg, 0.500 mmol); allylic alcohol **2a** (0.500 M in 1,4-dioxane, 2.00 mL, 1.00 mmol, 2.00 mol amt); [RuCp(CH_3CN)$_3$]PF_6 (2.17 mg, 5.00 μmol, 1.00 mol%); $\mathbf{L^2}_S$ (2.73 mg, 5.00 μmol, 1.00

mol%); Pd**L**^{1}*R*(OTf) (5.31 mg, 5.00 μmol, 1.00 mol%); 25 °C; 24 h. After 24 h: 98.1% conversion, branch/linear = >99.9:<0.1, *syn*:*anti* = 98.6:1.4. **Supplementary Figure 35** showed a ^{1}H-NMR spectrum of the reaction mixture. Work up: filtration through short pad of silica. Purification: SiO_2-chromatography (20 g; toluene). Results: **3ma**, 147 mg, 97.3% yield. ^{1}H NMR ($CDCl_3$) δ 1.24 (s, 9H, $C(CH_3)_3$), 1.82–1.92 (m, 2H, CH_2), 2.03–2.18 (m, 2H, CH_2), 2.36–2.51 (m, 2H, CH_2), 2.62–2.65 (m, 2H, CH_2), 4.21 (d, *J* = 7.80 Hz, 1H, PhC*H*CH=CH_2), 4.96 (d, *J* = 16.8 Hz, 1H, PhCHCH=C*H*H), 5.08 (d, *J* = 10.2 Hz, 1H, PhCHCH=CH*H*), 6.05 (ddd, *J* = 7.80, 10.2, 16.8 Hz, 1H, PhCHC*H*=CH_2), 7.19–7.28 (m, 5H, ArH); ^{13}C NMR ($CDCl_3$) δ 19.87, 27.71, 30.40, 53.73, 66.50, 82.14, 118.05, 126.97, 128.45, 129.34, 137.49, 139.97, 168.22, 213.89; HRMS (ESI) calcd for $C_{19}H_{24}FNaO_3$ [M+Na^{+}] 323.1623, found 323.1622; $[\alpha]_D^{22}$ –82.123 (*c* 0.448, $CHCl_3$).

Enantiomer ratio: 99.9:0.1 (4.60 mmϕ x 250 mm DAICEL CHIRALPAK IB and 4.60 mmϕ x 250 mm DAICEL CHIRALPAK IB-3 columns; 0.2:99.8 2-PrOH–Hex eluent; 0.50 mL/min flow rate; 254-nm detection; 25 °C; t_R, 25.1 min (major), 27.3 min (minor)). **Supplementary Figures 36** and **37** showed the ^{1}H- and ^{13}C-NMR spectra and HPLC charts, respectively.

tert-Butyl (2*R*,3*S*)-2-acetyl-3-(2-fluorophenyl)pent-4-enoate (*syn*-**3ab**)

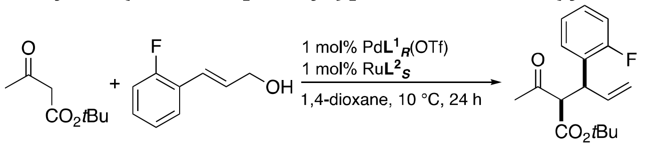

1a **2b** *syn*-**3ab**

Reaction conditions (**Table 2**, entry 14): β-keto ester **1a** (81.5 μL, 79.0 mg, 0.500 mmol); allylic alcohol **2a** (0.600 M in 1,4-dioxane, 1.00 mL, 0.600 mmol, 1.20 mol amt); $[RuCp(CH_3CN)_3]PF_6$ (2.16 mg, 4.99 μmol, 1.00 mol%); **L**^{2}*S* (2.74 mg, 5.03 μmol, 1.00 mol%); Pd**L**^{1}*R*(OTf) (5.31 mg, 5.00 μmol, 1.00 mol%); 10 °C; 24 h. After 24 h: 99% conversion, branch/linear = >99.9:<0.1, *syn*:*anti* = 98.8:1.2. **Supplementary Figure 38** showed a ^{1}H-NMR spectrum of the reaction mixture. Work up: filtration through short pad of silica. Purification: neutralized SiO_2-chromatography (20 g; toluene, within 5 min). Results: **3ab**, 144 mg, 99.8% yield. ^{1}H NMR ($CDCl_3$) δ 1.15 (s, 9H, $C(CH_3)_3$), 2.29 (s, 3H, CH_3CO), 4.04 (d, *J* = 11.4 Hz, 1H, COCH), 4.33 (dd, *J* = 7.80, 11.4 Hz, 1H, ArC*H*CH=CH_2), 5.05 (d, *J* = 4.20 Hz, 1H, ArCHCH=C*H*H), 5.07 (d, *J* = 10.8 Hz, 1H, ArCHCH=CH*H*), 5.88 (ddd, *J* = 4.20, 7.80, 10.8 Hz, 1H,ArCHC*H*=CH_2), 7.02–7.09 (m, 2H, ArH), 7.20–7.22 (m, 2H, ArH); ^{13}C NMR ($CDCl_3$) δ 27.54, 29.80, 43.80, 64.94, 76.95, 77.16, 77.37, 82.20, 115.89 (d, *J* = 21.7 Hz), 117.16, 124.29, 127.58 (d, *J* = 13.0 Hz), 128.72 (d, *J* = 7.2 Hz), 130.22 (d, *J* = 4.3 Hz), 136.92, 161.00 (d, *J* = 247.1 Hz), 166.62, 201.72; HRMS (ESI) calcd for $C_{17}H_{21}FNaO_3$ [M+Na^{+}] 315.1372, found 315.1378; $[\alpha]_D^{22}$ +35.861 (*c* 0.430, $CHCl_3$).

Enantiomer ratio: 99.7:0.3 (4.60 mmϕ x 250 mm DAICEL CHIRALPAK IA-3 column; 1.0:99.0 2-PrOH–Hex eluent; 0.50 mL/min flow rate; 220-nm detection; 25 °C; t_R, 14.2 min (2*R*,3*S*; major), 17.1 min (2*S*,3*R*; minor)). **Supplementary Figures 39** and **40** showed the ^{1}H- and ^{13}C-NMR spectra and HPLC charts, respectively.

The abs confign was determined by the X-ray crystallographic analysis of (2*R*,3*S*)-3-(2-fluorophenyl)-2-((*R*)-1-hydroxyethyl)pent-4-enoic acid ((1*R*,2*R*,3*S*)-$syn_{1,2}syn_{2,3}$-**11ab**), which was prepared by reduction of (2*R*,3*S*)-*syn*-**3ab** using K-selectride, followed by hydrolysis under acidic condition.

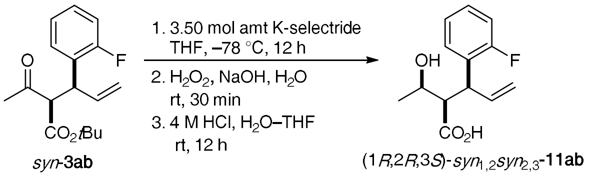

(2*R*,3*S*)-*syn*-**3ab** (73.1 mg, 0.250 mmol, 98.8:1.2 dr, 99.7:0.3 er) was dissolved in dry THF (2.50 mL) and cooled to −78 °C. To this mixture, 1.0 M solution of K-selectride in THF (875 μL, 0.875 mmol, 3.50 mol amt) was added dropwise and the reaction mixture was stirred at this temperature. After 12 h, 30% H_2O_2 aq. (1.00 mL) and 3.0 M NaOH aq. (1.00 mL) were added and the reaction mixture was slowly warmed to rt. The resulting solution was extracted with ether (3 x 15 mL). The organic layers were combined, washed with brine (20 mL), filtered through a pad of Celite, dried over Na_2SO_4, filtered, and concentrated. The product was used to next step without further purification. To a solution of the crude mixture in THF (1.00 mL) was added 4 M HCl aq. (4.00 mL) at rt. After 12 h at rt, the mixture was extracted with $CHCl_3$ (10.0 mL x 3). The organic extracts were dried over Na_2SO_4, filtered, and concentrated. The residue was purified by SiO_2-chromatography (ca. 20 g; 1:9 MeOH–$CHCl_3$ eluent) to give (1*R*,2*R*,3*S*)-*syn*$_{1,2}$*syn*$_{2,3}$-**11ab** (51.7 mg, 86.8% yield) as an off-white solid. ^{1}H NMR ($CDCl_3$) δ 1.26–1.29 (m, 3H, C*H*$_3$CHOH), 2.93 (d, *J* = 11.4 Hz, 1H, C*H*COOH), 4.07 (dd, *J* = 9.00, 11.4 Hz, 1H, ArC*H*CH=CH$_2$), 4.07–4.11 (m, 1H, CH$_3$C*H*OH), 5.17 (d, *J* = 9.60 Hz, 1H, ArCHCH=CH*H*), 5.27 (d, *J* = 16.8 Hz, 1H, ArCHCH=CH*H*), 5.94 (m, 1H, ArCHC*H*=CH$_2$), 6.98–7.05 (m, 2H, ArH), 7.16–7.20 (m, 2H, ArH); ^{13}C NMR ($CDCl_3$) δ 22.39, 45.08, 54.67, 65.69, 116.01 (d, *J* = 23.1 Hz), 118.46, 124.42 (d, *J* = 2.9 Hz), 127.96 (d, *J* = 13.0 Hz), 128.82 (d, *J* = 8.7 Hz), 129.91 (d, *J* = 5.8 Hz), 136.65, 160.92 (d, *J* = 247.1 Hz), 177.32; HRMS (ESI) calcd for $C_{13}H_{14}FO_3$ [M–H$^-$] 237.0932, found 237.0933; $[\alpha]_D^{21}$ −68.792 (*c* 0.566, $CHCl_3$). **Supplementary Figure 41** showed the ^{1}H- and ^{13}C-NMR spectra of (1*R*,2*R*,3*S*)-*syn*$_{1,2}$*syn*$_{2,3}$-**11ab**.

Recrystallization: (1*R*,2*R*,3*S*)-*syn*$_{1,2}$*syn*$_{2,3}$-**11ab** (10.0 mg); overlay of Hex (2 mL) on EtOAc solution of (1*R*,2*R*,3*S*)-*syn*$_{1,2}$*syn*$_{2,3}$-**11ab** (0.5 mL); −20 °C/12 h; crystallization yield, ca. 30%; mp 84.5 °C; Flack, 0.03(3) for (1*R*,2*R*,3*S*)-*syn*$_{1,2}$*syn*$_{2,3}$-**11ab**. **Supplementary Table 4** summarized the crystallographic data, and the molecular structure in a crystal was shown in **Supplementary Figure 42**. Crystallographic data have been deposited with Cambridge Crystallographic Data Centre as supplementary publication no. CCDC-2193054.

tert-Butyl (2*R**,3*S**)-2-acetyl-3-(*o*-tolyl)pent-4-enoate (*syn*-**3ac**)

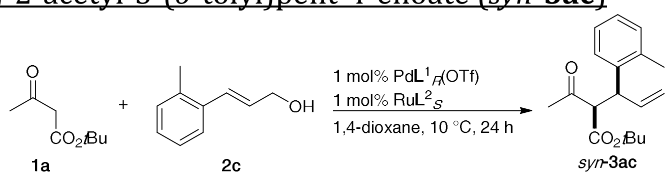

Reaction conditions (**Table 2**, entry 15): β-keto ester **1a** (81.5 μL, 79.0 mg, 0.500 mmol); allylic alcohol **2c** (0.600 M in 1,4-dioxane, 1.00 mL, 0.600 mmol, 1.20 mol amt); [RuCp(CH$_3$CN)$_3$]PF$_6$ (2.16 mg, 4.99 μmol, 1.00 mol%); **L^2*$_S$*** (2.73 mg, 5.01 μmol, 1.00 mol%); Pd**L^1*$_R$***(OTf) (5.31 mg, 5.00 μmol, 1.00 mol%); 10 °C; 24 h. After 24 h: 97.5% conversion, branch/linear = >99.9:<0.1, *syn*:*anti* = 99.0:1.0. **Supplementary Figure 43** showed the ^{1}H-NMR spectrum of the reaction mixture. Work up: filtration through short pad of silica. Purification: neutralized SiO_2-chromatography (20 g; toluene, within 5 min). Results: **3ac**, 140 mg, 96.5% yield, off-white solid. ^{1}H NMR ($CDCl_3$) δ 1.13 (s, 9H, C(CH$_3$)$_3$), 2.25 (s, 3H, CH$_3$CO), 2.32 (s, 3H, ArCH$_3$), 3.88 (d, *J* = 11.4 Hz, 1H, COCH), 4.02 (dd, *J* = 7.80, 11.4 Hz, 1H, ArC*H*CH=CHH), 5.02 (d, *J* = 4.20 Hz, 1H, ArCHCH=C*H*H), 5.04

(d, *J* = 10.2 Hz, 1H, ArCHCH=CH*H*), 5.86 (ddd, *J* = 4.20, 7.80, 10.2 Hz, 1H, ArCHC*H*=CH_2), 7.01–7.03 (m, 3H, ArH), 7.16–7.19 (m, 1H, ArH); ^{13}C NMR ($CDCl_3$) δ 21.52, 27.59, 29.52, 49.39, 66.45, 82.03, 116.22, 125.32, 127.77, 128.52, 129.18, 138.12, 138.66, 140.30, 166.88, 202.28; HRMS (ESI) calcd for $C_{18}H_{24}NaO_3$ [M+Na$^+$] 311.1623, found 311.1632. $[\alpha]_D^{23}$ +25.031 (*c* 0.733, $CHCl_3$).

Enantiomer ratio: 98.9:1.1 (4.60 mmϕ x 250 mm DAICEL CHIRALPAK ID-3 column; 1.0:99.0 2-PrOH–Hex eluent; 1.00 mL/min flow rate; 254-nm detection; 25 °C; t_R, 8.22 min (major), 9.46 min (minor)). **Supplementary Figures 44** and **45** showed the ^{1}H- and ^{13}C-NMR spectra and HPLC charts, respectively.

tert-Butyl (2*R**,3*S**)-2-acetyl-3-(2-methoxyphenyl)pent-4-enoate (*syn*-**3ad**)

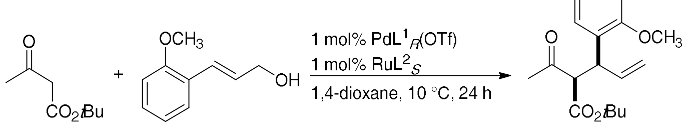

1a 2d *syn*-3ad

Reaction conditions (**Table 2**, entry 16): β-keto ester **1a** (81.5 μL,79.0 mg, 0.500 mmol); allylic alcohol **2d** (0.600 M in 1,4-dioxane, 1.00 mL, 0.600 mmol, 1.20 mol amt); $[RuCp(CH_3CN)_3]PF_6$ (2.16 mg, 4.99 μmol, 1.00 mol%); $\mathbf{L^2}_S$ (2.73 mg, 5.01 μmol, 1.00 mol%); Pd$\mathbf{L^1}_R$(OTf) (5.31 mg, 5.00 μmol, 1.00 mol%); 10 °C; 24 h. After 24 h: >99% conversion, branch/linear = >99.9:<0.1, *syn*:*anti* = 99.6:0.4. **Supplementary Figure 46** showed a ^{1}H-NMR spectrum of the reaction mixture. Work up: filtration through short pad of silica. Purification: neutralized SiO_2-chromatography (20 g; toluene, within 5 min). Results: **3ad**, 146 mg, 95.9% yield. ^{1}H NMR ($CDCl_3$) δ 1.13 (s, 9H, $C(CH_3)_3$), 2.29 (s, 3H, CH_3CO), 3.85 (s, 3H, $ArOCH_3$), 4.16 (d, 1H, *J* = 10.8 Hz, COCH), 4.35 (dd, *J* = 7.80, 10.8 Hz, 1H, ArC*H*CH=CHH), 4.98 (d, *J* = 9.60 Hz, 1H, ArCHCH=C*H*H), 5.05 (d, *J* = 16.8 Hz, 1H, ArCHCH=CH*H*), 5.97 (ddd, *J* = 7.80, 9.60, 16.8 Hz, 1H, ArCHC*H*=CH_2), 6.84–6.90 (m, 2H, ArH), 7.14–7.19 (m, 2H, ArH); ^{13}C NMR ($CDCl_3$) δ 27.55, 29.64, 45.28, 55.53, 64.76, 81.64, 111.11, 116.27, 120.72, 128.17, 128.73, 129.79, 137.81, 157.47, 167.16, 202.80; HRMS (ESI) calcd for $C_{18}H_{24}NaO_4$ [M+Na$^+$] 327.1572, found 327.1571; $[\alpha]_D^{23}$ +29.207 (*c* 0.454, $CHCl_3$).

Enantiomer ratio: 99.9:0.1 (4.60 mmϕ x 250 mm DAICEL CHIRALPAK ID-3 column; 1.0:99.0 2-PrOH–Hex eluent; 0.50 mL/min flow rate; 220-nm detection; 25 °C; t_R, 20.8 min (major), 22.3 min (minor)). **Supplementary Figures 47** and **48** showed the ^{1}H- and ^{13}C-NMR spectra and HPLC charts, respectively.

tert-Butyl (2*R*,3*S*)-2-acetyl-3-(*o*-tolyl)pent-4-enoate (**3ae**)

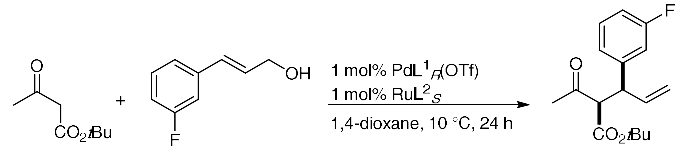

1a 2e *syn*-3ae

Reaction conditions (**Table 2**, entry 17): β-keto ester **1a** (81.5 μL, 79.0 mg, 0.500 mmol); allylic alcohol **2e** (0.600 M in 1,4-dioxane, 1.00 mL, 0.600 mmol, 1.20 mol amt); $[RuCp(CH_3CN)_3]PF_6$ (2.16 mg, 4.99 μmol, 1.00 mol%); $\mathbf{L^2}_S$ (2.73 mg, 5.01 μmol, 1.00 mol%); Pd$\mathbf{L^1}_R$(OTf) (5.31 mg, 5.00 μmol, 1.00 mol%); 10 °C; 24 h. After 24 h: >99% conversion, branch/linear = >99.9:<0.1, *syn*:*anti* = 99.0:1.0. **Supplementary Figure 49** showed a ^{1}H-NMR spectrum of the reaction mixture. Work up: filtration through short pad of silica. Purification: neutralized SiO_2-chromatography (20 g; toluene, within 5 min). Results: **3ac**, 145.8 mg, 99.7% yield. ^{1}H NMR ($CDCl_3$) δ 1.17 (s, 9H, $C(CH_3)_3$), 2.29 (s, 3H, CH_3CO), 3.87 (d, *J* = 10.8 Hz, 1H, COCH), 4.08 (dd, *J* = 7.80, 10.8 Hz, 1H,

ArC*H*CH=CH_2), 5.04 (d, *J* = 17.4 Hz, 1H, ArCHCH=C*H*H), 5.06 (d, *J* = 11.4 Hz, 1H, ArCHCH=CH*H*), 5.85 (ddd, *J* = 7.80, 11.4, 17.4 Hz, 1H, ArCHC*H*=CH_2), 6.90–6.95 (m, 3H, ArH), 7.01–7.02 (m, 1H, ArH); ^{13}C NMR ($CDCl_3$) δ 27.61, 29.66, 48.81, 66.08, 82.35, 113.94 (d, *J* = 21.5 Hz), 115.37 (d, *J* = 20.1 Hz), 116.89, 124.09, 130.08 (d, *J* = 7.2 Hz), 137.92, 143.03 (d, *J* = 7.2 Hz), 162.92 (d, *J* = 243.9 Hz), 166.54, 201.60; HRMS (ESI) calcd for $C_{17}H_{21}FNaO_3$ [M+Na$^+$] 315.1372, found 315.1375; $[\alpha]_D^{22}$ +29.502 (*c* 0.666, $CHCl_3$).

Enantiomer ratio: 99.9:0.1 (4.60 mmϕ x 250 mm DAICEL CHIRALPAK IA column; 1.0:99.0 2-PrOH–Hex eluent; 0.50 mL/min flow rate; 220-nm detection; 25 °C; t_R, 14.5 min (2*R*,3*S*; major), 16.1 min (2*S*,3*R*; minor)). **Supplementary Figures 50** and **51** showed the ^1H- and ^{13}C-NMR spectra and HPLC charts, respectively.

The abs confign was determined by the X-ray crystallographic analysis of (2*R*,3*S*)-3-(2-fluorophenyl)-2-((*R*)-1-hydroxyethyl)pent-4-enoic acid ((1*R*,2*R*,3*S*)-*syn*$_{1,2}$*syn*$_{2,3}$-**11ae**), which was prepared by reduction of (2*R*,3*S*)-*syn*-**3ae** using K-selectride, followed by hydrolysis under acidic condition.

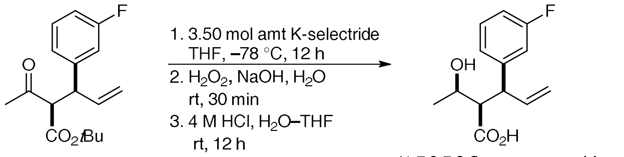

(2*R*,3*S*)-*syn*-**3ae** (73.0 mg, 0.249 mmol, 99.0:1.00 dr, 99.9:0.1 er) was dissolved in dry THF (2.50 mL) and cooled to −78 °C. To this mixture, 1.0 M solution of K-selectride in THF (875 μL, 0.875 mmol, 3.50 mol amt) was added dropwise and the reaction mixture was stirred at this temperature. After 12 h, 30% H_2O_2 aq. (1.00 mL) and 3.0 M NaOH aq. (1.00 mL) were added, and the reaction mixture was slowly warmed to rt. The resulting solution was extracted with ether (3 x 5 mL). The organic layers were combined, washed with brine (20 mL), filtered through a pad of Celite, dried over Na_2SO_4, filtered and concentrated. The residue was dissolved to THF (1.00 mL) and to this was added 4 M HCl aq. (4.00 mL) at rt. After 12 h at rt, the mixture was extracted with $CHCl_3$ (10 mL x 3). The organic extracts were dried over Na_2SO_4, filtered, and concentrated. The residue was purified by SiO_2-chromatography (ca. 40 g; 1:9 MeOH–$CHCl_3$ eluent) to give (1*R*,2*R*,3*S*)-*syn*$_{1,2}$*syn*$_{2,3}$-**11ae** (55.8 mg, 94.4% yield) as an off-white solid. ^1H NMR ($CDCl_3$) δ 1.29 (d, *J* = 6.00 Hz, 3H, C*H*$_3$CH(OH)), 2.75 (dd, *J* = 3.00, 10.8 Hz, 1H, C*H*COOH), 3.86 (dd, *J* =10.2, 10.8 Hz, 1H, ArC*H*CH=CH_2), 4.10–4.11 (m, 1H, C*H*OH), 5.18 (d, *J* = 10.2 Hz, 1H, ArCHCH=CH*H*), 5.25 (d, *J* = 16.8 Hz, 1H, ArCHCH=CH*H*), 5.90 (ddd, *J* = 10.2, 10.2, 16.8 Hz, 1H, ArCHC*H*=CH_2) , 6.89–6.99 (m, 3H, ArH), 7.22–7.24 (m, 1H, ArH); ^{13}C NMR ($CDCl_3$) δ 22.42, 49.52, 56.15, 65.73, 114.88 (d, *J* = 20.1 Hz), 114.95 (d, *J* = 21.5 Hz), 118.14, 123.63, 130.28 (d, *J* = 34.2 Hz), 137.72, 143.75 (d, *J* = 7.2 Hz), 163.48 (d, *J* = 93.9 Hz), 176.62; HRMS (ESI) calcd for $C_{13}H_{14}FO_3$ [M−H$^-$] 237.0932, found 237.0938; $[\alpha]_D^{21}$ −63.321 (*c* 0.674, $CHCl_3$). **Supplementary Figure 52** showed the ^1H- and ^{13}C-NMR spectra of (1*R*,2*R*,3*S*)-*syn*$_{1,2}$*syn*$_{2,3}$-**11ae**.

Recrystallization: (1*R*,2*R*,3*S*)-*syn*$_{1,2}$*syn*$_{2,3}$-**11ae** (10.0 mg); overlay of Hex (2 mL) on $CHCl_3$ solution of (1*R*,2*R*,3*S*)-*syn*$_{1,2}$*syn*$_{2,3}$-**11ae** (0.5 mL); −20 °C/12 h; crystallization yield, ca. 50%; mp 89.0 °C; Flack, −0.01(7) for (1*R*,2*R*,3*S*)-*syn*$_{1,2}$*syn*$_{2,3}$-**11ae**. **Supplementary Table 5** summarized the crystallographic data, and the molecular structure in a crystal was shown in **Supplementary Figure 53**. Crystallographic data have been deposited with Cambridge Crystallographic Data Centre as supplementary publication no. CCDC-2193059.

tert-Butyl (2R^*,3S^*)-2-acetyl-3-(m-tolyl)pent-4-enoate (syn-**3af**)

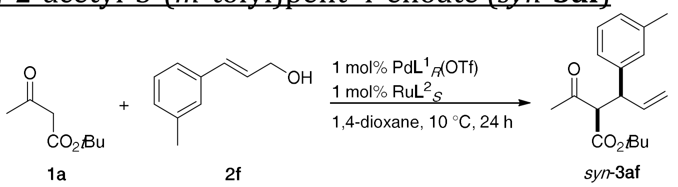

Reaction conditions (**Table 2**, entry 18): β-keto ester **1a** (81.5 μL, 79.0 mg, 0.500 mmol); allylic alcohol **2f** (0.600 M in 1,4-dioxane, 1.00 mL, 0.600 mmol, 1.20 mol amt); [RuCp(CH_3CN)$_3$]PF_6 (2.16 mg, 4.99 μmol, 1.00 mol%); $\mathbf{L}^2_S$ (2.73 mg, 5.01 μmol, 1.00 mol%); Pd$\mathbf{L}^1_R$(OTf) (5.31 mg, 5.00 μmol, 1.00 mol%); 10 °C; 24 h. After 24 h: >99% conversion, branch/linear = >99.9:<0.1, *syn*:*anti* = 99.3:0.7. **Supplementary Figure 54** showed a ^{1}H-NMR spectrum of the reaction mixture. Work up: filtration through short pad of silica. Purification: neutralized SiO_2-chromatography (20 g; toluene, within 5 min). Results: **3af**, 142 mg, 98.1% yield. ^{1}H NMR ($CDCl_3$) δ 1.15 (s, 9H, C(CH_3)$_3$), 2.29 (s, 3H, CH_3CO), 2.32 (s, 3H, ArCH_3), 3.88 (d, *J* = 10.8 Hz, 1H, COCH), 4.02 (dd, *J* = 8.40, 10.8 Hz, 1H, ArC*H*CH=CH_2), 5.02 (d, *J* = 4.20 Hz, 1H, ArCHCH=C*H*H), 5.04 (d, *J* = 10.8 Hz, 1H, ArCHCH=CH*H*), 5.87 (ddd, *J* = 4.20, 8.40, 10.8 Hz, 1H, ArCHC*H*=CH_2), 7.01–7.03 (m, 3H, ArH), 7.16–7.19 (m, 1H, ArH); ^{13}C NMR ($CDCl_3$) δ 21.52, 27.59, 29.52, 49.39, 66.44, 82.02, 116.21, 125.32, 127.76, 128.52, 129.18, 138.12, 138.65, 140.30, 166.88, 202.28; HRMS (ESI) cacld for $C_{18}H_{24}NaO_3$ [M+Na$^+$] 311.1623, found 311.1629; $[\alpha]_D^{22}$ +29.976 (*c* 0.590, $CHCl_3$).

Enantiomer ratio: 99.9:0.1 (4.60 mmϕ x 250 mm DAICEL CHIRALPAK ID-3 column; 1.0:99.0 2-PrOH–Hex eluent; 0.50 mL/min flow rate; 220-nm detection; 25 °C; t_R, 16.7 min (major), 19.5 min (minor)). **Supplementary Figures 55** and **56** showed the ^{1}H- and ^{13}C-NMR spectra and HPLC charts, respectively.

tert-Butyl (2R^*,3S^*)-2-acetyl-3-(3-methoxyphenyl)pent-4-enoate (syn-**3ag**)

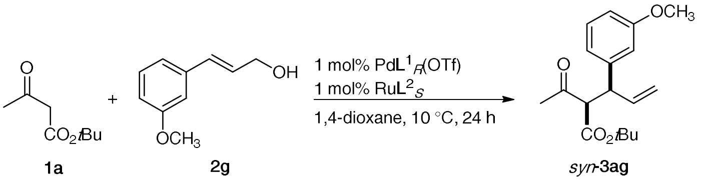

Reaction conditions (**Table 2**, entry 19): β-keto ester **1a** (81.5 μL, 79.0 mg, 0.500 mmol); allylic alcohol **2g** (0.600 M in 1,4-dioxane, 1.00 mL, 0.600 mmol, 1.20 mol amt); [RuCp(CH_3CN)$_3$]PF_6 (2.16 mg, 4.99 μmol, 1.00 mol%); $\mathbf{L}^2_S$ (2.73 mg, 5.01 μmol, 1.00 mol%); Pd$\mathbf{L}^1_R$(OTf) (5.31 mg, 5.00 μmol, 1.00 mol%); 10 °C; 24 h. After 24 h: >99% conversion, branch/linear = >99.9:<0.1, *syn*:*anti* = 99.8:0.2. **Supplementary Figure 57** showed a ^{1}H-NMR spectrum of the reaction mixture. Work up: filtration through short pad of silica. Purification: neutralized SiO_2-chromatography (20 g; toluene, within 5 min). Results: **3ag**, 147 mg, 96.4 % yield. ^{1}H NMR ($CDCl_3$) δ 1.17 (s, 9H, C(CH_3)$_3$), 2.29 (s, 3H, CH_3CO), 3.79 (s, 3H, ArOCH_3), 3.88 (d, *J* = 11.4 Hz, 1H, COCH), 4.04 (dd, *J* = 7.80, 11.4 Hz, 1H, ArC*H*CH=CH_2), 5.03 (d, *J* = 10.2 Hz, 1H, ArCHCH=C*H*H), 5.05 (d, *J* = 9.60 Hz, 1H, ArCHCH=CH*H*), 5.87 (ddd, *J* = 7.80, 9.60, 10.2 Hz, 1H, ArCHC*H*=CH_2), 6.75–6.82 (m, 3H, ArH), 7.19–7.22 (m, 1H, ArH); ^{13}C NMR ($CDCl_3$) δ 27.63, 29.58, 49.38, 55.34, 66.33, 82.11, 112.53, 114.13, 116.42, 120.66, 129.61, 138.42, 141.99, 159.79, 166.78, 202.15; HRMS (ESI) calcd for $C_{18}H_{24}NaO_4$ [M+Na$^+$] 327.1572, found 327.1574; $[\alpha]_D^{22}$ +27.673 (*c* 0.477, $CHCl_3$).

Enantiomer ratio: 99.8:0.2 (4.60 mmϕ x 250 mm DAICEL CHIRALPAK ID-3 column; 1.0:99.0 2-PrOH–Hex eluent; 0.50 mL/min flow rate; 220-nm detection; 25 °C;

t_R, 29.9 min (major), 31.8 min (minor)). **Supplementary Figures 58** and **59** showed the ^{1}H- and ^{13}C-NMR spectra and HPLC charts, respectively.

<u>*tert*-Butyl (2*R*,3*S*)-2-acetyl-3-(4-fluorophenyl)pent-4-enoate (*syn*-**3ah**)</u>

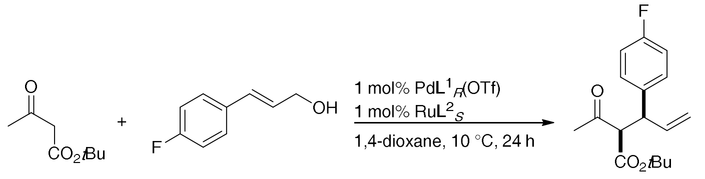

Reaction conditions (**Table 2**, entry 20): β-keto ester **1a** (81.5 μL, 79.0 mg, 0.500 mmol); allylic alcohol **2h** (0.600 M in 1,4-dioxane, 1.00 mL, 0.600 mmol, 1.20 mol amt); $[RuCp(CH_3CN)_3]PF_6$ (2.16 mg, 4.99 μmol, 1.00 mol%); $\mathbf{L^2}_S$ (2.73 mg, 5.01 μmol, 1.00 mol%); Pd$\mathbf{L^1}_R$(OTf) (5.31 mg, 5.00 μmol, 1.00 mol%); 10 °C; 24 h. After 24 h: >99% conversion, branch/linear = >99.9:<0.1, *syn*:*anti* = 99.3:0.7. **Supplementary Figure 60** showed a ^{1}H-NMR spectrum of the reaction mixture. Work up: filtration through short pad of silica. Purification: neutralized SiO_2-chromatography (20 g; toluene, within 5 min). Results: *syn*-**3ah**, 145 mg, 98.9% yield. ^{1}H NMR ($CDCl_3$) δ 1.17 (s, 9H, $C(CH_3)_3$), 2.29 (s, 3H, CH_3CO), 3.85 (d, J = 10.8 Hz, 1H, COCH), 4.07 (dd, J = 7.80, 10.8 Hz, 1H, ArC*H*CH=CH_2), 5.01 (d, J = 17.4 Hz, 1H, ArCHCH=C*H*H), 5.04 (d, J = 10.2 Hz, 1H, ArCHCH=CH*H*), 5.85 (ddd, J = 7.80, 10.2, 17.4 Hz, 1H, ArCHC*H*=CH_2), 6.98–7.01 (m, 2H, ArH), 7.18–7.20 (m, 2H, ArH); ^{13}C NMR ($CDCl_3$) δ 27.63, 29.57, 48.37, 66.45, 82.27, 115.41 (d, J = 21.7 Hz), 116.48, 129.95 (d, J = 7.2 Hz), 136.16 (d, J = 2.9 Hz), 138.39, 161.90 (d, J = 245.6 Hz), 166.68, 201.81; HRMS (ESI) calcd for $C_{17}H_{21}FNaO_3$ [M+Na^{+}] 315.1372, found 315.1377; $[\alpha]_D^{20}$ +2.538 (*c* 0.508, $CHCl_3$).

Enantiomer ratio: 99.4:0.6 (4.60 mmϕ x 250 mm DAICEL CHIRALPAK ID-3 column; 1.0:99.0 2-PrOH–Hex eluent; 0.50 mL/min flow rate; 220-nm detection; 25 °C; t_R, 16.1 min (2*R*,3*S*; major), 18.5 min (2*S*,3*R*; minor)). **Supplementary Figures 61** and **62** showed the ^{1}H- and ^{13}C-NMR spectra and HPLC charts, respectively.

The abs confign was determined by the X-ray crystallographic analysis of (2*R*,3*S*)-3-(4-fluorophenyl)-2-((*R*)-1-hydroxyethyl)pent-4-enoic acid ((1*R*,2*R*,3*S*)-*syn*$_{1,2}$*syn*$_{2,3}$-**11ah**), which was prepared by reduction of (2*R*,3*S*)-*syn*-**3ah** using K-selectride, followed by hydrolysis under acidic condition.

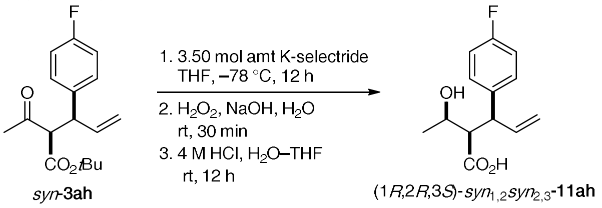

(2*R*,3*S*)-*syn*-**3ah** (73.2 mg, 0.250 mmol, 99.3:0.7 dr, 99.4:0.6 er) was dissolved in dry THF (2.50 mL) and cooled to −78 °C. To this mixture, 1.0 M solution of K-selectride in THF (875 μL, 0.875 mmol, 3.50 mol amt) was added dropwise and the reaction mixture was stirred at this temperature for 12 h. After 12 h, 30% H_2O_2 aq. (1.00 mL) and 3.0 M NaOH aq. (1.00 mL) were added and the reaction mixture was slowly warmed to rt. The resulting solution was extracted with ether (3 x 5 mL). The organic layers were combined, washed with brine (20 mL), filtered through a pad of Celite, dried over Na_2SO_4, filtered, and concentrated. The residue was dissolved to THF (1.00 mL) and to this was added 4 M HCl aq. (4.00 mL) at rt. After 12 h at rt, the mixture was extracted with $CHCl_3$ (10.0 mL x 3). The organic extracts were dried over Na_2SO_4, filtered, and concentrated. The residue was purified by SiO_2-chromatography (ca. 40 g; 1:9 MeOH–$CHCl_3$ eluent) to give (1*R*,2*R*,3*S*)-*syn*$_{1,2}$*syn*$_{2,3}$-**11ah** (55.8 mg, 94.4% yield) as an off-white

solid. ^{1}H NMR ($CDCl_3$) δ 1.29 (d, *J* = 6.60 Hz, 3H, C*H*$_3$CH(OH)), 2.74 (d, *J* = 11.4 Hz, 1H, C*H*COOH), 3.85 (dd, *J* = 9.60, 11.4 Hz, 1H, ArC*H*CH=CH_2), 4.10–4.11 (m, 1H, C*H*OH), 5.16 (d, *J* = 10.2 Hz, 1H, ArCHCH=CH*H*), 5.23 (d, *J* = 17.4 Hz, 1H, ArCHCH=CH*H*), 5.91 (ddd, *J* = 9.60, 10.2, 17.4 Hz, 1H, ArCHC*H*=CH_2) , 6.96–6.99 (m, 2H, ArH), 7.16–7.18 (m, 2H, ArH); ^{13}C NMR ($CDCl_3$) δ 22.44, 49.00 , 56.40 , 65.74, 115.66 (d, *J* = 21.5 Hz), 117.71, 129.49 (d, *J* = 7.2 Hz), 136.90, 138.20, 161.88 (d, *J* = 244.1 Hz), 176.62; HRMS (ESI) calcd for $C_{13}H_{14}FO_3$ [M–H$^-$] 237.0932, found 237.0940; $[\alpha]_D^{21}$ –79.978 (*c* 0.549, $CHCl_3$). **Supplementary Figure 63** showed the ^{1}H- and ^{13}C-NMR spectra of (1*R*,2*R*,3*S*)-*syn*$_{1,2}$*syn*$_{2,3}$-**11ah**.

Recrystallization: (1*R*,2*R*,3*S*)-*syn*$_{1,2}$*syn*$_{2,3}$-**11ah** (10.0 mg); overlay of Hex (2 mL) on $CHCl_3$ solution of (1*R*,2*R*,3*S*)-*syn*$_{1,2}$*syn*$_{2,3}$-**11ah** (0.5 mL); rt/12 h; crystallization yield, ca. 50%; mp 98.0 °C; Flack, 0.06(18) for (1*R*,2*R*,3*S*)-*syn*$_{1,2}$*syn*$_{2,3}$-**11ah**. **Supplementary Table 6** summarized the crystallographic data, and the molecular structure in a crystal was shown in **Supplementary Figure 64**. Crystallographic data have been deposited with Cambridge Crystallographic Data Centre as supplementary publication no. CCDC-2193062.

tert-Butyl (2*R**,3*S**)-2-acetyl-3-(4-chlorophenyl)pent-4-enoate (*syn*-**3ai**)

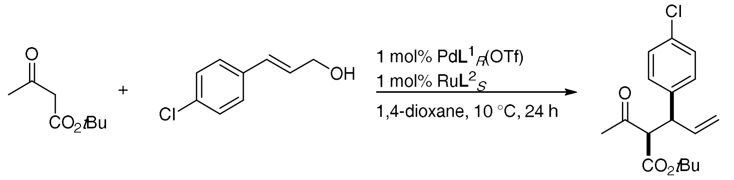

1a 2i *syn*-3ai

Reaction conditions (**Table 2**, entry 21): β-keto ester **1a** (81.5 μL, 79.0 mg, 0.500 mmol); allylic alcohol **2i** (0.600 M in 1,4-dioxane, 1.00 mL, 0.600 mmol, 1.20 mol amt); [RuCp(CH_3CN)$_3$]PF_6 (2.16 mg, 4.99 μmol, 1.00 mol%); **L**^{2}*S* (2.73 mg, 5.01 μmol, 1.00 mol%); Pd**L**^{1}*R*(OTf) (5.31 mg, 5.00 μmol, 1.00 mol%); 10 °C; 24 h. After 24 h: >99% conversion, branch/linear = >99.9:<0.1, *syn*:*anti* = 99.1:0.9. **Supplementary Figure 65** showed a ^{1}H-NMR spectrum of the reaction mixture. Work up: filtration through short pad of silica. Purification: neutralized SiO_2-chromatography (20 g; toluene, within 5 min). Results: **3ai**, 149 mg, 96.5% yield. ^{1}H NMR ($CDCl_3$) δ 1.18 (s, 9H, C(CH_3)$_3$), 2.28 (s, 3H, CH_3CO), 3.86 (d, *J* = 10.8 Hz, 1H, COCH), 4.06 (dd, *J* = 7.20, 10.8 Hz, 1H, ArC*H*CH=CH_2), 5.01 (d, *J* = 16.8 Hz, 1H, ArCHCH=C*H*H), 5.05 (d, *J* = 10.2 Hz, 1H, ArCHCH=CH*H*), 5.84 (ddd, *J* = 7.20, 10.2, 16.8 Hz, 1H, ArCHC*H*=CH_2), 7.15–7.17 (m, 2H, ArH), 7.27–7.31 (m, 2H, ArH); ^{13}C NMR ($CDCl_3$) δ 27.70, 29.59, 48.51, 66.25, 82.39, 116.78, 128.76, 129.84, 132.90, 138.16, 139.10, 166.64, 201.60; HRMS (ESI) calcd for $C_{17}H_{21}ClNaO_3$ [M+Na$^+$] 331.1077, found 331.1079; $[\alpha]_D^{23}$ +25.976 (*c* 0.581, $CHCl_3$).

Enantiomer ratio: 99.9:0.1 (4.60 mmϕ x 250 mm DAICEL CHIRALPAK ID-3 column; 1.0:99.0 2-PrOH–Hex eluent; 0.50 mL/min flow rate; 220-nm detection; 25 °C; t_R, 16.3 min (major), 17.8 min (minor)). **Supplementary Figures 66** and **67** showed the ^{1}H- and ^{13}C-NMR spectra and HPLC charts, respectively.

tert-Butyl (2*R**,3*S**)-2-acetyl-3-(4-(trifluoromethyl)phenyl)pent-4-enoate (*syn*-**3aj**)

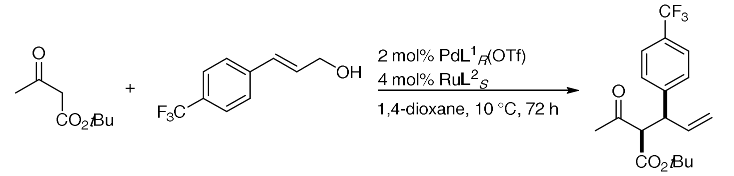

1a 2j *syn*-3aj

Reaction conditions (**Table 2**, entry 22): β-keto ester **1a** (81.5 μL, 79.0 mg, 0.500 mmol); allylic alcohol **2j** (0.600 M in 1,4-dioxane, 1.00 mL, 0.600 mmol, 1.20 mol amt);

$[RuCp(CH_3CN)_3]PF_6$ (8.68 mg, 20.0 μmol, 4.00 mol%); $\mathbf{L^2}_{S}$ (10.9 mg, 20.0 μmol, 4.00 mol%); Pd$\mathbf{L^1}_{R}$(OTf) (10.6 mg, 10.0 μmol, 2.00 mol%); 10 °C; 72 h. After 72 h: >99% conversion, branch/linear = 92.7:7.3, *syn*:*anti* = 98.4:1.6. **Supplementary Figure 68** showed a ^{1}H-NMR spectrum of the reaction mixture . Work up: filtration through short pad of silica. Purification: neutralized SiO_2-chromatography (20 g; toluene, within 5 min). Results: **3aj**, 149 mg, 87.0% yield. ^{1}H NMR ($CDCl_3$) δ 1.15 (s, 9H, $C(CH_3)_3$), 2.30 (s, 3H, CH_3CO), 3.91 (d, *J* = 10.8 Hz, 1H, COCH), 4.16 (dd, *J* = 7.80, 10.8 Hz, 1H, ArC*H*CH=CH_2), 5.04 (d, *J* = 11.4 Hz, 1H, ArCHCH=C*H*H), 5.08 (d, *J* = 10.2 Hz, 1H, ArCHCH=CH*H*), 5.86 (ddd, *J* = 7.80, 10.2, 11.4 Hz, 1H, ArCHC*H*=CH_2), 7.35 (d, *J* = 8.40 Hz, 2H, ArH), 7.56 (d, *J* = 8.40 Hz, 2H, ArH); ^{13}C NMR ($CDCl_3$) δ 27.59, 29.66, 29.72, 48.85, 65.94, 82.53, 117.27, 126.05 (q, *J* = 271 Hz), 125.57, 128.53, 128.55, 129.22 (q, *J* = 32.0 Hz), 137.69, 144.69, 166.49, 201.38.; HRMS (ESI) calcd for $C_{18}H_{21}F_3NaO_3$ $[M+Na^+]$ 365.1340, found 365.1335; $[\alpha]_D^{23}$ +31.159 (*c* 0.806, $CHCl_3$).

Enantiomer ratio: 99.1:0.9 (4.60 mmϕ x 250 mm DAICEL CHIRALPAK IG column; 0.5:99.5 2-PrOH–Hex eluent; 0.50 mL/min flow rate; 220-nm detection; 25 °C; t_R, 25.0 min (major), 30.1 min (minor)). **Supplementary Figures 69** and **70** showed the ^{1}H- and ^{13}C-NMR spectra and HPLC charts, respectively.

tert-Butyl (2*R**,3*S**)-2-acetyl-3-(*p*-tolyl)pent-4-enoate (*syn*-**3ak**)

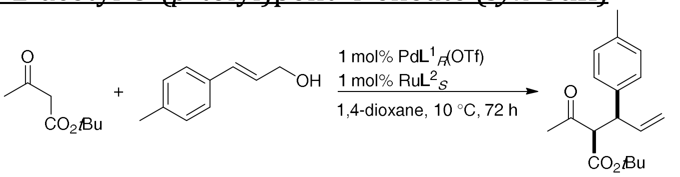

1a 2k *syn*-3ak

Reaction conditions (**Table 2**, entry 23): β-keto ester **1a** (81.5 μL, 79.0 mg, 0.500 mmol); allylic alcohol **2k** (0.600 M in 1,4-dioxane, 1.00 mL, 0.600 mmol, 1.20 mol amt); $[RuCp(CH_3CN)_3]PF_6$ (2.16 mg, 4.99 μmol, 1.00 mol%); $\mathbf{L^2}_{S}$ (2.73 mg, 5.01 μmol, 1.00 mol%); Pd$\mathbf{L^1}_{R}$(OTf) (5.31 mg, 5.00 μmol, 1.00 mol%); 10 °C; 24 h. After 24 h: >99% conversion, branch/linear = >99.9:<0.1, *syn*:*anti* = 99.4:0.6. **Supplementary Figure 71** showed a ^{1}H-NMR spectrum of the reaction mixture. Work up: filtration through short pad of silica. Purification: neutralized SiO_2-chromatography (20 g; toluene, within 5 min). Results: **3ak**, 142 mg, 98.4% yield. ^{1}H NMR ($CDCl_3$) δ 1.16 (s, 9H, $C(CH_3)_3$), 2.28 (s, 3H, CH_3CO), 2.30 (s, 3H, $ArCH_3$), 3.87 (d, *J* = 12.0 Hz, 1H, COCH), 4.03 (dd, *J* = 8.40, 12.0 Hz, 1H, ArC*H*CH=CH_2), 5.01 (d, *J* = 2.40 Hz, 1H, ArCHCH=C*H*H), 5.03 (d, *J* = 9.00 Hz, 1H, ArCHCH=CH*H*), 5.86 (ddd, *J* = 2.40, 9.00, 12.0 Hz, 1H, ArCHC*H*=CH_2), 7.09–7.11 (m, 4H, ArH); ^{13}C NMR ($CDCl_3$) δ 21.15, 27.62, 29.51, 49.00, 66.48, 82.02, 116.08, 128.20, 129.26 136.61, 137.35, 138.78, 166.87, 202.32; HRMS (ESI) calcd for $C_{18}H_{24}NaO_3$ $[M+Na^+]$ 311.1623, found 311.1628; $[\alpha]_D^{21}$ +17.614 (*c* 0.723, $CHCl_3$).

Enantiomer ratio: 99.2:0.8 (4.60 mmϕ x 250 mm DAICEL CHIRALPAK IA-3 column; 1.0:99.0 2-PrOH–Hex eluent; 0.50 mL/min flow rate; 220-nm detection; 25 °C; t_R, 13.1 min (major), 13.8 min (minor)). **Supplementary Figures 72** and **73** showed the ^{1}H- and ^{13}C-NMR spectra and HPLC charts, respectively.

tert-butyl (2*R**,3*S**)-2-acetyl-3-(4-methoxyphenyl)pent-4-enoate (*syn*-**3al**)

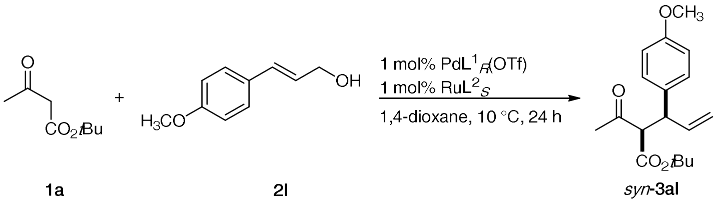

1a 2l *syn*-3al

Reaction conditions (**Table 2**, entry 24): β-keto ester **1a** (81.5 μL, 79.0 mg, 0.500 mmol); allylic alcohol **2l** (0.600 M in 1,4-dioxane, 1.00 mL, 0.600 mmol, 1.20 mol amt); [RuCp(CH_3CN)$_3$]PF_6 (2.16 mg, 4.99 μmol, 1.00 mol%); $\mathbf{L^2}_{\mathit{S}}$ (2.73 mg, 5.01 μmol, 1.00 mol%); Pd$\mathbf{L^1}_{\mathit{R}}$(OTf) (5.31 mg, 5.00 μmol, 1.00 mol%); 10 °C; 24 h. After 24 h: >99% conversion, branch/linear = >99.9:<0.1, *syn*:*anti* = 99.6:0.4. **Supplementary Figure 74** showed a ^{1}H-NMR spectrum of the reaction mixture. Work up: filtration through short pad of silica. Purification: neutralized SiO_2-chromatography (20 g; toluene, within 5 min). Results: **3al**, 150 mg, 98.5% yield. ^{1}H NMR ($CDCl_3$) δ 1.17 (s, 9H, C(CH_3)$_3$), 2.28 (s, 3H, CH_3CO), 3.77 (s, 3H, ArOCH_3), 3.85 (d, *J* = 11.4 Hz, 1H, COCH), 4.03 (dd, *J* = 7.80, 11.4 Hz, 1H, ArC*H*CH=CH_2), 4.99–5.02 (m, 2H, ArCHCH=CH_2), 5.86 (m, 1H, ArCHC*H*=CH_2), 6.83–6.85 (m, 3H, ArH), 7.13–7.15 (m, 2H, ArH); ^{13}C NMR ($CDCl_3$) δ 27.66, 29.45, 48.53, 55.42, 66.65, 82.04, 114.00, 115.94, 129.39, 132.45, 138.84, 158.65, 166.89, 202.29; HRMS (ESI) calcd for $C_{18}H_{24}NaO_4$ [M+Na$^+$] 327.1572, found 327.1570; $[\alpha]_D^{21}$ +26.578 (*c* 0.684, $CHCl_3$).

Enantiomer ratio: 99.9:0.1 (4.60 mmϕ x 250 mm DAICEL CHIRALPAK ID-3 column; 1.0:99.0 2-PrOH–Hex eluent; 0.50 mL/min flow rate; 220-nm detection; 25 °C; t_R, 28.4 min (major), 30.1 min (minor)). **Supplementary Figures 75** and **76** showed the ^{1}H- and ^{13}C-NMR spectra and HPLC charts, respectively.

<u>*tert*-Butyl (2*R*,3*S*)-2-acetyl-3-(naphthalen-2-yl)pent-4-enoate (*syn*-**3am**)</u>

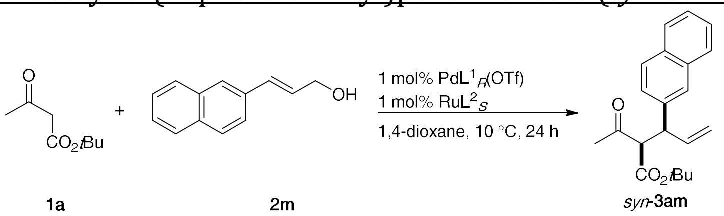

Reaction conditions (**Table 2**, entry 25): β-keto ester **1a** (81.5 μL, 79.0 mg, 0.500 mmol); allylic alcohol **2m** (0.600 M in 1,4-dioxane, 1.00 mL, 0.600 mmol, 1.20 mol amt); [RuCp(CH_3CN)$_3$]PF_6 (2.16 mg, 4.99 μmol, 1.00 mol%); $\mathbf{L^2}_{\mathit{S}}$ (2.73 mg, 5.01 μmol, 1.00 mol%); Pd$\mathbf{L^1}_{\mathit{R}}$(OTf) (5.31 mg, 5.00 μmol, 1.00 mol%); 10 °C; 24 h. After 24 h: >99% conversion, branch/linear = >99.9:<0.1, *syn*:*anti* = 99.5:0.5. **Supplementary Figure 77** showed a ^{1}H-NMR spectrum of the reaction mixture. Work up: filtration through short pad of silica. Purification: neutralized SiO_2-chromatography (20 g; toluene, within 5 min). Results: **3am**, 153 mg, 94.6% yield. ^{1}H NMR ($CDCl_3$) δ 1.08 (s, 9H, C(CH_3)$_3$), 2.33 (s, 3H, CH_3CO), 4.03 (d, *J* = 12.0 Hz, 1H, COCH), 4.25 (dd, *J* = 7.80, 12.0 Hz, 1H, ArC*H*CH=CH_2), 5.01–5.10 (m, 2H, ArCHCH=CH_2), 5.86 (m, 1H, ArCHC*H*=CH_2), 7.35–7.37 (m, 1H, ArH), 7.43–7.46 (m, 2H, ArH), 7.46 (s, 1H, ArH), 7.77–8.00 (m, 3H, ArH); ^{13}C NMR ($CDCl_3$) δ 27.60, 29.57, 49.37, 66.39, 82.15, 116.64, 125.83, 126.22, 126.62, 127.06, 127.73, 127.82, 128.29, 132.67, 133.61, 137.95, 138.49, 166.87, 202.00; HRMS (ESI) calcd for $C_{21}H_{24}NaO_3$ [M+Na$^+$] 347.1623, found 347.1625; $[\alpha]_D^{23}$ +7.686 (*c* 0.510, $CHCl_3$).

Enantiomer ratio: 99.9:0.1 (4.60 mmϕ x 250 mm DAICEL CHIRALPAK ID-3 column; 1.0:99.0 2-PrOH–Hex eluent; 0.50 mL/min flow rate; 220-nm detection; 25 °C; t_R, 23.6 min (2*R*,3*S*; major), 25.1 min (2*S*,3*R*; minor)). **Supplementary Figures 78** and **79** showed the ^{1}H- and ^{13}C-NMR spectra and HPLC charts, respectively.

The abs confign was determined by the X-ray crystallographic analysis of (2*R*,3*S*)-2-((*R*)-1-hydroxyethyl)-3-(naphthalen-2-yl)pent-4-enoic acid ((1*R*,2*R*,3*S*)-$syn_{1,2}syn_{2,3}$-**11am**), which was prepared by reduction of (2*R*,3*S*)-*syn*-**3am** using K-selectride, followed by hydrolysis under acidic condition.

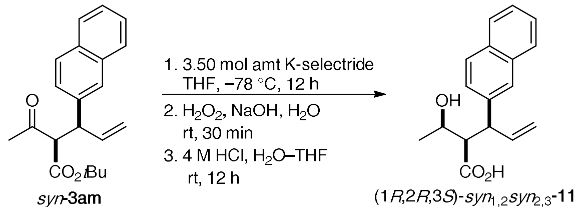

syn-3am (1*R*,2*R*,3*S*)-*syn*1,2*syn*2,3-11am

(2*R*,3*S*)-*syn*-**3am** (81.1 mg, 0.250 mmol, 99.2:0.8 dr, 99.9:0.1 er) was dissolved in dry THF (2.50 mL) and cooled to –78 °C. To this mixture, 1.0 M solution of K-selectride in THF (875 µL, 0.875 mmol, 3.50 mol amt) was added dropwise and the reaction mixture was stirred at this temperature for 12 h. After 12 h, 30% H_2O_2 aq. (1.00 mL) and 3.0 M NaOH aq. (1.00 mL) were added and the reaction mixture was slowly warmed to rt. The resulting solution was extracted with ether (3 x 5 mL). The organic layers were combined, washed with brine (20 mL), filtered through a pad of Celite, dried over Na_2SO_4, filtered, and concentrated. The residue was dissolved to THF (1.00 mL) and to this was added 4 M HCl aq. (4.00 mL) at rt. After 12 h at rt, the mixture was extracted with $CHCl_3$ (10.0 mL x 3). The organic extracts were dried over Na_2SO_4, filtered, and concentrated. The residue was purified by SiO_2-chromatography (ca. 40 g; 1:9 MeOH–$CHCl_3$ eluent) to give (1*R*,2*R*,3*S*)-$syn_{1,2}syn_{2,3}$-**11am** (65.1 mg, 96.3% yield) as an off-white solid. ^{1}H NMR ($CDCl_3$) δ 1.16 (d, *J* = 6.00 Hz, 3H, C*H*$_3$CH(OH)), 2.84 (dd, *J* = 10.2, 10.2 Hz, 1H, C*H*COOH), 3.99 (dd, *J* =10.2, 10.8 Hz, 1H, ArC*H*CH=CH$_2$), 4.07–4.13 (m, 1H, C*H*OH), 5.12 (d, *J* = 10.2 Hz, 1H, ArCHCH=CH*H*), 5.23 (d, *J* = 16.2 Hz, 1H, ArCHCH=CH*H*), 5.90 (ddd, *J* = 10.2, 10.8, 16.2 Hz, 1H, ArCHC*H*=CH$_2$) , 7.31 (d, *J* = 9.00 Hz, 1H, ArH), 7.41–7.45 (m, 2H, ArH), 7.63 (s, 1H), 7.72 (d, *J* = 9.00 Hz, 1H, ArH), 7.76 (t, *J* = 9.00 Hz, 1H, ArH); ^{13}C NMR ($CDCl_3$) δ 22.32, 49.84, 56.20, 65.78, 117.72, 125.86, 126.17, 126.23, 126.62, 127.76, 127.91, 128.47, 132.64, 133.4, 138.25, 138.68, 177.18; HRMS (ESI) calcd for $C_{17}H_{17}O_3$ [M–H$^-$] 269.1178, found 269.1178; $[\alpha]_D^{21}$ –70.876 (*c* 0.501, $CHCl_3$). **Supplementary Figure 80** showed the ^{1}H- and ^{13}C-NMR spectra of (1*R*,2*R*,3*S*)-$syn_{1,2}syn_{2,3}$-**11am**.

Recrystallization: (1*R*,2*R*,3*S*)-$syn_{1,2}syn_{2,3}$-**11am** (10.0 mg); overlay of Hex (2 mL) on $CHCl_3$ solution of (1*R*,2*R*,3*S*)-$syn_{1,2}syn_{2,3}$-**11am** (0.5 mL); rt/4 h; crystallization yield, ca. 50%; mp 142 °C; Flack, 0.03(5) for (1*R*,2*R*,3*S*)-$syn_{1,2}syn_{2,3}$-**11am**. **Supplementary Table 7** summarized the crystallographic data, and the molecular structure in a crystal was shown in **Supplementary Figure 81**. Crystallographic data have been deposited with Cambridge Crystallographic Data Centre as supplementary publication no. CCDC-2193063.

tert-Butyl 3-((3*S**,4*R**)-4-(*tert*-butoxycarbonyl)-5-oxohex-1-en-3-yl)-1*H*-pyrrole-1-carboxylate (*syn*-**3an**)

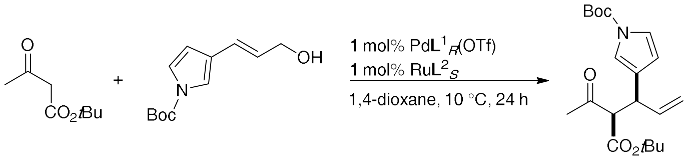

1a 2n *syn*-3an

Reaction conditions (**Table 2**, entry 26): β-keto ester **1a** (81.5 µL, 79.0 mg, 0.500 mmol); allylic alcohol **2n** (0.500 M in 1,4-dioxane, 2.00 mL, 1.00 mmol, 2.00 mol amt); [RuCp(CH_3CN)$_3$]PF_6 (2.16 mg, 4.99 µmol, 1.00 mol%); $\mathbf{L^2}_S$ (2.73 mg, 5.01 µmol, 1.00 mol%); Pd$\mathbf{L^1}_R$(OTf) (5.31 mg, 5.00 µmol, 1.00 mol%); 10 °C; 24 h. After 24 h: >99% conversion, branch/linear = >99.9:<0.1, *syn*:*anti* = 99.6:0.4. **Supplementary Figure 82** showed a ^{1}H-NMR spectrum of the reaction mixture. Work up: filtration through short pad of silica. Purification: neutralized SiO_2-chromatography (20 g; toluene, within 5

min). Results: **3an**, 176 mg, 96.7% yield. ^{1}H NMR ($CDCl_3$) δ 1.27 (s, 9H, $C(CH_3)_3$), 1.61 (s, 9H, $NOCOC(CH_3)_3$), 2.27 (s, 3H, CH_3CO), 3.92 (d, *J* = 10.8 Hz, 1H, COCH), 4.95 (d, *J* = 17.4 Hz, 1H, ArCHCH=C*H*H), 5.01 (d, *J* = 10.2 Hz, 1H, ArCHCH=CH*H*), 5.01 (br, 1H, ArC*H*CH=CH_2), 5.84–5.89 (m, 1H, ArCHC*H*=CH_2), 6.06–6.08 (m, 2H, ArH), 7.19–7.20 (m, 1H, ArH); ^{13}C NMR ($CDCl_3$) δ 27.79, 28.16, 29.78, 40.67, 66.17, 81.95, 83.91, 109.99, 111.77, 116.55, 121.70, 134.11, 137.87, 149.30, 166.89, 202.02; HRMS (ESI) calcd for $C_{20}H_{29}NNaO_5$ [M+Na$^+$] 386.1943, found 386.1946; $[\alpha]_D^{21}$ −1.443 (*c* 0.456, $CHCl_3$).

Enantiomer ratio: 99.7:0.3 (4.60 mmϕ x 250 mm DAICEL CHIRALPAK IE-3 column; 1.0:99.0 2-PrOH–Hex eluent; 1.00 mL/min flow rate; 220-nm detection; 25 °C; t_R, 10.2 min (major), 13.8 min (minor)). **Supplementary Figures 83** and **84** showed the ^{1}H- and ^{13}C-NMR spectra and HPLC charts, respectively.

tert-Butyl (α*R*,β*S*)-α-acetyl-β-vinyl-*N*-(*tert*-butoxycarbonyl)-1*H*-indole-3-propionate (*syn*-**3ao**)

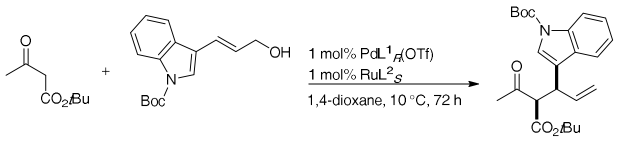

1a 2o (*R*,*S*)-*syn*-3ao

Reaction conditions (**Table 2**, entry 27): β-keto ester **1a** (81.5 μL, 79.0 mg, 0.500 mmol); allylic alcohol **2o** (0.600 M in 1,4-dioxane, 1.00 mL, 0.600 mmol, 1.20 mol amt); $[RuCp(CH_3CN)_3]PF_6$ (2.16 mg, 4.99 μmol, 1.00 mol%); $\mathbf{L^2}_S$ (2.73 mg, 5.01 μmol, 1.00 mol%); Pd$\mathbf{L^1}_R$(OTf) (5.31 mg, 5.00 μmol, 1.00 mol%); 10 °C; 72 h. After 72 h: >99% conversion, branch/linear = >99.9:<0.1, *syn*:*anti* = 97.8:2.2. **Supplementary Figure 85** showed a ^{1}H-NMR spectrum of the reaction mixture. Work up: filtration through short pad of silica. Purification: neutralized SiO_2-chromatography (20 g; toluene, within 5 min). Results: **3ao**, 192 mg, 92.8% yield. ^{1}H NMR ($CDCl_3$) δ 1.17 (s, 9H, $C(CH_3)_3$), 1.66 (s, 9H, $NOCOC(CH_3)_3$), 2.31 (s, 3H, CH_3CO), 4.03 (d, *J* = 12.0 Hz, 1H, COCH), 4.37 (dd, *J* = 7.80, 12.0 Hz, 1H, ArC*H*CH=CH_2), 5.08 (d, *J* = 9.60 Hz, 1H, ArCHCH=C*H*H), 5.15 (d, *J* = 16.8 Hz, 1H, ArCHCH=CH*H*), 5.90 (ddd, *J* = 7.80, 9.60, 16.8 Hz, 1H, ArCHC*H*=CH_2), 7.23 (dd, *J* = 7.20, 7.80 Hz, 1H, ArH), 7.43 (s, 1H, ArH), 7.59 (d, *J* = 7.20 Hz, 1H, ArH), 8.13 (br, 1H, ArH); ^{13}C NMR ($CDCl_3$) δ 27.61, 28.35, 29.80, 40.40, 65.35, 82.26, 83.75, 115.37, 117.21, 119.77, 119.94, 122.59, 123.00, 124.67, 129.66, 135.70, 137.10, 149.72, 166.92, 201.98; HRMS (ESI) calcd for $C_{24}H_{31}NNaO_5$ [M+Na$^+$] 436.2100, found 436.2101; $[\alpha]_D^{23}$ −3.796 (*c* 0.401, $CHCl_3$).

Enantiomer ratio: 99.9:0.1 (4.60 mmϕ x 250 mm DAICEL CHIRALPAK IE-3 column; 1.0:99.0 2-PrOH–Hex eluent; 1.00 mL/min flow rate; 220-nm detection; 25 °C; t_R, 17.7 min (α*R*,β*S*; major), 29.1 min (α*S*,β*R*; minor)). **Supplementary Figures 86** and **87** showed the ^{1}H- and ^{13}C-NMR spectra and HPLC charts, respectively.

The abs confign was determined by the X-ray crystallographic analysis of the product (α*R*,β*S*)-*syn*-**3ao**. White solid *syn*-**3ao** (20.0 mg, >99:1 dr, 99.9:0.1 er) was dissolved in 5:1 mixture of Hex/EtOAc (ca. 2 mL) in a 5-mL glass tube with a screw cap and the solution was kept at −20 °C for 2 h, giving colorless block crystals (ca. 40% yield, mp 104.5 °C). The crystal was subjected to X-ray crystallographic analysis and the abs confign of *syn*-**3ao** derived from the Pd$\mathbf{L^1}_R$(OTf) and Ru$\mathbf{L}_S$-catalyzed allylation was determined to be α*R*,β*S* (Flack parameter, 0.15(8) for (α*R*,β*S*)-*syn*-**3ao**). **Supplementary Table 8** summarized the crystallographic data, and the molecular structure in a crystal was shown in **Supplementary Figure 88**. Crystallographic data have been deposited with Cambridge Crystallographic Data Centre as supplementary publication no. CCDC-2193064.

tert-Butyl 2-acetylpent-4-enoate (*linear*-**3ap**)

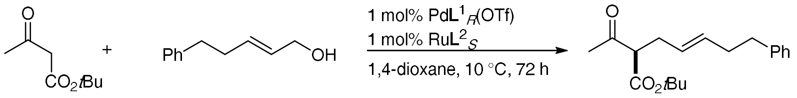

1a 2p *linear*-3ap

Reaction conditions (**Table 2**, entry 28): β-keto ester **1a** (81.5 μL, 79.0 mg, 0.500 mmol); allylic alcohol **2p** (0.600 M in 1,4-dioxane, 1.00 mL, 0.600 mmol, 1.20 mol amt); $[RuCp(CH_3CN)_3]PF_6$ (2.16 mg, 4.99 μmol, 1.00 mol%); $\mathbf{L^2}_S$ (2.73 mg, 5.01 μmol, 1.00 mol%); Pd $\mathbf{L^1}_R$(OTf) (5.31 mg, 5.00 μmol, 1.00 mol%); 10 °C; 72 h. After 72 h: 57.9% conversion, branch/linear = <0.1:>99.9. Work up: filtration through short pad of silica. **Supplementary Figure 89** showed the crude ^{1}H-NMR spectrum of the reaction mixture. Purification: neutralized SiO_2-chromatography (20 g; toluene, within 5 min). Results: **3ac**, 72.4 mg, 47.9% yield; ^{1}H NMR ($CDCl_3$) δ 1.45 (s, 9H, $C(CH_3)_3$), 2.17 (s, 3H, CH_3CO), 2.37–2.40 (m, 2H, $PhCH_2CH_2$), 2.45–2.48 (m, 2H, $PhCH_2CH_2$), 2.65–2.68 (m, 2H, $CH_3COCHCH_2$), 3.17 (d, *J* = 7.80 Hz, 1H, $CH_3COCHCH_2$), 5.27–5.30 (m, 1H, C*H*=CH), 5.47–5.51 (m, 1H, CH=C*H*), 7.18–7.29 (m, 5H, ArH); ^{13}C NMR ($CDCl_3$) δ 25.97, 28.00, 29.18, 29.27, 35.77, 60.56, 82.01, 125.91, 125.93, 128.39, 128.62, 131.53, 141.88, 168.64, 203.24; HRMS (ESI) calcd for $C_{19}H_{26}NaO_3$ [M+Na$^+$] 325.1780, found 325.1779; $[\alpha]_D^{20}$ +11.152 (*c* 1.00, $CDCl_3$).

Enantiomer ratio: 64.4:35.6 (4.60 mmϕ x 250 mm DAICEL CHIRALPAK IE-3 column; 0.5:99.5 2-PrOH–Hex eluent; 0.25 mL/min flow rate; 220-nm detection; 25 °C; t_R, 53.0 min (major), 57.1 min (minor)). **Supplementary Figures 90** and **91** showed the ^{1}H- and ^{13}C-NMR spectra and HPLC charts, respectively.

5.2. Mismatched system.

According to the general procedure described for dehydrative allylation of β-keto ester **1a** and allylic alcohol **2a**, all reactions were carried out basically under the conditions of 0.500-mmol scale of substrates, $[RuCp(CH_3CN)_3]PF_6$ (10.0 μmol), $\mathbf{L^2}_R$ (10.0 μmol), Pd$\mathbf{L^1}_R$(OTf) (5.00 μmol) and 1,4-dioxane (4.00 mL). Listed below are the reaction conditions (β-keto ester (mg, mmol), allylic alcohol (mg, mmol, mol amt), reaction time and reaction temperature), isolated yield, physical properties of the products. Relative configuration and abs confign of **3aa** was determined by X-ray crystallographic analysis as described below. Relative configurations of other products were assigned by comparison a chemical shift of *tert*-butyl ester moiety in ^{1}H-NMR analysis (*syn*: 1.05–1.30, *anti*: 1.35–1.65) with those of structure-confirmed products. Abs configns of them were not determined, and the structures in schemes and figures were drawn as estimated ones from the other structure-confirmed product.

tert-Butyl (2*R*,3*R*)-2-acetyl-3-phenylpent-4-enoate (*anti*-**3aa**)

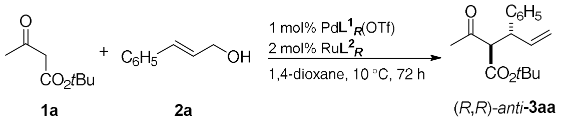

1a 2a (*R*,*R*)-*anti*-3aa

Reaction conditions (**Table 3**, entry 1): β-keto ester **1a** (81.5 μL, 79.0 mg, 0.500 mmol); allylic alcohol **2a** (0.150 M in 1,4-dioxane, 4.00 mL, 0.600 mmol, 1.20 mol amt); $[RuCp(CH_3CN)_3]PF_6$ (4.33 mg, 9.99 μmol, 2.00 mol%); $\mathbf{L^2}_R$ (5.47 mg, 10.0 μmol, 2.01 mol%); Pd$\mathbf{L^1}_R$(OTf) (5.32 mg, 5.00 μmol, 1.00 mol%); 10 °C; 72 h. After 72 h: >99% conversion, branch/linear = >99.9:<0.1, *syn*:*anti* = 3.9:96.1. **Supplementary Figure 92** showed a ^{1}H-NMR spectrum of the reaction mixture. Work up: filtration through short pad of silica. Purification: SiO_2-chromatography (20 g; toluene, within 5 min). Results: *anti*-**3aa**, 135 mg, 98.7% yield. ^{1}H NMR ($CDCl_3$) δ 1.46 (s, 9H, $C(CH_3)_3$), 2.00 (s, 3H, CH_3CO), 3.92 (d, *J* = 10.8 Hz, 1H, COCH), 4.07 (dd, *J* = 8.40, 10.8 Hz, 1H, ArC*H*CH=CH_2),

5.07 (d, J = 10.8 Hz, 1H, ArCHCH=CHH), 5.10 (d, J = 16.2 Hz, 1H, ArCHCH=CHH), 5.96 (ddd, J = 8.40, 10.8, 16.2 Hz, 1H, ArCHCH=CH_2), 7.19–7.30 (m, 5H, ArH); ^{13}C NMR ($CDCl_3$) δ 28.09, 29.81, 49.47, 66.02, 82.37, 116.22, 127.14, 128.21, 128.89, 138.53, 140.49, 167.23, 201.92; HRMS (ESI) cacld for $C_{17}H_{22}NaO_3$ [M+Na$^+$] 297.1467, found 297.1463; $[\alpha]_D^{20}$ +70.732 (c 0.710, $CHCl_3$).

Enantiomer ratio: 99.9:0.1 (4.60 mmϕ x 250 mm DAICEL CHIRALPAK ID-3 column; 1.0:99.0 2-PrOH–Hex eluent; 0.50 mL/min flow rate; 220-nm detection; 25 °C; t_R, 30.8 min (2*S*,3*S*; minor), 33.1 min (2*R*,3*R*; major)). **Supplementary Figure 93** showed the HPLC charts of the *anti*:*syn* = 96.0:4.0 mixtures obtained by SiO_2-chromatography.

Recrystallization: 100 mg of *anti*-**3aa** (*syn*:*anti* = 3.9:96.1) was dissolved to ca. 1 mL of pentane and cooled to –20 °C for 4 h to form white crystals (86.7 mg, *anti*/*syn* = >99:1, 87% yield). **Supplementary Figure 94** showed the ^{1}H- and ^{13}C-NMR spectra of pure *anti*-**3aa**.

The abs confign was determined by the X-ray crystallographic analysis of the product (2*R*,3*R*)-*anti*-**3aa** itself. White solid *anti*-**3aa** (30.0 mg, >99:1 dr, 99.9:0.1 er) was dissolved in Hex (2 mL) in a 5-mL glass tube with a screw cap and the solution was kept at 25 °C for 2 weeks, giving colorless block crystals (ca. 60% yield, mp 48.0 °C). The crystal was subjected to X-ray crystallographic analysis and the abs confign of *anti*-**3aa** derived from the Pd$\mathbf{L^1}_{\boldsymbol{R}}$(OTf) and Ru$\mathbf{L^2}_{\boldsymbol{R}}$-catalyzed allylation was determined to be 2*R*,3*R* (Flack parameter, 0.07(9) for (2*R*,3*R*)-*anti*-**3aa**). **Supplementary Table 9** summarized the crystallographic data, and the molecular structure in a crystal was shown in **Supplementary Figure 95**. Crystallographic data have been deposited with Cambridge Crystallographic Data Centre as supplementary publication no. CCDC-2193039.

<u>*tert*-Butyl (2*R**,3*R**)-3-phenyl-2-propionylpent-4-enoate (*anti*-**3ca**)</u>

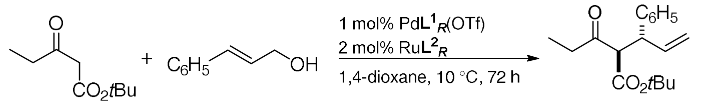

1c **2a** *anti*-**3ca**

Reaction conditions (**Table 3**, entry 2): β-keto ester **1c** (86.1 mg, 0.500 mmol); allylic alcohol **2a** (0.150 M in 1,4-dioxane, 4.00 mL, 0.600 mmol, 1.20 mol amt); $[RuCp(CH_3CN)_3]PF_6$ (4.33 mg, 9.99 μmol, 2.00 mol%); $\mathbf{L^2}_{\boldsymbol{R}}$ (5.47 mg, 10.0 μmol, 2.01 mol%); Pd$\mathbf{L^1}_{\boldsymbol{R}}$(OTf) (5.30 mg, 5.00 μmol, 1.00 mol%); 10 °C; 72 h. After 72 h: >99% conversion, branch/linear = >99.9:<0.1, *syn*:*anti* = 8.0:92.0. **Supplementary Figure 96** showed a ^{1}H-NMR spectrum of the reaction mixture. Work up: filtration through short pad of silica. Purification: SiO_2-chromatography (20 g; toluene, within 5 min). Results: *anti*-**3ca**, 136 mg, 94.2% yield. ^{1}H NMR ($CDCl_3$) δ 0.81 (t, J = 7.2 Hz, 3H, C$H_3$$CH_2$), 1.46 (s, 9H, C(C$H_3$)$_3$), 2.09–2.16 (m, 1H, CH_3CHH), 2.35–2.42 (m, 1H, CH_3CHH), 3.92 (d, J = 10.8 Hz, 1H, C$H$$CO_2C(CH_3)_3$), 4.09 (dd, J = 8.40, 10.8 Hz, 1H, ArCHCH=CH_2), 5.08 (d, J = 10.2 Hz, 1H, ArCHCH=CHH), 5.10 (d, J = 17.4 Hz, 1H, ArCHCH=CHH), 5.98 (ddd, J = 8.40, 10.2, 17.4 Hz, 1H, ArCHCH=CH_2), 7.18–7.20 (m, 3H, ArH), 7.26–7.28 (m, 2H, ArH); ^{13}C NMR ($CDCl_3$) δ 7.54, 28.08, 36.51, 49.38, 65.07, 82.22, 116.14, 127.05, 128.24, 128.81, 138.54, 140.58, 167.23, 204.59; HRMS (ESI) calcd for $C_{18}H_{24}NaO_3$ [M+Na$^+$] 311.1623, found 311.1616; $[\alpha]_D^{21}$ +72.751 (c 0.468, $CHCl_3$).

Enantiomer ratio: 99.8:0.2 (4.60 mmϕ x 250 mm DAICEL CHIRALPAK ID-3 column; 0.5:99.5 2-PrOH–Hex eluent; 0.50 mL/min flow rate; 220-nm detection; 25 °C; t_R, 24.0 min (2*S*,3*R*; minor), 25.5 min (2*R*,3*S*; major)). **Supplementary Figure 97**

showed the HPLC charts of the *syn*:*anti* = 8.0:92.0 mixtures obtained by SiO_2-chromatography.

Recrystallization: 100 mg of *anti*-**3ca** (*anti*/*syn* = 92.0:8.0) was dissolved to ca. 1 mL of pentane and cooled to −20 °C for 12 h to form white crystals (72.3 mg, *anti*/*syn* = >99:1, 72% yield). **Supplementary Figure 98** showed the ^{1}H- and ^{13}C-NMR spectra NMR charts of pure *anti*-**3ca**.

tert-Butyl 2-isobutyryl-3-phenylpent-4-enoate (*anti*-**3da**)

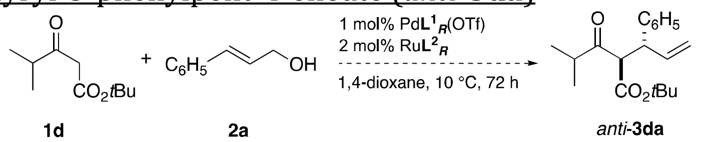

Reaction conditions (**Table 3**, entry 3): β-keto ester **1d** (93.3 mg, 0.500 mmol); allylic alcohol **2a** (0.150 M in 1,4-dioxane, 4.00 mL, 0.600 mmol, 1.20 mol amt); $[RuCp(CH_3CN)_3]PF_6$ (4.33 mg, 10.0 μmol, 2.00 mol%); $\mathbf{L^2}_{\boldsymbol{R}}$ (5.45 mg, 10.0 μmol, 2.00 mol%); Pd$\mathbf{L^1}_{\boldsymbol{R}}$(OTf) (5.30 mg, 5.00 μmol, 1.00 mol%); 10 °C; 72 h. After 72 h: <5% conversion.

tert-Butyl (2*R**,3*R**)-2-benzoyl-3-phenylpent-4-enoate (*anti*-**3ga**)

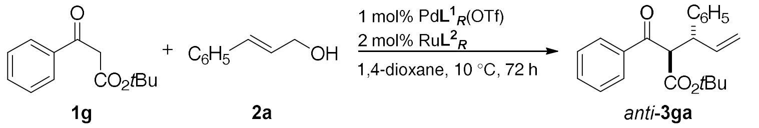

Reaction conditions (**Table 3**, entry 4): β-keto ester **1g** (110 mg, 0.500 mmol); allylic alcohol **2a** (0.150 M in 1,4-dioxane, 4.00 mL, 0.600 mmol, 1.20 mol amt); $[RuCp(CH_3CN)_3]PF_6$ (4.35 mg, 10.1 μmol, 2.00 mol%); $\mathbf{L^2}_{\boldsymbol{R}}$ (5.45 mg, 10.0 μmol, 2.00 mol%); Pd$\mathbf{L^1}_{\boldsymbol{R}}$(OTf) (5.31 mg, 5.00 μmol, 1.00 mol%); 10 °C; 72 h. After 72 h: 93.4% conversion, branch/linear = >99.9:<0.1, *syn*:*anti* = 2.2:97.8. **Supplementary Figure 99** showed a ^{1}H-NMR spectrum of the reaction mixture. Work up: filtration through short pad of silica. Purification: SiO_2-chromatography (20 g; toluene, within 5 min). Results: *anti*-**3ga**, 155 mg, 92.7% yield. ^{1}H NMR ($CDCl_3$) δ 1.36 (s, 9H, $C(CH_3)_3$), 4.34 (dd, *J* = 9.00, 10.8 Hz, 1H, PhC*H*CH=CH_2), 4.83 (d, *J* = 10.8 Hz, 1H, COCH), 5.10 (d, *J* = 9.60 Hz, 1H, PhCHCH=C*H*H), 5.17 (d, *J* = 16.8 Hz, 1H, PhCHCH=CH*H*), 6.06 (ddd, *J* = 9.00, 9.60, 16.8 Hz, 1H, PhCHC*H*=CH_2), 7.08–7.11 (m, 1H, ArH), 7.18–7.23 (m, 4H, ArH), 7.38–7.41 (m, 2H, ArH), 7.50–7.52 (m, 1H, ArH); ^{13}C NMR ($CDCl_3$) δ 27.97,49.48, 60.62, 82.40, 116.46, 126.75, 128.13, 128.60, 128.62, 128.65, 133.35, 137.00, 138.66, 141.21, 167.28, 193.24; HRMS (ESI) calcd for $C_{22}H_{24}NaO_3$ [M+Na$^+$] 359.1623, found 359.1620; $[\alpha]_D^{21}$ +76.190 (*c* 0.457, $CHCl_3$).

Enantiomer ratio: 99.8:0.2 (4.60 mmϕ x 250 mm DAICEL CHIRALPAK IB and CHIRALPAK IB-3 column; 0.2:99.8 2-PrOH–Hex eluent; 1.00 mL/min flow rate; 220-nm detection; 25 °C; t_R, 27.1 min (major), 30.7 min (minor)). **Supplementary Figures 100** and **101** showed the ^{1}H NMR, ^{13}C NMR spectra and HPLC charts of the *syn*:*anti* = 2.2:97.8 mixtures obtained by SiO_2-chromatography, respectively.

tert-Butyl (2*R**,3*R**)-2-acetyl-2-fluoro-3-phenylpent-4-enoate (*anti*-**3la**)

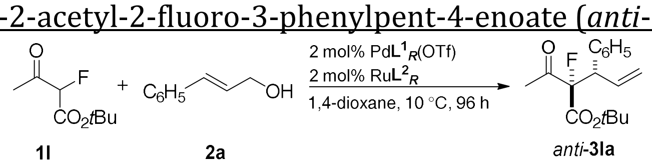

Reaction conditions (**Table 3**, entry 5): β-keto ester **1l** (88.1 mg, 0.50 mmol); allylic alcohol **2a** (0.150 M in 1,4-dioxane, 4.00 mL, 0.600 mmol, 1.20 mol amt); $[RuCp(CH_3CN)_3]PF_6$ (4.33 mg, 10.0 μmol, 2.00 mol%); $\mathbf{L^2}_{\boldsymbol{R}}$ (5.45 mg, 10.0 μmol, 2.00 mol%); Pd$\mathbf{L^1}_{\boldsymbol{R}}$(OTf) (10.6 mg, 5.00 μmol, 2.00 mol%); 10 °C; 96 h. After 96 h: 96.8%

conversion, branch/linear = >99.9:<0.1, *syn*:*anti* = 6.2:93.8. **Supplementary Figure 102** showed a ^{1}H-NMR spectrum of the reaction mixture. Work up: filtration through short pad of silica. Purification: SiO_2-chromatography (40 g; toluene). Results: **3la**, 135 mg, 92.4% yield. ^{1}H NMR ($CDCl_3$) δ 1.50 (s, 9H, $C(CH_3)_3$), 1.89 (d, *J* = 5.40 Hz, 3H, CH_3), 4.32 (dd, *J* = 9.00, 33.0 Hz, 1H, PhC*H*CH=CH_2), 5.22 (d, *J* = 1.80 Hz, 1H, PhCHCH=CH*H*), 5.24 (d, *J* = 9.60 Hz, 1H, PhCHCH=C*H*H), 6.10–6.16 (m, 1H, PhCHC*H*=CH_2), 7.22–7.35 (m, 5H, ArH); ^{13}C NMR ($CDCl_3$) δ 26.88, 27.98, 53.96 (d, *J* = 17.3 Hz), 84.31, 102.54 (d, *J* = 205.2 Hz, 2C), 119.11, 127.66, 128.70, 129.67, 134.17 (d, *J* = 4.3 Hz), 136.93, 164.04 (d, *J* = 25.8 Hz), 202.17 (d, *J* = 30.2 Hz); HRMS (ESI) calcd for $C_{17}H_{21}FNaO_3$ [M+Na$^+$] 315.1372, found 315.1368; $[\alpha]_D^{22}$ +58.139 (*c* 0.596, $CHCl_3$).

Enantiomer ratio: 99.9:0.1 (4.60 mmϕ x 250 mm DAICEL CHIRALPAK ID-3 column; 1.0:99.0 2-PrOH–Hex eluent; 0.50 mL/min flow rate; 254-nm detection; 25 °C; t_R, 36.5 min (major), 43.7 min (minor)). **Supplementary Figures 103** and **104** showed the ^{1}H- and ^{13}C-NMR spectra and HPLC charts, respectively.

tert-Butyl (2*R**,3*R**)-2-acetyl-3-(*o*-tolyl)pent-4-enoate (*anti*-**3ac**)

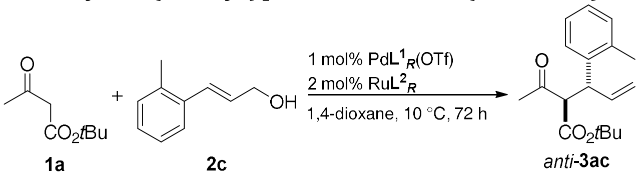

Reaction conditions (**Table 3**, entry 6): β-keto ester **1a** (81.5 μL, 79.0 mg, 0.500 mmol); allylic alcohol **2c** (0.150 M in 1,4-dioxane, 4.00 mL, 0.600 mmol, 1.20 mol amt); $[RuCp(CH_3CN)_3]PF_6$ (4.34 mg, 10.0 μmol, 2.00 mol%); $\mathbf{L^2}_{\boldsymbol{S}}$ (5.45 mg, 10.0 μmol, 2.00 mol%); Pd$\mathbf{L^1}_{\boldsymbol{R}}$(OTf) (5.31 mg, 5.00 μmol, 1.00 mol%); 10 °C; 72 h. After 72 h: 97.6% conversion, branch/linear = >99.9:<0.1, *syn*:*anti* =5.0:95.0. **Supplementary Figure 105** showed a ^{1}H-NMR spectrum of the reaction mixture. Work up: filtration through short pad of silica. Purification: neutralized SiO_2-chromatography (20 g; toluene, within 5 min). Results: **3ac**, 133 mg, 92.2% yield, colorless oil. ^{1}H NMR ($CDCl_3$) δ 1.47 (s, 9H, $C(CH_3)_3$), 2.01 (s, 3H, CH_3CO), 2.31 (s, 3H, $ArCH_3$), 3.91 (d, *J* = 10.8 Hz, 1H, COCH), 4.03 (dd, *J* = 8.40, 10.8 Hz, 1H, ArC*H*CH=CH_2), 5.06 (d, *J* = 9.60 Hz, 1H, ArCHCH=C*H*H), 5.10 (d, *J* = 16.2 Hz, 1H, ArCHCH=CH*H*), 5.94 (ddd, *J* = 8.40, 9.60, 16.2 Hz, 1H, ArCHC*H*=CH_2), 6.98–7.02 (m, 3H, ArH), 7.15–7.18 (m, 1H, ArH); ^{13}C NMR ($CDCl_3$) δ 21.58, 28.07, 29.86, 49.49, 65.93, 82.34, 116.09, 125.03, 127.92, 128.75, 128.94, 138.50, 138.59, 140.36, 167.27, 202.11; HRMS (ESI) calcd for $C_{18}H_{24}NaO_3$ [M+Na$^+$] 311.1623, found 311.1628; $[\alpha]_D^{22}$ +93.335 (*c* 0.809, $CHCl_3$).

Enantiomer ratio: 99.2:0.8 (4.60 mmϕ x 250 mm DAICEL CHIRALPAK ID-3 column; 1.0:99.0 2-PrOH–Hex eluent; 1.00 mL/min flow rate; 254-nm detection; 25 °C; t_R, 10.4 min (major), 13.3 min (minor)). **Supplementary Figures 106** and **107** showed the ^{1}H- and ^{13}C-NMR spectra and HPLC charts, respectively.

tert-Butyl (2*R**,3*R**)-2-acetyl-3-(*o*-tolyl)pent-4-enoate (*anti*-**3ae**)

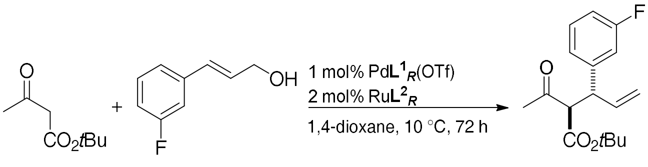

Reaction conditions (**Table 3**, entry 7): β-keto ester **1a** (81.5 μL, 79.0 mg, 0.500 mmol); allylic alcohol **2e** (0.150 M in 1,4-dioxane, 4.00 mL, 0.600 mmol, 1.20 mol amt); $[RuCp(CH_3CN)_3]PF_6$ (4.30 mg, 9.91 μmol, 1.98 mol%); $\mathbf{L^2}_{\boldsymbol{R}}$ (5.46 mg, 10.0 μmol, 2.00 mol%); Pd$\mathbf{L^1}_{\boldsymbol{R}}$(OTf) (5.32 mg, 5.00 μmol, 1.00 mol%); 10 °C; 72 h. After 72 h: >99.9%

conversion, branch/linear = >99.9:<0.1, *syn*:*anti* = 5.0:95.0. **Supplementary Figure 108** showed a ^{1}H-NMR spectrum of the reaction mixture. Work up: filtration through short pad of silica. Purification: SiO_2-chromatography (20 g; toluene, within 5 min). Results: *anti*-**3ae**, 138 mg, 94.6% yield. ^{1}H NMR ($CDCl_3$) δ 1.45 (s, 9H, $C(CH_3)_3$), 2.02 (s, 3H, CH_3CO), 3.89 (d, *J* = 11.4 Hz, 1H, COCH), 4.07 (dd, *J* = 8.40, 11.4 Hz, 1H, ArC*H*CH=CH_2), 5.09 (d, *J* = 5.40 Hz, 1H, ArCHCH=C*H*H), 5.11 (d, *J* = 12.0 Hz, 1H, ArCHCH=CH*H*), 5.93 (ddd, *J* = 5.40, 8.40, 12.0 Hz, 1H, ArCHC*H*=CH_2), 6.88–6.98 (m, 3H, ArH), 7.23–7.26 (m, 1H, ArH); ^{13}C NMR ($CDCl_3$) δ 28.04, 29.95, 48.93, 65.78, 82.61, 114.12 (d, *J* = 20.2 Hz), 115.07 (d, *J* = 21.5 Hz), 116.81, 123.86 (d, *J* = 2.9 Hz), 130.35 (d, *J* = 8.6 Hz), 137.8, 143.17 (d, *J* = 5.7 Hz), 163.04 (d, *J* = 245.4 Hz), 166.90, 201.50; HRMS (ESI) calcd for $C_{17}H_{21}FNaO_3$ [M+Na$^+$] 315.1372, found 315.1376; $[\alpha]_D^{22}$ +78.556 (*c* 0.568, $CHCl_3$).

Enantiomer ratio: 99.8:0.2 (4.60 mmϕ x 250 mm DAICEL CHIRALPAK IA and DAICEL CHIRALPAK IA-3 column; 0.5:99.5 2-PrOH–Hex eluent; 0.50 mL/min flow rate; 220-nm detection; 25 °C; t_R, 49.7 min (minor), 51.3 min (major)). **Supplementary Figures 109** and **110** showed the ^{1}H- and ^{13}C-NMR spectra and HPLC charts, respectively.

<u>*tert*-Butyl (2*R**,3*R**)-2-acetyl-3-(4-fluorophenyl)pent-4-enoate (*anti*-**3ah**)</u>

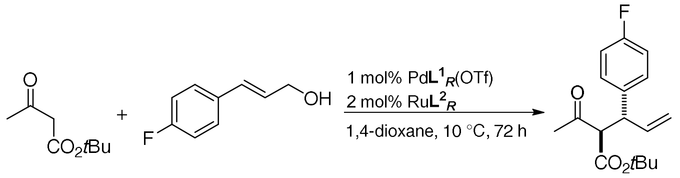

1a **2h** *anti*-**3ah**

Reaction conditions (**Table 3**, entry 8): β-keto ester **1a** (81.5 μL, 79.0 mg, 0.500 mmol); allylic alcohol **2h** (0.150 M in 1,4-dioxane, 4.00 mL, 0.600 mmol, 1.20 mol amt); $[RuCp(CH_3CN)_3]PF_6$ (4.34 mg, 10.0 μmol, 2.00 mol%); $\mathbf{L}^2{}_S$ (5.46 mg, 10.0 μmol, 2.00 mol%); Pd$\mathbf{L}^1{}_R$(OTf) (5.31 mg, 5.00 μmol, 1.00 mol%); 10 °C; 72 h. After 72 h: >99% conversion, branch/linear = >99.9:<0.1, *syn*:*anti* = 6.1:93.9. **Supplementary Figure 111** showed a ^{1}H-NMR spectrum of the reaction mixture. Work up: filtration through short pad of silica. Purification: neutralized SiO_2-chromatography (20 g; toluene, within 5 min). Results: *anti*-**3ah**, 133 mg, 90.9% yield. ^{1}H NMR ($CDCl_3$) δ 1.49 (s, 9H, $C(CH_3)_3$), 2.01 (s, 3H, CH_3CO), 3.87 (d, *J* = 11.4 Hz, 1H, COCH), 4.07 (dd, *J* = 8.40, 11.4 Hz, 1H, ArC*H*CH=CH_2), 5.07–5.10 (m, 2H, ArCHCH=C*H₂*), 5.93 (m, 1H, ArCHC*H*=CH_2), 6.96–6.99 (m, 2H, ArH), 7.15–7.18 (m, 2H, ArH); ^{13}C NMR ($CDCl_3$) δ 28.04, 29.93, 48.49, 66.05, 82.53, 115.72 (d, *J* = 21.6 Hz), 116.39, 129.72 (d, *J* = 8.7 Hz), 136.21, 138.24, 161.85 (d, *J* = 243.9 Hz), 167.01, 201.77; HRMS (ESI) calcd for $C_{17}H_{21}FNaO_3$ [M+Na$^+$] 315.1372, found 315.1372; $[\alpha]_D^{23}$ +71.774 (*c* 0.541, $CHCl_3$).

Enantiomer ratio: 99.9:0.1 (4.60 mmϕ x 250 mm DAICEL CHIRALPAK ID-3 column; 1.0:99.0 2-PrOH–Hex eluent; 0.50 mL/min flow rate; 220-nm detection; 25 °C; t_R, 21.7 min (major), 25.8 min (minor)). **Supplementary Figure 112** showed the HPLC charts of the *syn*:*anti* = 6.1:93.9 mixtures obtained by SiO_2-chromatography.

Recrystallization: 144 mg of *anti*-**3ah** (*anti*/*syn* = 93.9:6.1) was dissolved to ca. 2 mL of pentane and cooled to −20 °C for 12 h to form white crystals (114 mg, *anti*/*syn* = >99:1, 79.1% yield). **Supplementary Figure 113** showed the ^{1}H- and ^{13}C-NMR spectra NMR charts of pure *anti*-**3ah**.

tert-Butyl (2*R**,3*R**)-2-acetyl-3-(4-chlorophenyl)pent-4-enoate (*anti*-**3ai**)

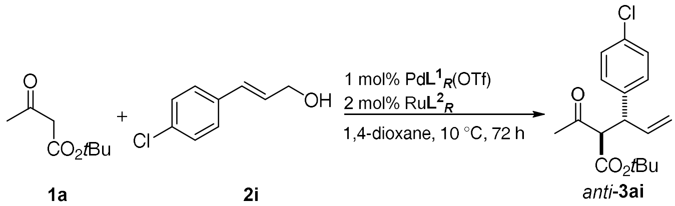

Reaction conditions (**Table 3**, entry 9): β-keto ester **1a** (81.5 μL, 79.0 mg, 0.500 mmol); allylic alcohol **2i** (0.150 M in 1,4-dioxane, 4.00 mL, 0.600 mmol, 1.20 mol amt); [RuCp(CH_3CN)$_3$]PF_6 (4.31 mg, 9.93 μmol, 1.99 mol%); $\mathbf{L}^2{}_{\boldsymbol{R}}$ (5.44 mg, 10.0 μmol, 2.00 mol%); Pd$\mathbf{L}^1{}_{\boldsymbol{R}}$(OTf) (5.31 mg, 5.00 μmol, 1.00 mol%); 10 °C; 72 h. After 72 h: 99% conversion, branch/linear = >99.9:<0.1, *syn*:*anti* = 4.4:95.6. **Supplementary Figure 114** showed a ^{1}H-NMR spectrum of the reaction mixture. Work up: filtration through short pad of silica. Purification: neutralized SiO_2-chromatography (20 g; toluene, within 5 min). Results: *anti*-**3ai**, 146 mg, 94.6% yield. ^{1}H NMR ($CDCl_3$) δ 1.46 (s, 9H, C(CH_3)$_3$), 2.02 (s, 3H, CH_3CO), 3.87 (d, *J* = 11.4 Hz, 1H, COCH), 4.06 (dd, *J* = 7.20, 11.4 Hz, 1H, ArC*H*CH=CH_2), 5.08–5.10 (m, 2H, ArCHCH=C*H*$_2$), 5.92 (m, ArCHC*H*=CH_2), 7.13–7.26 (m, 4H, ArH); ^{13}C NMR ($CDCl_3$) δ 28.08, 29.87, 48.60, 65.98, 82.60, 116.67, 129.04, 129.61, 132.95, 138.03, 139.17, 166.95, 201.45; HRMS (ESI) calcd for $C_{17}H_{21}ClNaO_3$ [M+Na$^+$] 331.1077, found 331.1076; $[\alpha]_D^{23}$ +96.867 (*c* 0.542, $CHCl_3$).

Enantiomer ratio: 99.8:0.2 (4.60 mmϕ x 250 mm DAICEL CHIRALPAK ID-3 column; 1.0:99.0 2-PrOH–Hex eluent; 0.50 mL/min flow rate; 220-nm detection; 25 °C; t_R, 21.9 min (major), 26.6 min (minor)). **Supplementary Figure 115** showed the HPLC charts of the *syn*:*anti* = 4.7:95.3 mixtures obtained by SiO_2-chromatography.

Recrystallization: 143 mg of *anti*-**3ai** (*anti*/*syn* = 95.3:4.7) was dissolved to ca. 1 mL of 20:1 mixture of pentane/ethyl acetate and cooled to −20 °C for 12 h to form white crystals (121 mg, *anti*/*syn* = >99:1, 84.6% yield). **Supplementary Figure 116** showed the ^{1}H- and ^{13}C-NMR spectra NMR charts of pure *anti*-**3ai**.

tert-Butyl (2*R**,3*R**)-2-acetyl-3-(*p*-tolyl)pent-4-enoate (*anti*-**3ak**)

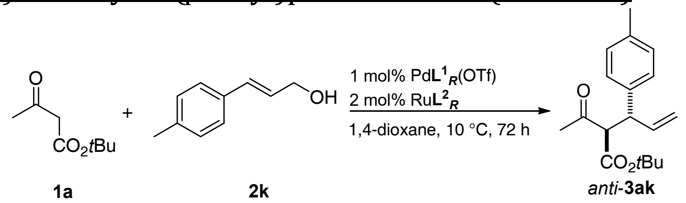

Reaction conditions (**Table 3**, entry 10): β-keto ester **1a** (81.5 μL, 79.0 mg, 0.500 mmol); allylic alcohol **2k** (0.150 M in 1,4-dioxane, 4.00 mL, 0.600 mmol, 1.20 mol amt); [RuCp(CH_3CN)$_3$]PF_6 (4.32 mg, 9.95 μmol, 1.95 mol%); $\mathbf{L}^2{}_{\boldsymbol{R}}$ (5.47 mg, 10.0 μmol, 2.00 mol%); Pd$\mathbf{L}^1{}_{\boldsymbol{R}}$(OTf) (5.31 mg, 5.00 μmol, 1.00 mol%); 10 °C; 72 h. After 72 h: >99% conversion, branch/linear = >99.9:<0.1, *syn*:*anti* = 4.9:95.1. **Supplementary Figure 117** showed a ^{1}H-NMR spectrum of the reaction mixture. Work up: filtration through short pad of silica. Purification: neutralized SiO_2-chromatography (20 g; toluene, within 5 min). Results: *anti*-**3ak**, 140 mg, 97.1% yield. ^{1}H NMR ($CDCl_3$) δ 1.48 (s, 9H, C(CH_3)$_3$), 2.00 (s, 3H, CH_3CO), 2.30 (s, 3H, ArCH_3), 3.90 (d, *J* = 11.4 Hz, 1H, COCH), 4.03 (dd, *J* = 7.80, 11.4 Hz, 1H, ArC*H*CH=CH_2), 5.04 (d, *J* = 10.2 Hz, 1H, ArCHCH=C*H*H), 5.09 (d, *J* = 15.6 Hz, 1H, ArCHCH=CH*H*), 5.93 (ddd, *J* = 7.80, 10.2, 15.6 Hz, 1H, ArCHC*H*=CH_2), 7.07–7.10 (m, 4H, ArH); ^{13}C NMR ($CDCl_3$) δ 21.17, 28.06, 29.81, 49.13, 66.02, 82.32, 115.95, 127.99, 129.60, 136.73, 138.68, 167.29, 202.19; HRMS (ESI) calcd for $C_{18}H_{24}NaO_3$ [M+Na$^+$] 311.1623, found 311.1626; $[\alpha]_D^{19}$ +67.992 (*c* 0.557, $CHCl_3$).

Enantiomer ratio: 99.6:0.4 for *anti*-**3ak** (4.60 mmϕ x 250 mm DAICEL CHIRALPAK IA-3 column; 1.0:99.0 2-PrOH–Hex eluent; 0.50 mL/min flow rate; 220-nm detection; 25 °C; t_R, 18.7 min (major), 25.8 min (minor). **Supplementary Figure 118** showed the HPLC charts of the *syn*:*anti* = 5.1:94.9 mixtures obtained by SiO_2-chromatography.

Recrystallization: 140 mg of *anti*-**3ak** (*anti*/*syn* = 95.1:4.9) was dissolved to ca. 2 mL of pentane and cooled to –20 °C for 12 h to form white crystals (116 mg, *anti*/*syn* = >99:1, 82.8% yield). **Supplementary Figure 119** showed the ^{1}H- and ^{13}C-NMR spectra of pure *anti*-**3ak**.

6. Diastereoselective reduction of 3aa.

6.1. *Syn*-selective reduction using K-selectride.

tert-Butyl (2*R*,3*S*)-2-((*R*)-1-hydroxyethyl)-3-phenylpent-4-enoate ((1*R*,2*R*,3*S*)-$syn_{1,2}syn_{2,3}$-**4aa**)

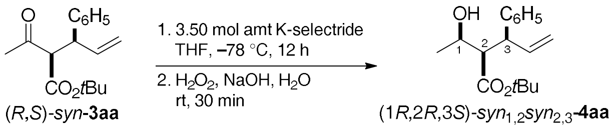

The detailed operation of diastereoselective reduction of **3aa** using K-selectride was described by taking the reduction of (*R*,*S*)-*syn*-**3aa** as the representative (**Figure 4**). A 20 mL Young-type Schlenk tube was charged with (*R*,*S*)-*syn*-**3aa** (137 mg, 0.500 mmol, 1.00 mol amt), and dry THF (5.00 mL). The solution was cooled to –78 °C. K-Selectride (1.0 M in THF, 1.75 mL, 1.75 mmol, 3.50 mol amt) was added dropwise at –78 °C and the reaction mixture was stirred at this temperature for 12 h. Then 30% H_2O_2 aq. (2.50 mL) and 3.0 M NaOH aq. (2.50 mL) were added and the reaction mixture was slowly warmed to rt. The resulting solution was extracted with ether (3 x 15 mL). The organic layers were combined, washed with brine (3 x 20 mL), filtered through a pad of Celite, dried over Na_2SO_4, filtered, and concentrated. After work-up: $syn_{1,2}syn_{2,3}$-**4aa**, single diastereomer. The residue was purified by SiO_2-chromatography (40 g; 1:5 EtOAc–Hex eluent) to give the product (1*R*,2*R*,3*S*)- $syn_{1,2}syn_{2,3}$-**4aa** as a colorless oil (127 mg, 91.9% yield). ^{1}H NMR ($CDCl_3$) δ 1.09 (s, 9H, $C(CH_3)_3$), 1.26 (d, *J* = 6.20 Hz, 3H, $CH(OH)CH_3$), 2.63 (dd, *J* = 6.20, 10.8 Hz, 1H, $CHCO_2C(CH_3)_3$), 3.17 (d, *J* = 10.8 Hz, 1H, OH), 3.85 (dd, *J* = 10.8, 10.8 Hz, 1H, PhC*H*CH=CH_2), 4.02–4.05 (m, 1H, C*H*(OH)CH_3), 5.12 (d, *J* = 10.8 Hz, 1H, PhCHCH=C*H*H), 5.23 (d, *J* = 16.5 Hz, 1H, PhCHCH=CH*H*), 5.89–5.95 (m, 1H, PhCHC*H*=CH_2), 7.18–7.29 (m, 5H, ArH); ^{13}C NMR ($CDCl_3$) δ 22.68, 27.73, 50.10, 56.44, 65.97, 81.61, 117.13, 126.87, 128.50, 128.57, 138.99, 141.60, 173.71; HRMS (ESI) calcd for $C_{17}H_{24}NaO_3$ [M+Na$^+$] 299.1623, found 299.1624; $[\alpha]_D^{23}$ –38.283 (*c* 0.628, $CHCl_3$).

Enantiomer ratio: 99.9:0.1 (4.60 mmϕ x 250 mm DAICEL CHIRALPAK IA-3 column; 2.0:98.0 2-PrOH–Hex eluent; 1.00 mL/min flow rate; 220-nm detection; 25 °C; t_R, 14.2 min (1*S*,2*R*,3*S*; major), 15.2 min (1*R*,2*S*,3*R*; minor)). **Supplementary Figures 120** and **121** showed the ^{1}H- and ^{13}C-NMR spectra and HPLC charts, respectively.

The abs confign was determined by the X-ray crystallographic analysis of (2*R*,3*S*)-2-((*R*)-1-hydroxyethyl)-3-phenylpent-4-enoic acid ((1*R*,2*R*,3*S*)-$syn_{1,2}syn_{2,3}$-**11aa**), which was prepared by hydrolysis of (1*R*,2*R*,3*S*)-$syn_{1,2}syn_{2,3}$-**4aa** under acidic condition.

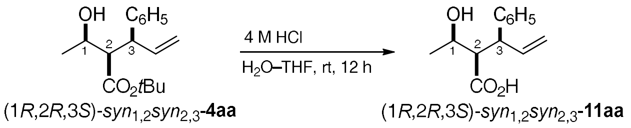

To a solution of (1*R*,2*R*,3*S*)-*syn*$_{1,2}$*syn*$_{2,3}$-**4aa** (140 mg, 0.507 mmol) in THF (2.00 mL) was added 4 M HCl aq. (8.00 mL) at rt. After 12 h at rt, the mixture was extracted with $CHCl_3$ (10 mL x 3). The organic extracts were dried over Na_2SO_4, filtered, and concentrated under vacuum to afford (1*R*,2*R*,3*S*)-*syn*$_{1,2}$*syn*$_{2,3}$-**11aa** (109 mg, 97.6% yield) as off-white solid. ^1H NMR ($CDCl_3$) δ 1.28 (d, *J* = 7.20 Hz, 3H, CH(OH)C*H*$_3$), 2.78 (dd, *J* = 10.8, 10.8 Hz, 1H, C*H*COOH), 3.85 (dd, *J* = 10.8, 10.8 Hz, 1H, PhC*H*CH=CH$_2$), 4.10–4.12 (m, 1H, C*H*(OH)CH$_3$), 5.14 (d, *J* = 10.2 Hz, 1H, PhCHCH=C*H*H), 5.24 (d, *J* = 16.2 Hz, 1H, PhCHCH=CH*H*), 5.92–5.98 (ddd, *J* = 10.2, 10.8, 16.2 Hz, 1H, PhCHC*H*=CH$_2$), 7.19–7.29 (m, 5H, ArH); ^{13}C NMR ($CDCl_3$) δ 22.45, 49.85, 56.32, 65.80, 117.55, 127.14, 127.95, 128.81, 138.39, 141.14, 176.94; HRMS (ESI) calcd for $C_{13}H_{15}O_3$ [M–H$^-$] 219.1021, found 219.1027; $[\alpha]_D^{22}$ –73.111 (*c* 0.497, $CHCl_3$). **Supplementary Figure 122** showed the ^1H- and ^{13}C-NMR spectra of (1*R*,2*R*,3*S*)-*syn*$_{1,2}$*syn*$_{2,3}$-**11aa**.

Recrystallization: (1*R*,2*R*,3*S*)-*syn*$_{1,2}$*syn*$_{2,3}$-**11aa** (10.0 mg); overlay of Hex (2 mL) on $CHCl_3$ solution of (1*R*,2*R*,3*S*)-*syn*$_{1,2}$*syn*$_{2,3}$-**11aa** (0.5 mL); rt/12 h; crystallization yield, ca. 60%; mp 136 °C; Flack, 0.11(4) for (1*R*,2*R*,3*S*)-*syn*$_{1,2}$*syn*$_{2,3}$-**11aa**. **Supplementary Table 10** summarized the crystallographic data, and the molecular structure in a crystal was shown in **Supplementary Figure 123**. Crystallographic data have been deposited with Cambridge Crystallographic Data Centre as supplementary publication no. CCDC-2193045.

<u>*tert*-Butyl (2*S*,3*R*)-2-((*S*)-1-hydroxyethyl)-3-phenylpent-4-enoate ((1*S*,2*S*,3*R*)-*syn*$_{1,2}$*syn*$_{2,3}$-**4aa**)</u>

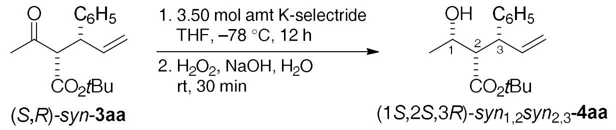

Reaction conditions (**Figure 4**): (*S*,*R*)-*syn*-**3aa** (137 mg, 0.500 mmol, 1.00 mol amt, >99:1 dr, >99:1 er); K-selectride (1.0 M in THF, 1.75 mL, 1.75 mmol, 3.50 mol amt); THF (5.00 mL); –78 °C; 12 h. Work up: 30% H_2O_2 aq. (2.50 mL), 3.0 M NaOH aq. (2.50 mL); 25 °C; 30 min. After work-up: *syn*$_{1,2}$*syn*$_{2,3}$-**4**, single diastereomer. Purification: SiO_2-chromatography (30 g; 1:10 EtOAc–Hex). Results: (1*S*,2*S*,3*R*)-*syn*$_{1,2}$*syn*$_{2,3}$-**4aa**, 121 mg, 87.2% yield, single diastereomer. $[\alpha]_D^{23}$ +39.788 (*c* 0.660, $CHCl_3$).

Enantiomer ratio: 99.9:0.1 (4.60 mmϕ x 250 mm DAICEL CHIRALPAK IA-3 column; 2.0:98.0 2-PrOH–Hex eluent; 1.00 mL/min flow rate; 220-nm detection; 25 °C; t_R, 14.2 min (1*R*,2*R*,3*S*; minor), 15.2 min (1*S*,2*S*,3*R*; major)). **Supplementary Figure 121** showed the HPLC charts.

<u>*tert*-Butyl (2*R*,3*R*)-2-((*R*)-1-hydroxyethyl)-3-phenylpent-4-enoate ((1*R*,2*R*,3*R*)-*syn*$_{1,2}$*anti*$_{2,3}$-**4aa**)</u>

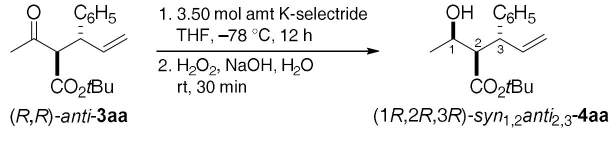

Reaction conditions (**Figure 4**): (*R*,*R*)-*anti*-**3aa** (137 mg, 0.500 mmol, 1.00 mol amt, >99:1 dr, >99:1 er); K-selectride (1.0 M in THF, 1.75 mL, 1.75 mmol, 3.50 mol amt); THF (5.00 mL); –78 °C; 12 h. Work up: 30% H_2O_2 aq. (2.50 mL), 3.0 M NaOH aq. (2.50 mL); 25 °C; 30 min. After work up: 98.8:1.2 dr. Purification: SiO_2-chromatography (30 g; 1:10 EtOAc–Hex). Results: (1*R*,2*R*,3*R*)-*syn*$_{1,2}$*anti*$_{2,3}$-**4aa**, 123 mg, 89.1% yield, single diastereomer. ^{1}H NMR ($CDCl_3$) δ 1.13 (d, *J* = 6.60 Hz, 3H, CH(OH)C*H*$_3$), 1.49 (s, 9H, $C(CH_3)_3$), 2.63 (dd, *J* = 3.00, 9.00 Hz, 1H, C*H*$CO_2C(CH_3)_3$), 3.02 (d, *J* = 10.2 Hz, 1H, OH), 3.39–3.42 (m, 1H, C*H*(OH)CH_3), 3.83 (dd, *J* = 9.00, 10.8 Hz, 1H, PhC*H*CH=CH_2), 5.03 (d, *J* = 10.2 Hz, 1H, PhCHCH=C*H*H), 5.13 (d, *J* = 16.8 Hz, 1H, PhCHCH=CH*H*), 6.03 (ddd, *J* = 10.2, 10.8, 16.8 Hz, 1H, PhCHC*H*=CH_2), 7.21–7.33 (m, 5H, ArH); ^{13}C NMR ($CDCl_3$) δ 19.85, 28.22, 49.54, 58.09, 67.72, 81.3, 116.16, 126.98, 128.04, 128.95, 139.21, 141.55, 172.13; HRMS (ESI) calcd for $C_{17}H_{24}NaO_3$ [M+Na$^+$] 299.1623, found 299.1625; $[\alpha]_D^{23}$ –43.233 (*c* 0.455, $CHCl_3$).

Enantiomer ratio: 99.9:0.1 (4.60 mmϕ x 250 mm DAICEL CHIRALPAK IA-3 column; 2.0:98.0 2-PrOH–Hex eluent; 1.00 mL/min flow rate; 220-nm detection; 25 °C; t_R, 16.4 min (1*S*,2*S*,3*S*; minor), 18.7 min (1*R*,2*R*,3*R*; major)). **Supplementary Figures 124** and **125** showed the ^{1}H- and ^{13}C-NMR spectra and HPLC charts, respectively.

The abs confign was determined by the X-ray crystallographic analysis of (2*R*,3*R*)-2-((*R*)-1-hydroxyethyl)-3-phenylpent-4-enoic acid ((1*R*,2*R*,3*R*)-*syn*$_{1,2}$*anti*$_{2,3}$-**11aa**), which was prepared by hydrolysis of (1*R*,2*R*,3*R*)-*syn*$_{1,2}$*anti*$_{2,3}$-**4aa** under acidic condition.

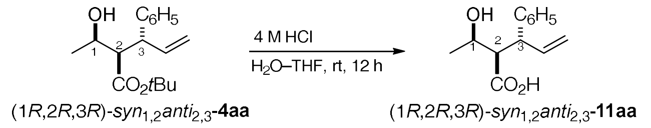

To a solution of (1*R*,2*R*,3*R*)-*syn*$_{1,2}$*anti*$_{2,3}$-**4aa** (100 mg, 0.362 mmol) in THF (1.50 mL) was added 4 M HCl aq. (6.00 mL) at rt. After 12 h at rt, the mixture was extracted with $CHCl_3$ (10 mL x 3). The organic extracts were dried over Na_2SO_4, filtered, and concentrated under vacuum to afford (1*R*,2*R*,3*R*)-*syn*$_{1,2}$*anti*$_{2,3}$-**11aa** (78.3 mg, 98.7% yield) as white solid. ^{1}H NMR ($CDCl_3$) δ 1.21 (d, *J* = 6.60 Hz, 3H, CH(OH)C*H*$_3$), 2.78 (dd, *J* = 2.40, 11.4 Hz, 1H, C*H*COOH), 3.54 (m, 1H, C*H*(OH)CH_3), 3.85 (dd, *J* = 10.8, 10.8 Hz, 1H, PhC*H*CH=CH_2), 5.07 (d, *J* = 10.2 Hz, 1H, PhCHCH=C*H*H), 5.16 (d, *J* = 17.4 Hz, 1H, PhCHCH=CH*H*), 6.07 (ddd, *J* = 10.2, 10.8, 17.4 Hz, 1H, PhCHC*H*=CH_2), 7.25–7.36 (m, 5H, ArH); ^{13}C NMR ($CDCl_3$) δ 22.45, 49.87, 57.25, 65.52, 116.60, 127.24, 128.09, 129.09, 138.69, 140.66, 178.00; HRMS (ESI) calcd for $C_{13}H_{15}O_3$ [M–H$^-$] 219.1021, found 219.1022; $[\alpha]_D^{22}$ +60.790 (*c* 0.512, $CHCl_3$). **Supplementary Figure 126** showed the ^{1}H- and ^{13}C-NMR spectra of (1*R*,2*R*,3*R*)-*syn*$_{1,2}$*anti*$_{2,3}$-**11aa**.

Recrystallization: (1*R*,2*R*,3*R*)-*syn*$_{1,2}$*anti*$_{2,3}$-**11aa** (10.0 mg); overlay of Hex (2 mL) on $CHCl_3$ solution of (1*R*,2*R*,3*R*)-*syn*$_{1,2}$*anti*$_{2,3}$-**11aa** (0.5 mL); rt/24 h; crystallization yield, ca. 40%; mp 108.5 °C; Flack, 0.07(7) for (1*R*,2*R*,3*R*)-*syn*$_{1,2}$*anti*$_{2,3}$-**11aa**. **Supplementary Table 11** summarized the crystallographic data, and the molecular structure in a crystal was shown in **Supplementary Figure 127**. Crystallographic data have been deposited with Cambridge Crystallographic Data Centre as supplementary publication no. CCDC-2193050.

tert-Butyl (2*S*,3*S*)-2-((*S*)-1-hydroxyethyl)-3-phenylpent-4-enoate ((1*S*,2*S*,3*S*)-*syn*$_{1,2}$*anti*$_{2,3}$-**4aa**)

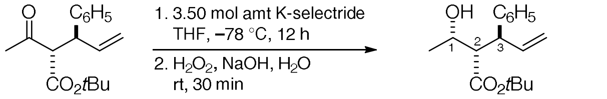

(*S*,*S*)-*anti*-**3aa** (1*S*,2*S*,3*S*)-*syn*$_{1,2}$*anti*$_{2,3}$-**4aa**

Reaction conditions (**Figure 4**): (*S*,*S*)-*anti*-**3aa** (137 mg, 0.500 mmol, 1.00 mol amt, >99:1 dr, >99:1 er); K-selectride (1.0 M in THF, 1.75 mL, 1.75 mmol, 3.50 mol amt); THF (5.00 mL); –78 °C; 12 h. Work up: 30% H_2O_2 aq. (2.50 mL), 3.0 M NaOH aq. (2.50 mL); 25 °C; 30 min. After work up: 98.7:1.3 dr. Purification: SiO_2-chromatography (30 g; 1:10 EtOAc–Hex). Results: (1*S*,2*S*,3*S*)-*syn*$_{1,2}$*anti*$_{2,3}$-**4**, 118 mg, 85.4% yield, single diastereomer. $[\alpha]_D^{23}$ +44.059 (*c 0*.577, $CHCl_3$).

Enantiomer ratio: 99.6:0.4 (4.60 mmϕ x 250 mm DAICEL CHIRALPAK IA-3 column; 2.0:98.0 2-PrOH–Hex eluent; 1.00 mL/min flow rate; 220-nm detection; 25 °C; t_R, 18.0 min (1*S*,2*S*,3*S*; major), 20.4 min (1*R*,2*R*,3*R*; minor). **Supplementary Figure 125** showed the HPLC charts.

6.2. *Syn*-selective reduction using LaCl$_3$.

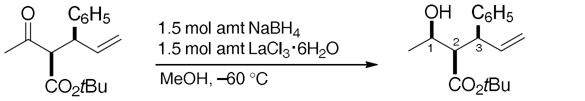

(*R*,*S*)-*syn*-**3aa** (1*R*,2*R*,3*S*)-*syn*$_{1,2}$*syn*$_{2,3}$-**4aa**

A 10 mL Young-type Schlenk tube was charged with (*R*,*S*)-*syn*-**3aa** (137 mg, 0.500 mmol, 1.00 mol amt), $LaCl_3\cdot 6H_2O$ (265 mg, 0.750 mmol) and dry MeOH (2.50 mL). The solution was cooled to –60 °C and $NaBH_4$ (28.5 mg, 0.750 mmol) was added. The reaction mixture was stirred at this temperature for 0.5 h. Sat. NH_4Cl aq. (ca. 5 mL) was added carefully, and the reaction mixture was slowly warmed to rt. The resulting solution was extracted with EtOAc (5 x 5 mL). The organic layers were combined, washed with brine (3 x 10 mL), dried over Na_2SO_4, filtered, and concentrated. After work up: a mixture of (1*S*,2*R*,3*S*)- *syn*$_{1,2}$*syn*$_{2,3}$-**4aa** and (1*R*,2*R*,3*S*)- *anti*$_{1,2}$*syn*$_{2,3}$-**4aa**, 92.5:7.5 dr. **Supplementary Figure 128** showed a ^{1}H-NMR spectrum of the reaction mixture.

6.3. *Anti*-selective reduction using Zn(BH$_4$)$_2$.

tert-Butyl (2*R*,3*S*)-2-((*S*)-1-hydroxyethyl)-3-phenylpent-4-enoate ((1*S*,2*R*,3*S*)-*anti*$_{1,2}$*syn*$_{2,3}$-**4aa**)

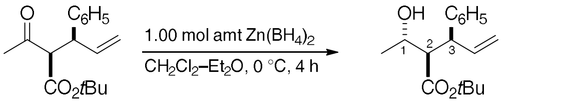

(*R*,*S*)-*syn*-**3aa** (1*S*,2*R*,3*S*)-*anti*$_{1,2}$*syn*$_{2,3}$-**4aa**

The detailed operation of diastereoselective reduction of **3aa** using $Zn(BH_4)_2$ was described by taking the reduction of (*R*,*S*)-*syn*-**3aa** as the representative (**Figure 4**). A 20 mL Young-type Schlenk tube was charged with (*R*,*S*)-*syn*-**3aa** (137 mg, 0.500 mmol, 1.00 mol amt), and dry CH_2Cl_2 (10.0 mL). The solution was cooled to 0 °C and $Zn(BH_4)_2$ (0.500 M in Et_2O, 1.00 mL, 0.500 mmol, 1.00 mol amt) was added dropwise at 0 °C and the reaction mixture was stirred at this temperature for 4 h. Then sat. NH_4Cl aq. (ca. 10 mL) were added and the reaction mixture was slowly warmed to rt. The resulting solution was extracted with CH_2Cl_2 (3 x 15 mL). The organic layers were

combined, washed with brine (3 x 20 mL), dried over Na_2SO_4, filtered, and concentrated. After work-up: *anti*$_{1,2}$*syn*$_{2,3}$-**4aa**, single diastereomer. The residue was purified by SiO_2-chromatography (20 g; 1:10 EtOAc–Hex eluent) to give the product (1*S*,2*R*,3*S*)-*anti*$_{1,2}$*syn*$_{2,3}$-**4aa** as an off-white solid (131 mg, 95.2% yield). ^{1}H NMR ($CDCl_3$) δ 1.14 (s, 9H, $C(CH_3)_3$), 1.27 (d, *J* = 7.20 Hz, 3H, CH(OH)C*H*$_3$), 2.44 (d, *J* = 5.40 Hz, 1H, OH), 2.96 (dd, *J* = 5.40, 10.8 Hz, 1H, C*H*$CO_2C(CH_3)_3$), 3.60 (dd, *J* = 10.2, 10.8 Hz, 1H, PhC*H*CH=CH_2), 4.10–4.13 (m, 1H, C*H*(OH)CH_3), 5.06 (d, *J* = 10.2 Hz, 1H, PhCHCH=C*H*H), 5.21 (d, *J* = 16.8 Hz, 1H, PhCHCH=CH*H*), 6.01 (ddd, *J* = 10.2, 10.2, 16.8 Hz, 1H, PhCHC*H*=CH_2), 7.17–7.29 (m, 5H, ArH); ^{13}C NMR ($CDCl_3$) δ 19.64, 27.79, 51.86, 58.14, 68.75, 81.15, 116.07, 126.80, 128.04, 128.62, 140.78, 142.14, 171.64; HRMS (ESI) calcd for $C_{17}H_{24}NaO_3$ [M+Na$^+$] 299.1623, found 299.1622; $[\alpha]_D^{23}$ –78.646 (*c* 0.554, $CHCl_3$).

Enantiomer ratio: 99.4:0.6 (4.60 mmϕ x 250 mm DAICEL CHIRALPAK ID-3 column; 1.0:99.0 2-PrOH–Hex eluent; 0.50 mL/min flow rate; 220-nm detection; 25 °C; t_R, 32.3 min (1*R*,2*S*,3*R*; minor), 34.1min (1*S*,2*R*,3*S*; major)). **Supplementary Figures 129** and **130** showed the ^{1}H- and ^{13}C-NMR spectra and HPLC charts, respectively.

<u>*tert*-Butyl (2*S*,3*R*)-2-((*R*)-1-hydroxyethyl)-3-phenylpent-4-enoate ((1*R*,2*S*,3*R*)-*anti*$_{1,2}$*syn*$_{2,3}$-**4aa**)</u>

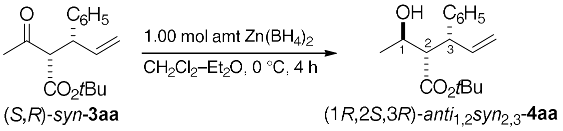

Reaction conditions (**Figure 4**): (*S*,*R*)-*anti*-**3aa** (137 mg, 0.500 mmol, 1.00 mol amt, >99:1 dr, >99:1 er); $Zn(BH_4)_2$ (0.500 M in Et_2O, 1.00 mL, 0.500 mmol, 1.00 mol amt); CH_2Cl_2 (10.0 mL); 0 °C; 4 h. Work up: sat. NH_4Cl aq. (ca. 10 mL). After work-up: *anti*$_{1,2}$*syn*$_{2,3}$-**4**, single diastereomer. Purification: SiO_2-chromatography (30 g; 1:10 EtOAc–Hex). Results: (1*R*,2*S*,3*R*)-*anti*$_{1,2}$*syn*$_{2,3}$-**4**, 129 mg, 93.3% yield, single diastereomer. $[\alpha]_D^{23}$ +78.809 (*c* 0.603, $CHCl_3$).

Enantiomer ratio: 99.9:0.1 (4.60 mmϕ x 250 mm DAICEL CHIRALPAK ID-3 column; 1.0:99.0 2-PrOH–Hex eluent; 0.50 mL/min flow rate; 220-nm detection; 25 °C; t_R, 31.0 min (1*R*,2*S*,3*R*; major), 34.9 min (1*S*,2*R*,3*S*; minor)). **Supplementary Figure 130** showed the and HPLC charts.

<u>*tert*-Butyl (2*R*,3*R*)-2-((*S*)-1-hydroxyethyl)-3-phenylpent-4-enoate ((1*S*,2*R*,3*R*)-*anti*$_{1,2}$*anti*$_{2,3}$-**4aa**)</u>

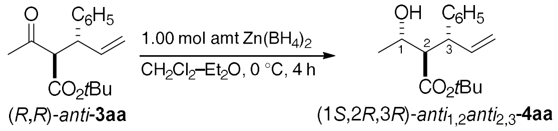

Reaction conditions (**Figure 4**): (*R*,*R*)-*anti*-**3aa** (137 mg, 0.500 mmol, 1.00 mol amt, >99:1 dr, >99:1 er); $Zn(BH_4)_2$ (0.500 M in Et_2O, 1.00 mL, 0.500 mmol, 1.00 mol amt); CH_2Cl_2 (10.0 mL); 0 °C; 4 h. Work up: sat. NH_4Cl aq. (ca. 10 mL). After work-up: *anti*$_{1,2}$*syn*$_{2,3}$-**4aa**, single diastereomer. Purification: SiO_2-chromatography (30 g; 1:10 EtOAc–Hex). Results: (1*S*,2*R*,3*R*)-*anti*$_{1,2}$*anti*$_{2,3}$-**4aa**, 126 mg, 91.1% yield, single diastereomer. ^{1}H NMR ($CDCl_3$) δ 1.12 (d, *J* = 6.60 Hz, 3H, CH(OH)C*H*$_3$), 1.42 (s, 9H, $C(CH_3)_3$), 1.89 (d, *J* = 4.80 Hz, 1H, OH), 2.88 (dd, *J* = 6.60, 9.00 Hz, 1H, C*H*$CO_2C(CH_3)_3$), 3.69 (dd, *J* = 9.00, 9.00 Hz, 1H, PhC*H*CH=CH_2), 3.79–3.82 (m, 1H, C*H*(OH)CH_3), 5.08 (d, *J* =

10.2 Hz, 1H, PhCHCH=C*H*H), 5.21 (d, *J* = 17.4 Hz, 1H, PhCHCH=CH*H*), 6.01 (ddd, *J* = 9.00, 10.2, 17.4 Hz, 1H, PhCHC*H*=CH_2), 7.21–7.32 (m, 5H, ArH); ^{13}C NMR ($CDCl_3$) δ 19.85, 28.22, 49.54, 58.09, 67.72, 81.43, 116.15, 126.98, 128.08, 128.95, 139.21, 141.55, 172.13; HRMS (ESI) calcd for $C_{17}H_{24}NaO_3$ [$M+Na^+$] 299.1623, found 299.1624; $[\alpha]_D^{23}$ +43.019 (*c* 0.529, $CHCl_3$).

Enantiomer ratio: 99.9:0.1 (4.60 mmϕ x 250 mm DAICEL CHIRALPAK ID-3 column; 1.0:99.0 2-PrOH–Hex eluent; 0.50 mL/min flow rate; 220-nm detection; 25 °C; t_R, 34.3 min (1*R*,2*S*,3*S*; minor), 38.3 min (1*S*,2*R*,3*R*; major)). **Supplementary Figures 131** and **132** showed the ^{1}H- and ^{13}C-NMR spectra and HPLC charts, respectively.

The abs confign was determined by the X-ray crystallographic analysis of (2*R*,3*R*)-2-((*S*)-1-hydroxyethyl)-3-phenylpent-4-enoic acid ((1*S*,2*R*,3*R*)-$syn_{1,2}anti_{2,3}$-**11aa**), which was prepared by hydrolysis of (1*S*,2*R*,3*R*)-$anti_{1,2}anti_{2,3}$-**4aa** under acidic condition.

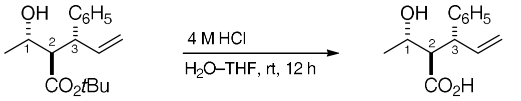

(1*S*,2*R*,3*R*)-$anti_{1,2}anti_{2,3}$-**4aa** (1*S*,2*R*,3*R*)-$anti_{1,2}anti_{2,3}$-**11aa**

To a solution of (1*S*,2*R*,3*R*)-$anti_{1,2}anti_{2,3}$-**4aa** (100 mg, 0.362 mmol) in THF (1.50 mL) was added 4 M HCl aq. (6.00 mL) at rt. After 12 h at rt, the mixture was extracted with $CHCl_3$ (10.0 mL x 3). The organic extracts were dried over Na_2SO_4, filtered, and concentrated under vacuum to afford (1*S*,2*R*,3*R*)-$syn_{1,2}anti_{2,3}$-**11aa** (76.8 mg, 95.4% yield) as white solid. ^{1}H NMR ($CDCl_3$) δ 1.19 (d, *J* = 6.00 Hz, 3H, CH(OH)C*H*$_3$), 3.09 (dd, *J* = 5.40, 9.60 Hz, 1H, C*H*COOH), 3.69 (dd, *J* = 9.00, 9.60 Hz, 1H, PhC*H*CH=CH_2), 3.83–3.85 (m, 1H, C*H*(OH)CH_3), 5.09 (d, *J* = 10.2 Hz, 1H, PhCHCH=C*H*H), 5.13 (d, *J* = 16.8 Hz, 1H, PhCHCH=CH*H*), 6.09 (ddd, *J* = 9.00, 10.2, 16.8 Hz, 1H, PhCHC*H*=CH_2), 7.23–7.34 (m, 5H, ArH); ^{13}C NMR ($CDCl_3$) δ 19.39, 49.37, 57.14, 67.40, 116.48, 127.27, 128.00, 129.15, 138.99, 140.79, 177.19; HRMS (ESI) calcd for $C_{13}H_{15}O_3$ [$M-H^-$] 219.1021, found 219.1028; $[\alpha]_D^{23}$ +21.023 (*c* 0.673, $CHCl_3$). **Supplementary Figure 133** showed the ^{1}H- and ^{13}C-NMR spectra of (1*S*,2*R*,3*R*)-$anti_{1,2}anti_{2,3}$-**11aa**.

Recrystallization: (1*S*,2*R*,3*R*)-$anti_{1,2}anti_{2,3}$-**11aa** (10.0 mg); overlay of Hex (2 mL) on $CHCl_3$ solution of (1*S*,2*R*,3*R*)-$anti_{1,2}anti_{2,3}$-**11aa** (0.5 mL); rt/4 h; crystallization yield, ca. 50%; mp 105 °C; Flack, 0.11(15) for (1*S*,2*R*,3*R*)-$anti_{1,2}anti_{2,3}$-**11aa**. **Supplementary Table 12** summarized the crystallographic data, and the molecular structure in a crystal was shown in **Supplementary Figure 134**. Crystallographic data have been deposited with Cambridge Crystallographic Data Centre as supplementary publication no. CCDC-2193051.

tert-Butyl (2*S*,3*S*)-2-((*R*)-1-hydroxyethyl)-3-phenylpent-4-enoate ((1*R*,2*S*,3*S*)-$anti_{1,2}anti_{2,3}$-**4aa**)

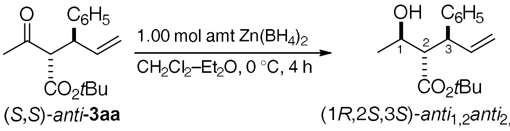

(*S*,*S*)-*anti*-**3aa** (1*R*,2*S*,3*S*)-$anti_{1,2}anti_{2,3}$-**4aa**

Reaction conditions (**Figure 4**): (*S*,*S*)-*anti*-**3aa** (137 mg, 0.500 mmol, 1.00 mol amt, >99:1 dr, >99:1 er); $Zn(BH_4)_2$ (0.500 M in Et_2O, 1.00 mL, 0.500 mmol, 1.00 mol amt); CH_2Cl_2 (10.0 mL); 0 °C; 4 h. Work up: sat. NH_4Cl aq. (ca. 10 mL). After work-up:

anti$_{1,2}$*syn*$_{2,3}$-**4**, single diastereomer. Purification: SiO_2-chromatography (30 g; 1:10 EtOAc–Hex). Results: (1*R*,2*S*,3*S*)-*anti*$_{1,2}$*anti*$_{2,3}$-**4aa**, 121 mg, 87.6% yield, single diastereomer. $[\alpha]_D^{23}$ –41.842 (*c* 0.405, $CHCl_3$).

Enantiomer ratio: 99.9:0.1 (4.60 mmϕ x 250 mm DAICEL CHIRALPAK ID-3 column; 1.0:99.0 2-PrOH–Hex eluent; 0.50 mL/min flow rate; 220-nm detection; 25 °C; t_R, 34.9 min (1*R*,2*S*,3*S*; major), 40.0 min (1*S*,2*R*,3*R*; minor)). **Supplementary Figure 132** showed the HPLC charts.

7. Condition optimization.

The results using other solvents and/or under different temperatures were listed in **Supplementary Table 13**.

8. Formal synthesis of (+)-pancratistatin.

Synthesis of *tert*-butyl (2*R*,3*R*,*E*)-2-((*S*)-1-(benzo[*d*][1,3]dioxol-5-yl)allyl)-3-hydroxyhex-4-enoate ((1*R*,2*R*,3*S*)-*syn*$_{1,2}$*syn*$_{2,3}$-**5**)

A 200 mL Young-type Schlenk tube was charged with $[RuCp(CH_3CN)_3]PF_6$ (43.2 mg, 100 μmol), $\mathbf{L^2}_{S}$ (54.6 mg, 100 μmol), and CH_2Cl_2 (20.0 mL). The mixture was stirred at rt for 30 min, and then the resulting pale-yellow solution was concentrated *in vacuo*. To this were added Pd$\mathbf{L^1}_{R}$(OTf) (106 mg, 100 μmol), **2q** (4.27 g, 20.0 mmol) and a 0.500 M solution of **1n** in 1,4-dioxane (40.0 mL) under Ar atmosphere. The resulting red-brown solution was stirred at 25 °C for 48 h. The reaction was finished after 48 h and the *syn*/*anti* ratio was determined as 99.1:0.9 by ^{1}H-NMR analysis. **Supplementary Figure 135** showed a ^{1}H-NMR spectrum of the reaction mixture.

The reaction mixture was concentrated *in vacuo*. To this was added 100 mL of dry MeOH and $LaCl_3 \cdot 6H_2O$ (10.6 g, 30.0 mmol). The mixture was then cooled to –60 °C and $NaBH_4$ (1.13 g, 30.0 mmol) was added in portions. The reaction mixture was stirred at this temperature for 0.5 h. Sat. NH_4Cl aq. (ca. 100 mL) was added carefully and the reaction mixture was slowly warmed to rt. The resulting solution was extracted with EtOAc (5 x 100 mL). The organic layers were combined, washed with brine (3 x 200 mL), dried over Na_2SO_4, filtered, and concentrated. After work up: 98.7:1.3 dr. The residue was purified by SiO_2-chromatography (500 g; 1:8 EtOAc–Hex eluent) to give the product *syn*$_{1,2}$*syn*$_{2,3}$-**5** as a white solid (6.38 g, 92.1% yield, single diastereomer). ^{1}H NMR ($CDCl_3$) δ 1.14 (s, 9H, $C(CH_3)_3$), 1.69 (d, *J* = 6.00 Hz, 3H, CH=CHC*H*$_3$), 2.64 (dd, *J* = 1.80, 10.8 Hz, 1H, CHO*H*), 3,42 (d, *J* = 10.2 Hz, 1H, C*H*$CO_2C(CH_3)_3$), 3.81 (dd, *J* = 10.2, 10.2 Hz, 1H, C*H*OH), 4.29–4.30 (m, 1H, ArC*H*CH=CH_2), 5.14 (d, *J* = 10.2 Hz, 1H, ArCHCH=CH*H*), 5.24 (d, *J* = 16.2 Hz, 1H, ArCHCH=CH*H*), 5.48–5.52 (m, 1H, CH=C*H*CH_3), 5.74 (m, 1H, C*H*=$CHCH_3$), 5.85–5.91 (m, 1H, ArCHC*H*=CH_2), 5.91 (s, 2H, OCH_2O), 6.68–6.73 (m, 3H, ArH); ^{13}C NMR ($CDCl_3$) δ 17.81, 27.84, 49.29, 55.60, 70.10, 81.53, 100.98, 108.27, 108.88, 117.20, 121.62, 126.33, 132.06, 135.34, 138.69, 146.35, 147.62, 173.36; HRMS (ESI) calcd for $C_{20}H_{26}NaO_5$ [M+Na$^+$] 369.1678, found 369.1678; $[\alpha]_D^{22}$ –35.038 (*c* 0.693, CH_3OH).

Determination of er: 99.9:0.1 (4.60 mmϕ x 250 mm DAICEL CHIRALPAK IE-3 column; 2.0:98.0 2-PrOH–Hex eluent; 1.00 mL/min flow rate; 220-nm detection; 25 °C;

t_R, 17.5 min (1*S*,2*S*,3*R*; minor), 18.6 min (1*R*,2*R*,3*S*; major)). **Supplementary Figures 136** and **137** showed the ^{1}H- and ^{13}C-NMR spectra and HPLC charts, respectively.

Synthesis of *tert*-butyl (2*R*,3*S*)-3-(benzo[*d*][1,3]dioxol-5-yl)-2-((*S*)-hydroxy((2*S*,3*S*)-3-methyloxiran-2-yl)methyl)pent-4-enoate (**6**).

A 100 mL Young-type Schlenk tube was charged with (1*R*,2*R*,3*S*)-**5** (3.46 g, 10.0 mmol) and dry CH_2Cl_2 (50.0 mL). The mixture was then cooled to –40 °C. To this was added VO(acac)$_2$ (132 mg, 0.500 mmol, 5.00 mol%) and the reaction mixture was stirred at this temperature for 15 min. TBHP (5.00 M in dodecane, 4.00 mL, 20.0 mmol, 2.00 mol amt) was added dropwise and the reaction mixture was stirred at –40 °C for 24 h. The resulting brown solution was then filtered by a short-pad of SiO_2 and washed twice by 1:8 EtOAc–Hex. The filtrate was concentrated and purified by SiO_2-chromatography (200 g; 1:8 EtOAc–Hex eluent) to give the product **6** as a mixture of two diastereomer (3.42 g, 91.9:8.1 dr, 94.4% yield). ^{1}H NMR ($CDCl_3$) δ 1.17 (s, 9H, C(CH_3)$_3$), 1.33 (d, *J* = 5.40 Hz, 3H, CHOCHC*H*$_3$), 2.67 (d, *J* = 5.40 Hz, 1H, CHOC*H*CH$_3$), 2.78 (d, *J* = 10.8 Hz, 1H, CHOC*H*CH$_3$), 3.01–3.02 (m, 1H, C*H*CO$_2$C(CH$_3$)$_3$), 3.69–3.71 (m, 1H, C*H*(OH)), 3.78 (dd, *J* = 9.60, 9.60 Hz, 1H, ArC*H*CH=CH$_2$), 5.12 (d, *J* = 9.60 Hz, 1H, ArCHCH=CH*H*), 5.24 (d, *J* = 16.8 Hz, 1H, ArCHCH=CH*H*), 5.84 (ddd, *J* = 9.60, 9.60, 16.8 Hz, 1H, ArCHC*H*=CH$_2$), 5.92 (s, 2H, OCH$_2$O), 6.68–6.74 (m, 3H, ArH); ^{13}C NMR ($CDCl_3$) δ17.47, 27.78, 49.46, 51.92, 53.51, 60.82, 70.02, 82.07, 101.04, 108.34, 108.88, 117.42, 121.61, 134.99, 138.52, 146.49, 147.71, 173.58; HRMS (ESI) calcd for $C_{20}H_{26}NaO_6$ [M+Na$^+$] 385.1627, found 385.1623; $[\alpha]_D^{22}$ –66.040 (*c* 0.676, CH_3OH). **Supplementary Figure 138** showed the ^{1}H- and ^{13}C-NMR spectra.

Synthesis of *tert*-butyl (2*R*,3*S*,4*R*)-2-((*S*)-1-(benzo[*d*][1,3]dioxol-5-yl)allyl)-3,4-dihydroxyhex-5-enoate (**7**):

A 100 mL two-necked round flask was charged diphenyl diselenide (2.59 g, 8.29 mmol, 1.00 mol amt) and dry EtOH (33.1 mL). The solution was cooled to 0 °C and to this was added $NaBH_4$ (631 mg, 16.7 mmol, 2.01 mol amt) in portions under Ar atmosphere. The resulting mixture was stirred in this temperature for 30 min and became a white suspension. To this mixture, a solution of **6** (3.00 g, 8.29 mmol) in dry EtOH (8.00 mL) was added dropwise. Reaction vessel was removed from the cold bath, stirred at 25 °C until judged complete by TLC (3 h). THF (10.0 mL) and sat. $NaHCO_3$ aq. (20.0 mL) was then added. 35% H_2O_2 aq. (20.0 mL) was added slowly, and the reaction mixture was stirred for 1 h. Upon completion, the aqueous phase was extracted with toluene (5 × 25 mL). To the combined organic extracts, were added sat. $NaHCO_3$ aq. (50.0 mL). The mixture was heated to 100 °C to 2 h and cooled to rt. The phases were separated, and the aqueous phase was extracted with toluene (2 × 100 mL). The combined organic extracts were washed with and brine (200 mL), dried over Na_2SO_4, filtered, and concentrated under reduced pressure. The residue was purified by flash chromatography (SiO_2, 1:5 to 1:3 EtOAc–Hex eluent) to give the desired compound as a white solid (2.15 g, 5.93 mmol, 71.6% yield). ^{1}H NMR ($CDCl_3$) δ 1.15 (s, 9H, C(CH$_3$)$_3$), 2.05 (d, *J* = 5.40 Hz, 1H, CHO*H*), 2.85 (d, *J* = 12.0 Hz, 1H, CH(O*H*)CH=CH$_2$), 3.74 (dd, *J* = 9.60, 9.60 Hz, 1H, C*H*CO$_2$C(CH$_3$)$_3$), 3.80 (dd, *J* = 5.40, 9.60 Hz, 1H, C*H*OH), 4.04 (d, *J* = 9.60 Hz, 1H, C*H*(OH)CH=CH$_2$), 4.18–4.19 (m, 1H, ArC*H*CH=CH$_2$), 5.12 (d, *J* = 9.60 Hz, 1H,

ArCHCH=CH*H*), 5.22 (d, *J* = 16.8 Hz, 1H, ArCHCH=CH*H*), 5.28 (d, *J* = 10.2 Hz, 1H, CH(OH)CH=C*H*H), 5.38 (d, *J* = 17.4 Hz, 1H, CH(OH)CH=C*H*H), 5.79–5.85 (m, 1H, ArCHC*H*=CH_2), 5.92 (s, 2H, OCH_2O), 5.93–5.99 (m, 1H, CH(OH)C*H*=CH_2), 6.68–6.74 (m, 3H, ArH); ^{13}C NMR ($CDCl_3$) δ 27.71, 49.43, 50.09, 73.32, 75.79, 81.84, 101.01, 108.30, 108.94, 116.82, 117.29, 121.70, 135.11, 137.10, 138.81, 146.43, 147.66, 174.27; HRMS (ESI) calcd for $C_{20}H_{26}NaO_6$ [M+Na$^+$] 385.1627, found 385.1624; $[\alpha]_D^{21}$ –44.697 (*c* 0.609, CH_3OH). **Supplementary Figure 139** showed the ^{1}H- and ^{13}C-NMR spectra.

<u>Synthesis of *tert*-butyl (1*R*,2*S*,5*R*,6*S*)-2-(benzo[*d*][1,3]dioxol-5-yl)-5,6-dihydroxycyclohex-3-ene-1-carboxylate (**8**):</u>

An 80 mL Young-type Schlenk tube was charged with **7** (1.45 g, 4.00 mmol) and dry CH_2Cl_2 (40.0 mL). To this was added Grubb's 2nd catalyst (33.8 mg, 40.0 μmol, 1.00 mol%) under Ar atmosphere and the reaction mixture was stirred at rt until judged complete by TLC (2.5 h). The resulting brown solution was then filtered by a short-pad of SiO_2 and washed twice by 1:2 EtOAc–Hex eluent. The filtrate was concentrated and purified by SiO_2-chromatography (100 g; 1:4 EtOAc–Hex eluent) to give the product **8** as an off-white solid (1.21 g, 3.63 mmol, 90.8% yield). ^{1}H NMR ($CDCl_3$) δ 1.33 (s, 9H, $C(CH_3)_3$), 2.26 (br, 1H, CHO*H*), 2.65 (dd, *J* = 10.8, 10.8 Hz, 1H, C*H*$CO_2C(CH_3)_3$), 2.71 (d, *J* = 6.60 Hz, 1H, CHO*H*), 3.56 (d, *J* = 10.8 Hz, 1H, C*H*OH), 3.94–3.97 (m, 1H, ArC*H*CH=CH_2), 4.28 (br, 1H, C*H*OH), 5.80 (d, *J* = 9.60 Hz, 1H, ArCHCH=C*H*), 5.94 (s, 2H, OCH_2O), 5.95–5.98 (m, 1H, ArCHC*H*=CH), 6.63–6.73 (m, 3H, ArH); ^{13}C NMR ($CDCl_3$) δ 28.14, 46.36, 51.91, 65.23, 70.93, 81.47, 101.11, 108.22, 108.48, 121.42, 126.10, 134.96, 135.77, 146.64, 147.86, 172.92; HRMS (ESI) calcd for $C_{18}H_{22}NaO_6$ [M+Na$^+$] 3571314, found 357.1312; $[\alpha]_D^{21}$ +99.863 (*c* 0.512, CH_3OH). **Supplementary Figure 140** showed the ^{1}H- and ^{13}C-NMR spectra.

Recrystallization: **8** (20.0 mg); overlay of Hex (2.00 mL) on $CHCl_3$ solution of **8** (2.00 mL); rt/24 h; crystallization yield, ca. 70%; mp 216.5 °C; Flack, 0.09(8) for (1*R*,2*S*,5*R*,6*S*)-**8**. **Supplementary Table 14** summarized the crystallographic data, and the molecular structure in a crystal was shown in **Supplementary Figure 141**. Crystallographic data have been deposited with Cambridge Crystallographic Data Centre as supplementary publication no. CCDC-2193066.

<u>Synthesis of (1*R*,2*S*,5*R*,6*S*)-2-(benzo[*d*][1,3]dioxol-5-yl)-5,6-dihydroxycyclohex-3-ene-1-carboxylic acid (**9**):</u>

A 100 mL round-bottomed flask was charged with **8** (1.00 g, 3.00 mmol), THF (6.00 mL) and 2 M HCl aq. (30.0 mL). The reaction mixture was stirred at 50 °C until judged complete by TLC (5 h). The resulting solution was extracted with EtOAc (5 x 50 mL). The organic layers were combined, dried over Na_2SO_4, filtered, and concentrated to give compound **9** as a white solid (804 mg, 2.89 mmol, 96.4% yield). ^{1}H NMR (CD_3OD) δ 2.73 (dd, *J* = 11.4, 11.4 Hz, 1H, C*H*COOH), 3.57 (d, *J* = 10.2 Hz, 1H, C*H*(OH)), 3.85 (dd, *J* = 4.20, 11.4 Hz, 1H, C*H*(OH)), 4.09 (dd, *J* = 4.80, 4.80 Hz, 1H, ArC*H*CH=CH), 5.72 (dd, *J* = 2.40, 9.60 Hz, 1H, ArCHCH=C*H*), 5.85–5.94 (m, 1H, ArCHC*H*=CH), 5.90 (s, 2H, OCH_2O), 6.65–6.72 (m, 3H, ArH); ^{13}C NMR (CD_3OD) δ 48.08, 52.77, 66.30, 72.20, 102.27, 109.06, 109.18, 122.22, 127.87, 134.67, 137.50, 148.03, 149.26, 177.64; HRMS

(ESI): calcd for $C_{14}H_{14}NaO_6$ [M+Na$^+$] 301.0688, found 301.0681; $[\alpha]_D^{20}$ +134.387 (*c* 0.605, CH_3OH). **Supplementary Figure 142** showed the ^{1}H- and ^{13}C-NMR spectra.

Synthesis of methyl ((1*R*,2*R*,5*R*,6*S*)-2-(benzo[*d*][1,3]dioxol-5-yl)-5,6-dihydroxycyclohex-3-en-1-yl)carbamate (**10**)

A 100 mL round-bottomed flask was charged with **9** (500 mg, 1.80 mmol) and toluene (7.20 mL). To this was added Et_3N (758 μL, 5.40 mmol, 3.00 mol amt) and DPPA (1.16 mL, 5.40 mmol, 3.00 mol amt). The reaction mixture was then heated to reflux (110 °C) and stirred at this temperature for 2 h. The resulting solution was concentrated *in vacuo*. To this was added CH_3OH (36.0 mL) and CH_3OK (382 mg, 5.41 mmol, 3.00 mol amt). The reaction mixture was heated to reflux (90 °C) for 30 min before cooling to rt. The reaction mixture was quenched with sat. NH_4Cl aq. (20 mL) and extracted with EtOAc (5 x 20 mL). The combined organic solution was dried by Na_2SO_4, filtered, concentrated, and purified by SiO_2-chromatography (50 g; 1:20 to 1:10 CH_3OH–CH_2Cl_2 eluent) to give the product **10** as a white solid (507 mg, 1.65 mmol, 91.7% yield). ^{1}H NMR (CD_3OD) δ 3.29 (m, 1H, C*H*NHCO$_2$CH$_3$), 3.50 (s, 3H, NHCO$_2$C*H$_3$*), 3.60–3.63 (m, 1H, C*H*(OH)), 3.77 (m, 1H, C*H*(OH)), 4.21 (t, *J* = 4.20 Hz, 1H, ArC*H*CH=CH), 5.68 (dd, *J* = 1.20, 9.60 Hz, 1H, ArCHCH=C*H*), 5.89 (s, 2H, OCH$_2$O), 5.90–5.92 (m, 1H, ArCHC*H*=CH), 6.67–6.74 (m, 3H, ArH); ^{13}C NMR (CD_3OD) δ 50.29, 52.31, 56.14, 67.93, 73.39, 102.18, 108.79, 109.57, 122.76, 127.86, 134.88, 137.25, 147.86, 149.09, 159.83; HRMS (ESI): calcd for $C_{15}H_{17}NNaO_6$ [M+Na$^+$] 330.0954, found 330.0952; $[\alpha]_D^{20}$ +114.082 (*c* 1.22, CH_3OH) (lit[21] $[\alpha]_D^{25}$ +107.7 (*c* 1.22, CH_3OH)). **Supplementary Figure 143** showed the ^{1}H- and ^{13}C-NMR spectra. The ^{1}H-, ^{13}C-NMR spectra and $[\alpha]_D^{20}$ of the product were consistent with those reported[23].

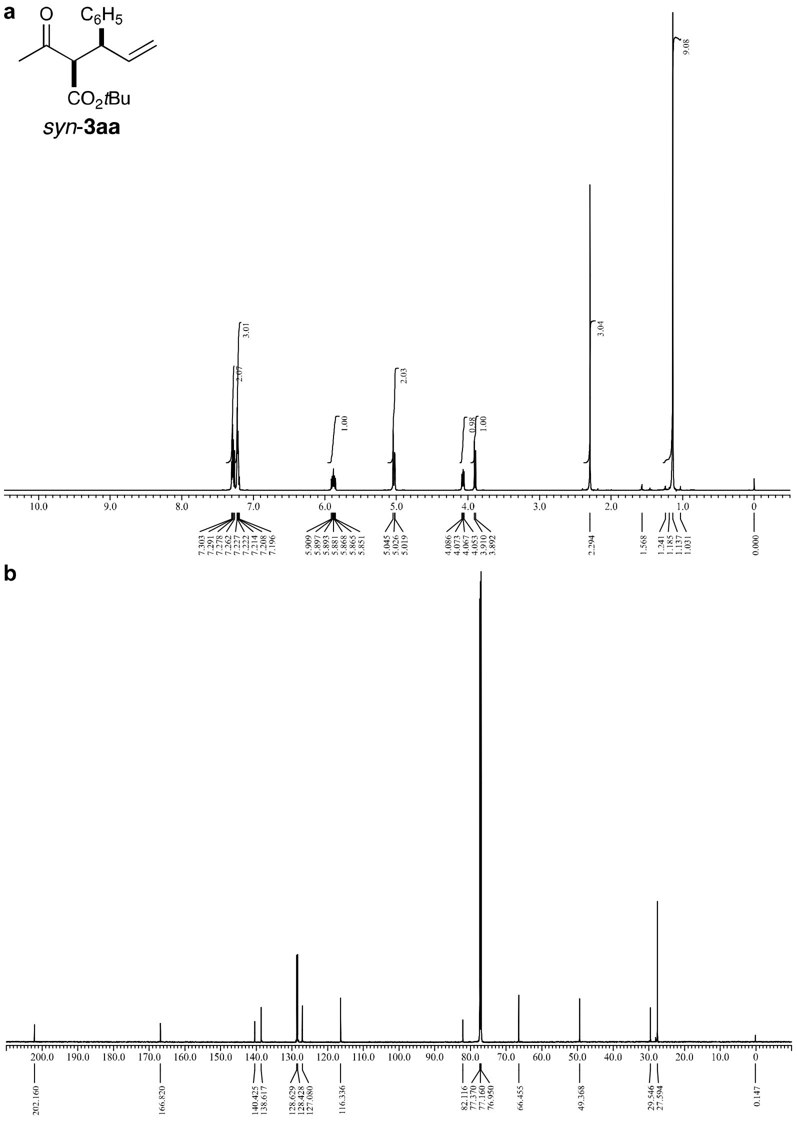

Supplementary Figure 1. ¹H-NMR (**a**) and ¹³C-NMR (**b**) spectra of *tert*-butyl (2*R*,3*S*)-2-acetyl-3-phenylpent-4-enoate (*syn*-**3aa**) in $CDCl_3$ (**Table 2**, entry 2).

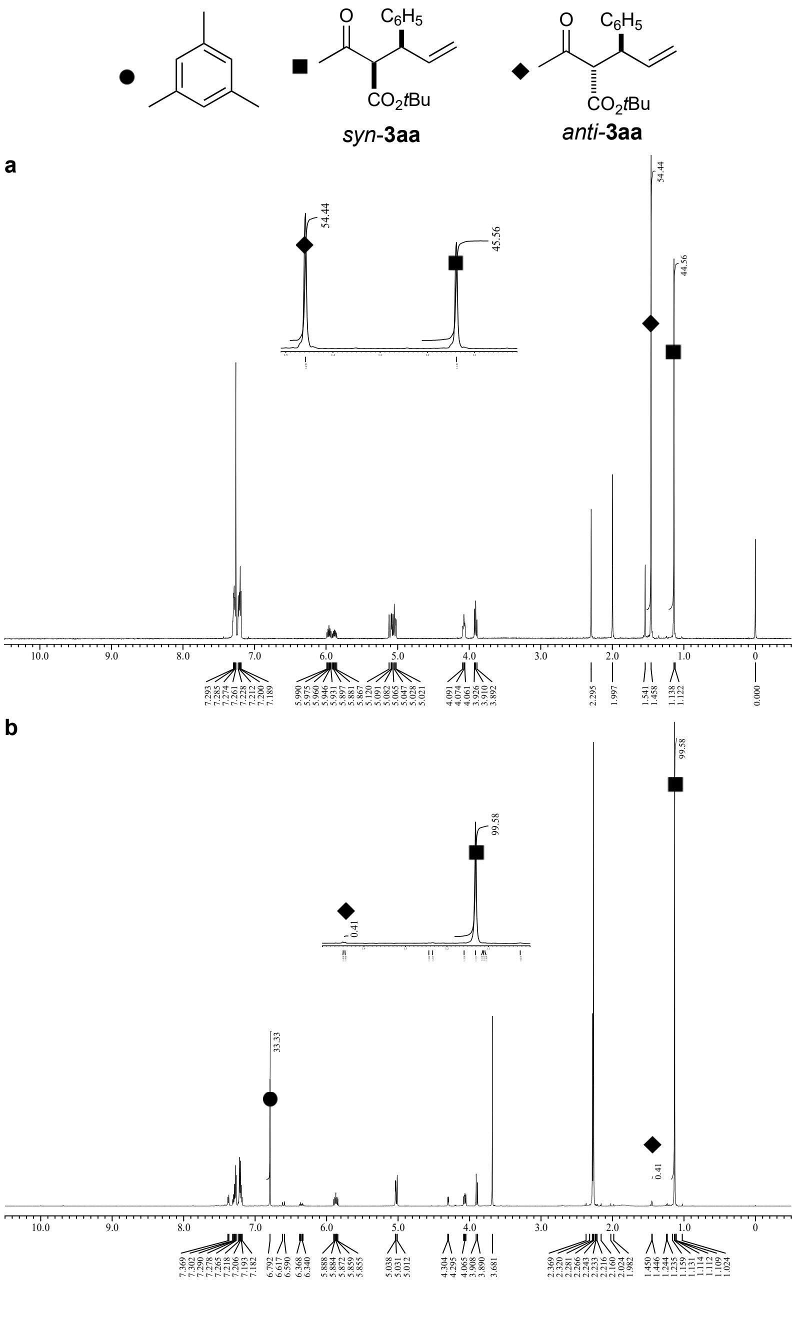

Supplementary Figure 2. ^{1}H-NMR spectrum of diastereomer mixture of **3aa** (**a**) and 0.500-mmol scale reaction mixture containing mesitylene (0.500 mmol) as internal standard (**b**) in $CDCl_3$. Repetition time: 10 sec.

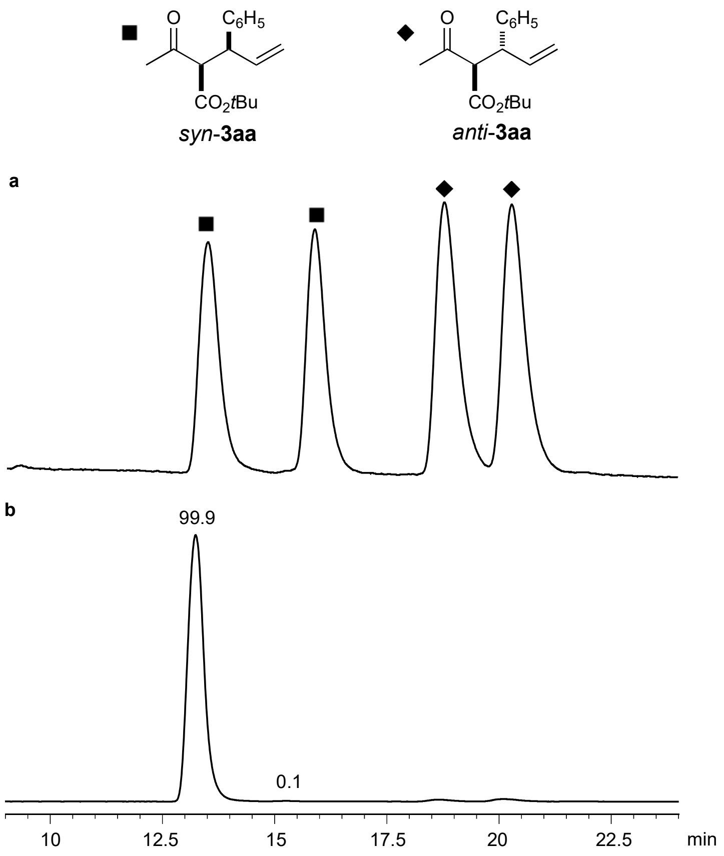

Supplementary Figure 3. HPLC charts of racemic **3aa** (*syn*/*anti* mixture) (**a**) and synthetic (**b**) *tert*-butyl (2*R*,3*S*)-2-acetyl-3-phenylpent-4-enoate (*syn*-**3aa**) (**Table 2**, entry 2). Conditions: column, CHIRALPAK ID-3; eluent, 1.0:99.0 2-PrOH–Hex; flow rate, 0.50 mL/min; detection, 220-nm light.

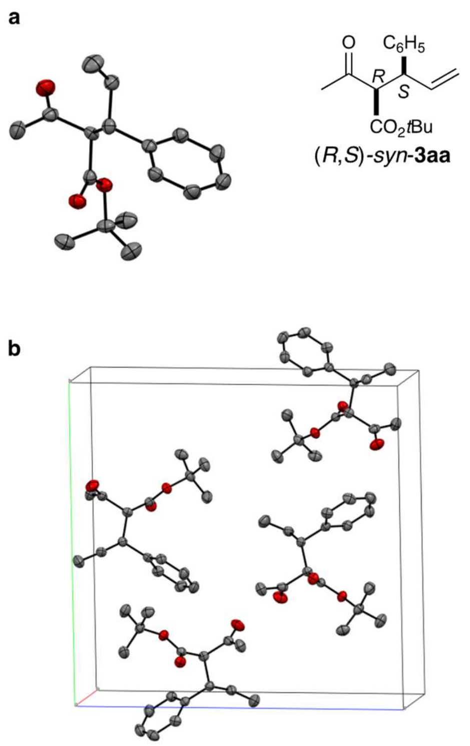

Supplementary Figure 4. ORTEP drawing (**a**) and packing diagram (**b**) of *tert*-butyl (2*R*,3*S*)-2-acetyl-3-phenylpent-4-enoate (*syn*-**3aa**).

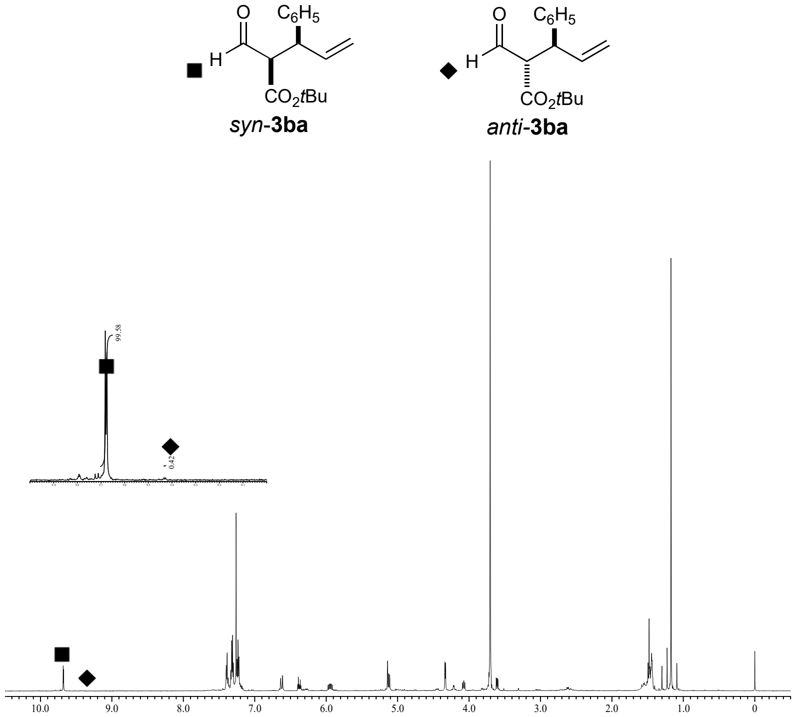

Supplementary Figure 5. ^{1}H-NMR spectrum of 0.500-mmol scale reaction mixture of *syn*-**3ba** in $CDCl_3$. Repetition time: 10 sec.

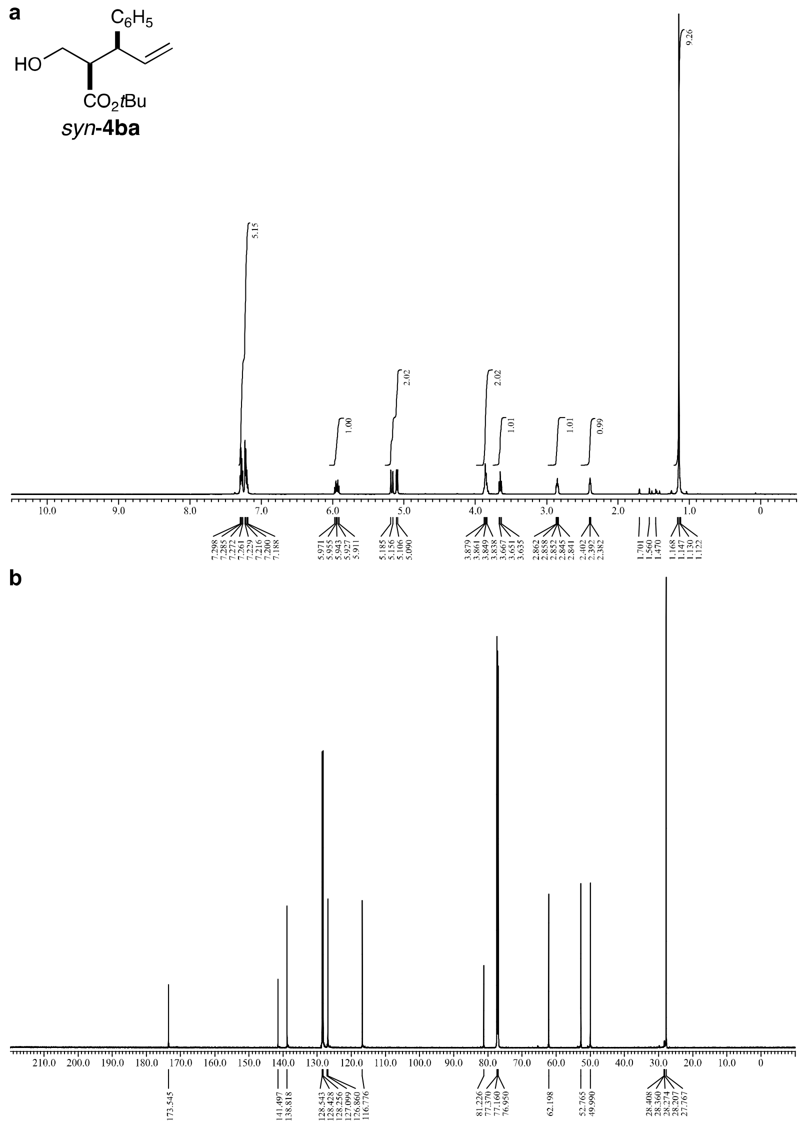

Supplementary Figure 6. ^{1}H-NMR (**a**) and ^{13}C-NMR (**b**) spectra of *tert*-butyl (2*R*,3*S*)-2-(hydroxymethyl)-3-phenylpent-4-enoate (*syn*-**4ba**) in $CDCl_3$ (**Table 2**, entry 1).

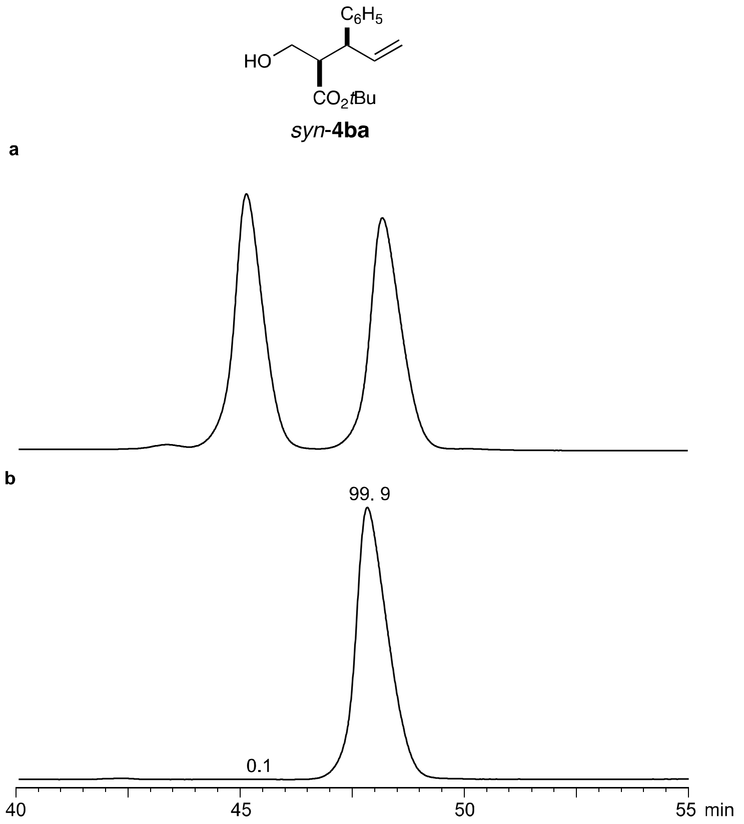

Supplementary Figure 7. HPLC charts of racemic (**a**) and synthetic (**b**) *tert*-butyl (2*S*,3*R*)-2-(hydroxymethyl)-3-phenylpent-4-enoate (*syn*-**4ba**) (**Table 2**, entry 1). Conditions: column, IA and IA-3; eluent, 1.0:99.0 2-PrOH–Hex; flow rate, 0.50 mL/min; detection, 220-nm light.

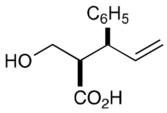

(*R*,*S*)-*syn*-**11ba**

a

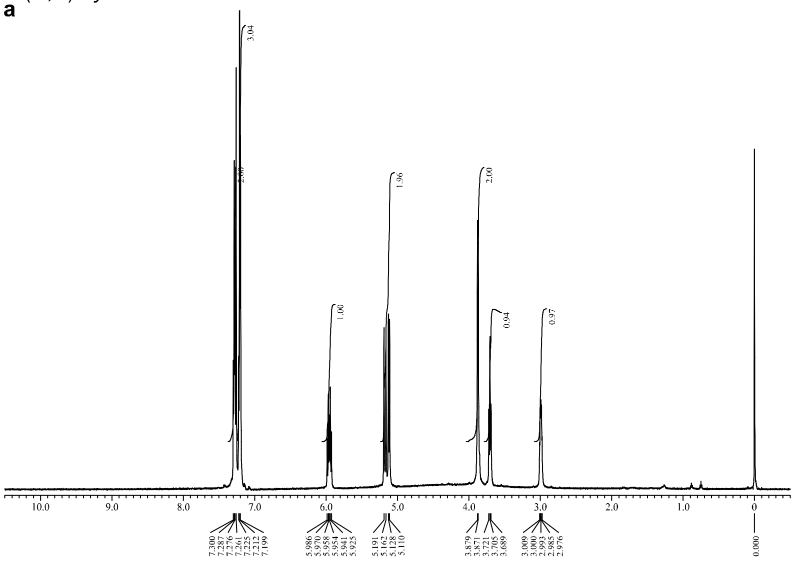

b

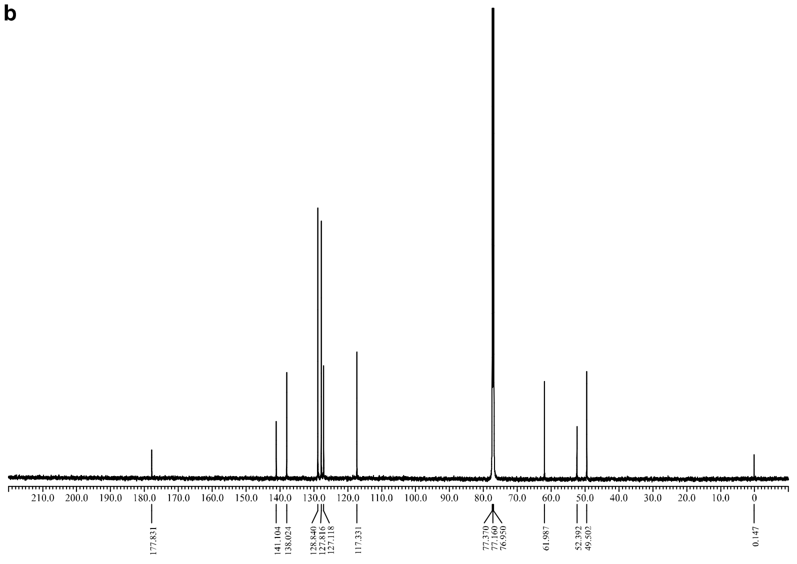

Supplementary Figure 8. ^{1}H-NMR (**a**) and ^{13}C-NMR (**b**) spectra of (2*R*,3*S*)-2-(hydroxymethyl)-3-phenylpent-4-enoic acid (*syn*-**11ba**) in $CDCl_3$.

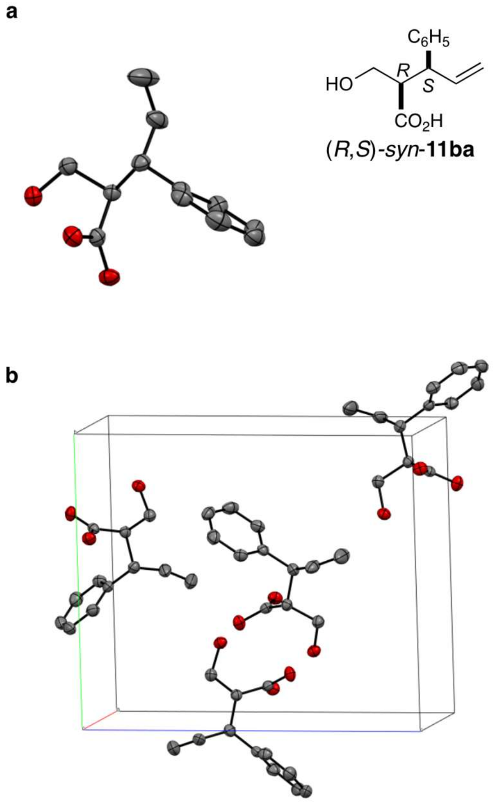

Supplementary Figure 9. ORTEP drawing (**a**) and packing diagram (**b**) of (2*R*,3*S*)-2-(hydroxymethyl)-3-phenylpent-4-enoic acid (*syn*-**11ba**).

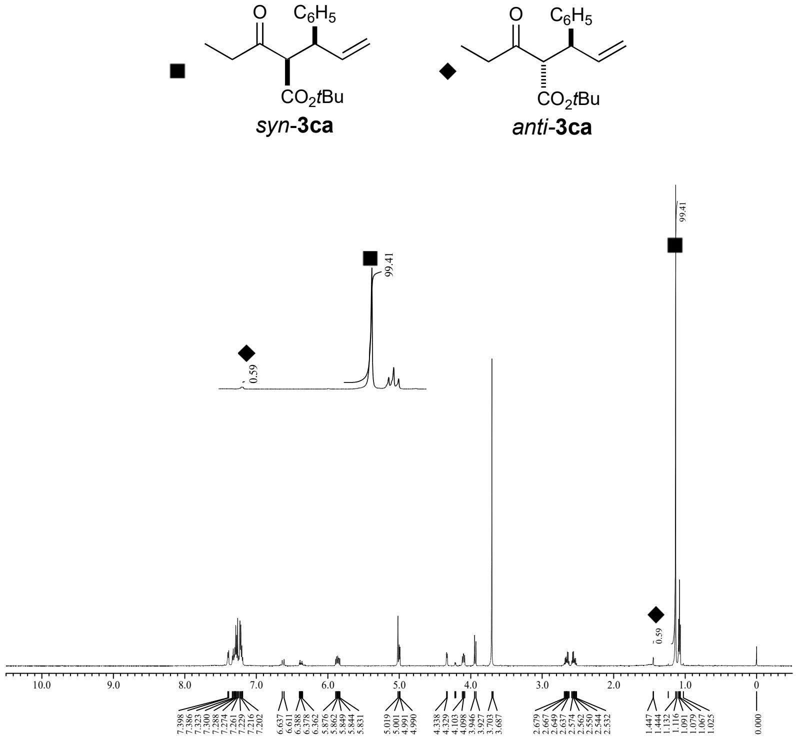

Supplementary Figure 10. ^{1}H-NMR spectrum of 0.500-mmol scale reaction mixture for synthesis of *syn*-**3ca** in $CDCl_3$. Repetition time: 10 sec.

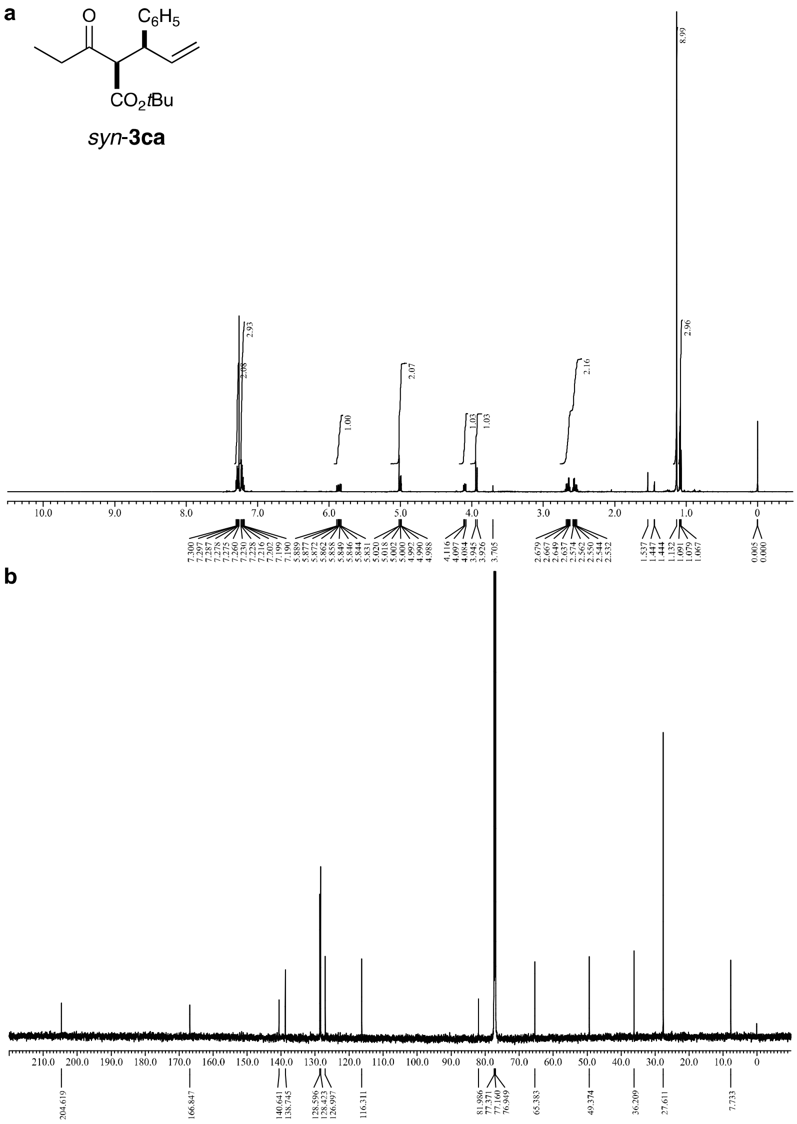

Supplementary Figure 11. ^{1}H-NMR (**a**) and ^{13}C-NMR (**b**) spectra of *tert*-butyl (2*R*,3*S*)-3-phenyl-2-propionylpent-4-enoate (*syn*-**3ca**) in $CDCl_3$ (**Table 2**, entry 3).

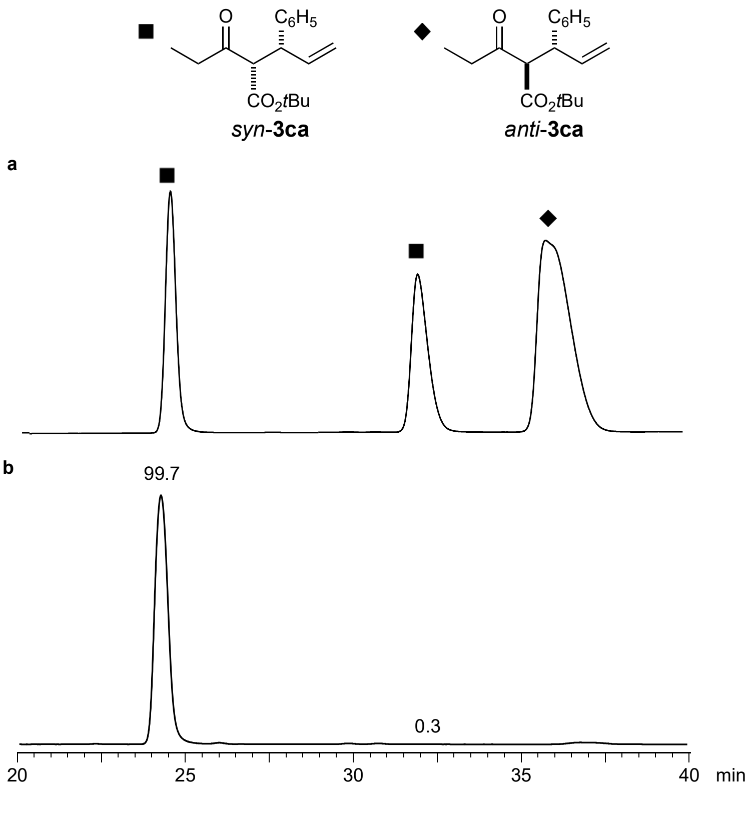

Supplementary Figure 12. HPLC charts of racemic **3ca** (*syn*/*anti* mixture) (**a**) and synthetic (**b**) *tert*-butyl (2*R*,3*S*)-3-phenyl-2-propionylpent-4-enoate (*syn*-**3ca**) (**Table 2**, entry 3). Conditions: column, CHIRALPAK IG; eluent, 0.5:99.5 2-PrOH–Hex; flow rate, 0.50 mL/min; detection, 220-nm light.

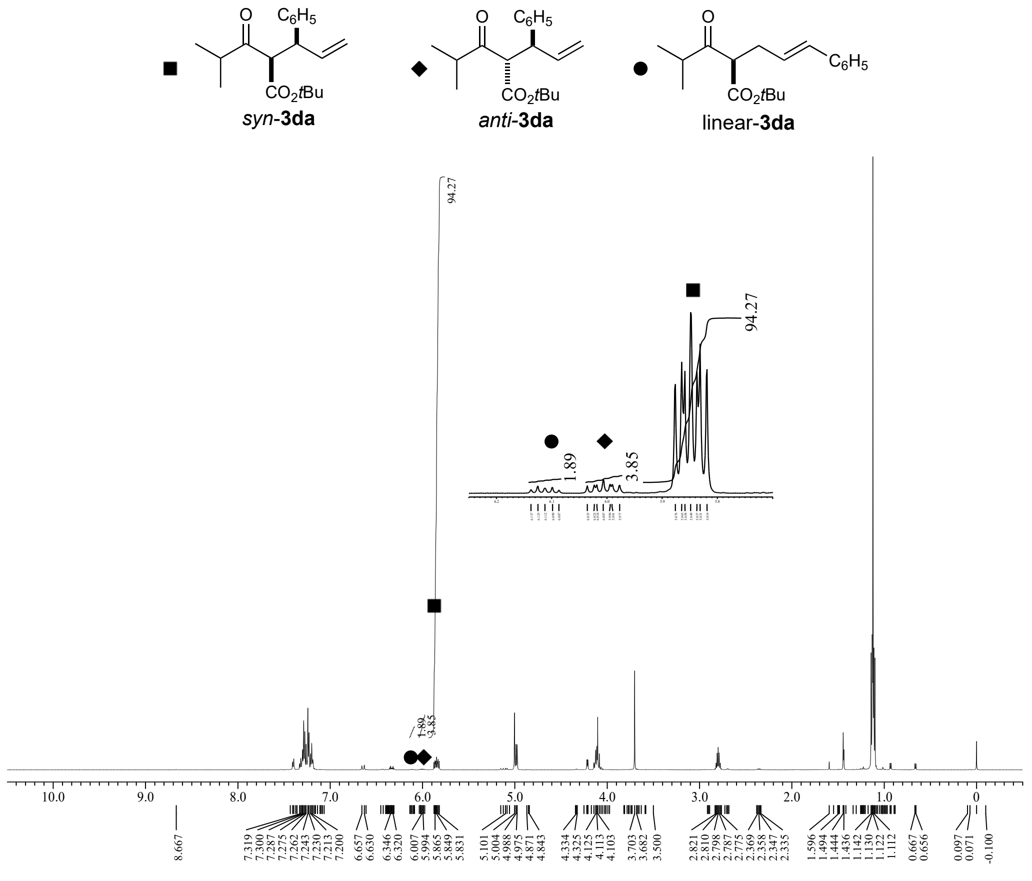

Supplementary Figure 13. ^{1}H-NMR spectrum of 0.500-mmol scale reaction mixture for synthesis of *syn*-**3da** in $CDCl_3$. Repetition time: 10 sec.

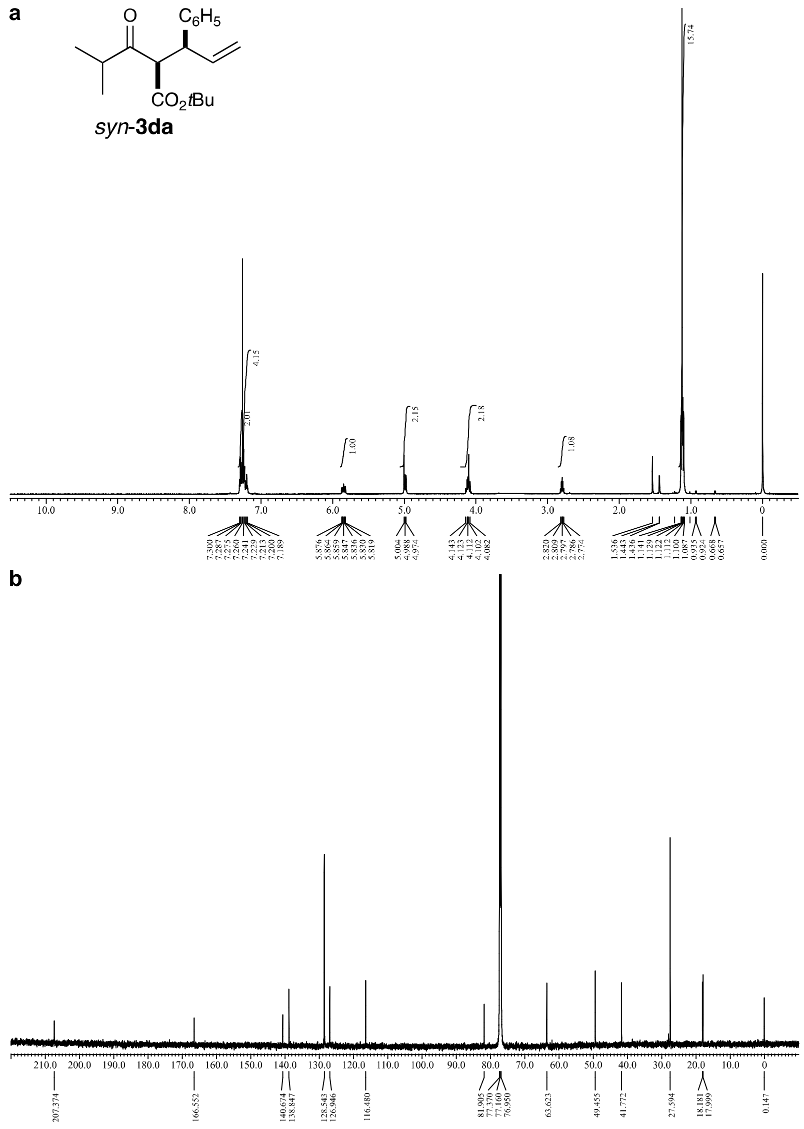

Supplementary Figure 14. ^{1}H-NMR (**a**) and ^{13}C-NMR (**b**) spectra of *tert*-butyl (2*R*,3*S*)-2-isobutyryl-3-phenylpent-4-enoate (*syn*-**3da**) in $CDCl_3$ (**Table 2**, entry 4).

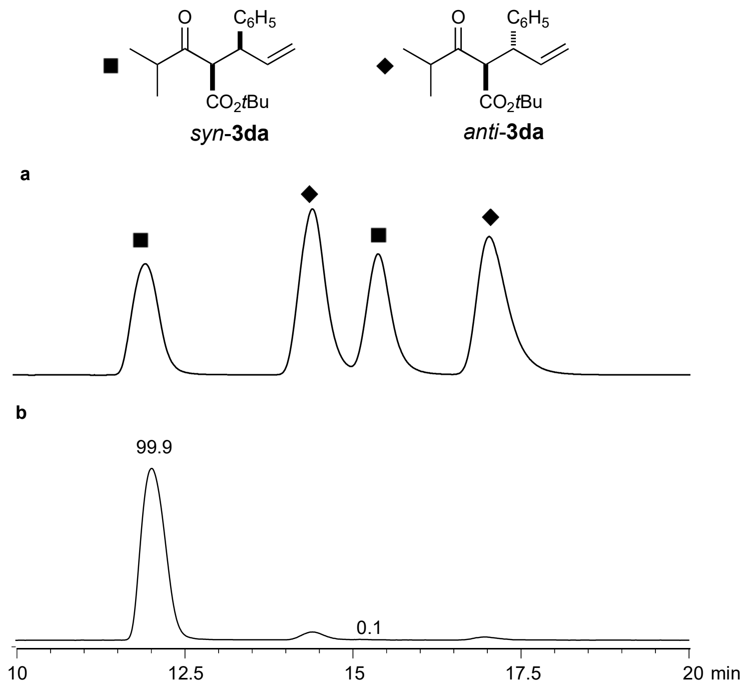

Supplementary Figure 15. HPLC charts of racemic **3da** (*syn*/*anti* mixture) (**a**) and synthetic (**b**) *tert*-butyl (2*R*,3*S*)-2-isobutyryl-3-phenylpent-4-enoate (*syn*-**3da**) (**Table 2**, entry 4). Conditions: column, IA-3; eluent, 1.0:99.0 2-PrOH–Hex; flow rate, 0.50 mL/min; detection, 220-nm light.

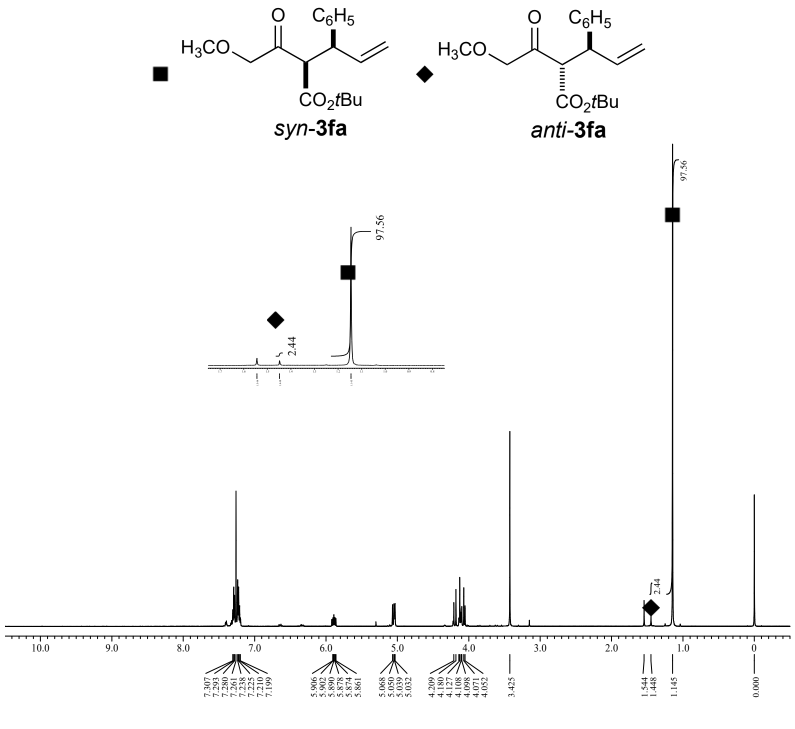

Supplementary Figure 16. ^{1}H-NMR spectrum of 0.500-mmol scale reaction mixture for synthesis of *syn*-**3fa** in $CDCl_3$. Repetition time: 10 sec.

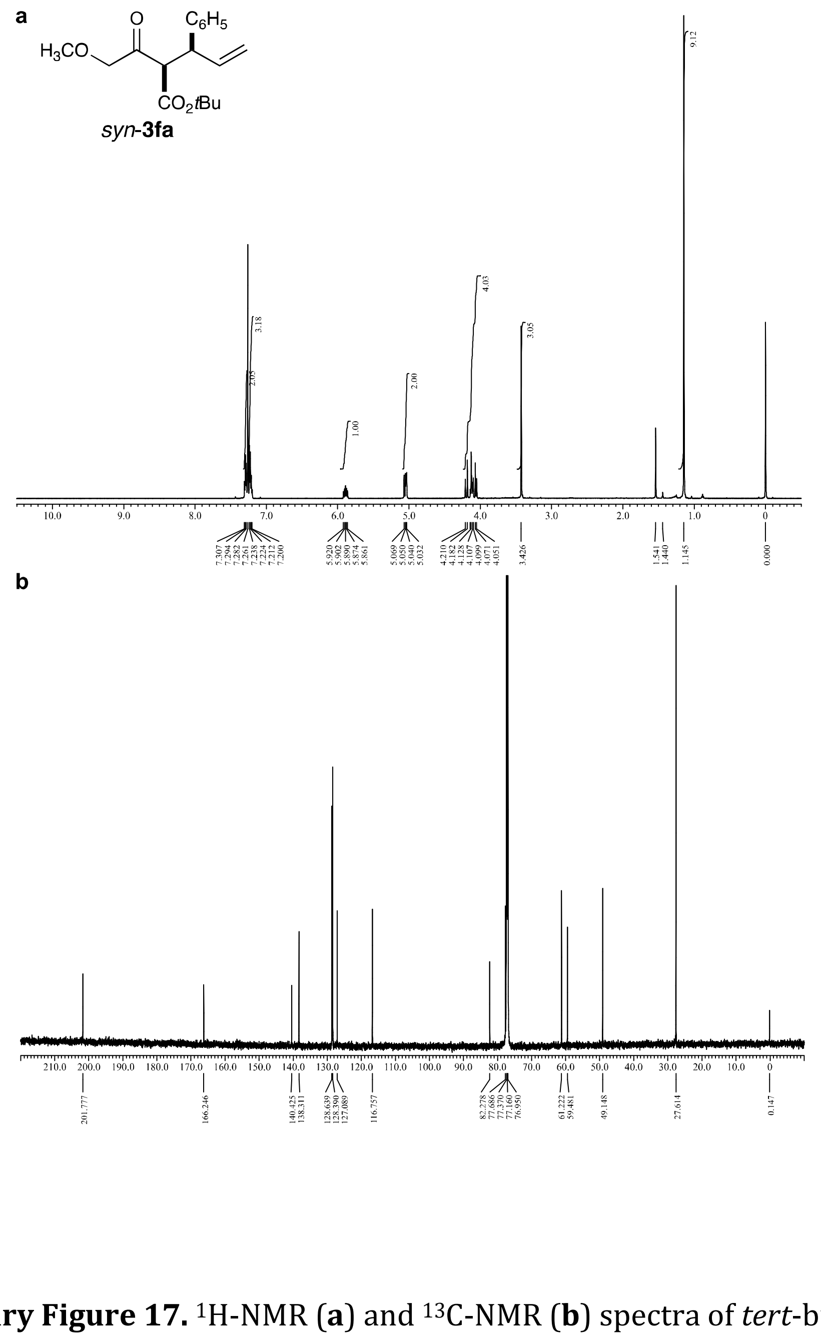

Supplementary Figure 17. ^{1}H-NMR (**a**) and ^{13}C-NMR (**b**) spectra of *tert*-butyl (2*R*,3*S*)-2-(2-methoxyacetyl)-3-phenylpent-4-enoate (*syn*-**3fa**) in $CDCl_3$ (**Table 2**, entry 6).

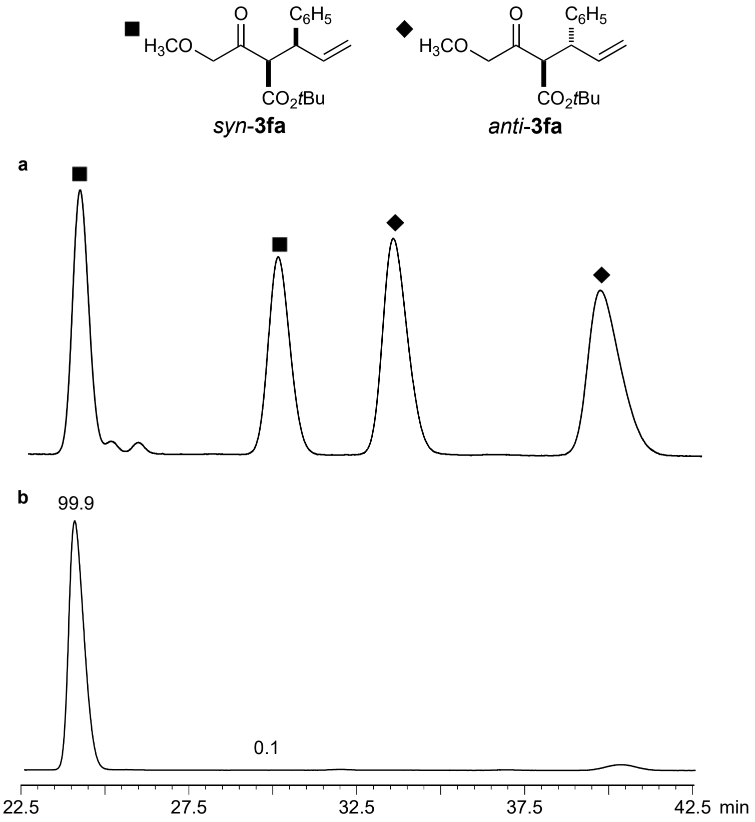

Supplementary Figure 18. HPLC charts of racemic **3fa** (*syn*/*anti* mixture) (**a**) and synthetic (**b**) *tert*-butyl (2*R*,3*S*)-2-(2-methoxyacetyl)-3-phenylpent-4-enoate (*syn*-**3fa**) (**Table 2**, entry 6). Conditions: column, CHIRALPAK IG; eluent, 1.0:99.0 2-PrOH–Hex; flow rate, 1.00 mL/min; detection, 220-nm light.

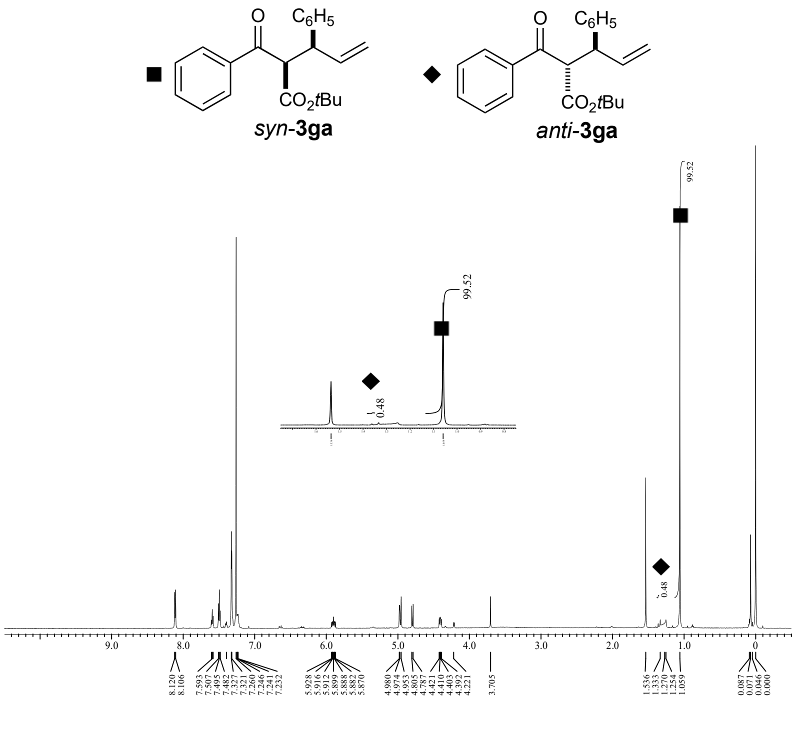

Supplementary Figure 19. ^{1}H-NMR spectrum of 0.500-mmol scale reaction mixture for synthesis of *syn*-**3ga** in $CDCl_3$. Repetition time: 10 sec.

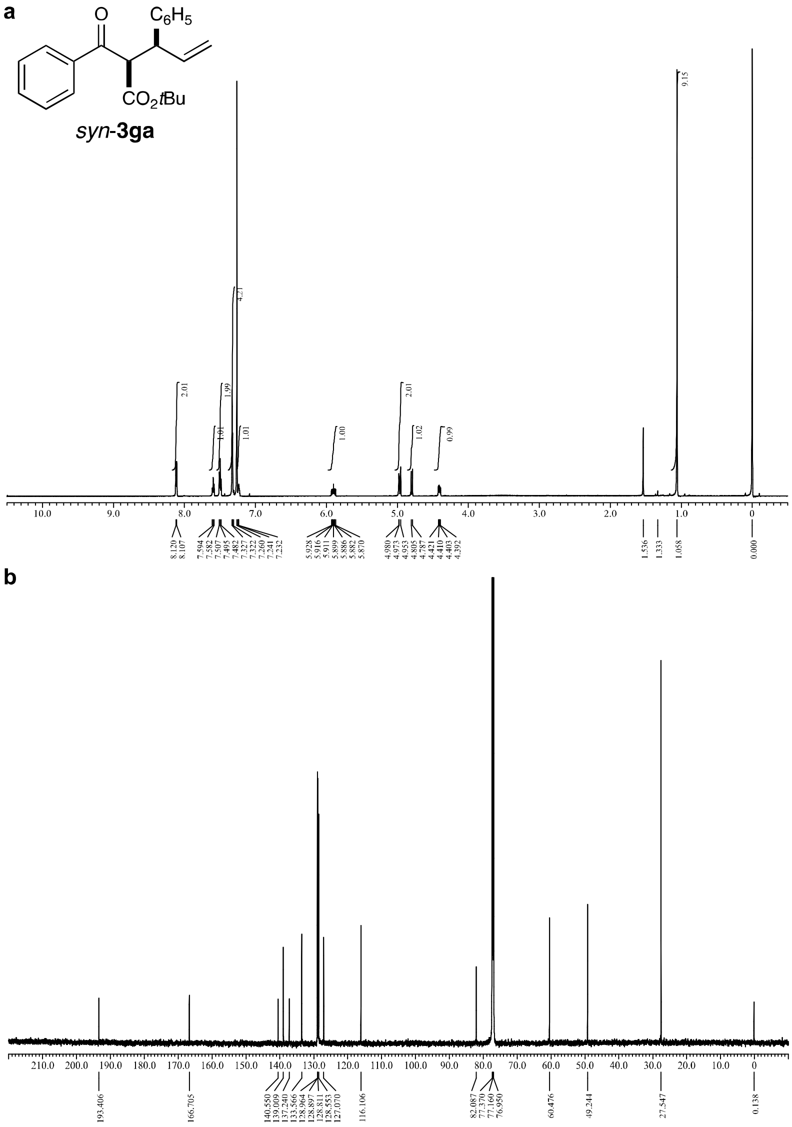

Supplementary Figure 20. ^{1}H-NMR (**a**) and ^{13}C-NMR (**b**) spectra of *tert*-butyl (2*R*,3*S*)-2-benzoyl-3-phenylpent-4-enoate (*syn*-**3ga**) in $CDCl_3$ (**Table 2**, entry 7).

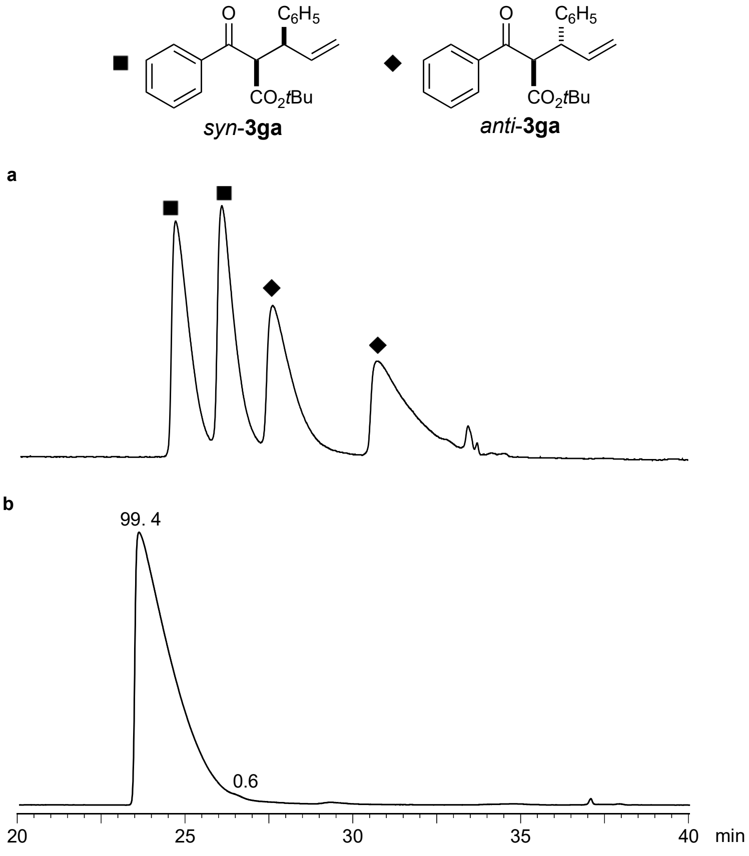

Supplementary Figure 21. HPLC charts of racemic **3ga** (*syn*/*anti* mixture) (**a**) and synthetic (**b**) *tert*-butyl (2*R*,3*S*)-2-benzoyl-3-phenylpent-4-enoate (*syn*-**3ga**) (**Table 2**, entry 7). Conditions: column, CHIRALPAK IB and CHIRALPAK IB-3; eluent, 0.2:99.8 2-PrOH–Hex; flow rate, 1.00 mL/min; detection, 220-nm light.

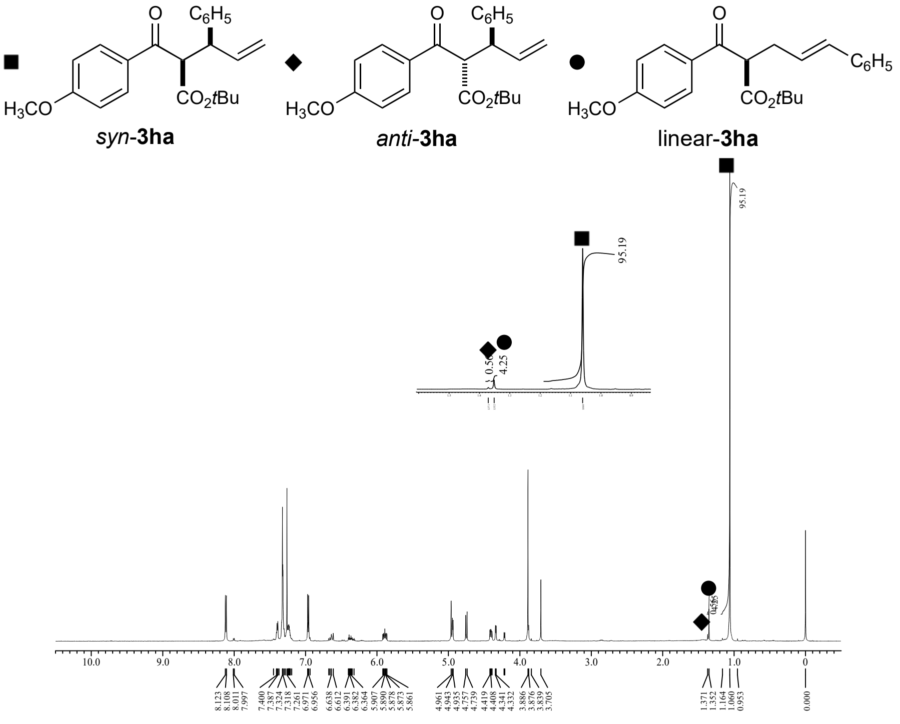

Supplementary Figure 22. ^{1}H-NMR spectrum of 0.500-mmol scale reaction mixture for synthesis of *syn*-**3ha** in $CDCl_3$. Repetition time: 10 sec.

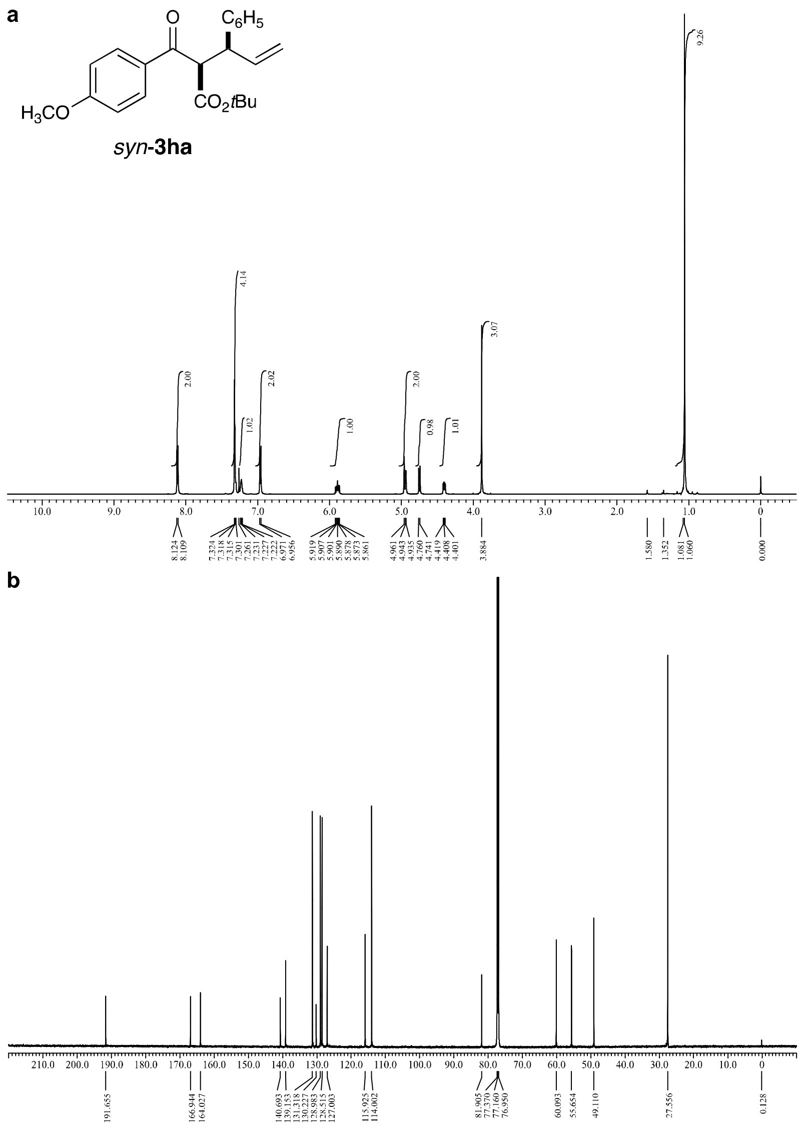

Supplementary Figure 23. ^{1}H-NMR (**a**) and ^{13}C-NMR (**b**) spectra of *tert*-butyl (2*R*,3*S*)-2-(4-methoxybenzoyl)-3-phenylpent-4-enoate (*syn*-**3ha**) in $CDCl_3$ (**Table 2**, entry 8).

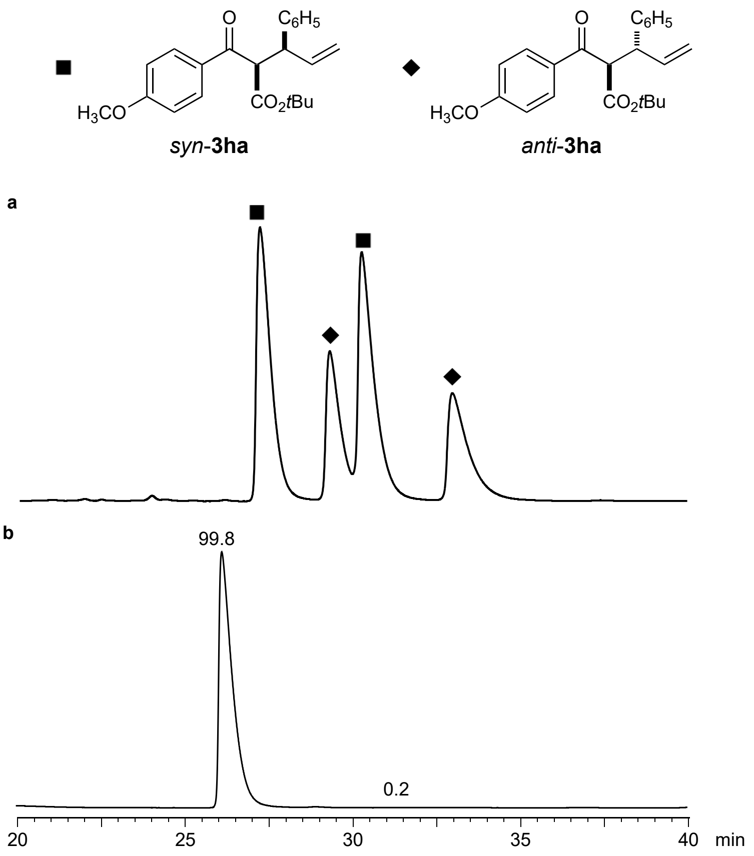

Supplementary Figure 24. HPLC charts of racemic **3ha** (*syn*/*anti* mixture) (**a**) and synthetic (**b**) *tert*-butyl (2*R*,3*S*)-2-(4-methoxybenzoyl)-3-phenylpent-4-enoate (*syn*-**3ha**) (**Table 2**, entry 8). Conditions: column, CHIRALPAK IB and CHIRALPAK IB-3; eluent, 0.5:99.5 2-PrOH–Hex; flow rate, 1.00 mL/min; detection, 220-nm light.

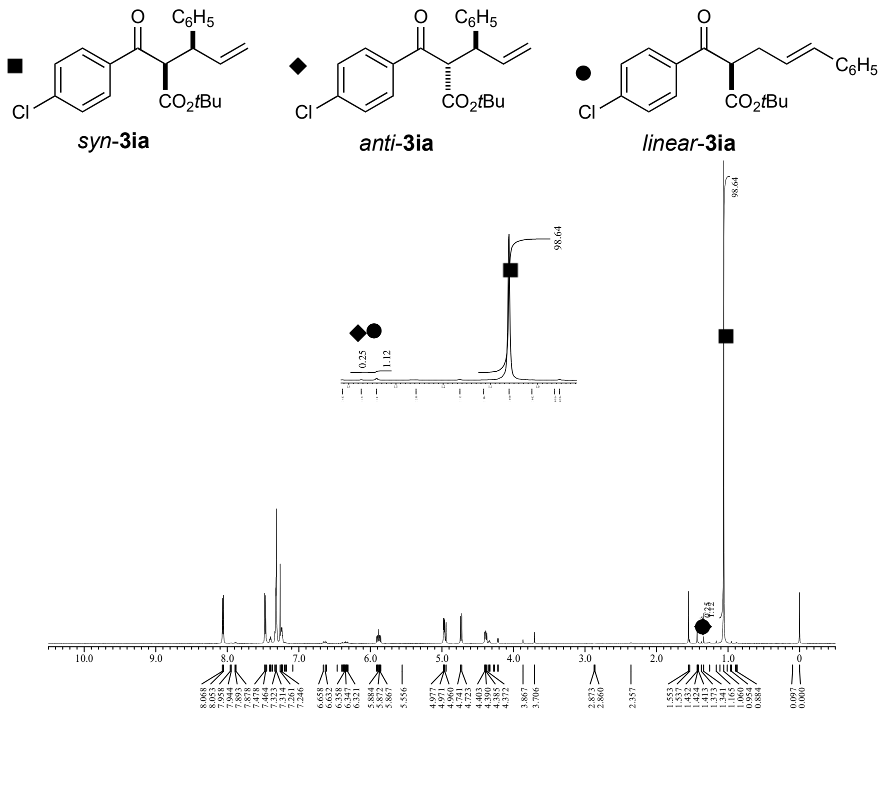

Supplementary Figure 25. ^{1}H-NMR spectrum of 0.500-mmol scale reaction mixture for synthesis of *syn*-**3ia** in $CDCl_3$. Repetition time: 10 sec.

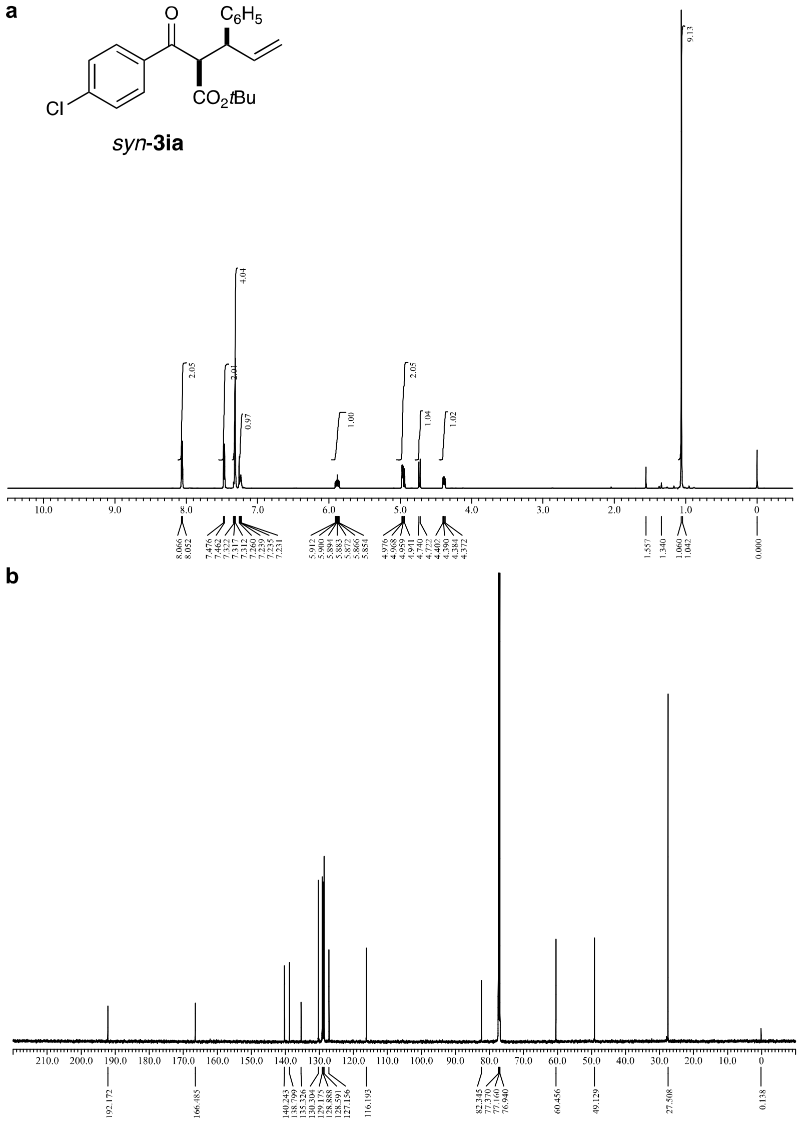

Supplementary Figure 26. ^{1}H-NMR (**a**) and ^{13}C-NMR (**b**) spectra of *tert*-butyl (2*R*,3*S*)-2-(4-chlorobenzoyl)-3-phenylpent-4-enoate (*syn*-**3ia**) in $CDCl_3$ (**Table 2**, entry 9).

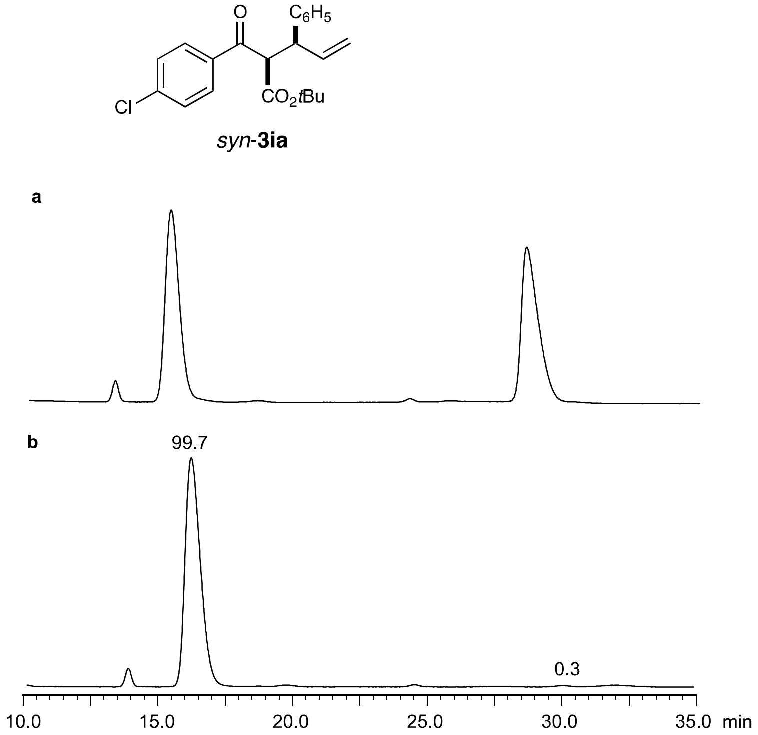

Supplementary Figure 27. HPLC charts of racemic (**a**) and synthetic (**b**) *tert*-butyl (2*R*,3*S*)-2-(4-chlorobenzoyl)-3-phenylpent-4-enoate (*syn*-**3ia**) (**Table 2**, entry 9). Conditions: column, CHIRALPAK IG; eluent, 1.0:99.0 2-PrOH–Hex; flow rate, 1.00 mL/min; detection, 220-nm light.

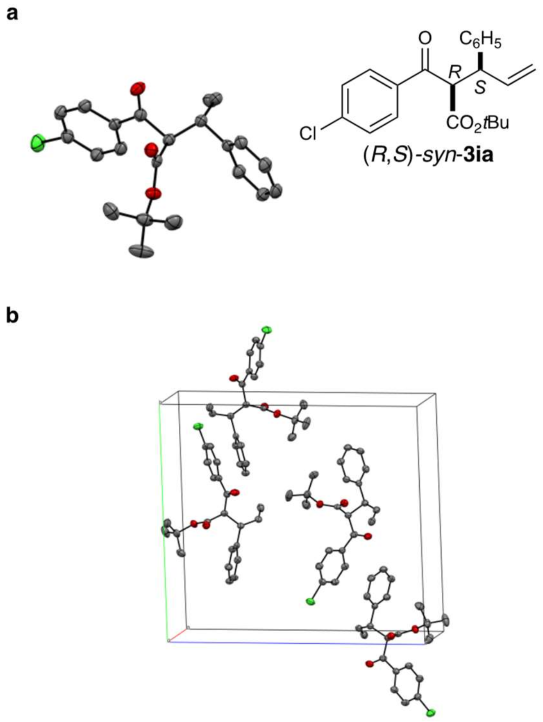

Supplementary Figure 28. ORTEP drawing (**a**) and packing diagram (**b**) of *tert*-butyl (2*R*,3*S*)-2-(4-chlorobenzoyl)-3-phenylpent-4-enoate (*syn*-**3ia**).

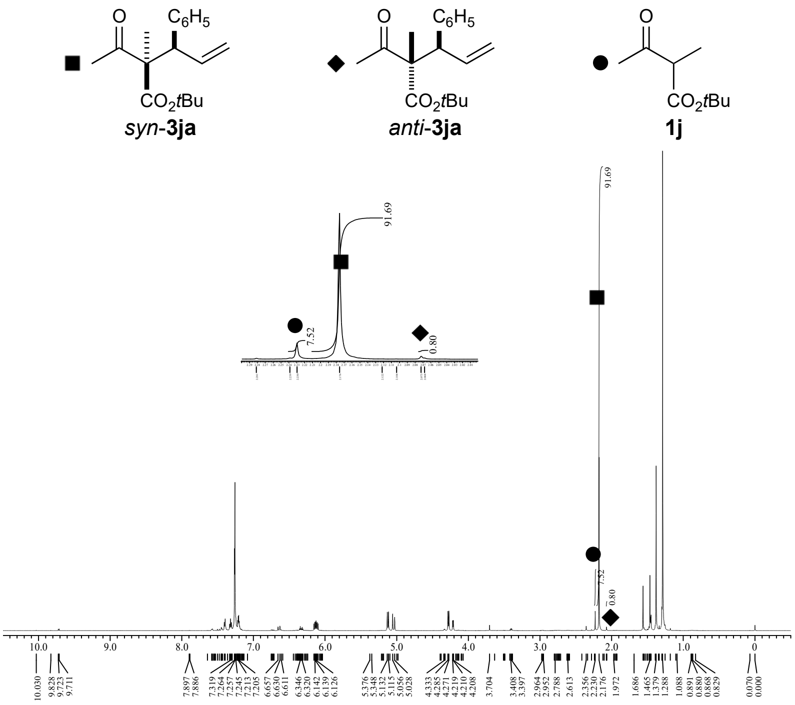

Supplementary Figure 29. ^{1}H-NMR spectrum of 0.500-mmol scale reaction mixture for synthesis of *syn*-**3ja** in $CDCl_3$. Repetition time: 10 sec.

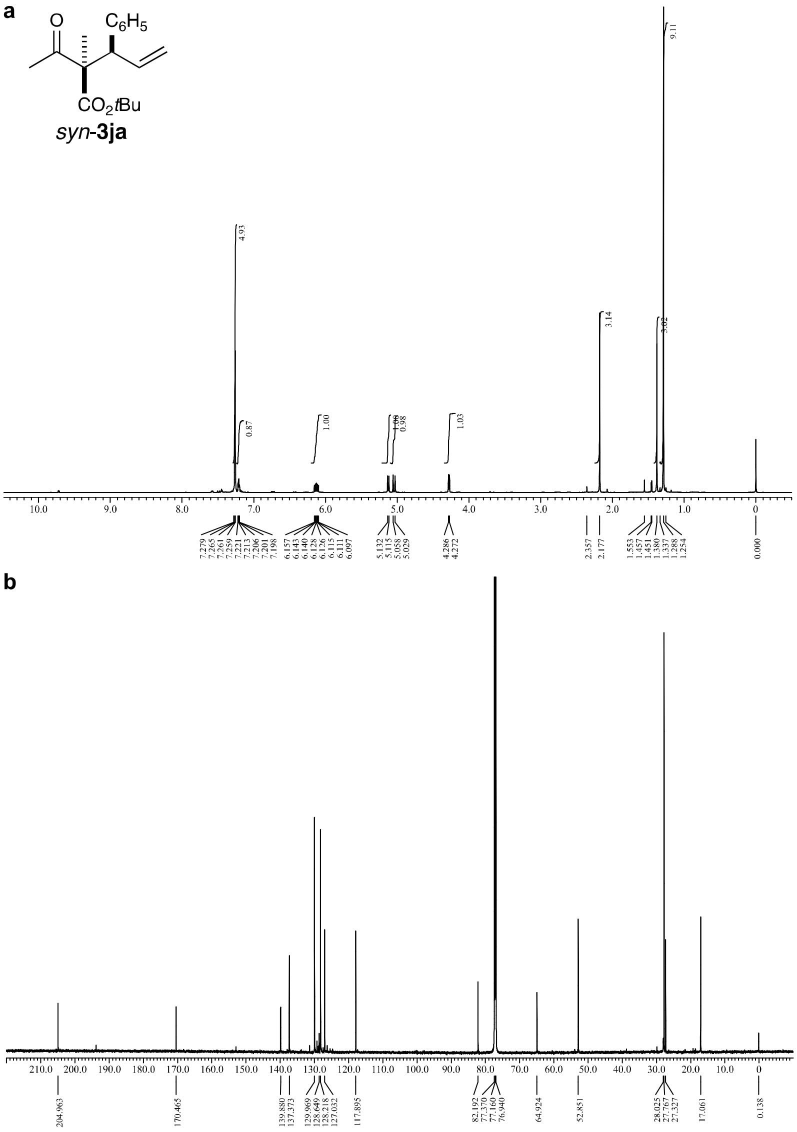

Supplementary Figure 30. ^{1}H-NMR (**a**) and ^{13}C-NMR (**b**) spectra of *tert*-butyl (2*R*,3*R*)-2-acetyl-2-methyl-3-phenylpent-4-enoate (*syn*-**3ja**) in $CDCl_3$ (**Table 2**, entry 10).

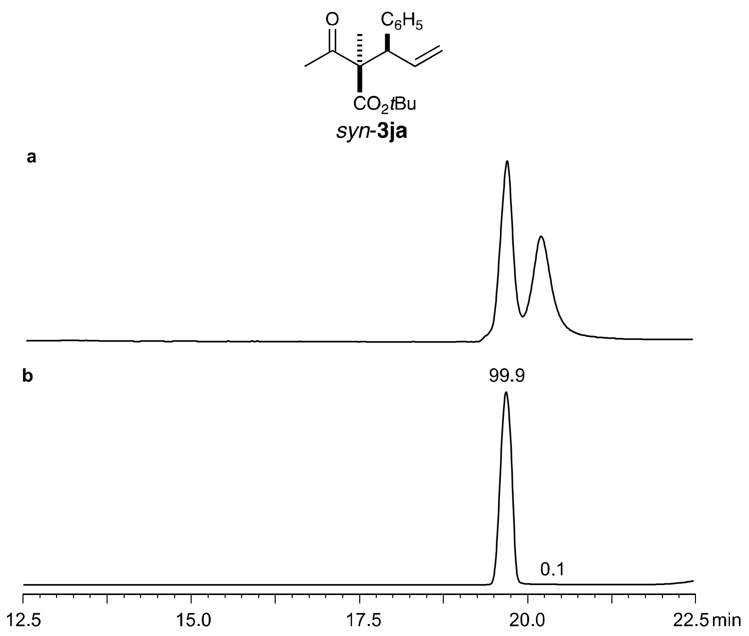

Supplementary Figure 31. HPLC charts of racemic (**a**) and synthetic (**b**) *tert*-butyl (2*R*,3*R*)-2-acetyl-2-methyl-3-phenylpent-4-enoate (*syn*-**3ja**) (**Table 2**, entry 10). Conditions: column, CHIRALPAK IE-3; eluent, 0.2:99.8 2-PrOH–Hex; flow rate, 1.00 mL/min; detection, 220-nm light.

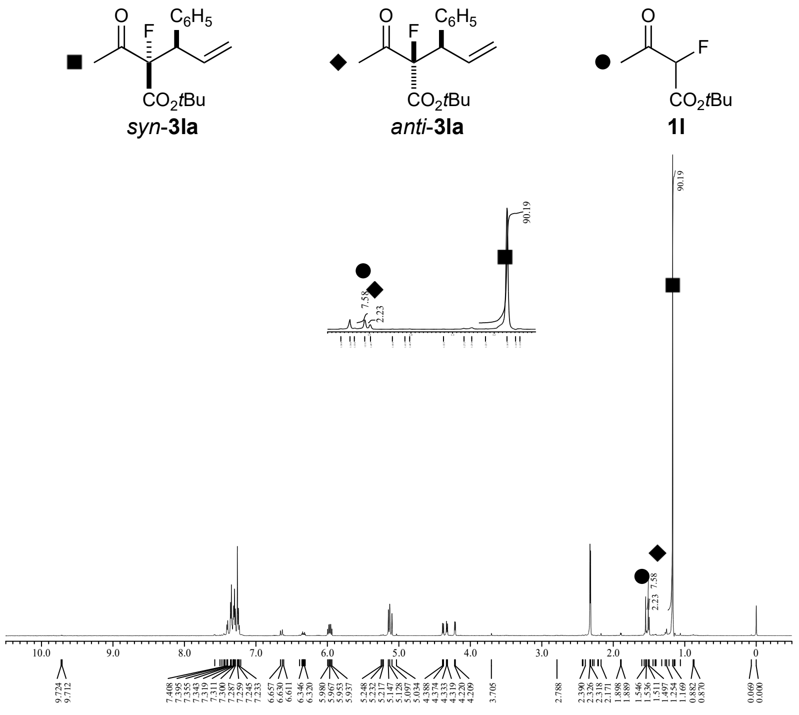

Supplementary Figure 32. ^{1}H-NMR spectrum of 0.500-mmol scale reaction mixture for synthesis of *syn*-**3la** in $CDCl_3$. Repetition time: 10 sec.

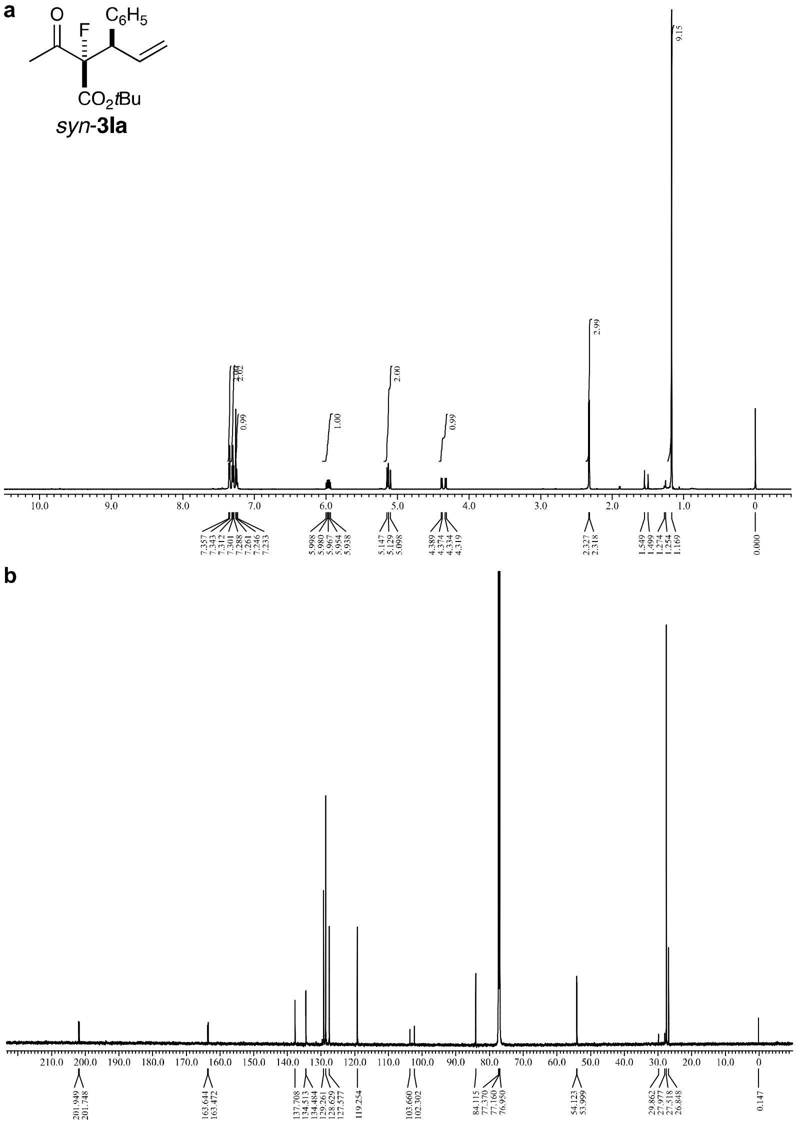

Supplementary Figure 33. ^{1}H-NMR (**a**) and ^{13}C-NMR (**b**) spectra of *tert*-butyl (2*S*,3*R*)-2-acetyl-2-fluoro-3-phenylpent-4-enoate (*syn*-**3la**) in $CDCl_3$ (**Table 2**, entry 12).

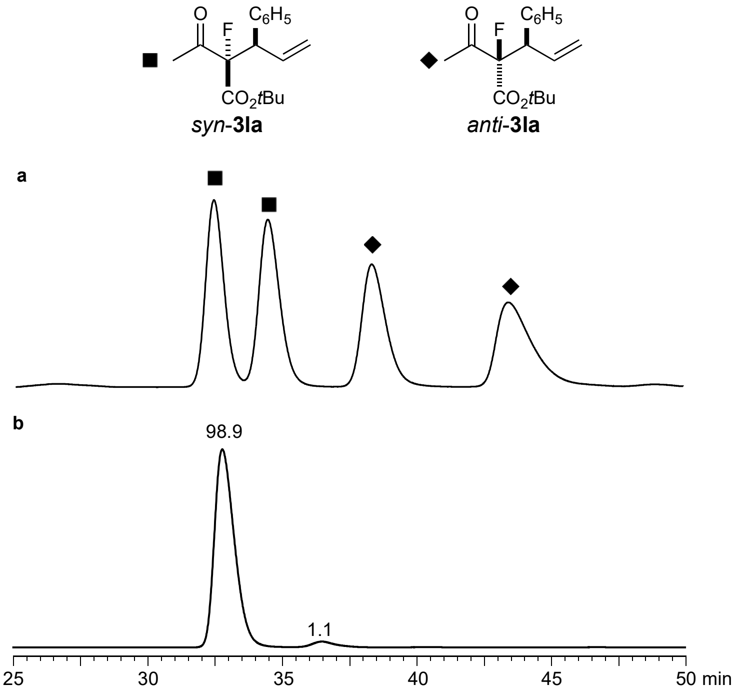

Supplementary Figure 34. HPLC charts of racemic **3la** (*syn*/*anti* mixture) (**a**) and synthetic (**b**) *tert*-butyl (2*S*,3*R*)-2-acetyl-2-fluoro-3-phenylpent-4-enoate (*syn*-**3la**) (**Table 2**, entry 12). Conditions: column, 20.0 mmϕ x 250 mm CHIRALCEL OD-H; eluent, 0.5:99.5 2-PrOH–Hex; flow rate, 3.00 mL/min; detection, 220-nm light.

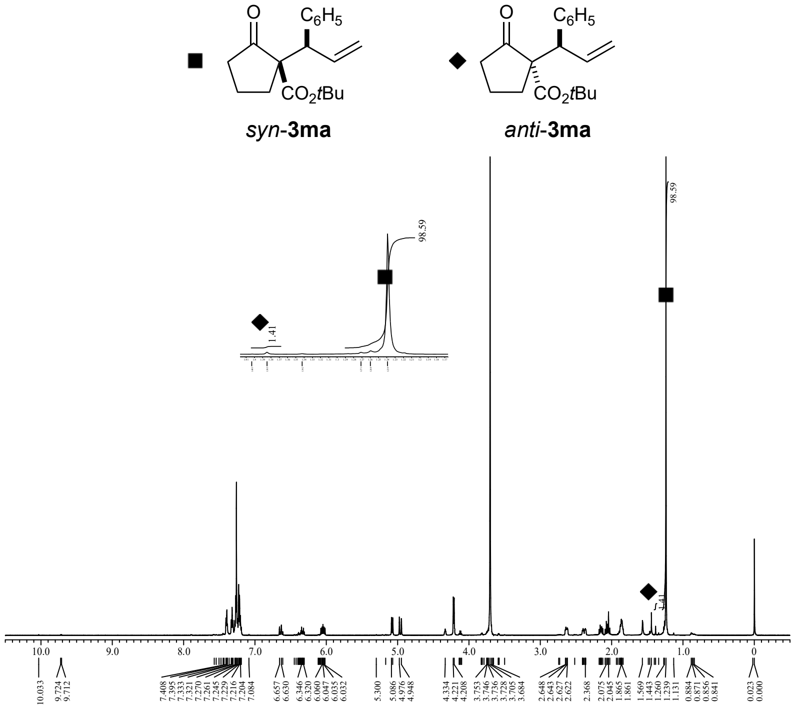

Supplementary Figure 35. ^{1}H-NMR spectrum of 0.500-mmol scale reaction mixture for synthesis of *syn*-**3ma** in $CDCl_3$. Repetition time: 10 sec.

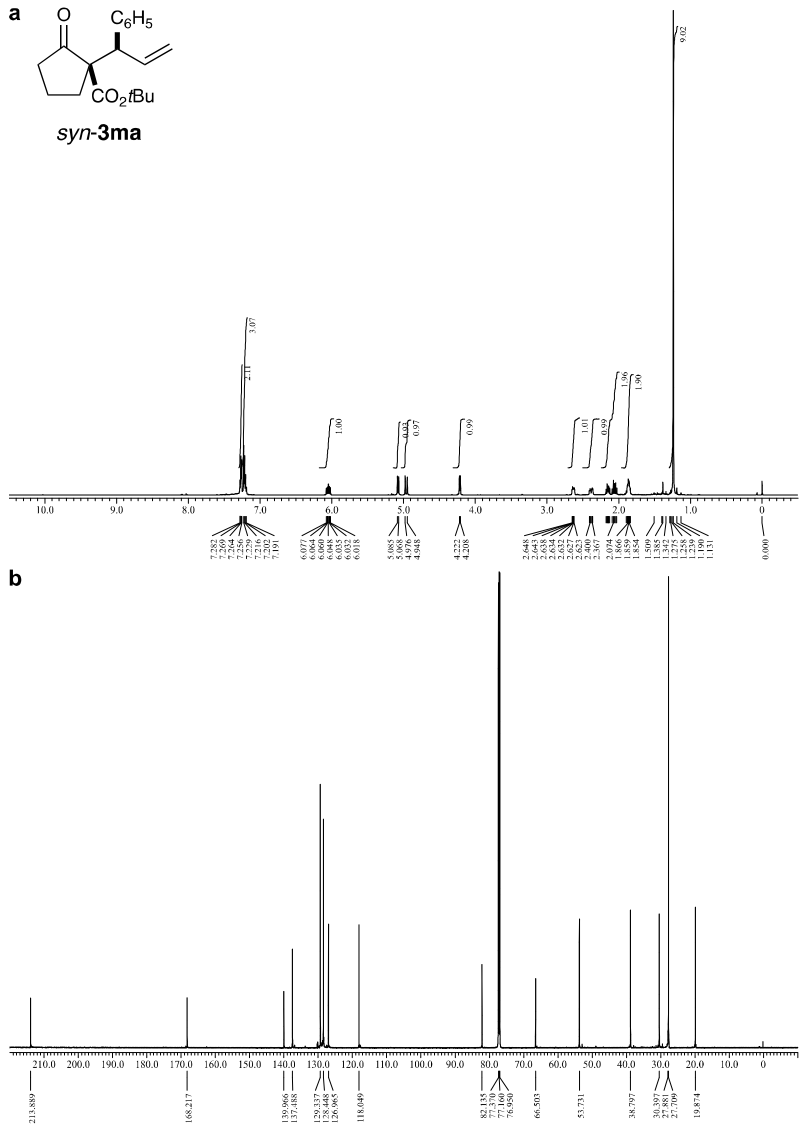

Supplementary Figure 36. ^{1}H-NMR (**a**) and ^{13}C-NMR (**b**) spectra of *tert*-butyl (*S*)-2-oxo-1-((*R*)-1-phenylallyl)cyclopentane-1-carboxylate (*syn*-**3ma**) in $CDCl_3$ (**Table 2**, entry 13).

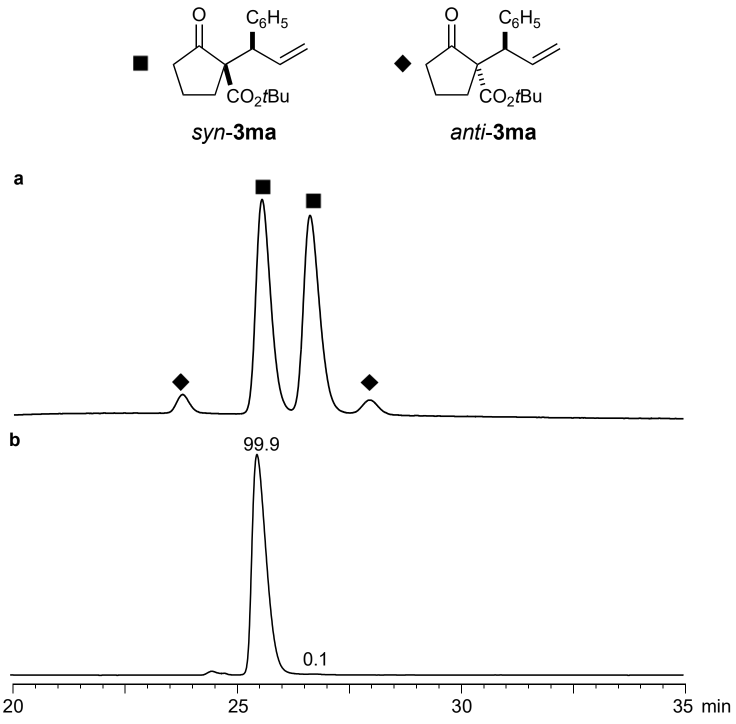

Supplementary Figure 37. HPLC charts of racemic **3ma** (*syn*/*anti* mixture) (**a**) and synthetic (**b**) *tert*-butyl (*S*)-2-oxo-1-((*R*)-1-phenylallyl)cyclopentane-1-carboxylate (*syn*-**3ma**) (**Table 2**, entry 13). Conditions: column, CHIRALPAK IB and CHIRALPAK IB-3; eluent, 0.2:99.8 2-PrOH–Hex; flow rate, 0.50 mL/min; detection, 220-nm light.

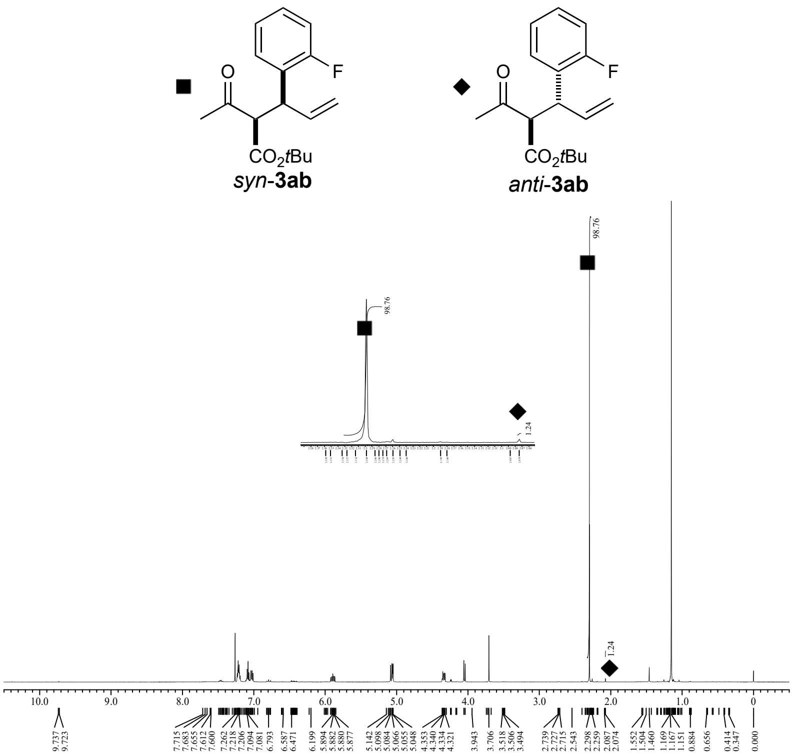

Supplementary Figure 38. ^{1}H-NMR spectrum of 0.500-mmol scale reaction mixture for synthesis of *syn*-**3ab** in $CDCl_3$. Repetition time: 10 sec.

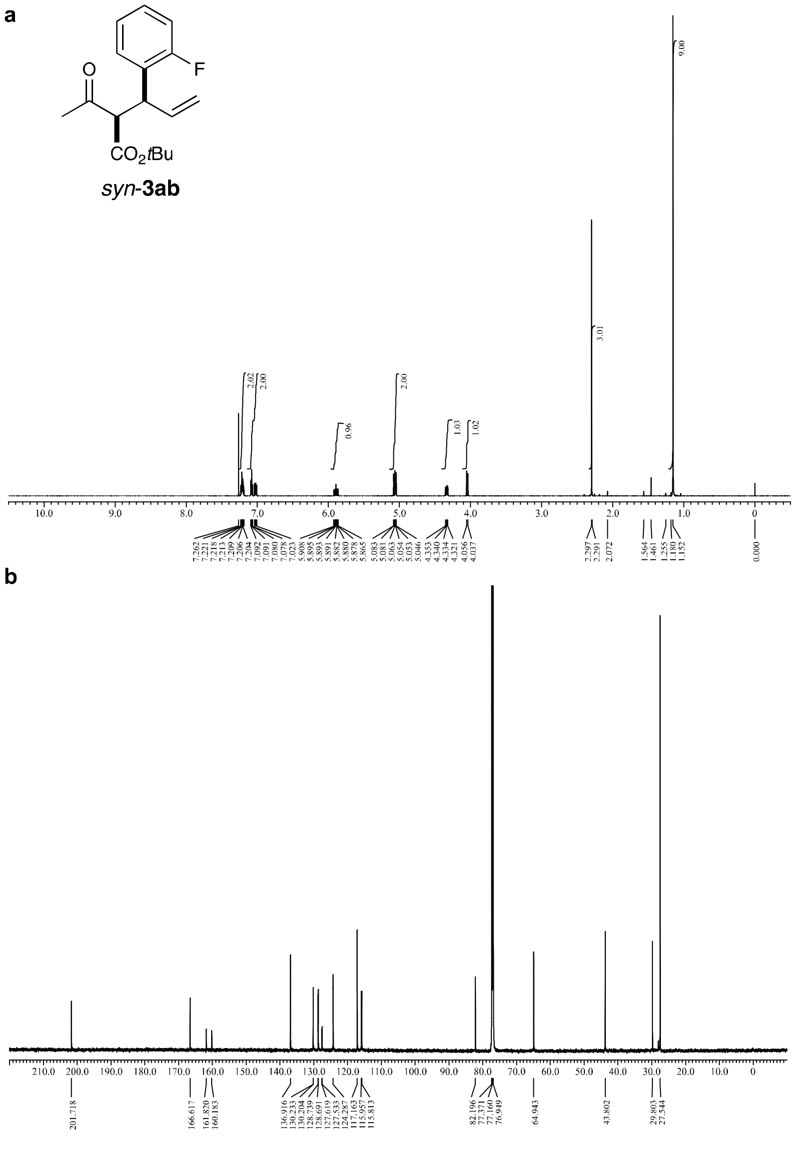

Supplementary Figure 39. ^{1}H-NMR (**a**) and ^{13}C-NMR (**b**) spectra of *tert*-butyl (2*R*,3*S*)-2-acetyl-3-(2-fluorophenyl)pent-4-enoate (*syn*-**3ab**) in $CDCl_3$ (**Table 2**, entry 14).

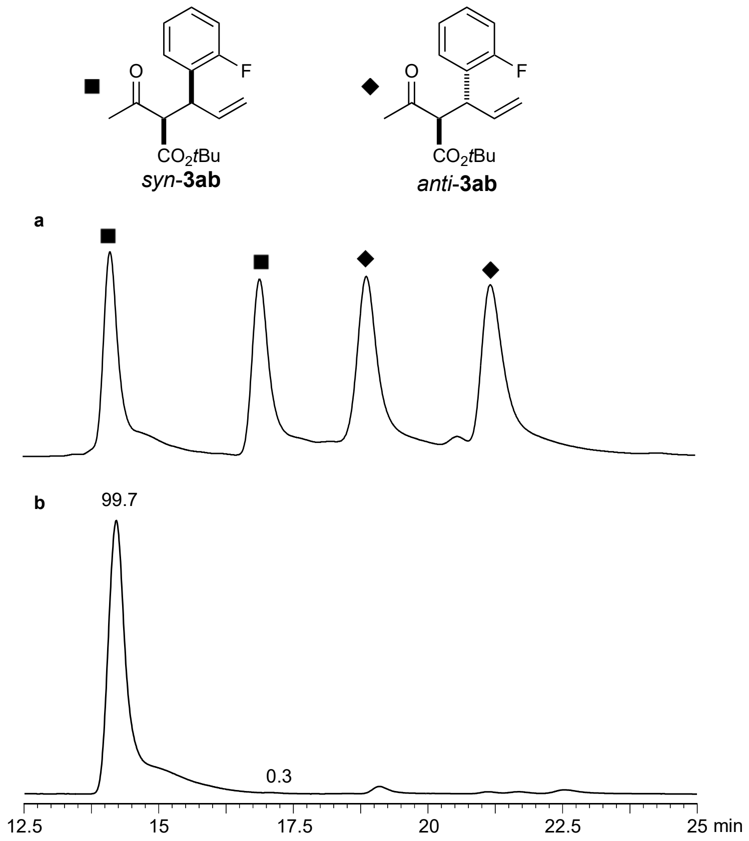

Supplementary Figure 40. HPLC charts of racemic **3ab** (*syn*/*anti* mixture) (**a**) and synthetic (**b**) *tert*-butyl (2*R*,3*S*)-2-acetyl-3-(2-fluorophenyl)pent-4-enoate (*syn*-**3ab**) (**Table 2**, entry 14). Conditions: column, IA-3; eluent, 1.0:99.0 2-PrOH–Hex; flow rate, 0.50 mL/min; detection, 220-nm light.

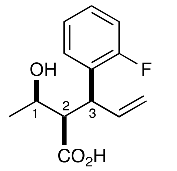

(1*R*,2*R*,3*S*)-*syn*$_{1,2}$*syn*$_{2,3}$-**11ab**

a

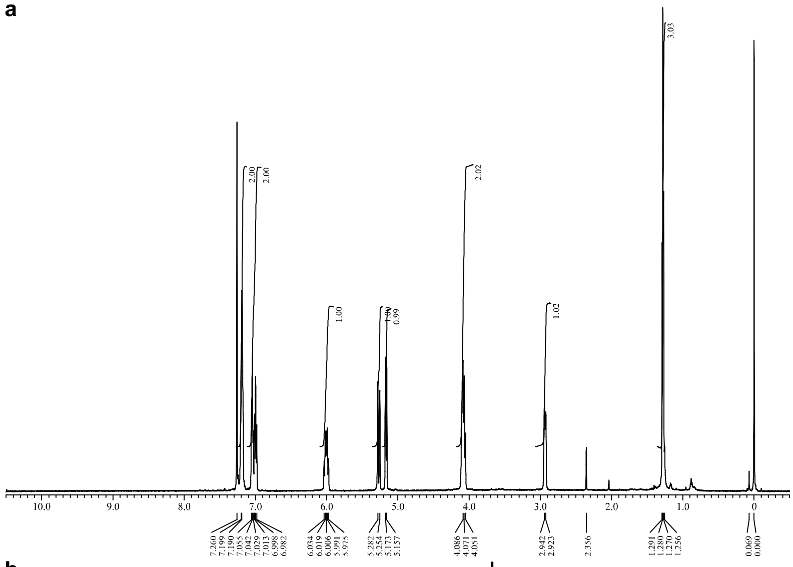

b

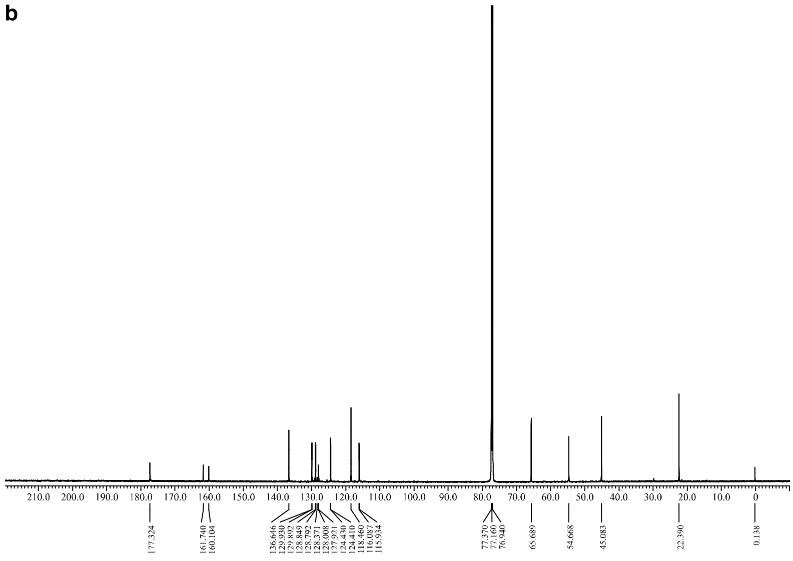

Supplementary Figure 41. ^{1}H-NMR (**a**) and ^{13}C-NMR (**b**) spectra of (2*R*,3*S*)-3-(2-fluorophenyl)-2-((*R*)-1-hydroxyethyl)pent-4-enoic acid ((1*R*,2*R*,3*S*)-*syn*$_{1,2}$*syn*$_{2,3}$-**11ab**) in $CDCl_3$.

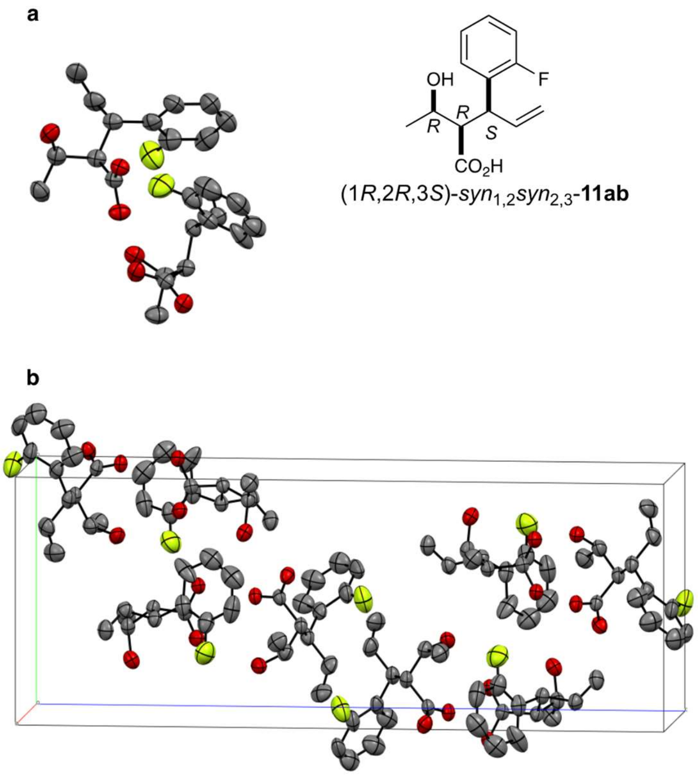

Supplementary Figure 42. ORTEP drawing (**a**) and packing diagram (**b**) of (2*R*,3*S*)-3-(2-fluorophenyl)-2-((*R*)-1-hydroxyethyl)pent-4-enoic acid ((1*R*,2*R*,3*S*)-*syn*$_{1,2}$*syn*$_{2,3}$-**11ab**).

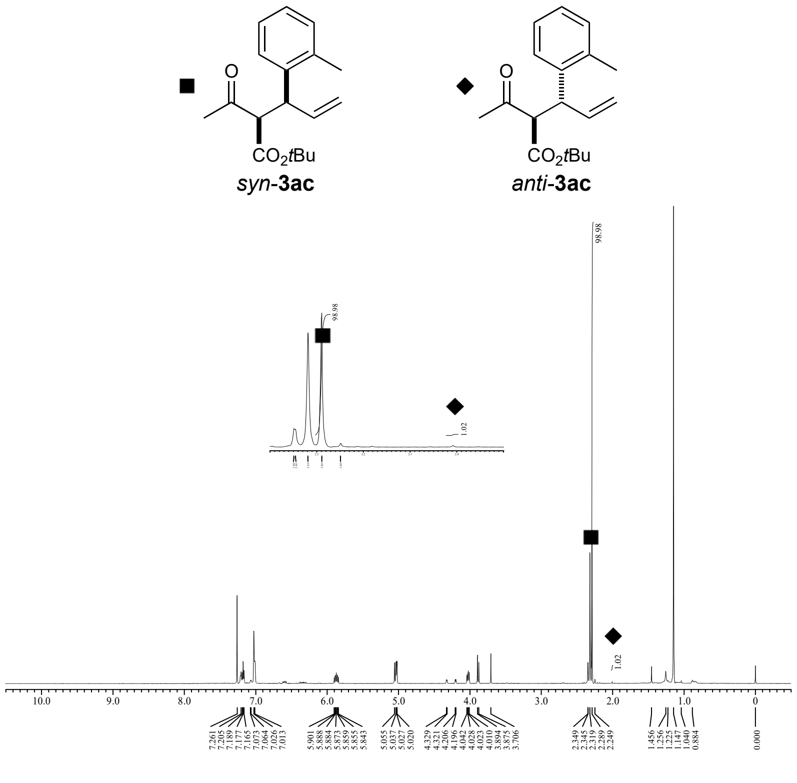

Supplementary Figure 43. ^{1}H-NMR spectrum of 0.500-mmol scale reaction mixture for synthesis of *syn*-**3ac** in $CDCl_3$. Repetition time: 10 sec.

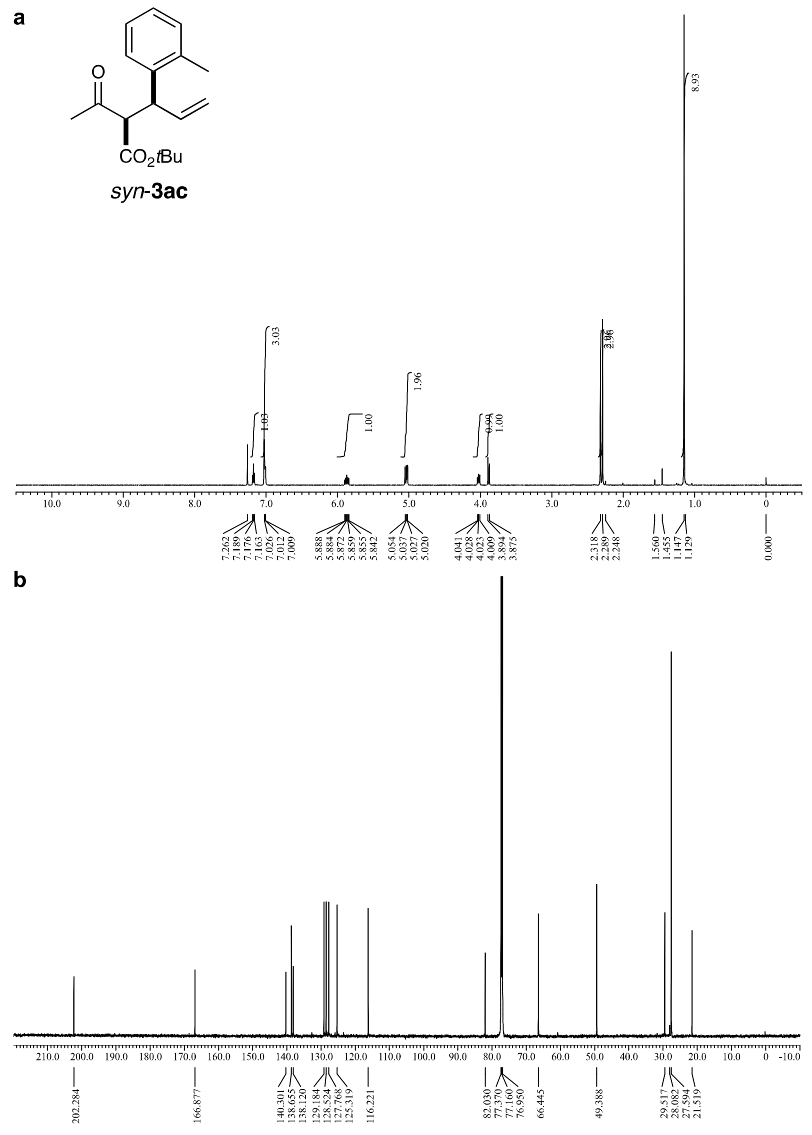

Supplementary Figure 44. ^{1}H-NMR (**a**) and ^{13}C-NMR (**b**) spectra of *tert*-butyl (2*R*,3*S*)-2-acetyl-3-(*o*-tolyl)pent-4-enoate (*syn*-**3ac**) in $CDCl_3$ (**Table 2**, entry 15).

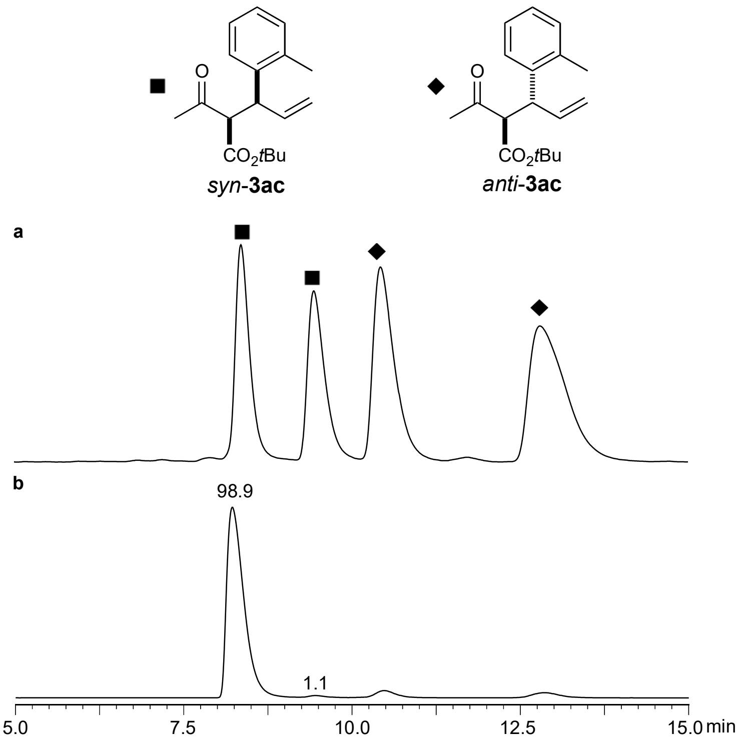

Supplementary Figure 45. HPLC charts of racemic **3ac** (*syn*/*anti* mixture) (**a**) and synthetic (**b**) *tert*-butyl (2*R*,3*S*)-2-acetyl-3-(*o*-tolyl)pent-4-enoate (*syn*-**3ac**) (**Table 2**, entry 15). Conditions: column, CHIRALPAK ID-3; eluent, 1.0:99.0 2-PrOH–Hex; flow rate, 1.00 mL/min; detection, 220-nm light.

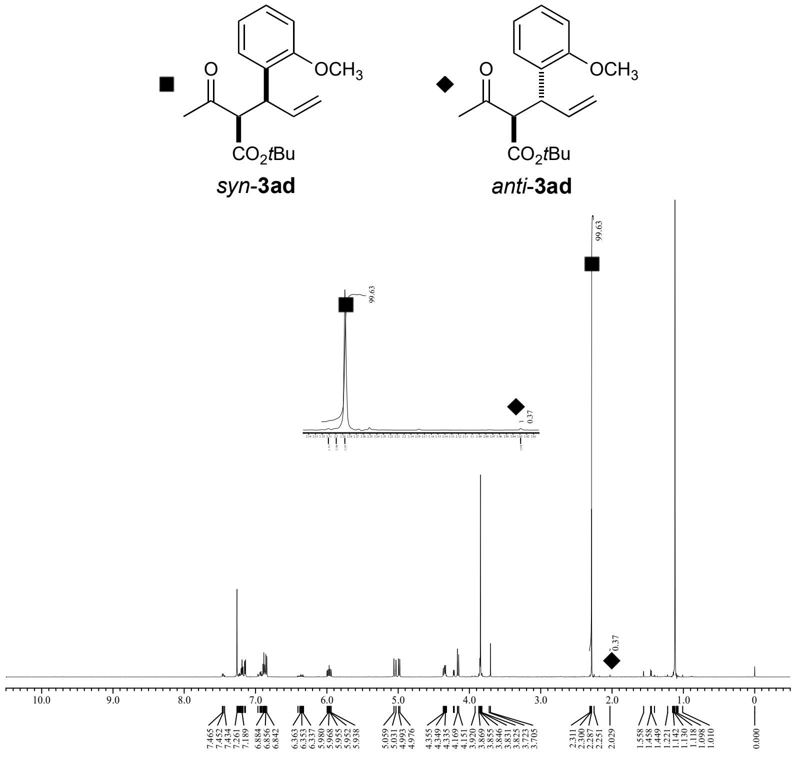

Supplementary Figure 46. ^{1}H-NMR spectrum of 0.500-mmol scale reaction mixture for synthesis of *syn*-**3ad** in $CDCl_3$. Repetition time: 10 sec.

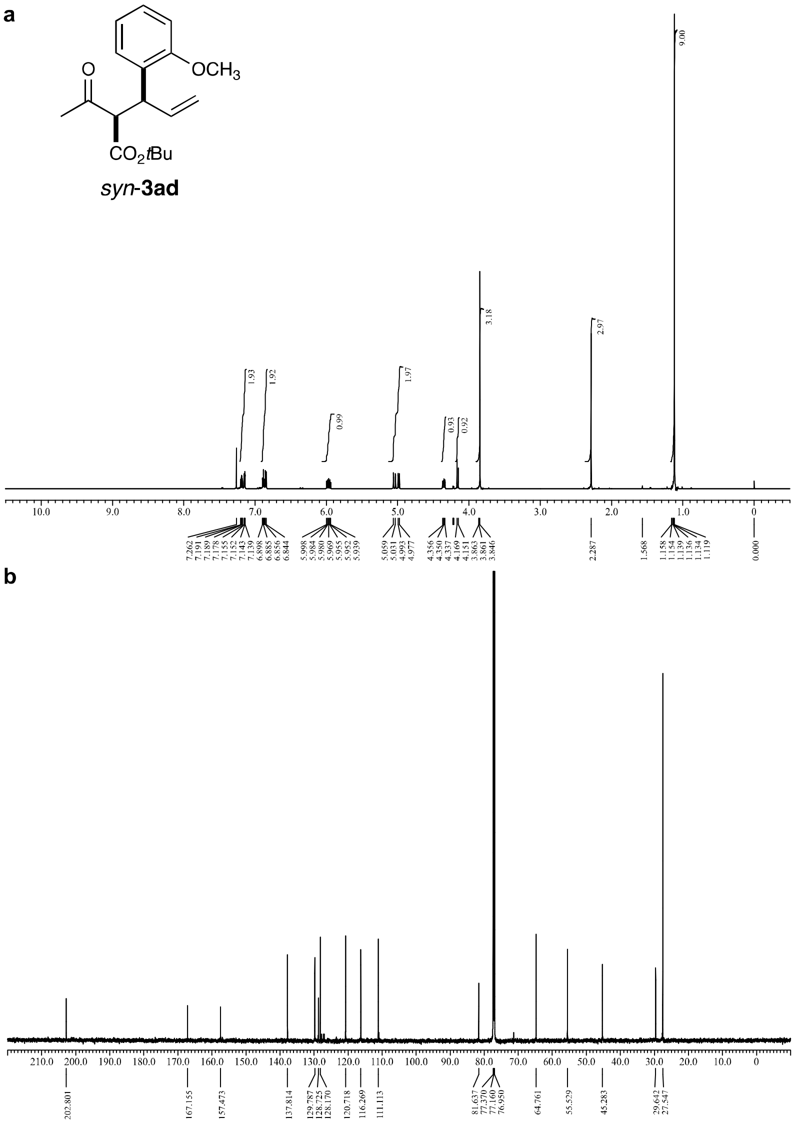

Supplementary Figure 47. ^{1}H-NMR (**a**) and ^{13}C-NMR (**b**) spectra *tert*-butyl (2*R*,3*S*)-2-acetyl-3-(2-methoxyphenyl)pent-4-enoate (*syn*-**3ad**) in CDCl3 (**Table 2**, entry 16).

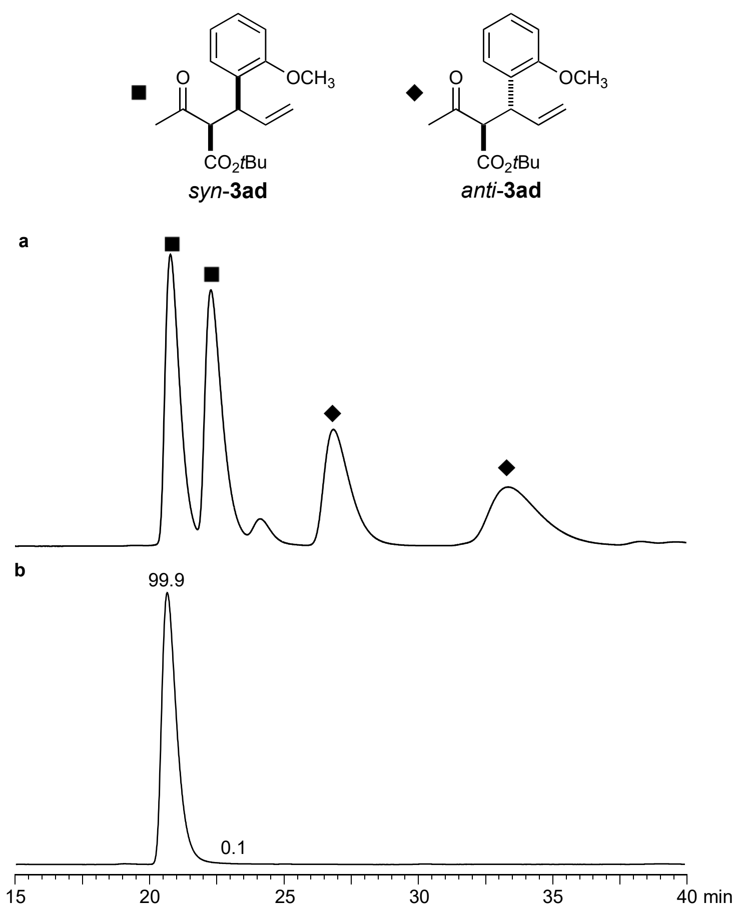

Supplementary Figure 48. HPLC charts of racemic **3ad** (*syn*/*anti* mixture) (**a**) and synthetic (**b**) *tert*-butyl (2*R*,3*S*)-2-acetyl-3-(2-methoxyphenyl) pent-4-enoate (*syn*-**3ad**) (**Table 2**, entry 16). Conditions: column, CHIRALPAK ID-3; eluent, 1.0:99.0 2-PrOH–Hex; flow rate, 0.50 mL/min; detection, 220-nm light.

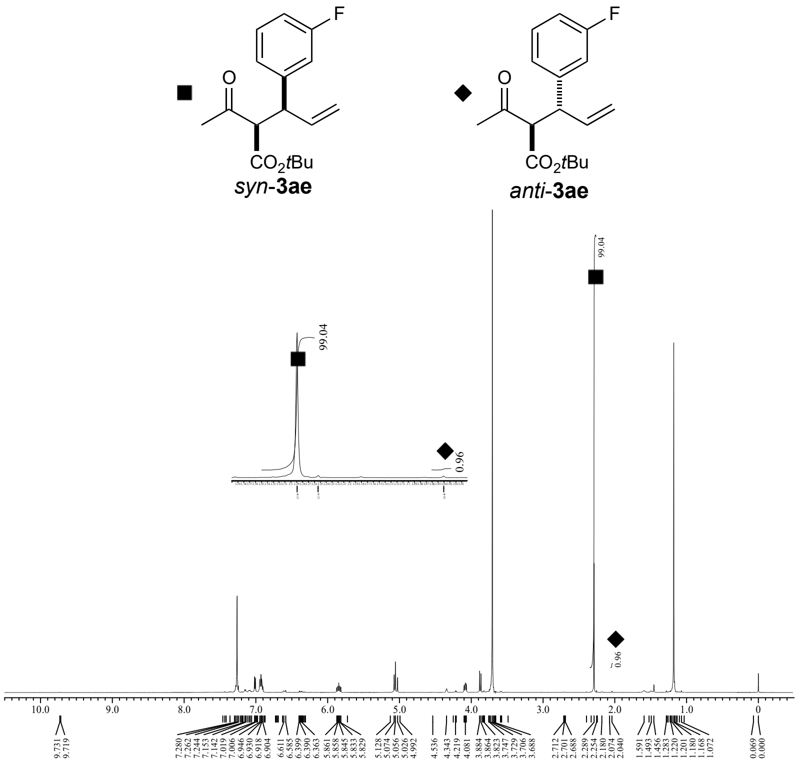

Supplementary Figure 49. ^{1}H-NMR spectrum of 0.500-mmol scale reaction mixture for synthesis of *syn*-**3ae** in $CDCl_3$. Repetition time: 10 sec.

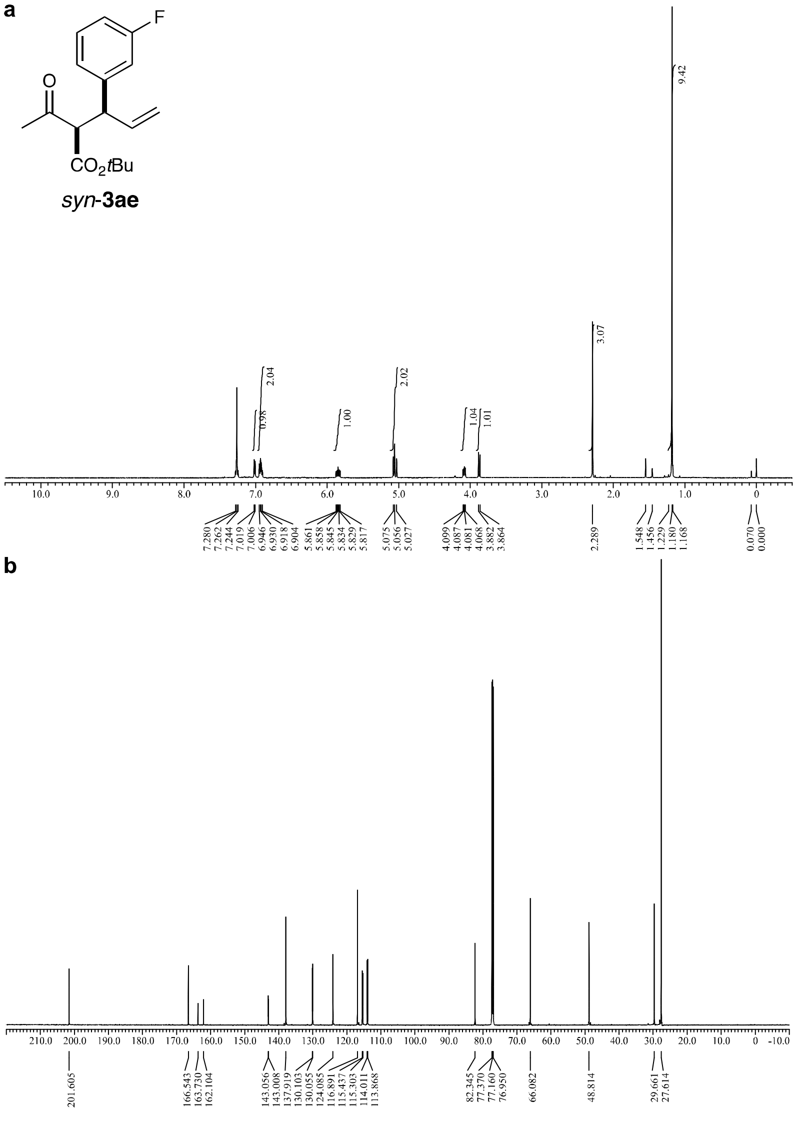

Supplementary Figure 50. ^{1}H-NMR (**a**) and ^{13}C-NMR (**b**) spectra *tert*-butyl (2*R*,3*S*)-2-acetyl-3-(3-fluorophenyl)pent-4-enoate (*syn*-**3ae**) in $CDCl_3$ (**Table 2**, entry 17).

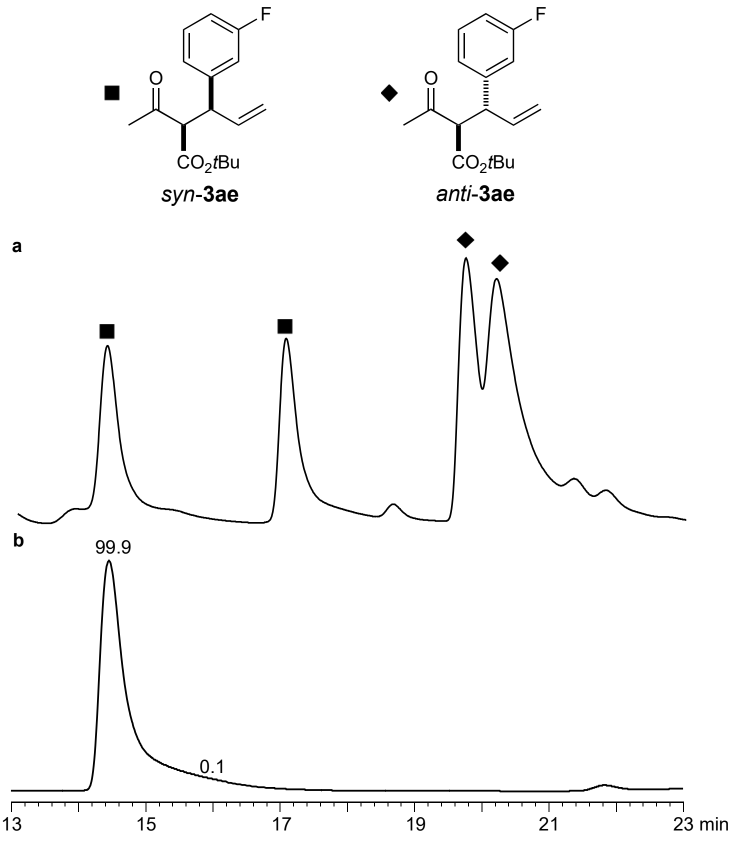

Supplementary Figure 51. HPLC charts of racemic **3ae** (*syn*/*anti* mixture) (**a**) and synthetic (**b**) *tert*-butyl (2*R*,3*S*)-2-acetyl-3-(3-fluorophenyl)pent-4-enoate (*syn*-**3ae**) (**Table 2**, entry 17). Conditions: column, CHIRALPAK IA-3; eluent, 1.0:99.0 2-PrOH–Hex; flow rate, 0.50 mL/min; detection, 220-nm light.

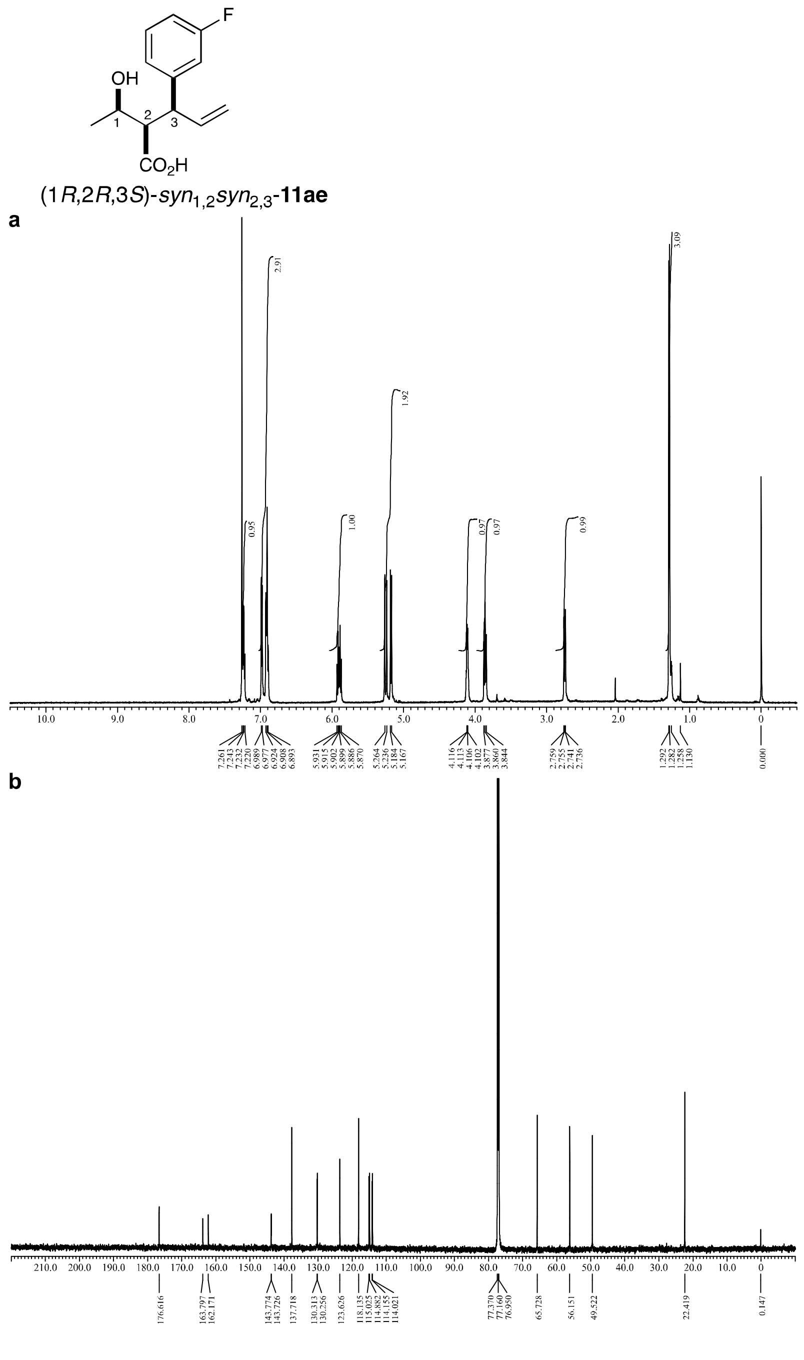

Supplementary Figure 52. ^{1}H-NMR (**a**) and ^{13}C-NMR (**b**) spectra of (2*R*,3*S*)-3-(3-fluorophenyl)-2-((*R*)-1-hydroxyethyl)pent-4-enoic acid ((1*R*,2*R*,3*S*)-*syn*$_{1,2}$*syn*$_{2,3}$-**11ae**) in $CDCl_3$.

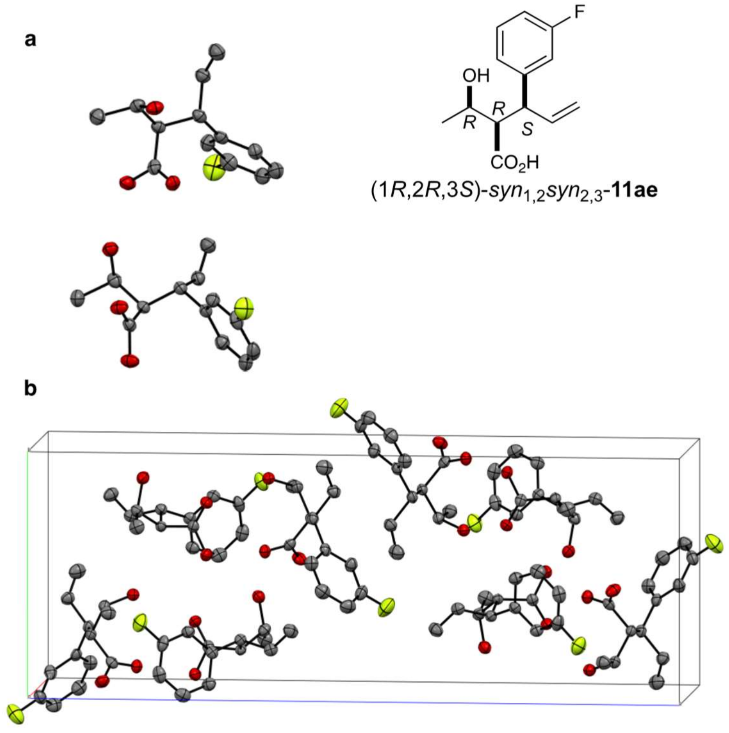

Supplementary Figure 53. ORTEP drawing (**a**) and packing diagram (**b**) of (2*R*,3*S*)-3-(3-fluorophenyl)-2-((*R*)-1-hydroxyethyl)pent-4-enoic acid ((1*R*,2*R*,3*S*)-*syn*$_{1,2}$*syn*$_{2,3}$-**11ae**).

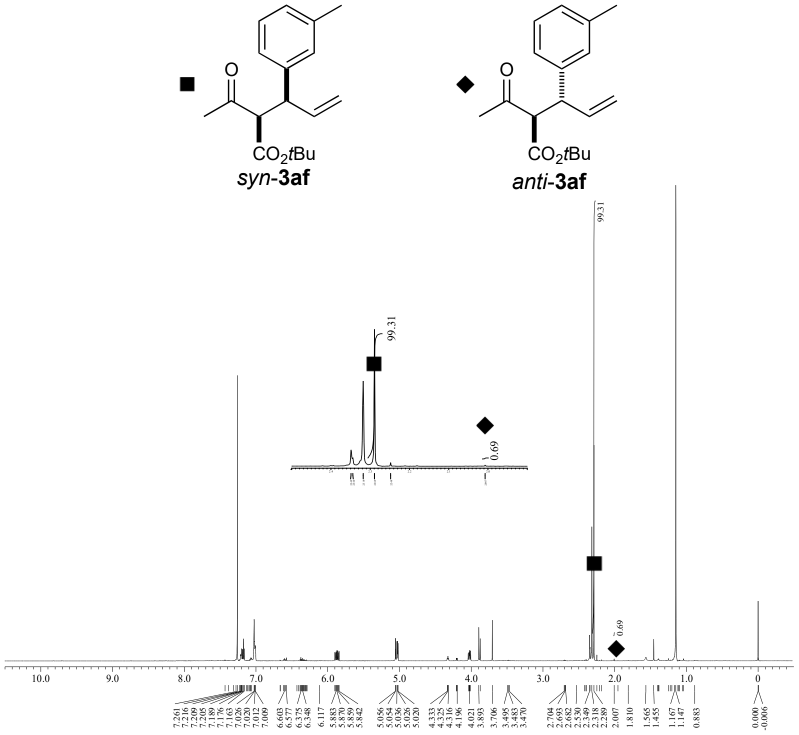

Supplementary Figure 54. ^{1}H-NMR spectrum of 0.500-mmol scale reaction mixture for synthesis of *syn*-**3af** in $CDCl_3$. Repetition time: 10 sec.

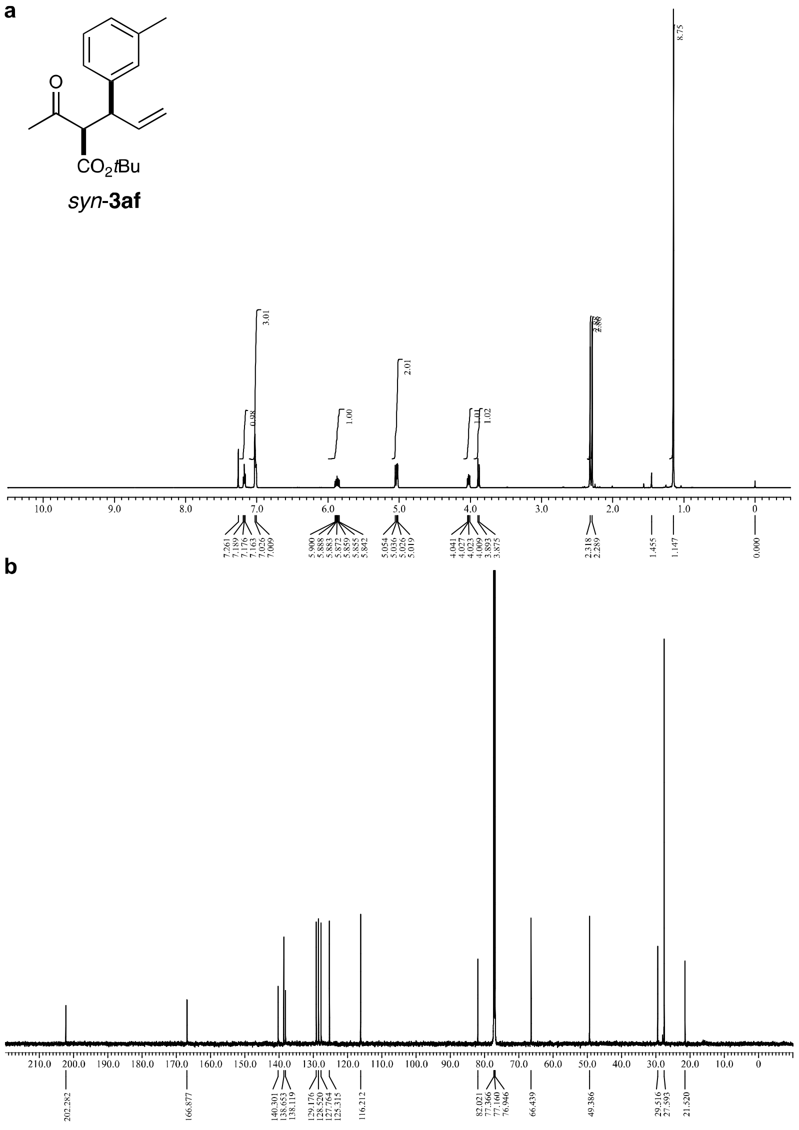

Supplementary Figure 55. ^{1}H-NMR (**a**) and ^{13}C-NMR (**b**) spectra of *tert*-butyl (2*R*,3*S*)-2-acetyl-3-(*m*-tolyl)pent-4-enoate (*syn*-**3af**) in $CDCl_3$ (**Table 2**, entry 18).

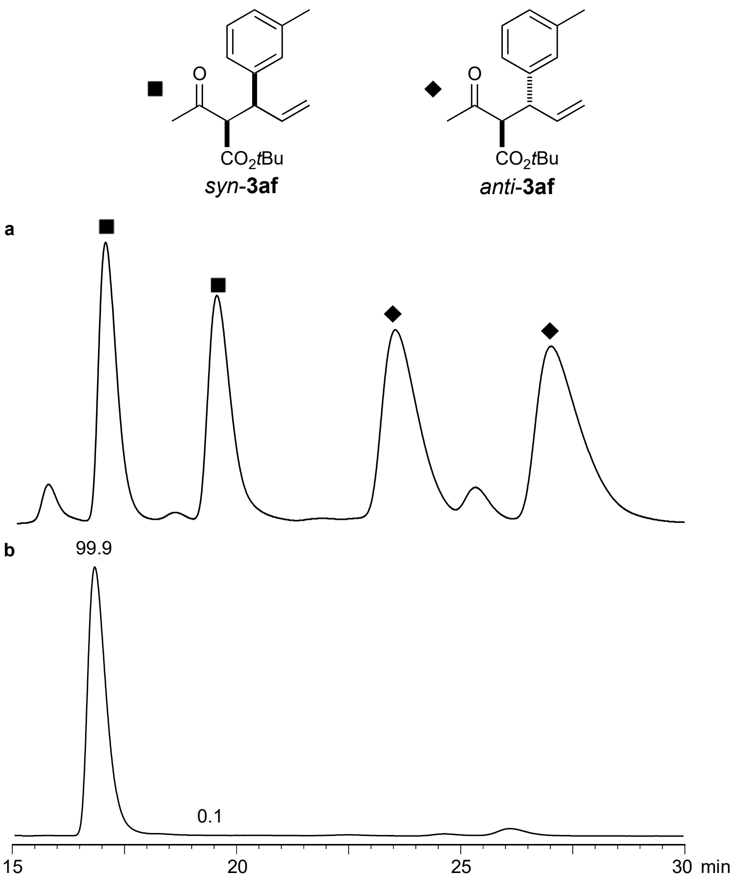

Supplementary Figure 56. HPLC charts of racemic **3af** (*syn*/*anti* mixture) (**a**) and synthetic (**b**) *tert*-butyl (2*R*,3*S*)-2-acetyl-3-(*m*-tolyl)pent-4-enoate (*syn*-**3af**) (**Table 2**, entry 18). Conditions: column, CHIRALPAK ID-3; eluent, 1.0:99.0 2-PrOH–Hex; flow rate, 0.50 mL/min; detection, 220-nm light.

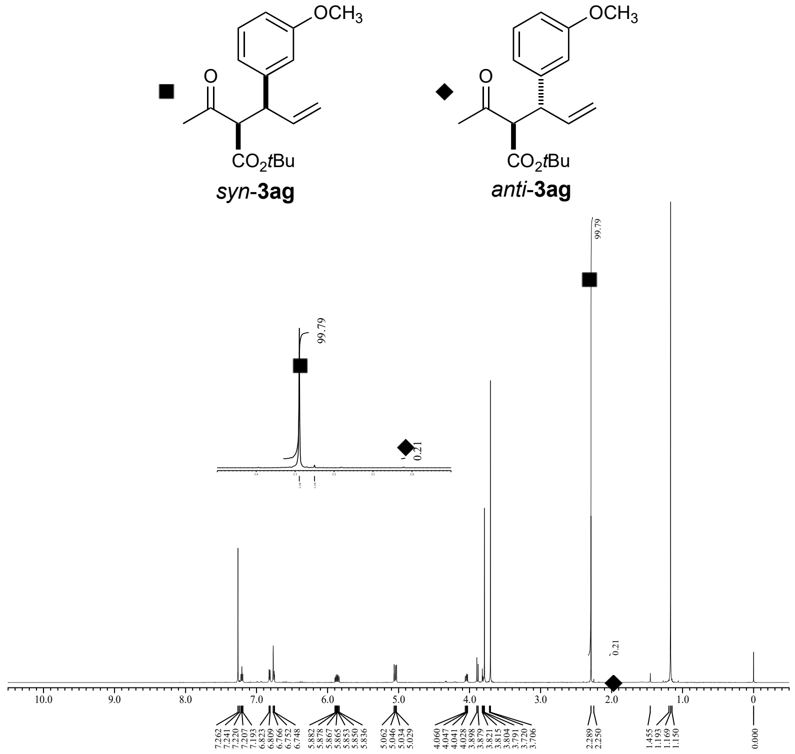

Supplementary Figure 57. ^{1}H-NMR spectrum of 0.500-mmol scale reaction mixture for synthesis of *syn*-**3ag** in $CDCl_3$. Repetition time: 10 sec.

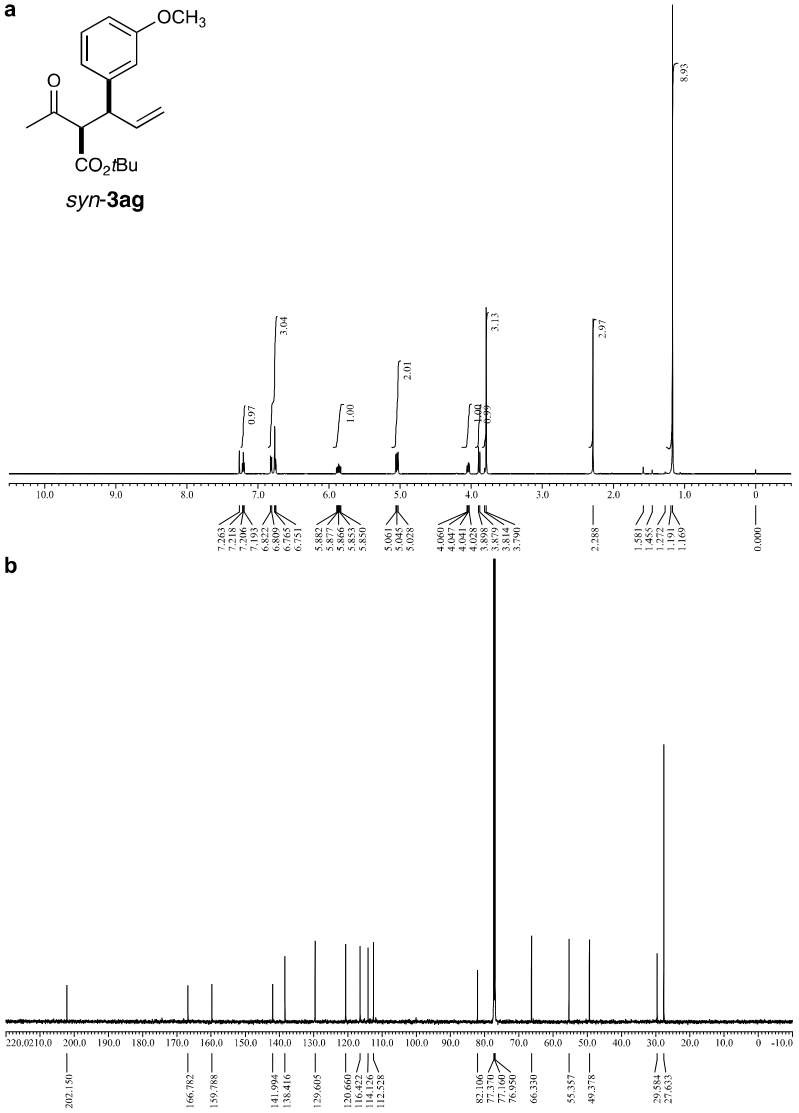

Supplementary Figure 58. ^{1}H-NMR (**a**) and ^{13}C-NMR (**b**) spectra of *tert*-butyl (2*R*,3*S*)-2-acetyl-3-(3-methoxyphenyl)pent-4-enoate (*syn*-**3ag**) in $CDCl_3$ (**Table 2**, entry 19).

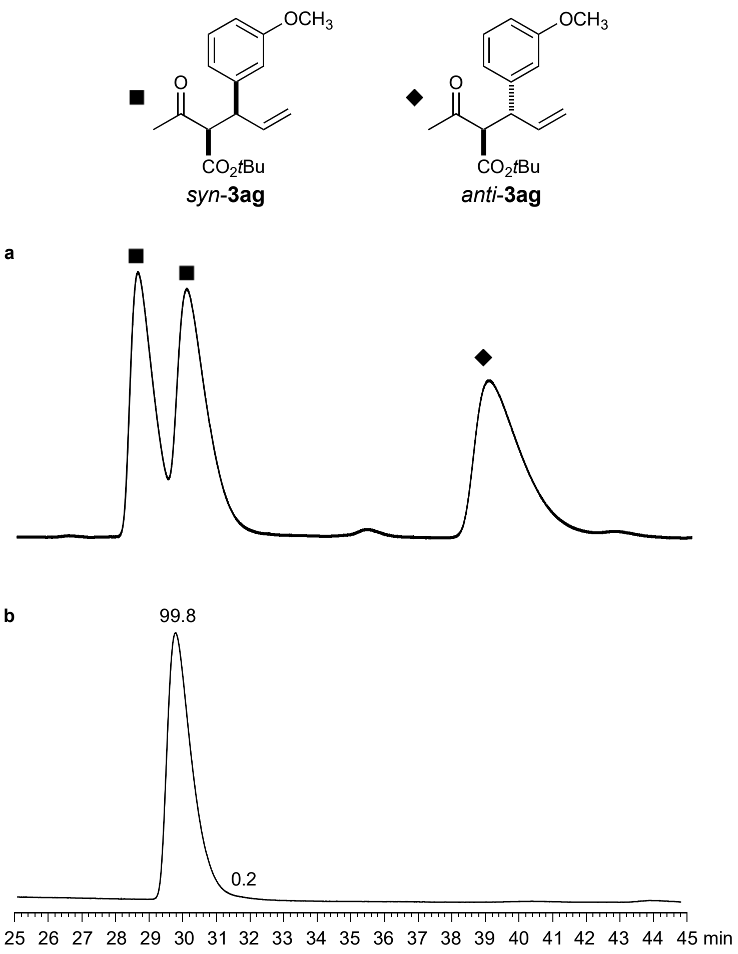

Supplementary Figure 59. HPLC charts of racemic **3ag** (*syn*/*anti* mixture) (**a**) and synthetic (**b**) *tert*-butyl (2*R*,3*S*)-2-acetyl-3-(3-methoxyphenyl)pent-4-enoate (*syn*-**3ag**) (**Table 2**, entry 19). Conditions: column, CHIRALPAK ID-3; eluent, 1.0:99.0 2-PrOH–Hex; flow rate, 0.50 mL/min; detection, 220-nm light.

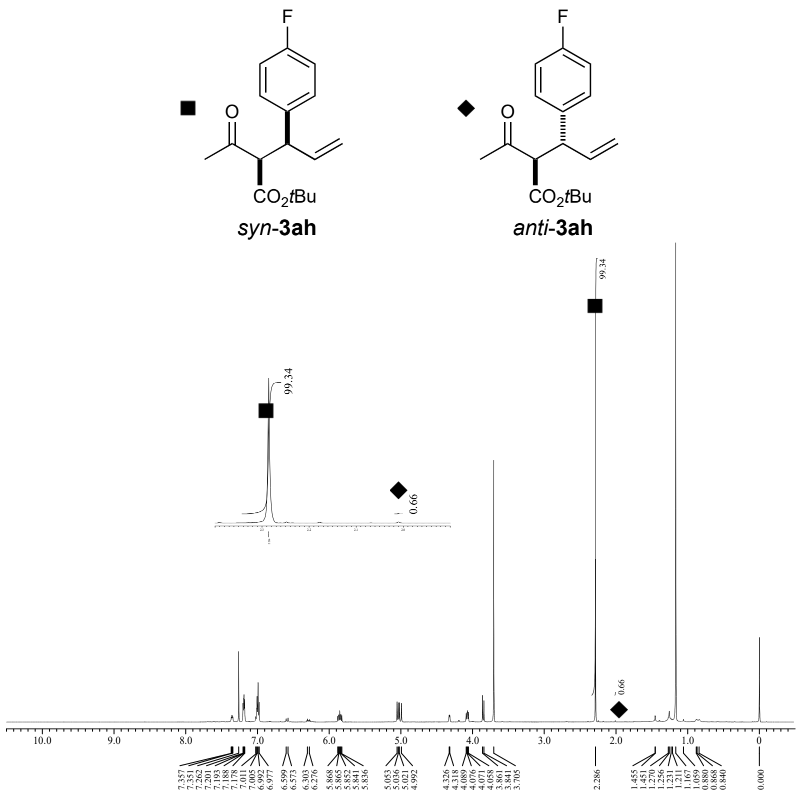

Supplementary Figure 60. ^{1}H-NMR spectrum of 0.500-mmol scale reaction mixture for synthesis of *syn*-**3ah** in $CDCl_3$. Repetition time: 10 sec.

a

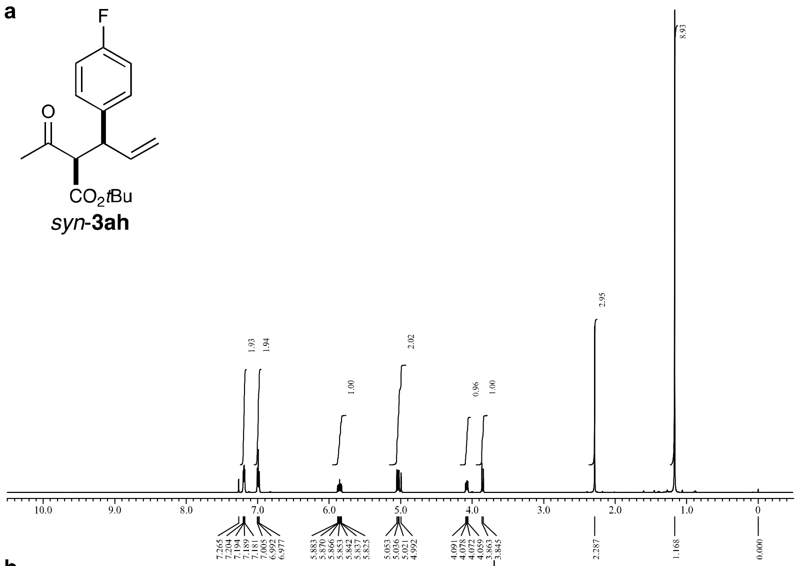

b

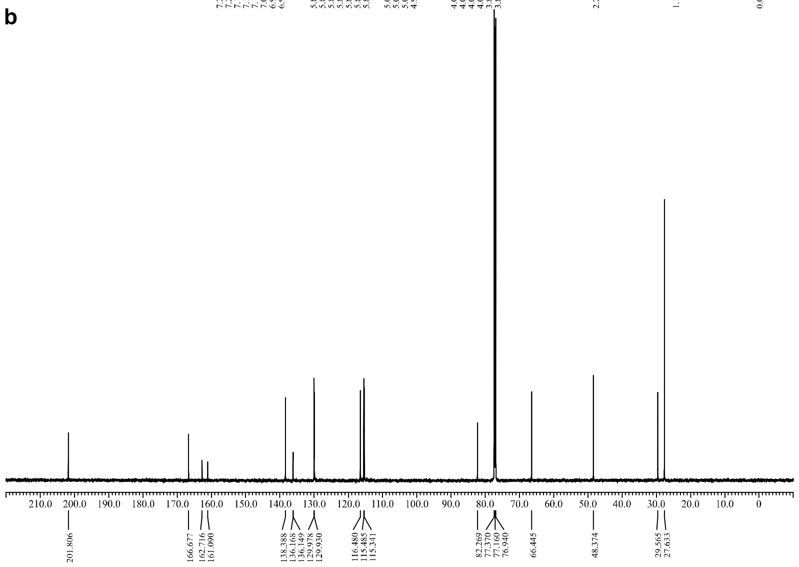

Supplementary Figure 61. ^{1}H-NMR (**a**) and ^{13}C-NMR (**b**) spectra of *tert*-butyl (2*R*,3*S*)-2-acetyl-3-(4-fluorophenyl)pent-4-enoate (*syn*-**3ah**) in $CDCl_3$ (**Table 2**, entry 20).

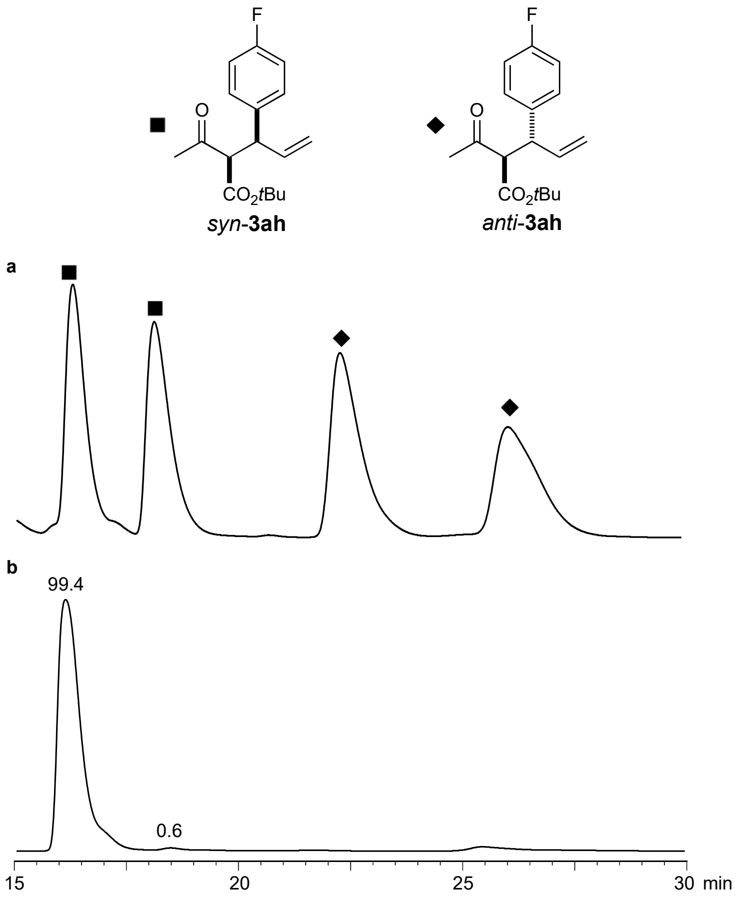

Supplementary Figure 62. HPLC charts of racemic **3ah** (*syn*/*anti* mixture) (**a**) and synthetic (**b**) *tert*-butyl (2*R*,3*S*)-2-acetyl-3-(4-fluorophenyl)pent-4-enoate (*syn*-**3ah**) (**Table 1**, entry 20). Conditions: column, CHIRALPAK ID-3; eluent, 1.0:99.0 2-PrOH–Hex; flow rate, 0.50 mL/min; detection, 220-nm light.

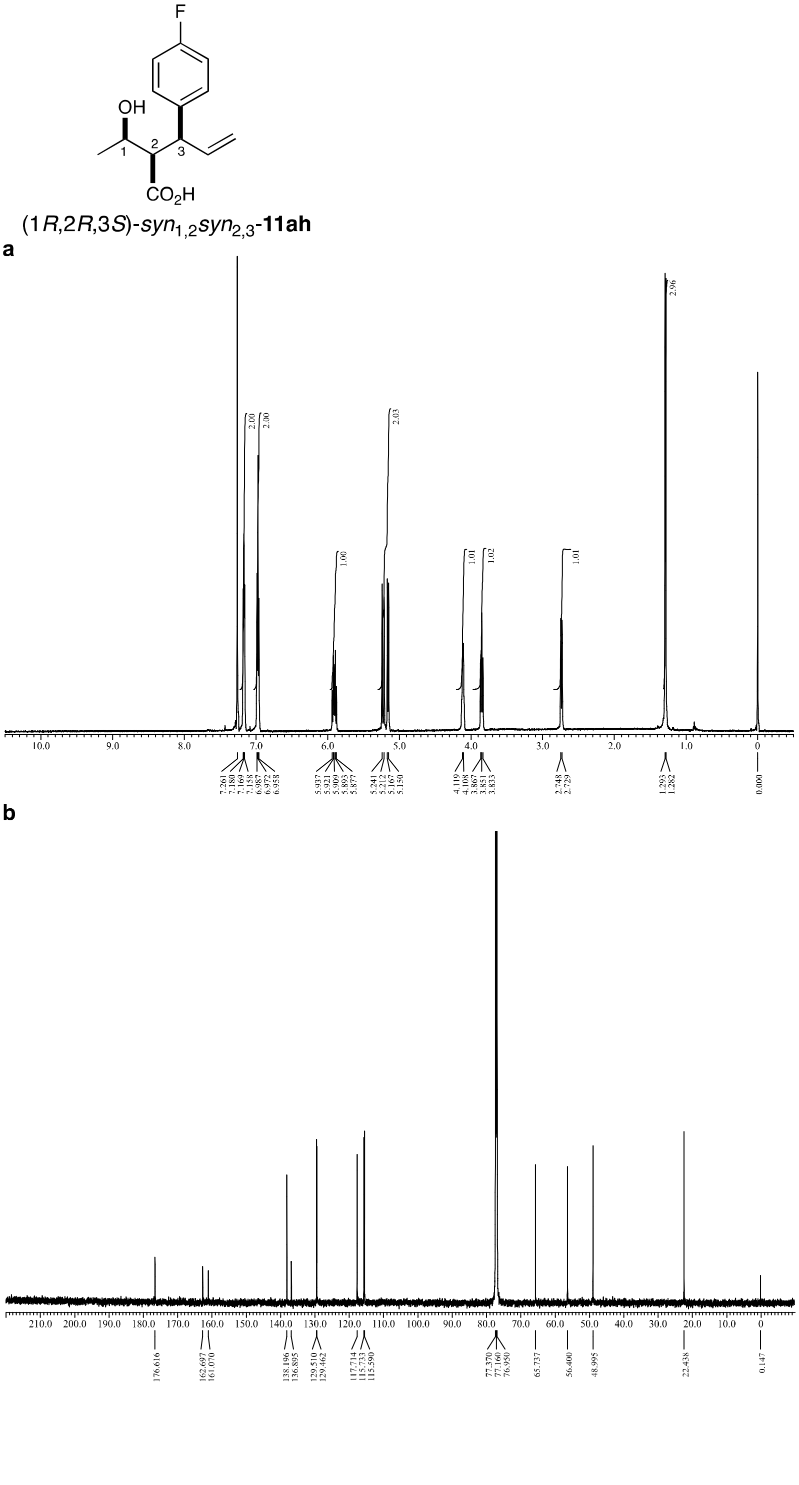

Supplementary Figure 63. ^{1}H-NMR (**a**) and ^{13}C-NMR (**b**) spectra of (2*R*,3*S*)-3-(4-fluorophenyl)-2-((*R*)-1-hydroxyethyl)pent-4-enoic acid ((1*R*,2*R*,3*S*)-*syn*1,2*syn*2,3-**11ah**) in $CDCl_3$.

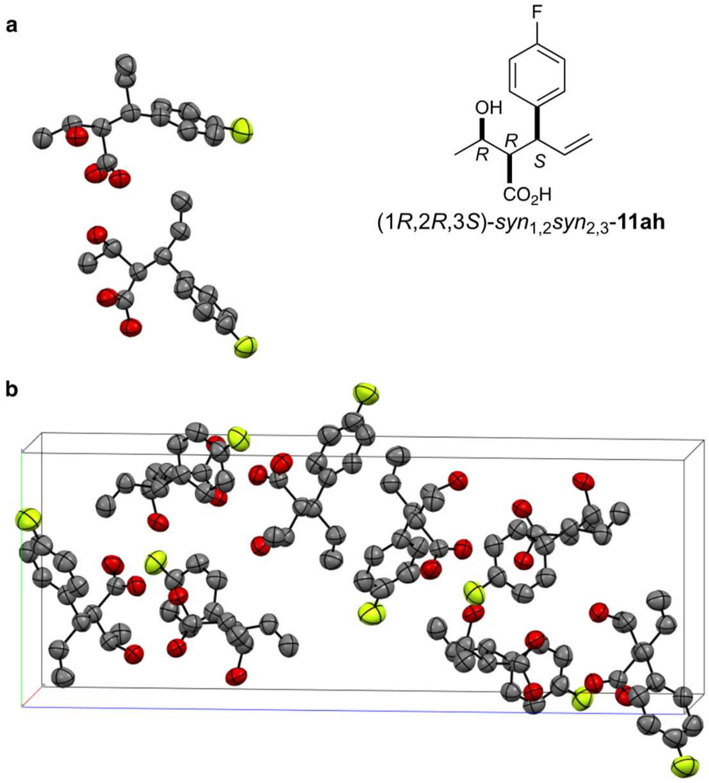

Supplementary Figure 64. ORTEP drawing (**a**) and packing diagram (**b**) of (2*R*,3*S*)-3-(4-fluorophenyl)-2-((*R*)-1-hydroxyethyl)pent-4-enoic acid ((1*R*,2*R*,3*S*)-*syn*$_{1,2}$*syn*$_{2,3}$-**11ah**).

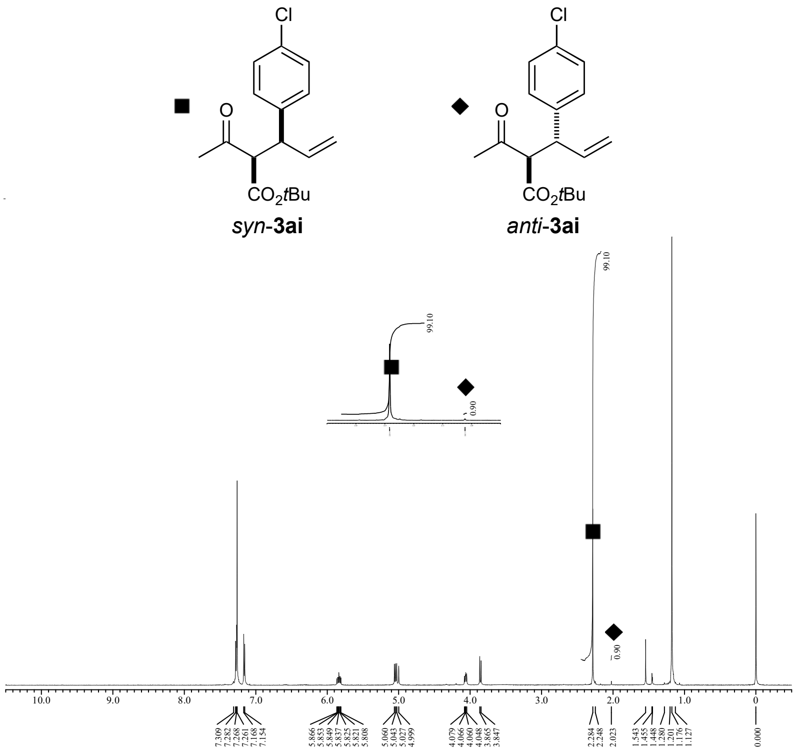

Supplementary Figure 65. ^{1}H-NMR spectrum of 0.500-mmol scale reaction mixture for synthesis of *syn*-**3ai** in $CDCl_3$. Repetition time: 10 sec.

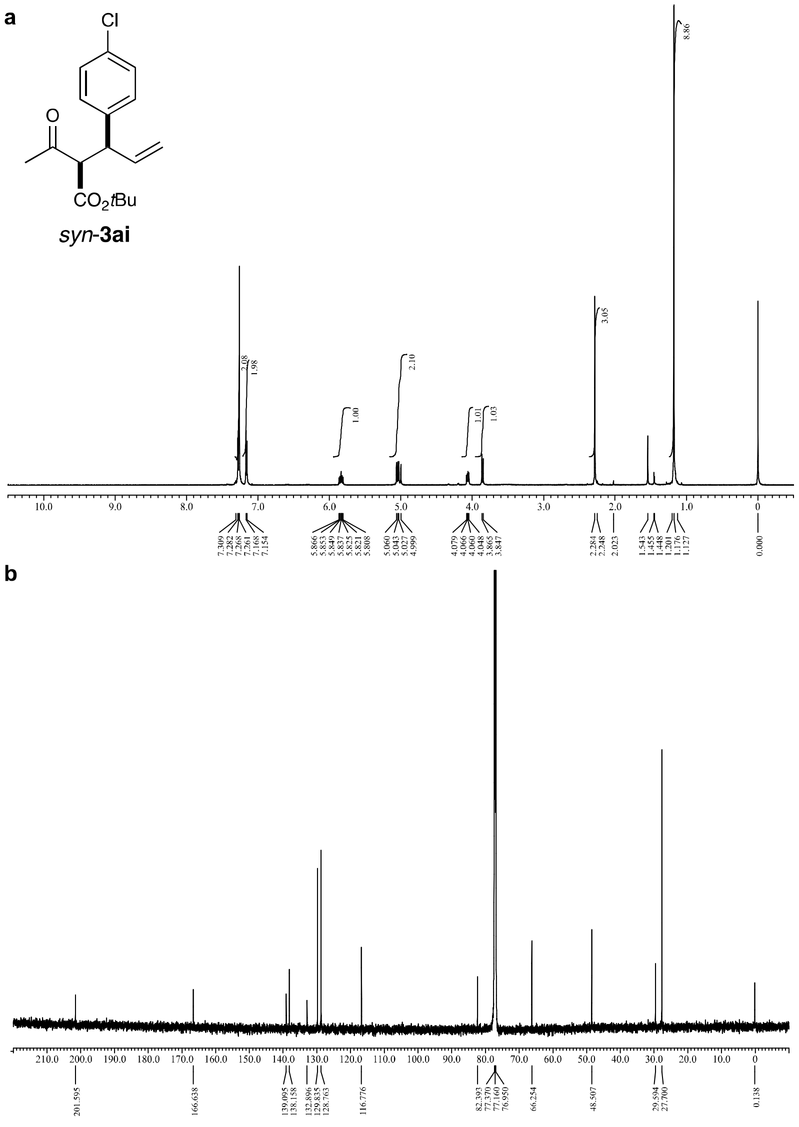

Supplementary Figure 66. ^{1}H-NMR (**a**) and ^{13}C-NMR (**b**) spectra of *tert*-butyl (2*R*,3*S*)-2-acetyl-3-(4-chlorophenyl)pent-4-enoate (*syn*-**3ai**) in $CDCl_3$ (**Table 2**, entry 21).

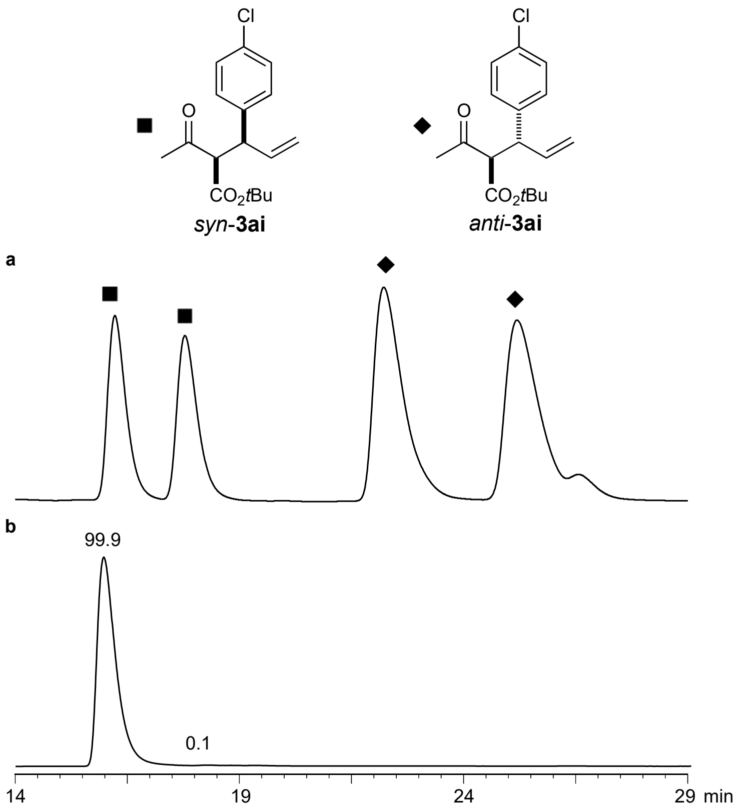

Supplementary Figure 67. HPLC charts of racemic **3ai** (*syn*/*anti* mixture) (**a**) and synthetic (**b**) *tert*-butyl (2*R*,3*S*)-2-acetyl-3-(4-chlorophenyl)pent-4-enoate (*syn*-**3ai**) (**Table 2**, entry 21). Conditions: column, CHIRALPAK ID-3; eluent, 1.0:99.0 2-PrOH–Hex; flow rate, 0.50 mL/min; detection, 220-nm light.

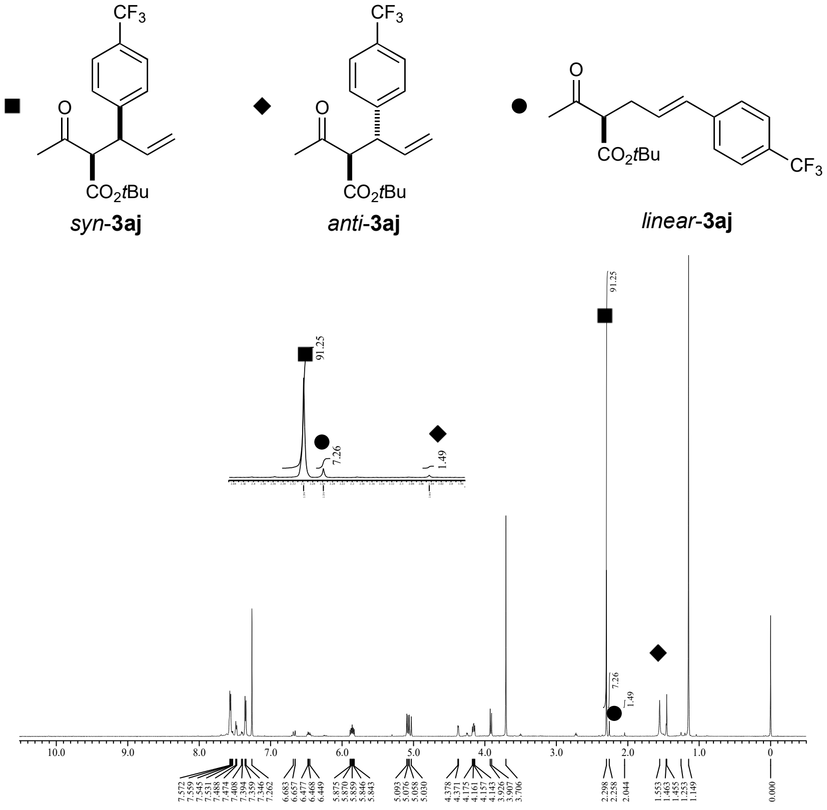

Supplementary Figure 68. ^{1}H-NMR spectrum of 0.500-mmol scale reaction mixture for synthesis of *syn*-**3aj** in $CDCl_3$. Repetition time: 10 sec.

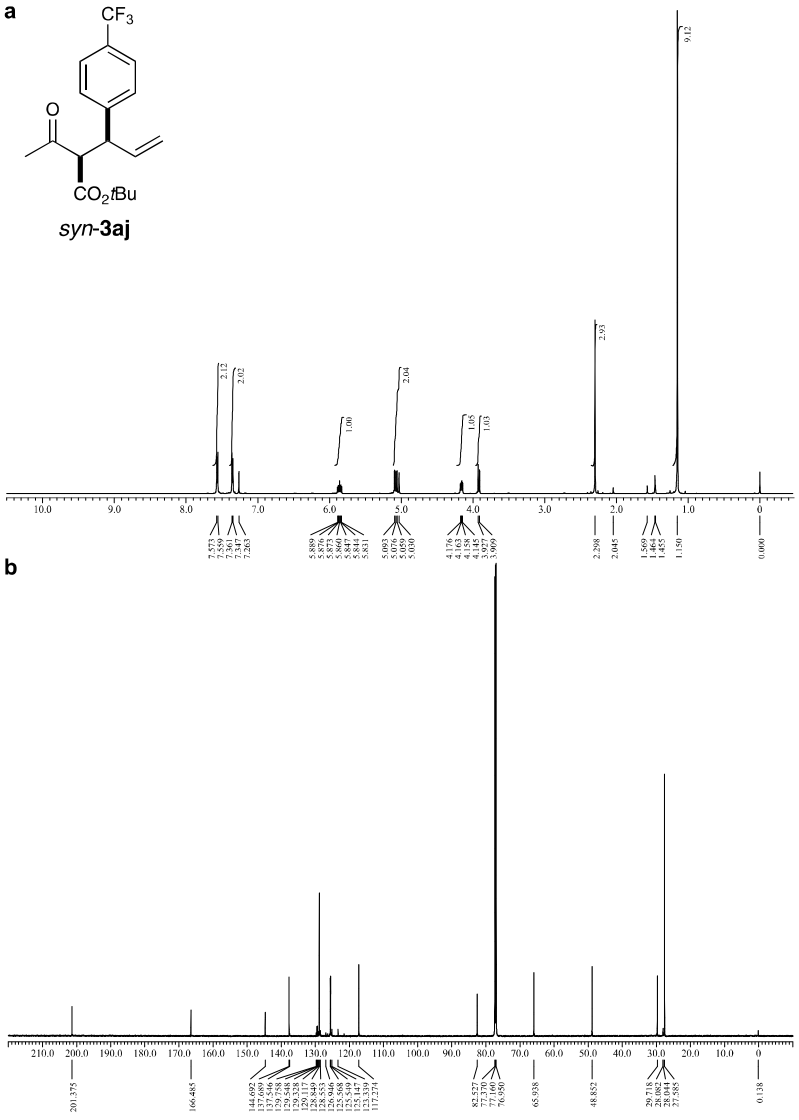

Supplementary Figure 69. ^{1}H-NMR (**a**) and ^{13}C-NMR (**b**) spectra of *tert*-butyl (2*R*,3*S*)-2-acetyl-3-(4-(trifluoromethyl)phenyl)pent-4-enoate (*syn*-**3aj**) in $CDCl_3$ (**Table 2**, entry 22).

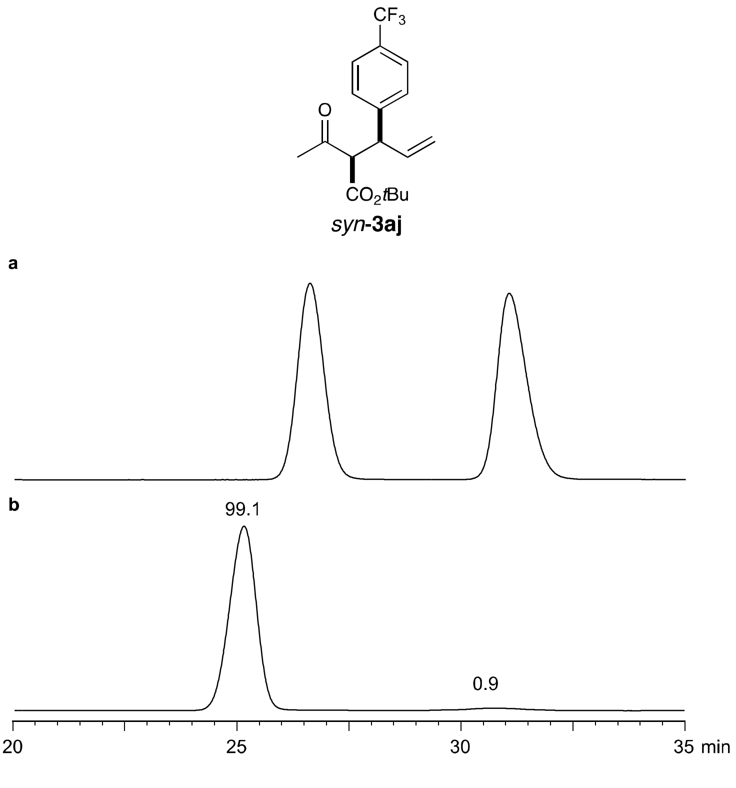

Supplementary Figure 70. HPLC charts of racemic (**a**) and synthetic (**b**) *tert*-butyl (2*R*,3*S*)-2-acetyl-3-(4-(trifluoromethyl)phenyl)pent-4-enoate ((2*R*,3*S*)-*syn*-**3aj**) (**Table 2**, entry 22). Conditions: column, IG; eluent, 0.5:99.5 2-PrOH–Hex; flow rate, 0.50 mL/min; detection, 220-nm light.

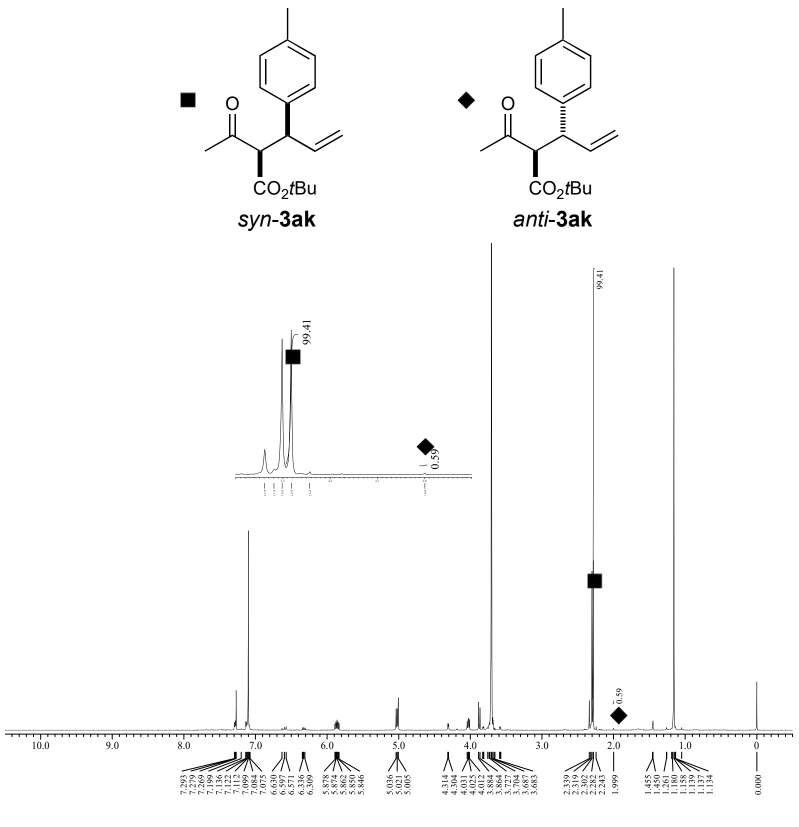

Supplementary Figure 71. ^{1}H-NMR spectrum of 0.500-mmol scale reaction mixture for synthesis of *syn*-**3ak** in $CDCl_3$. Repetition time: 10 sec.

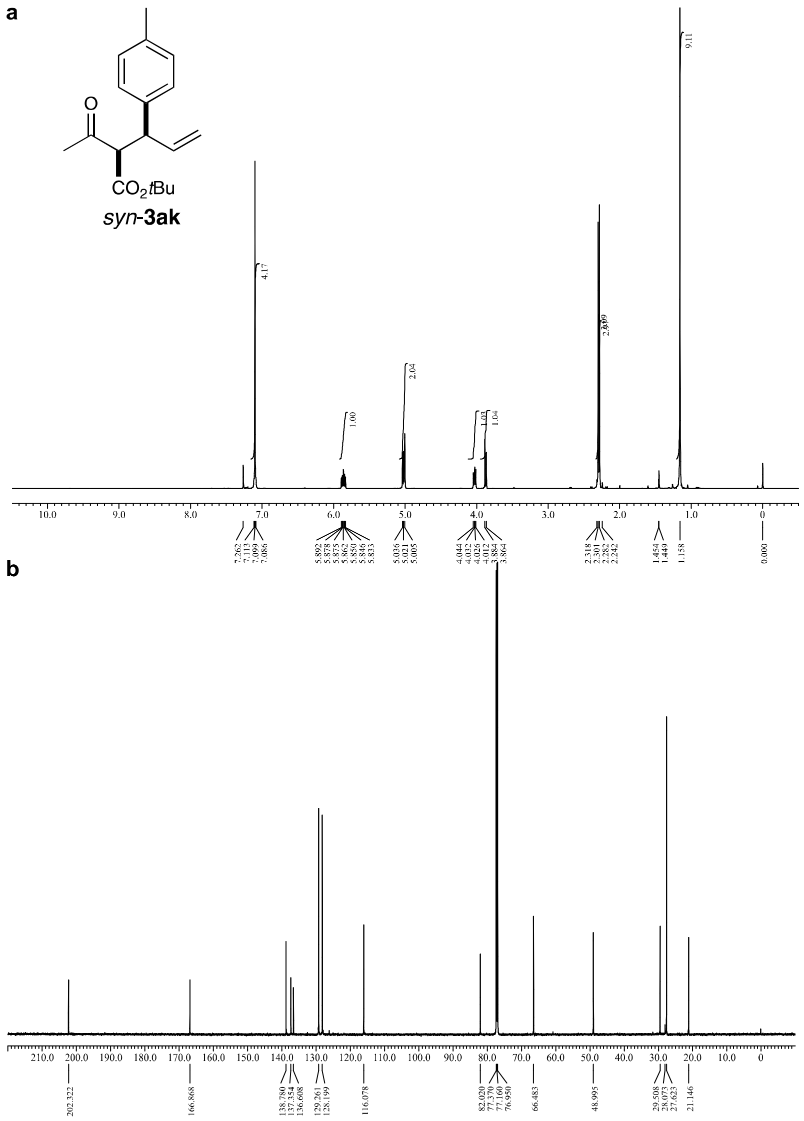

Supplementary Figure 72. ^{1}H-NMR (**a**) and ^{13}C-NMR (**b**) spectra of *tert*-butyl (2*R*,3*S*)-2-acetyl-3-(*p*-tolyl)pent-4-enoate (*syn*-**3ak**) in $CDCl_3$ (**Table 2**, entry 23).

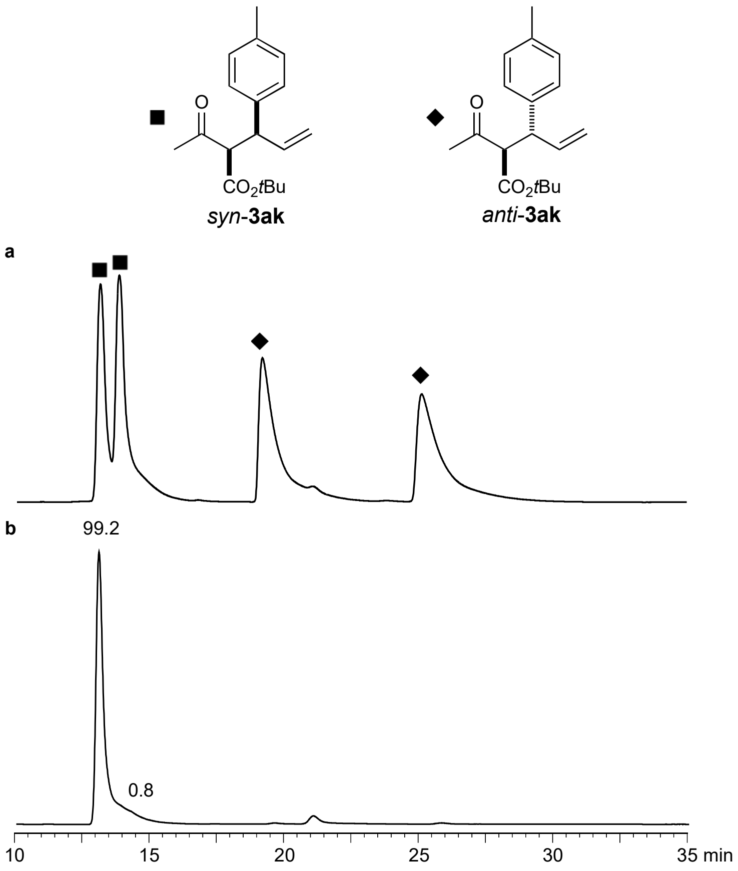

Supplementary Figure 73. HPLC charts of racemic **3ak** (*syn*/*anti* mixture) (**a**) and synthetic (**b**) *tert*-butyl (2*R*,3*S*)-2-acetyl-3-(*p*-tolyl)pent-4-enoate (*syn*-**3ak**) (**Table 2**, entry 23). Conditions: column, CHIRALPAK IA-3; eluent, 1.0:99.0 2-PrOH–Hex; flow rate, 0.50 mL/min; detection, 220-nm light.

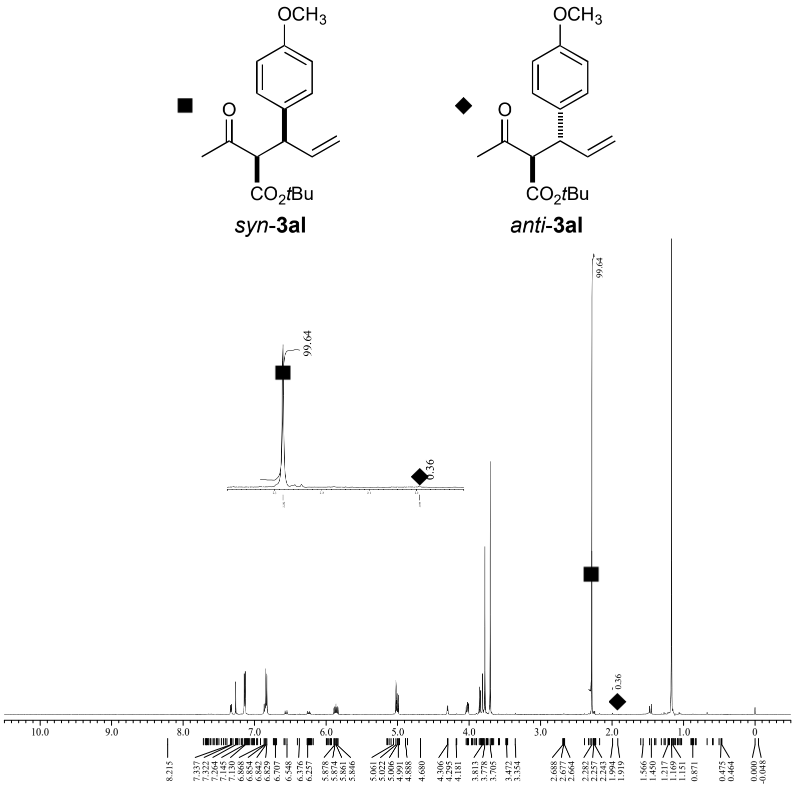

Supplementary Figure 74. ^{1}H-NMR spectrum of 0.500-mmol scale reaction mixture for synthesis of *syn*-**3al** in $CDCl_3$. Repetition time: 10 sec.

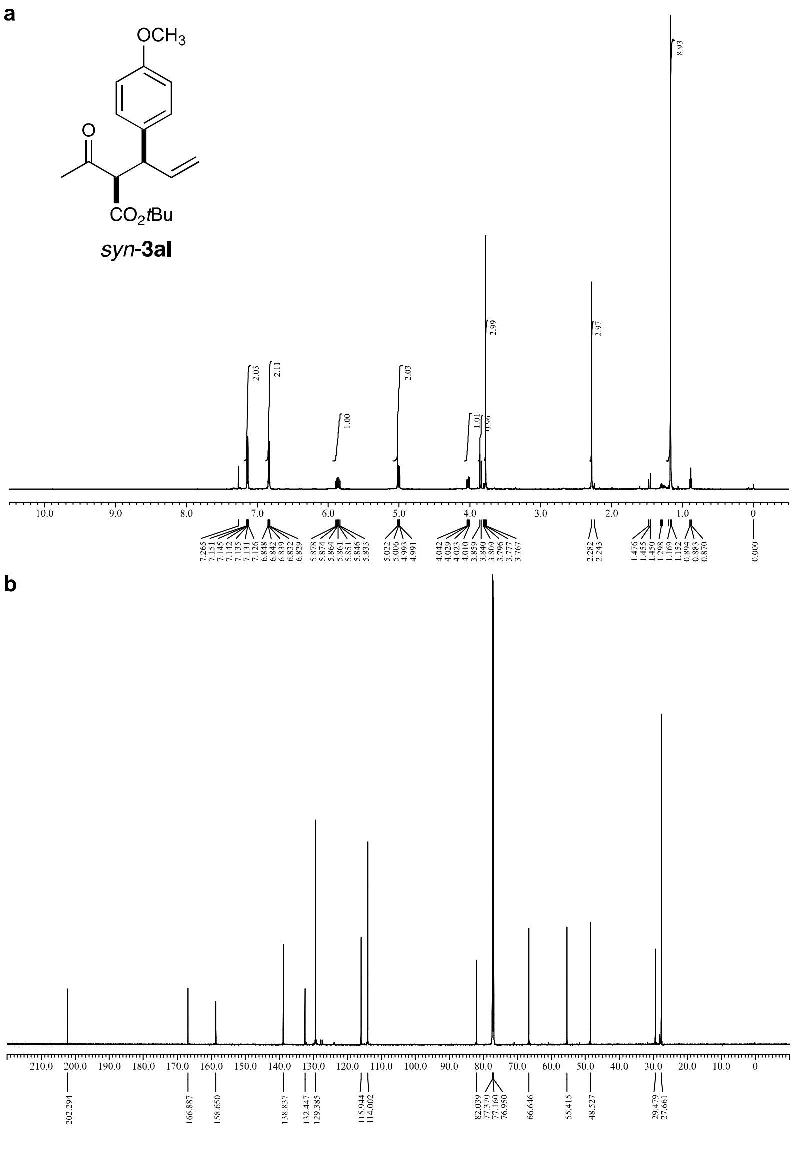

Supplementary Figure 75. ^{1}H-NMR (**a**) and ^{13}C-NMR (**b**) spectra of *tert*-butyl (2*R*,3*S*)-2-acetyl-3-(4-methoxyphenyl)pent-4-enoate (*syn*-**3al**) in $CDCl_3$ (**Table 2**, entry 24).

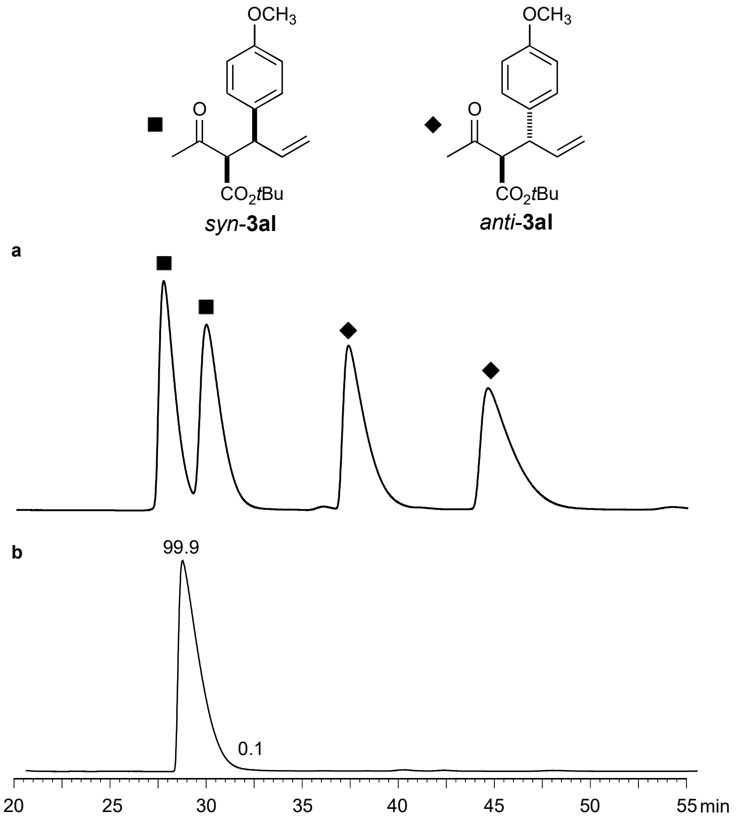

Supplementary Figure 76. HPLC charts of racemic **3al** (*syn*/*anti* mixture) (**a**) and synthetic (**b**) *tert*-butyl (2*R*,3*S*)-2-acetyl-3-(4-methoxyphenyl)pent-4-enoate (*syn*-**3al**) (**Table 2**, entry 24). Conditions: column, CHIRALPAK ID-3; eluent, 1.0:99.0 2-PrOH–Hex; flow rate, 0.50 mL/min; detection, 220-nm light.

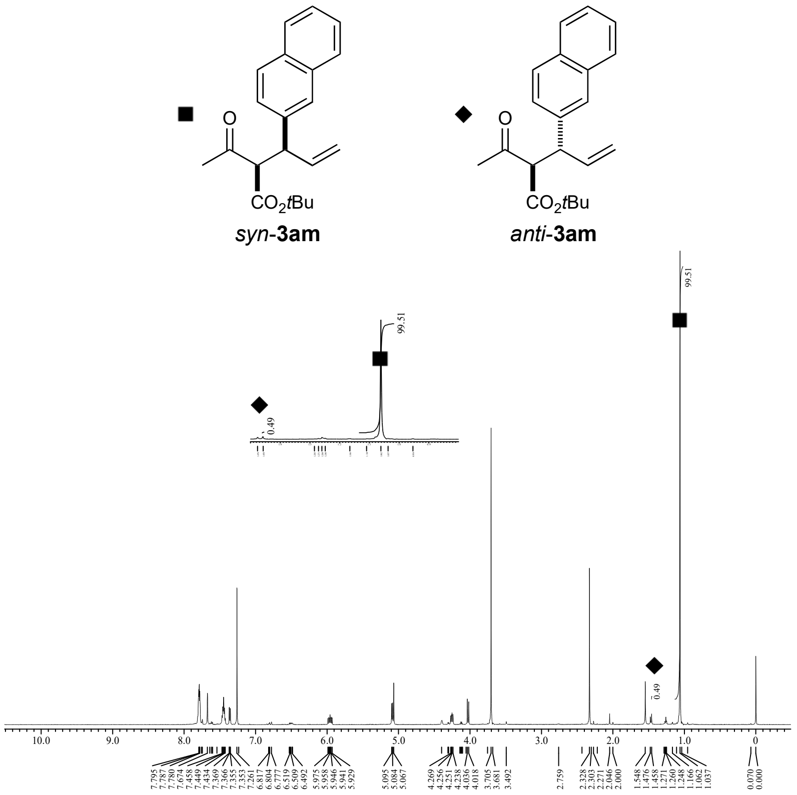

Supplementary Figure 77. ^{1}H-NMR spectrum of 0.500-mmol scale reaction mixture for synthesis of *syn*-**3am** in $CDCl_3$. Repetition time: 10 sec.

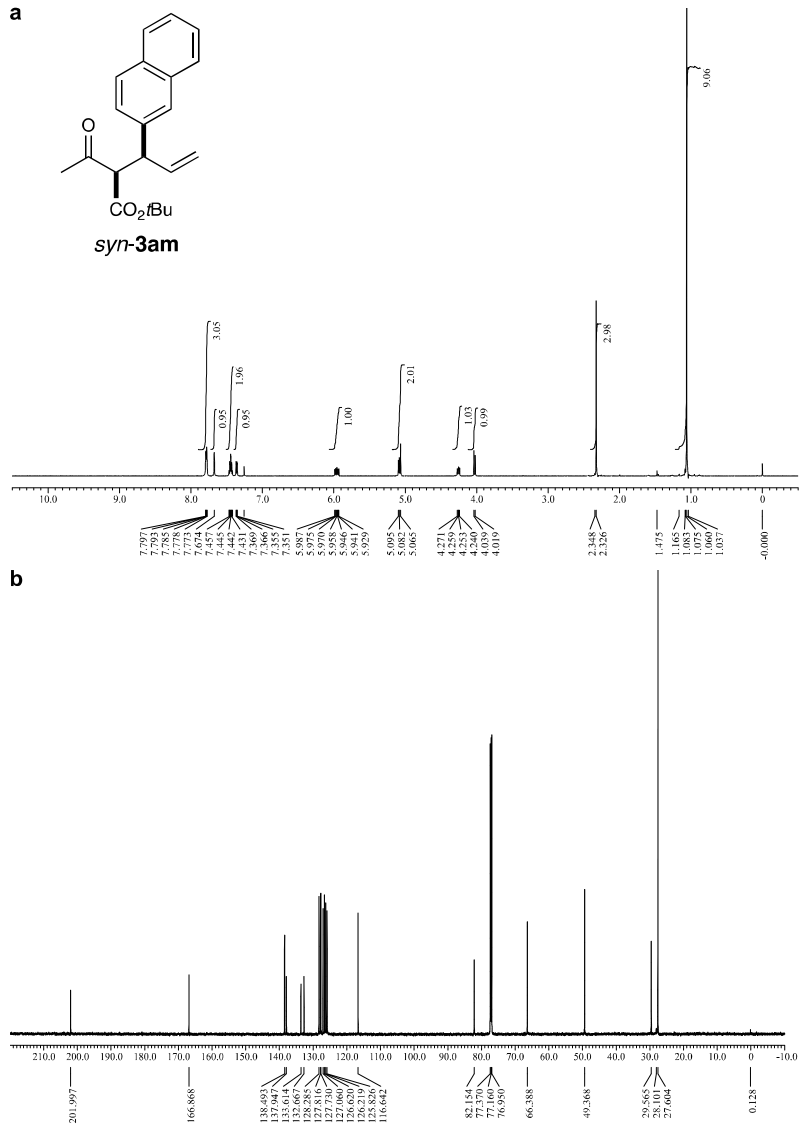

Supplementary Figure 78. ^{1}H-NMR (**a**) and ^{13}C-NMR (**b**) spectra of *tert*-butyl (2*R*,3*S*)-2-acetyl-3-(naphthalen-2-yl)pent-4-enoate (*syn*-**3am**) in CDCl3 (**Table 2**, entry 25).

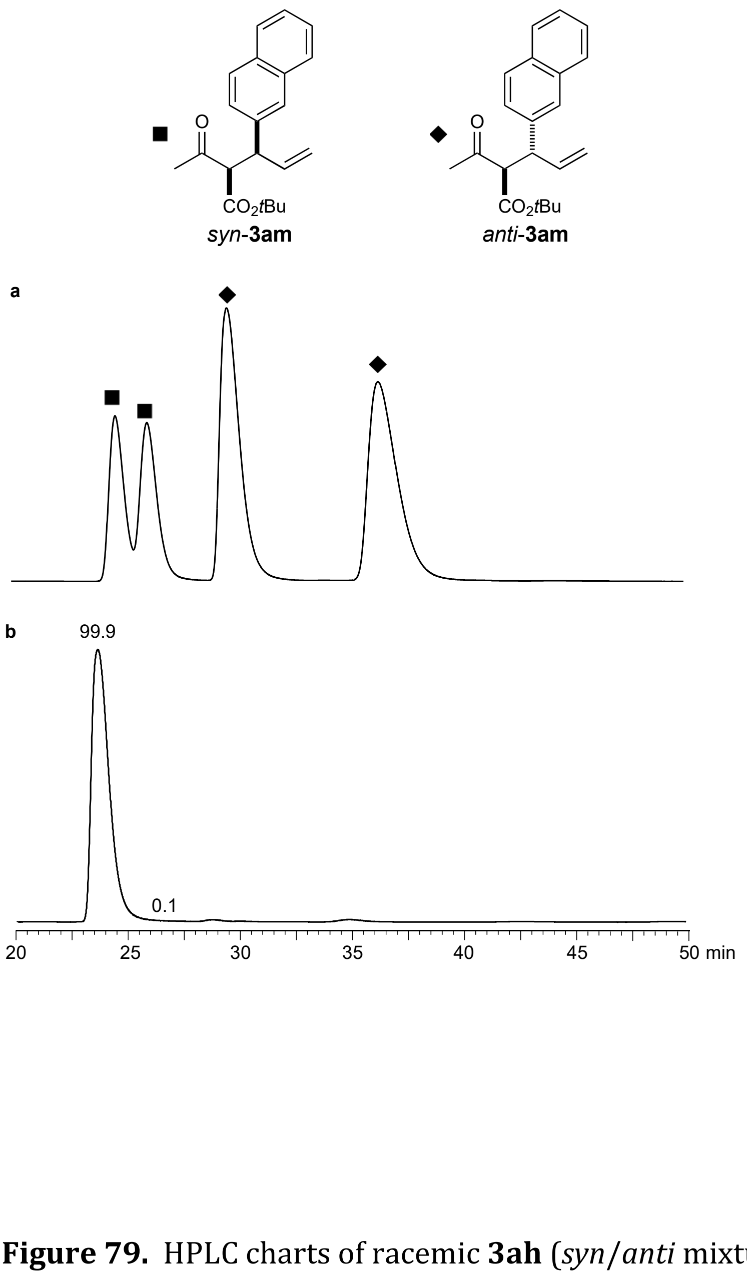

Supplementary Figure 79. HPLC charts of racemic **3ah** (*syn*/*anti* mixture) (**a**) and synthetic (**b**) *tert*-butyl (2*R*,3*S*)-2-acetyl-3-(4-fluorophenyl)pent-4-enoate (*syn*-**3ah**) (**Table 2**, entry 25). Conditions: column, CHIRALPAK ID-3; eluent, 1.0:99.0 2-PrOH–Hex; flow rate, 0.50 mL/min; detection, 220-nm light.

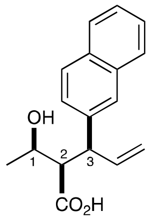

(1*R*,2*R*,3*S*)-*syn*$_{1,2}$*syn*$_{2,3}$-**11am**

a

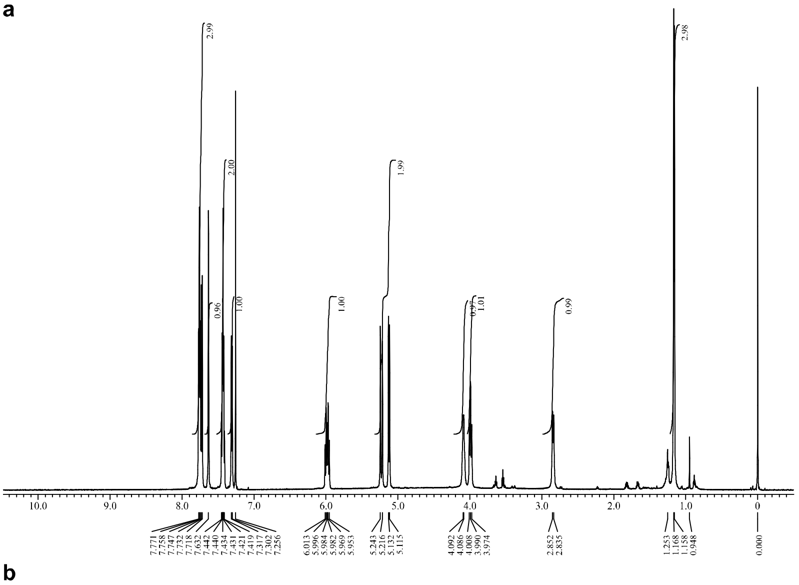

b

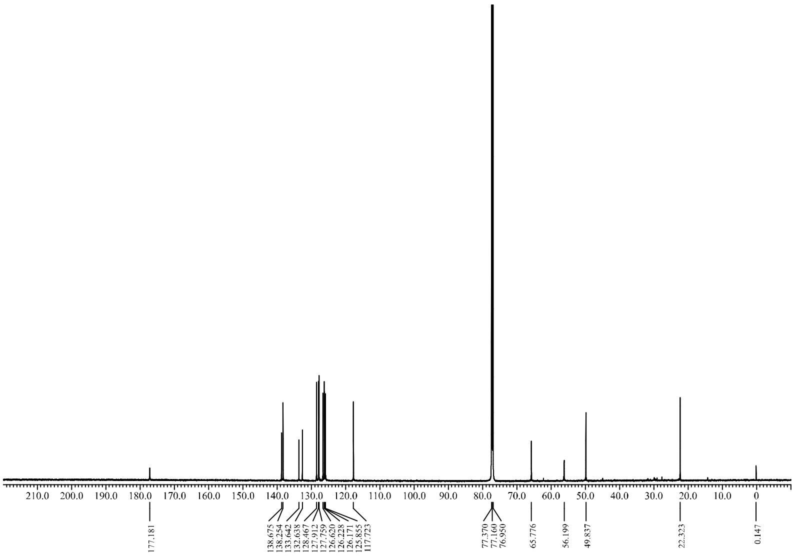

Supplementary Figure 80. ^{1}H-NMR (**a**) and ^{13}C-NMR (**b**) spectra of (2*R*,3*S*)-2-((*R*)-1-hydroxyethyl)-3-(naphthalen-2-yl)pent-4-enoic acid ((1*R*,2*R*,3*S*)-*syn*$_{1,2}$*syn*$_{2,3}$-**11am**) in $CDCl_3$.

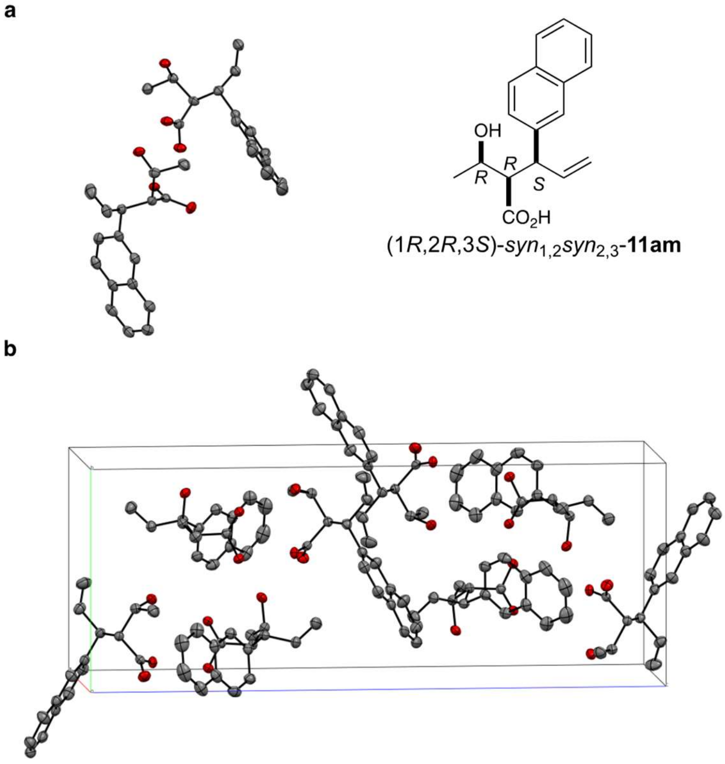

Supplementary Figure 81. ORTEP drawing (**a**) and packing diagram (**b**) of (2*R*,3*S*)-2-((*R*)-1-hydroxyethyl)-3-(naphthalen-2-yl)pent-4-enoic acid ((1*R*,2*R*,3*S*)-*syn*$_{1,2}$*syn*$_{2,3}$-**11am**).

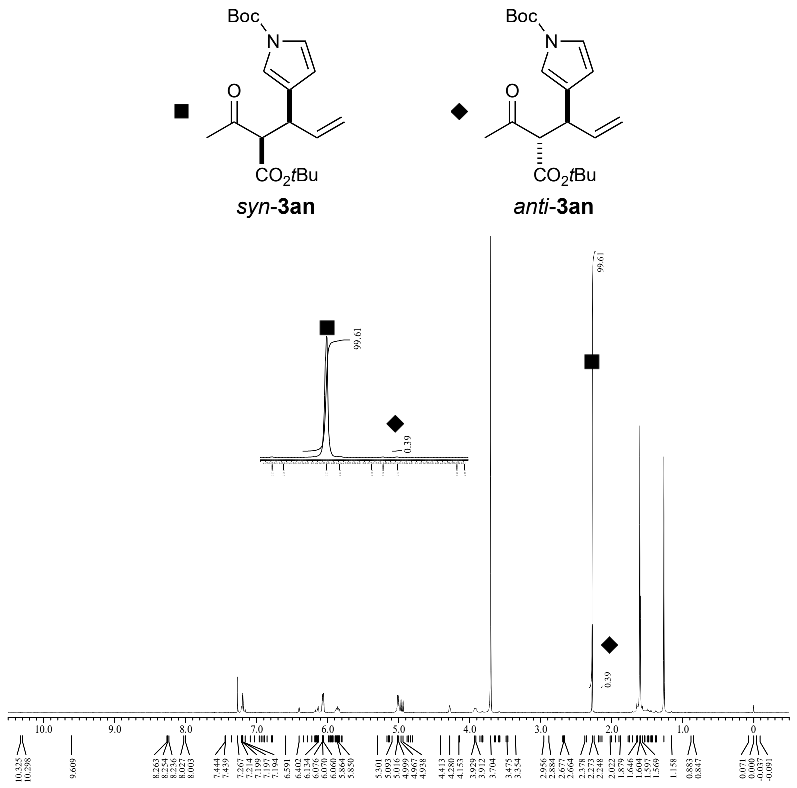

Supplementary Figure 82. ^{1}H-NMR spectrum of 0.500-mmol scale reaction mixture for synthesis of *syn*-**3an** in $CDCl_3$. Repetition time: 10 sec.

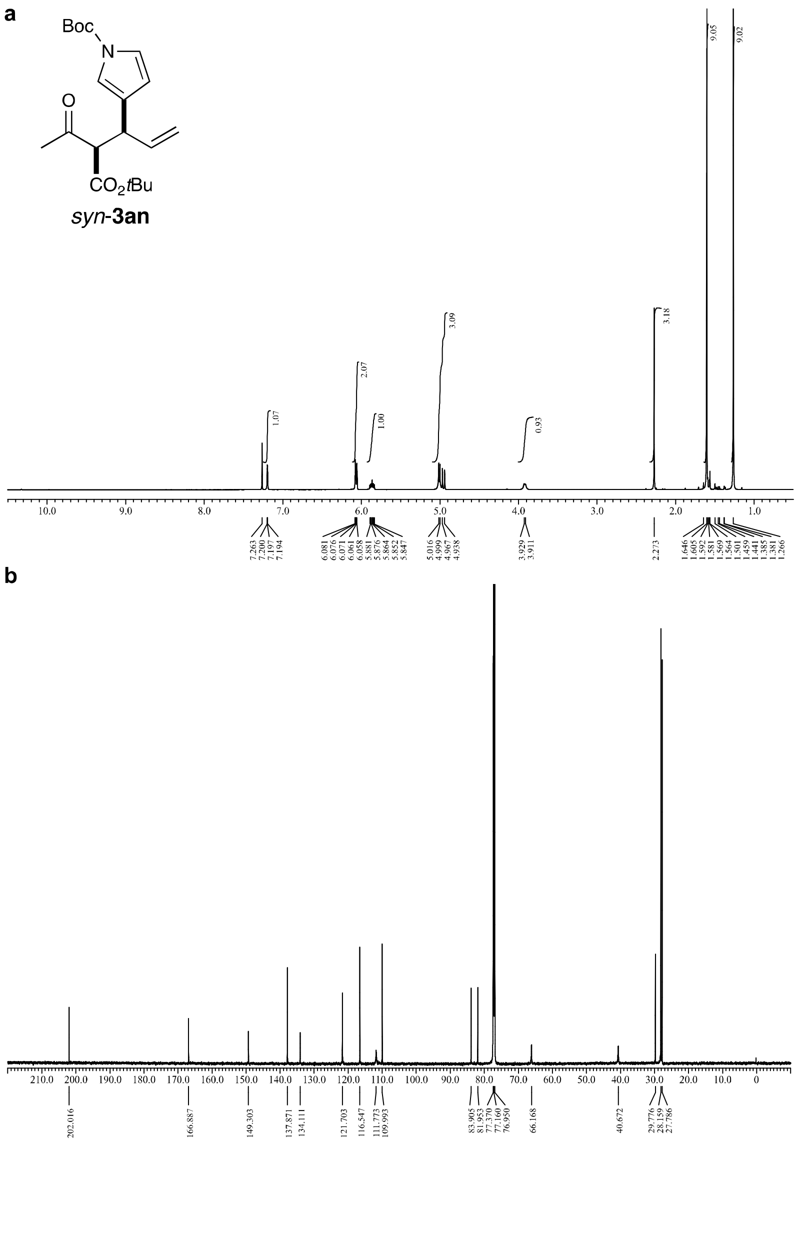

Supplementary Figure 83. ^{1}H-NMR (**a**) and ^{13}C-NMR (**b**) spectra of *tert*-butyl 3-((3*S*,4*R*)-4-(*tert*-butoxycarbonyl)-5-oxohex-1-en-3-yl)-1*H*-pyrrole-1-carboxylate (*syn*-**3an**) in $CDCl_3$ (**Table 2**, entry 26).

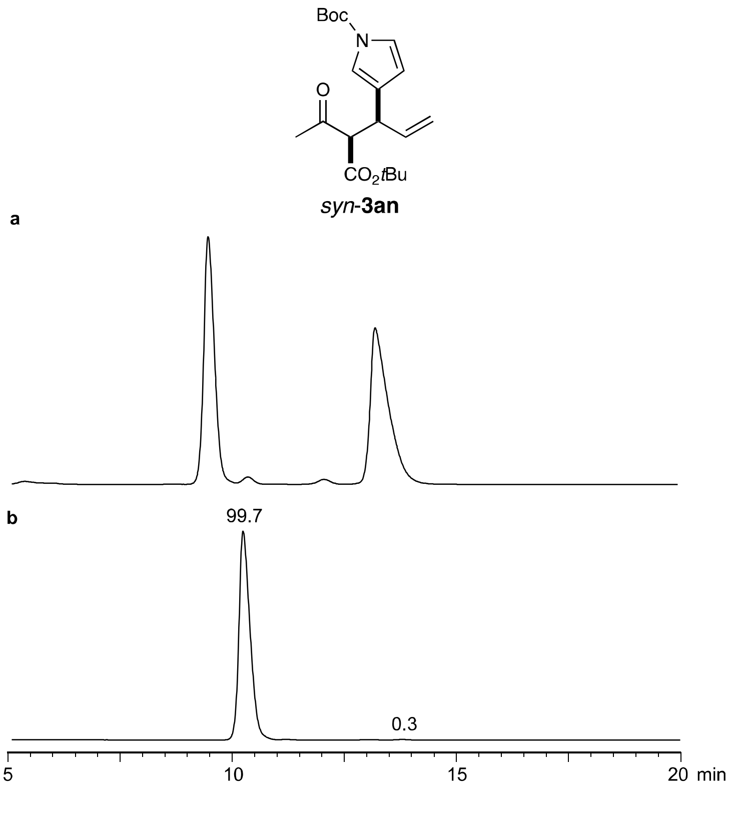

Supplementary Figure 84. HPLC charts of racemic (**a**) and synthetic (**b**) *tert*-butyl 3-((3*S*,4*R*)-4-(*tert*-butoxycarbonyl)-5-oxohex-1-en-3-yl)-1*H*-pyrrole-1-carboxylate (*syn*-**3an**) (**Table 2**, entry 26). Conditions: column, CHIRALPAK IE-3; eluent, 1.0:99.0 2-PrOH–Hex; flow rate, 1.00 mL/min; detection, 220-nm light.

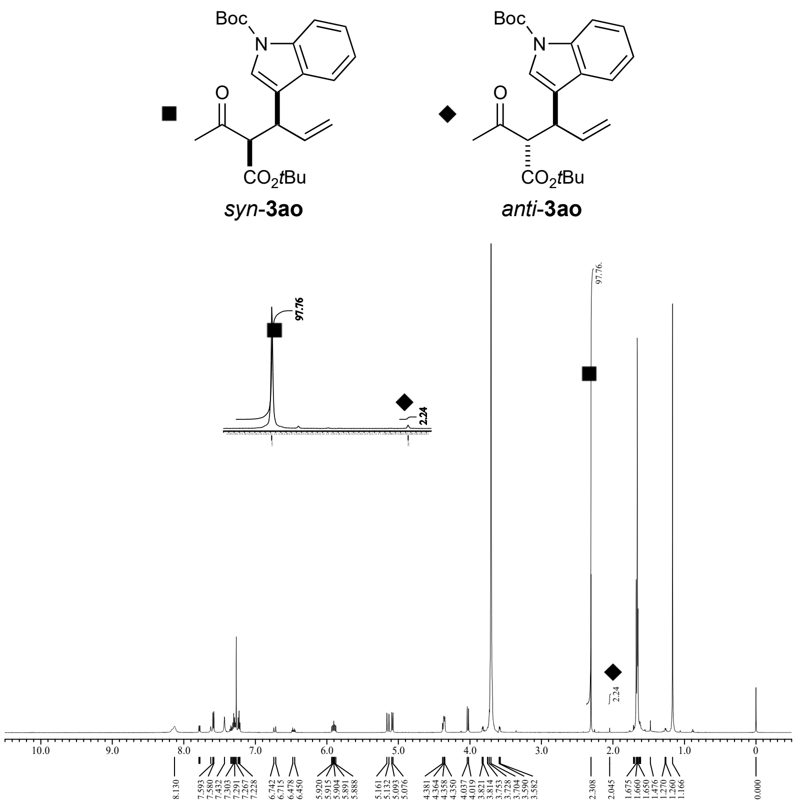

Supplementary Figure 85. ^{1}H-NMR spectrum of 0.500-mmol scale reaction mixture for synthesis of *syn*-**3ao** in $CDCl_3$. Repetition time: 10 sec.

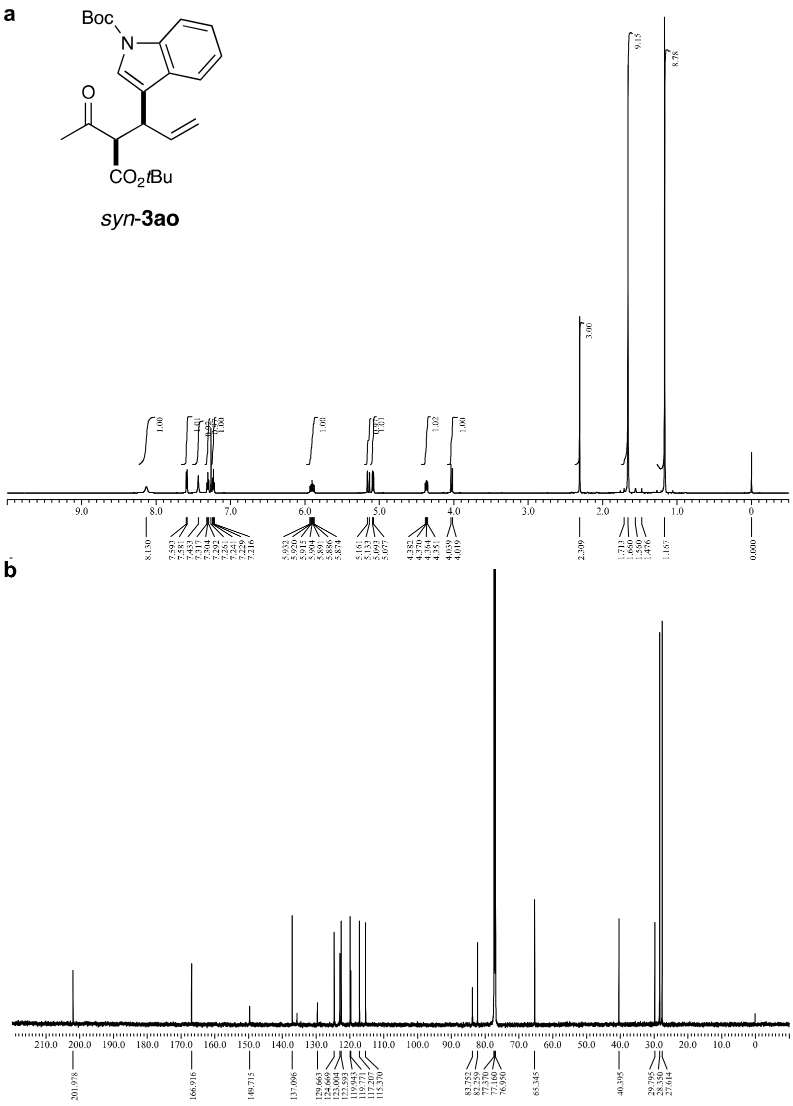

Supplementary Figure 86. ^{1}H-NMR (**a**) and ^{13}C-NMR (**b**) spectra of *tert*-butyl (α*R*,β*S*)-α-acetyl-β-vinyl-*N*-(*tert*-butoxycarbonyl)-1*H*-indole-3-propionate (*syn*-**3ao**) in $CDCl_3$ (**Table 2**, entry 27).

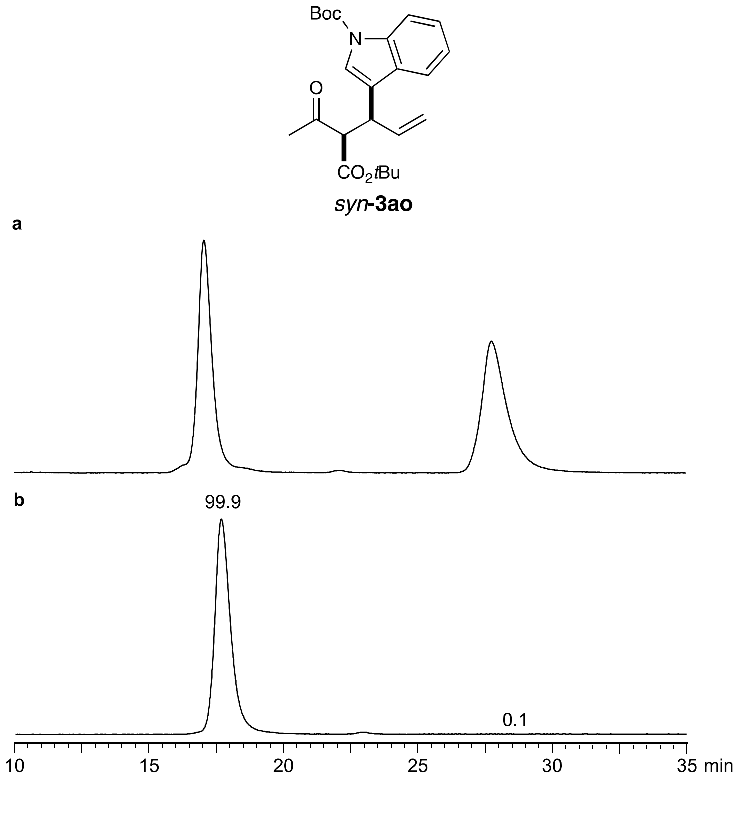

Supplementary Figure 87. HPLC charts of racemic (**a**) and synthetic (**b**) *tert*-butyl (α*R*,β*S*)-α-acetyl-β-vinyl-*N*-(*tert*-butoxycarbonyl)-1*H*-indole-3-propionate (*syn*-**3ao**) (**Table 2**, entry 27). Conditions: column, CHIRALPAK IE-3; eluent, 1.00:99.0 2-PrOH–Hex; flow rate, 1.00 mL/min; detection, 220-nm light.

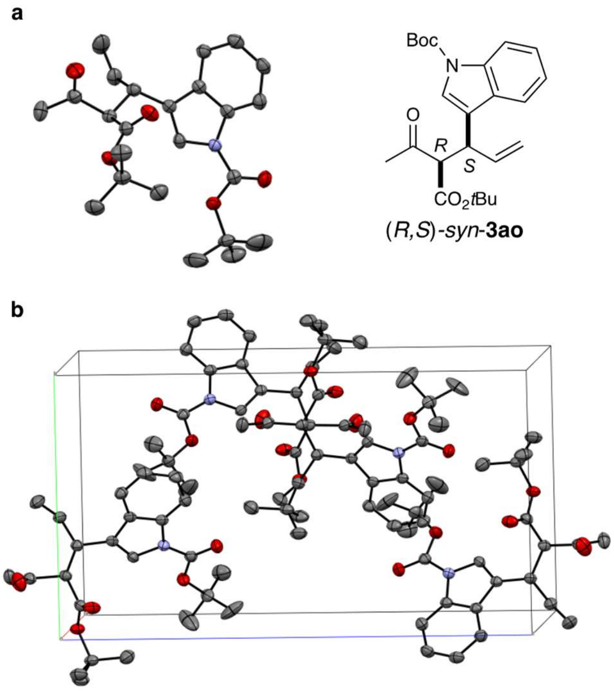

Supplementary Figure 88. ORTEP drawing (**a**) and packing diagram (**b**) of *tert*-butyl (αR,βS)-α-acetyl-β-vinyl-*N*-(*tert*-butoxycarbonyl)-1*H*-indole-3-propionate (*syn*-**3ao**).

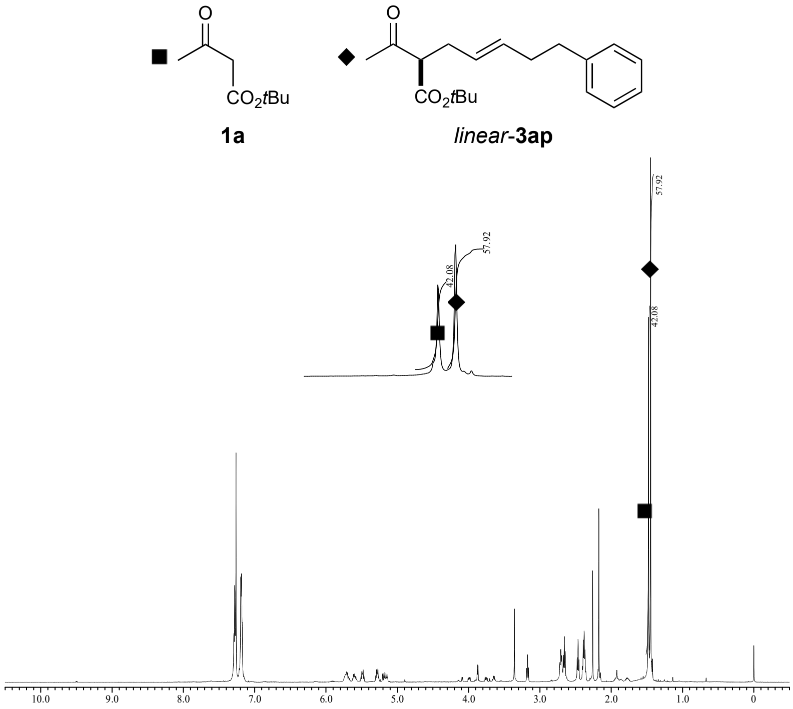

Supplementary Figure 89. ^{1}H-NMR spectrum of 0.500-mmol scale reaction mixture for synthesis of *linear*-**3ap** in $CDCl_3$. Repetition time: 10 sec.

a

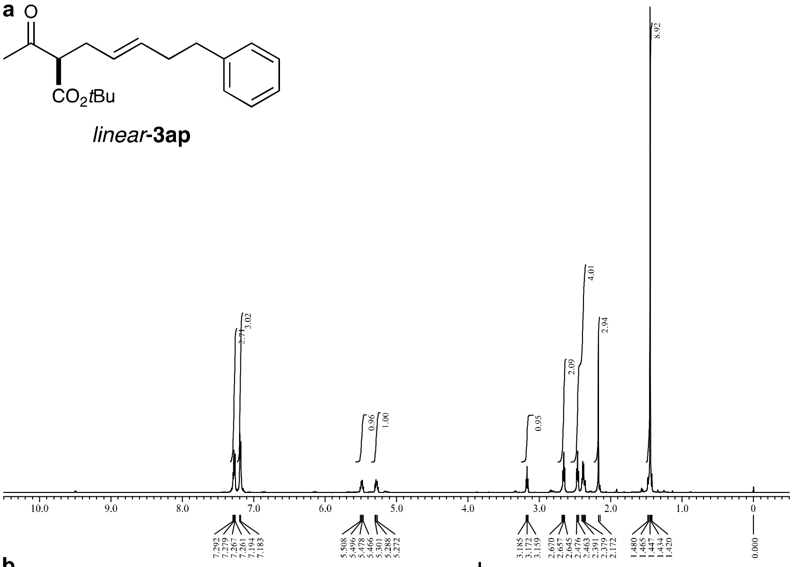

b

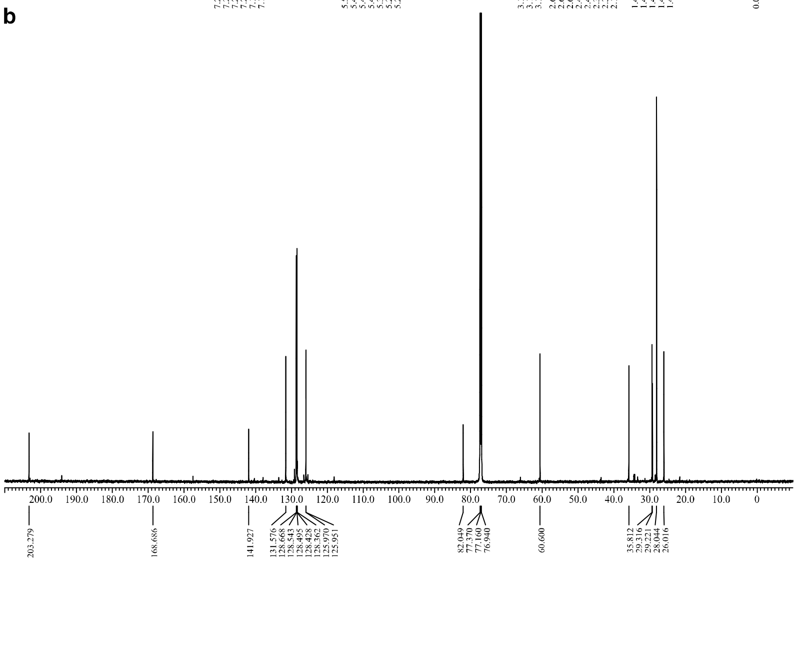

Supplementary Figure 90. ^{1}H-NMR (**a**) and ^{13}C-NMR (**b**) spectra of *tert*-butyl (*R*,*E*)-2-acetyl-7-phenylhept-4-enoate (*linear*-**3ap**) in $CDCl_3$ (**Table 2**, entry 28).

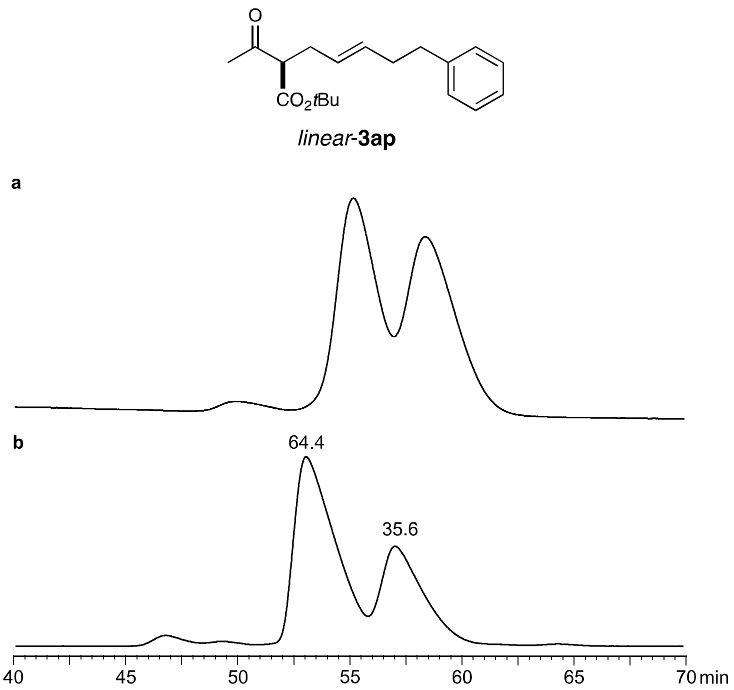

Supplementary Figure 91. HPLC charts of racemic (**a**) and synthetic (**b**) *tert*-butyl (*R*,*E*)-2-acetyl-7-phenylhept-4-enoate (*linear*-**3ap**)(**Table 2**, entry 28). Conditions: column, CHIRALPAK IE-3; eluent, 0.5:99.5 2-PrOH–Hex; flow rate, 0.25 mL/min; detection, 220-nm light.

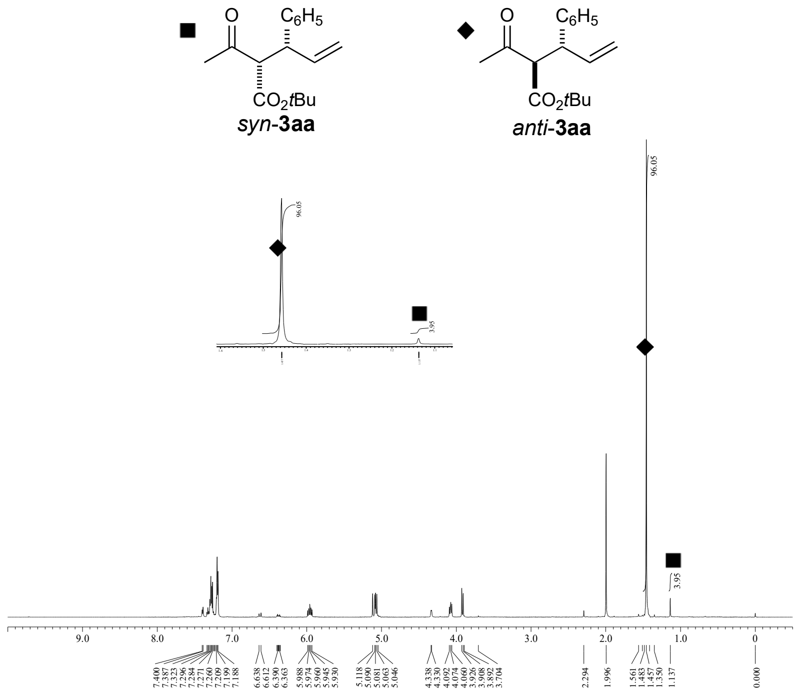

Supplementary Figure 92. ^{1}H-NMR spectrum of 0.500-mmol scale reaction mixture for synthesis of *anti*-**3aa** in $CDCl_3$. Repetition time: 10 sec.

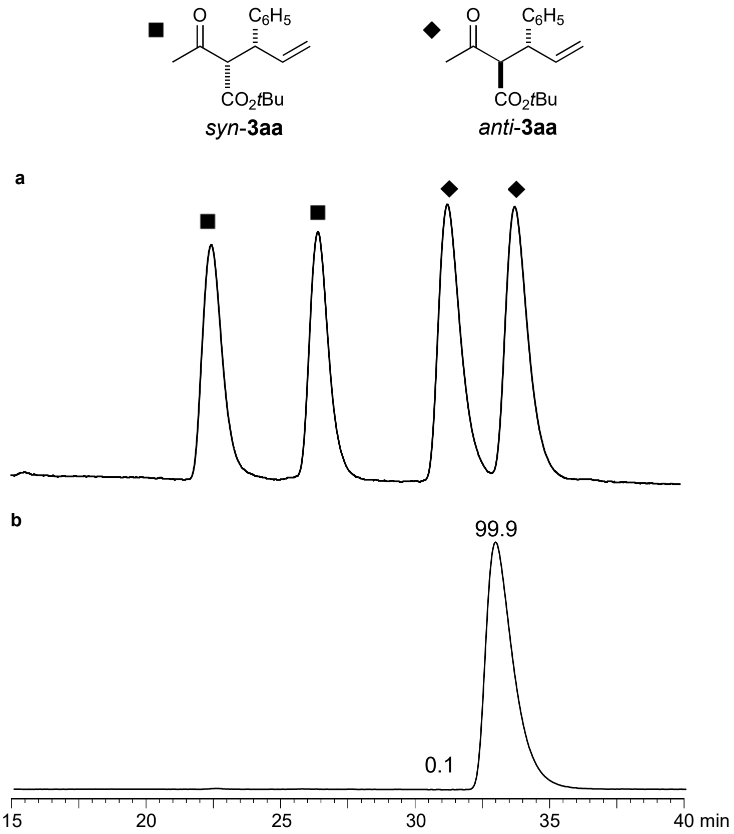

Supplementary Figure 93. HPLC charts of racemic **3aa** (*syn*/*anti* mixture) (**a**) and synthetic (**b**) *tert*-butyl (2*R*,3*R*)-2-acetyl-3-phenylpent-4-enoate (*anti*-**3aa**) (**Table 3**, entry 1). Conditions: column, CHIRALPAK ID-3; eluent, 1.0:99.0 2-PrOH–Hex; flow rate, 0.50 mL/min; detection, 220-nm light.

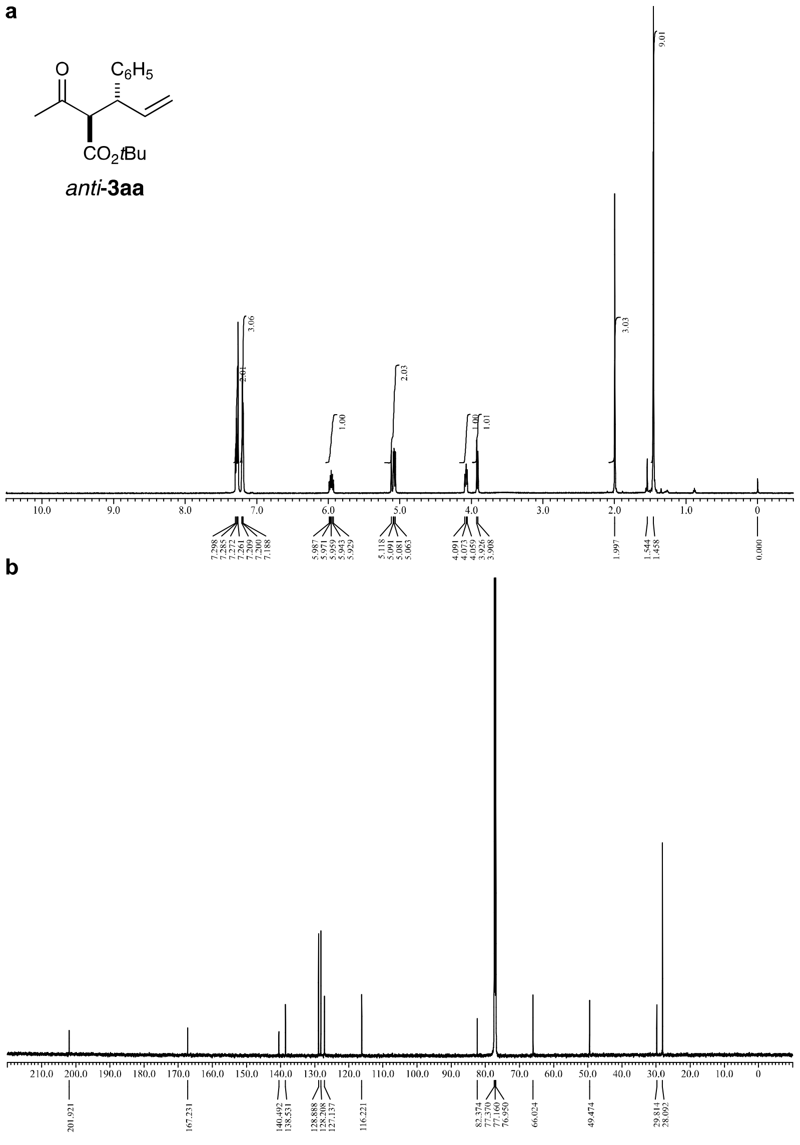

Supplementary Figure 94. ^{1}H-NMR (**a**) and ^{13}C-NMR (**b**) spectra of *tert*-butyl (2*R*,3*R*)-2-acetyl-3-phenylpent-4-enoate (*anti*-**3aa**) in $CDCl_3$ (**Table 3**, entry 1).

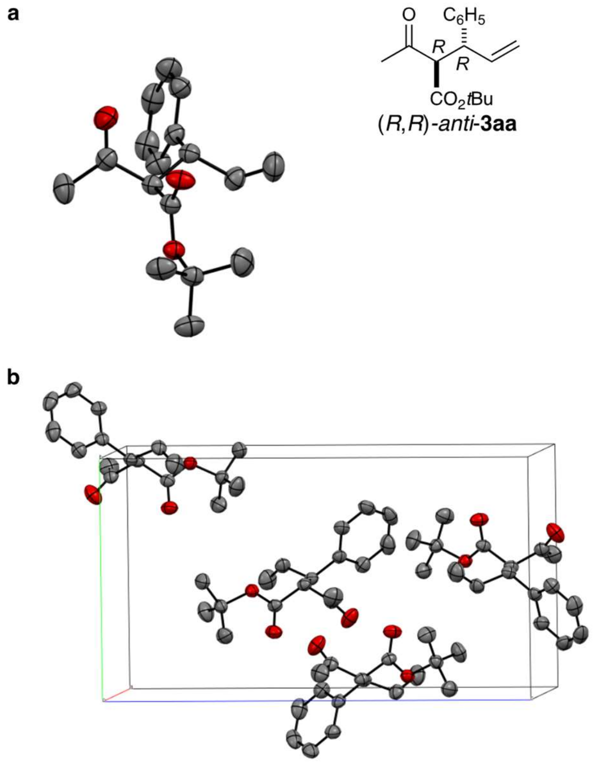

Supplementary Figure 95. ORTEP drawing (**a**) and packing diagram (**b**) of *tert*-butyl (2*R*,3*R*)-2-acetyl-3-phenylpent-4-enoate (*anti*-**3aa**).

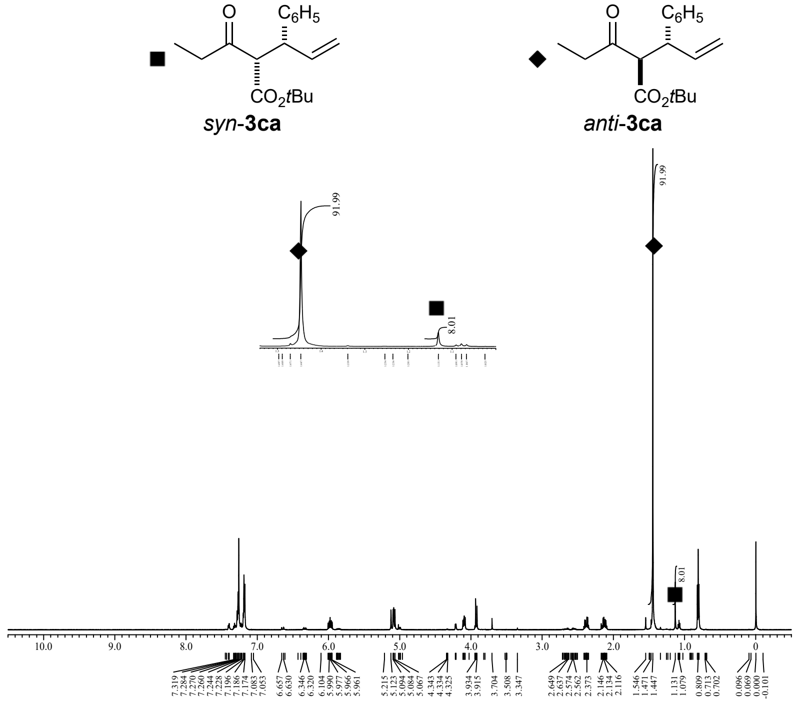

Supplementary Figure 96. ^{1}H-NMR spectrum of 0.500-mmol scale reaction mixture for synthesis of *anti*-**3ca** in $CDCl_3$. Repetition time: 10 sec.

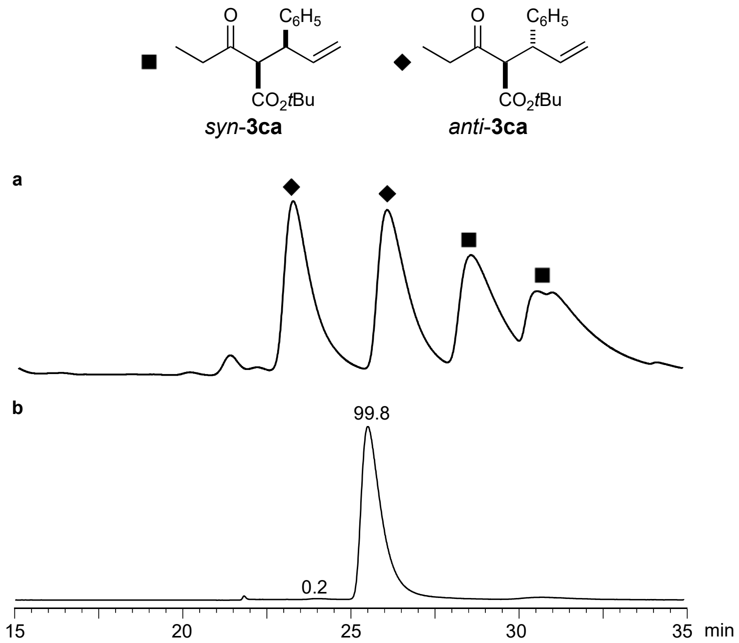

Supplementary Figure 97. HPLC charts of racemic **3ca** (*syn*/*anti* mixture) (**a**) and synthetic (**b**) *tert*-butyl (2*R*,3*R*)-3-phenyl-2-propionylpent-4-enoate (*anti*-**3ca**) (**Table 3**, entry 2). Conditions: column, CHIRALPAK ID-3; eluent, 0.5:99.5 2-PrOH–Hex; flow rate, 0.50 mL/min; detection, 220-nm light.

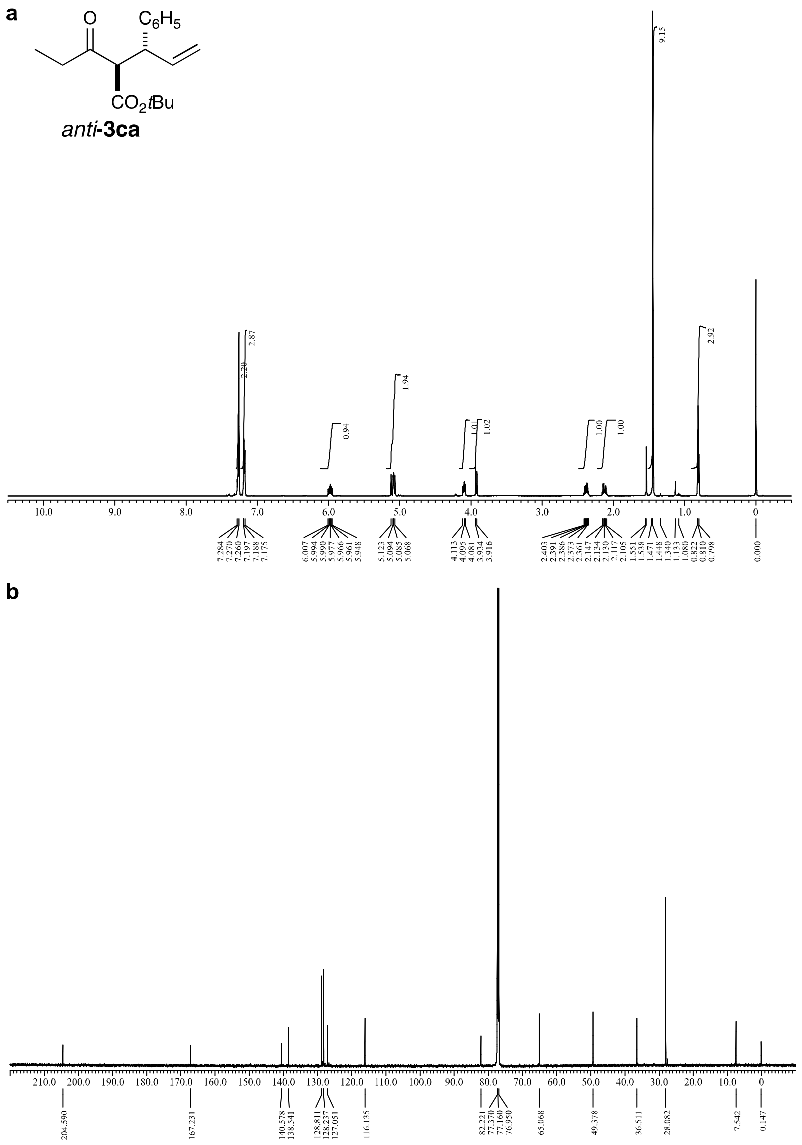

Supplementary Figure 98. ^{1}H-NMR (**a**) and ^{13}C-NMR (**b**) spectra of *tert*-butyl (2*R*,3*R*)-3-phenyl-2-propionylpent-4-enoate (*anti*-**3ca**) in $CDCl_3$ (**Table 3**, entry 2).

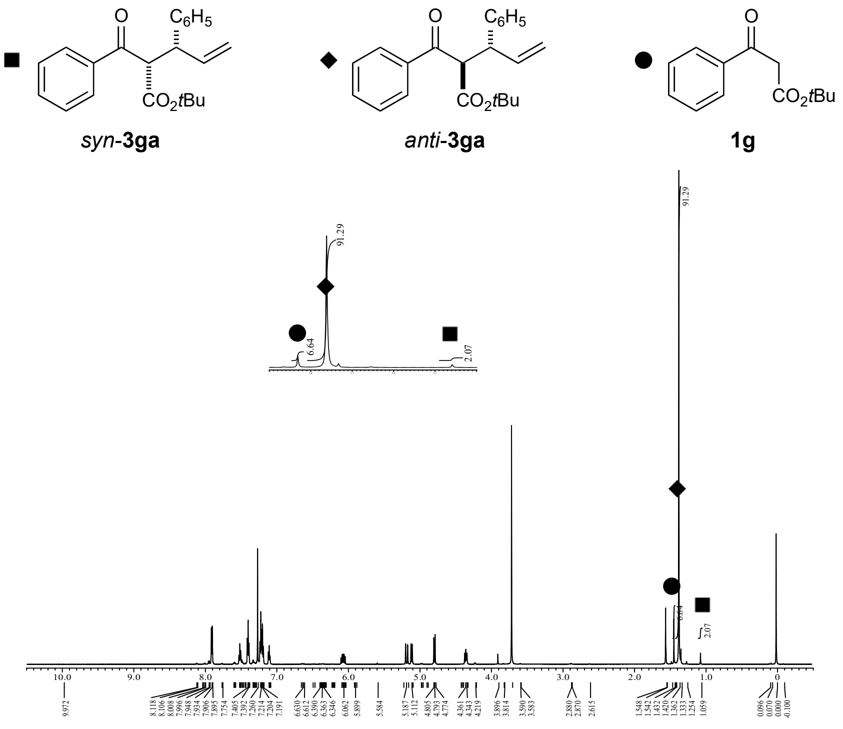

Supplementary Figure 99. ^{1}H-NMR spectrum of 0.500-mmol scale reaction mixture for synthesis of *anti*-**3ga** in $CDCl_3$. Repetition time: 10 sec.

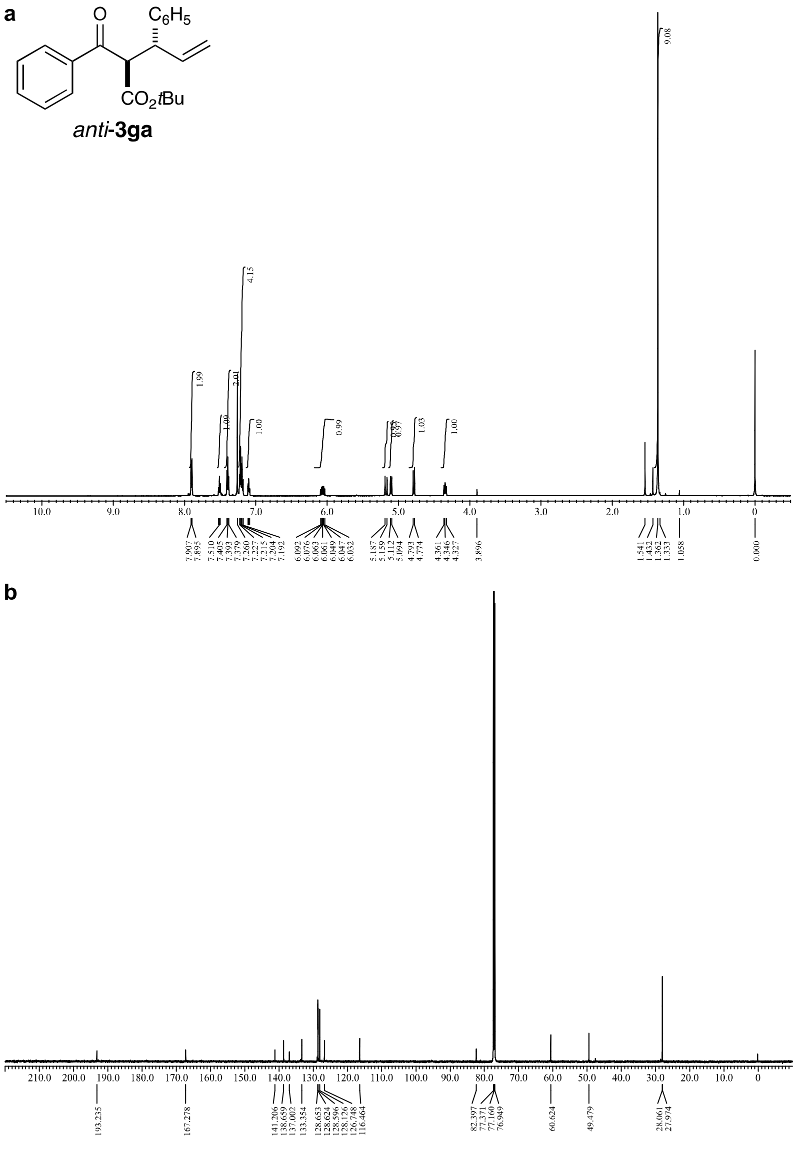

Supplementary Figure 100. ^{1}H-NMR (**a**) and ^{13}C-NMR (**b**) spectra of *tert*-butyl (2*R*,3*R*)-2-benzoyl-3-phenylpent-4-enoate (*anti*-**3ga**) in $CDCl_3$ (**Table 3**, entry 4).

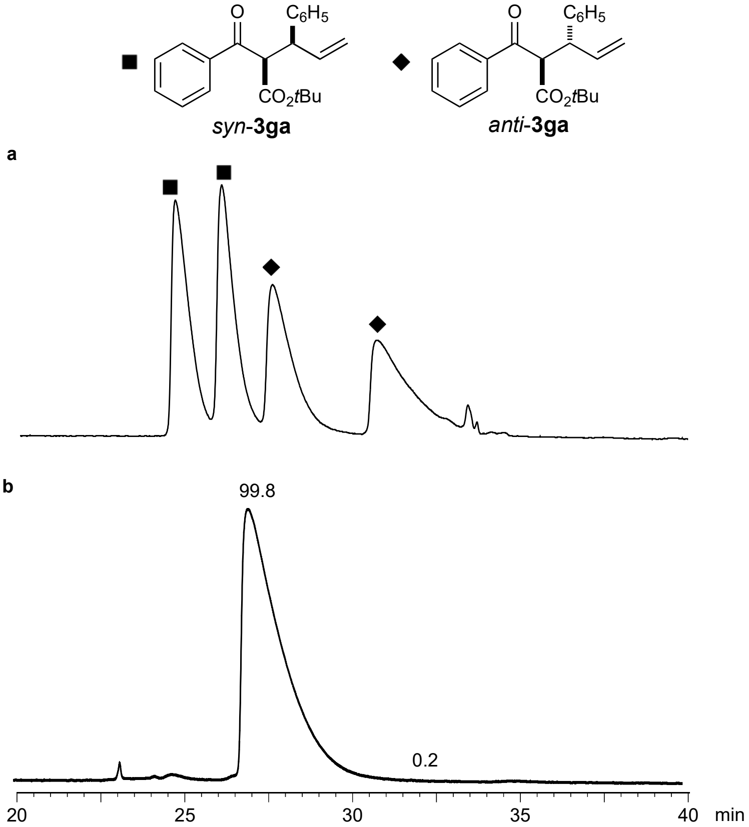

Supplementary Figure 101. HPLC charts of racemic **3ga** (*syn*/*anti* mixture) (**a**) and synthetic (**b**) *tert*-butyl (2*R*,3*R*)-3-phenyl-2-propionylpent-4-enoate (*anti*-**3ga**) (**Table 3**, entry 4). Conditions: column, CHIRALPAK IB and CHIRALPAK IB-3; eluent, 0.2:99.8 2-PrOH–Hex; flow rate, 1.00 mL/min; detection, 220-nm light.

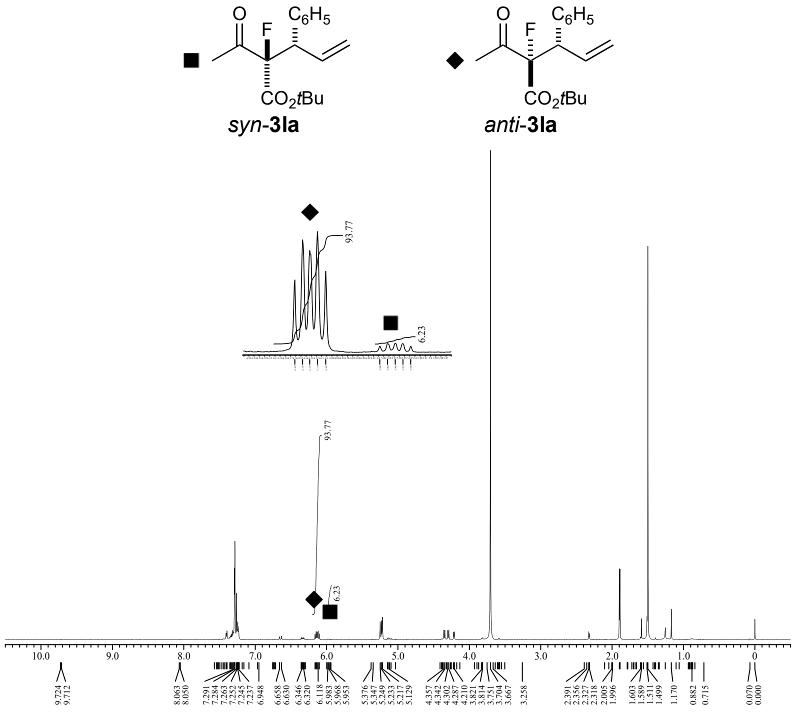

Supplementary Figure 102. ^{1}H-NMR spectrum of 0.500-mmol scale reaction mixture for synthesis of *anti*-**3la** in $CDCl_3$. Repetition time: 10 sec.

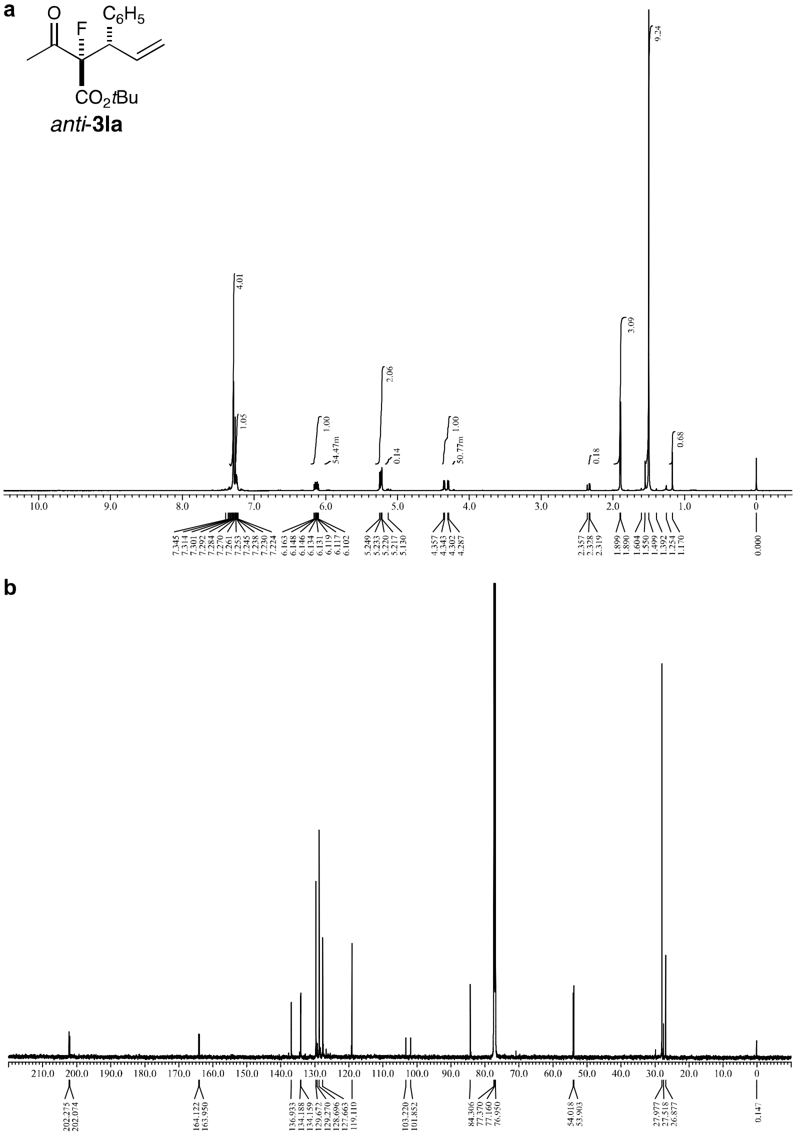

Supplementary Figure 103. ^{1}H-NMR (**a**) and ^{13}C-NMR (**b**) spectra of *tert*-butyl (2*S*,3*S*)-2-acetyl-2-fluoro-3-phenylpent-4-enoate (*anti*-**3la**) in $CDCl_3$ (**Table 3**, entry 5).

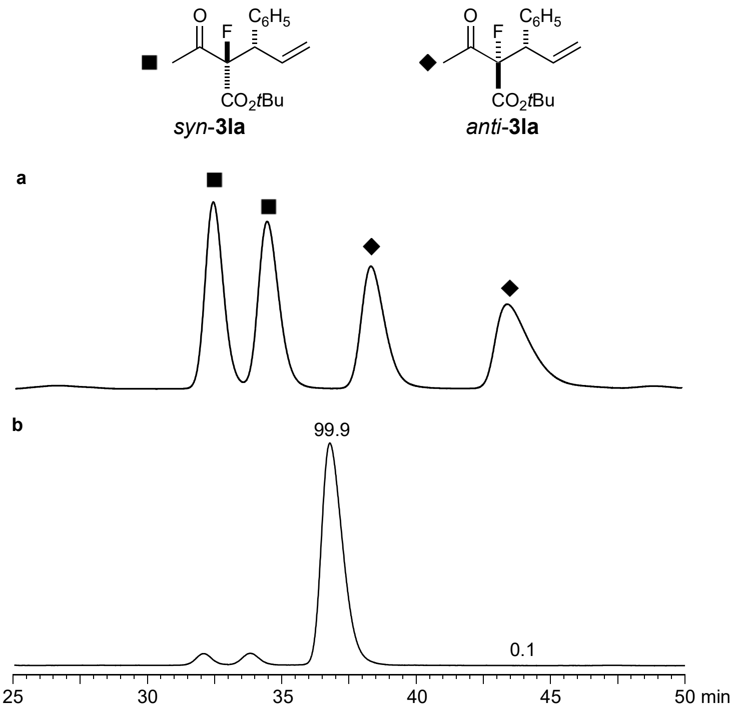

Supplementary Figure 104. HPLC charts of racemic **3la** (*syn*/*anti* mixture) (**a**) and synthetic (**b**) *tert*-butyl (2*S*,3*S*)-2-acetyl-2-fluoro-3-phenylpent-4-enoate (*anti*-**3la**) (**Table 3**, entry 5). Conditions: column, ϕ20 mm OD-H; eluent, 0.5:99.5 2-PrOH–Hex; flow rate, 3.00 mL/min; detection, 220-nm light.

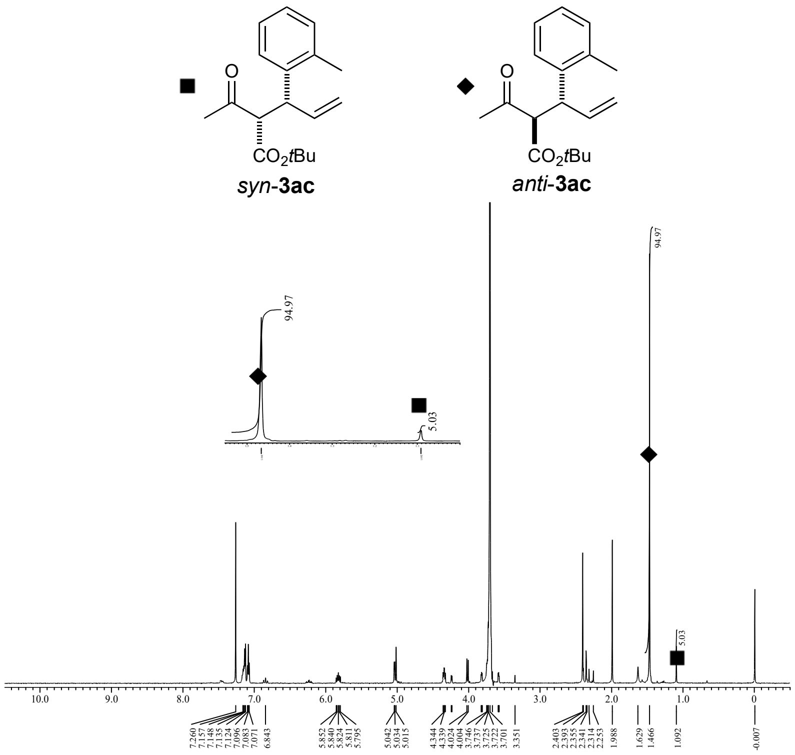

Supplementary Figure 105. ^{1}H-NMR spectrum of 0.500-mmol scale reaction mixture for synthesis of *anti*-**3ac** in $CDCl_3$. Repetition time: 10 sec.

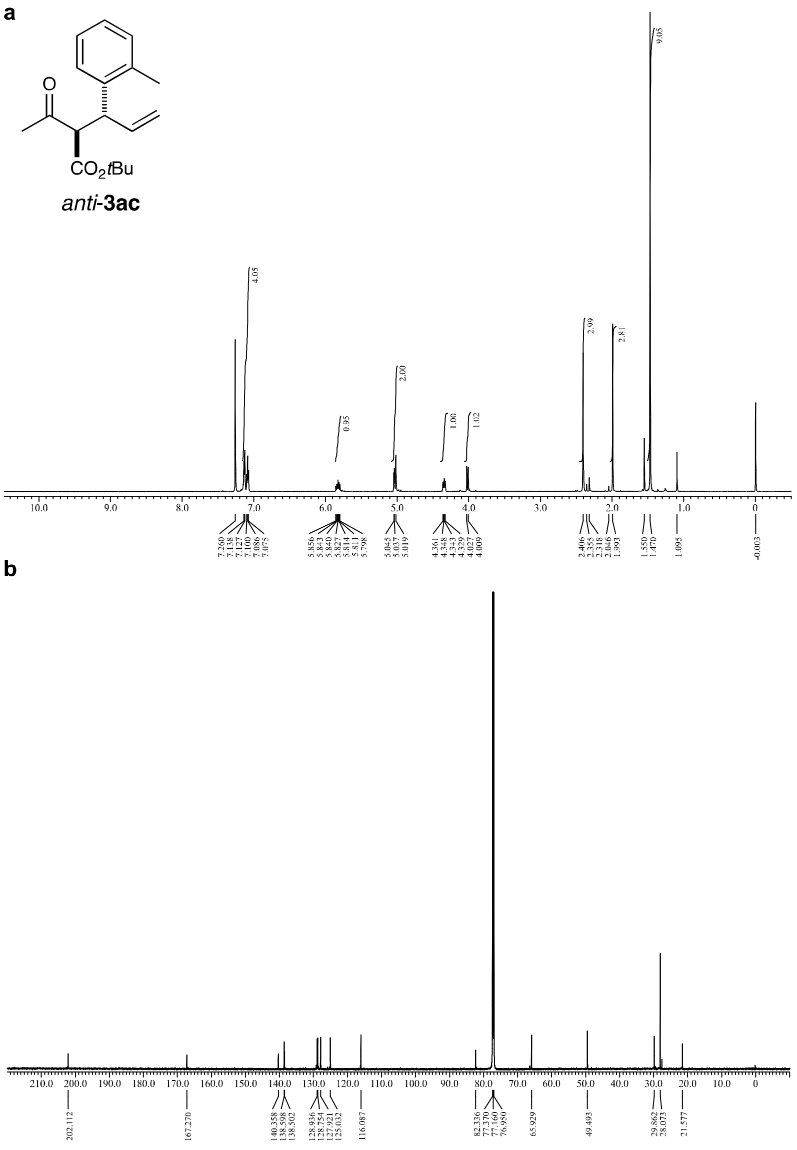

Supplementary Figure 106. ^{1}H-NMR (**a**) and ^{13}C-NMR (**b**) spectra of *tert*-butyl (2*R*,3*R*)-2-acetyl-3-(2-methylphenyl)pent-4-enoate (*anti*-**3ac**) in $CDCl_3$ (**Table 3**, entry 6).

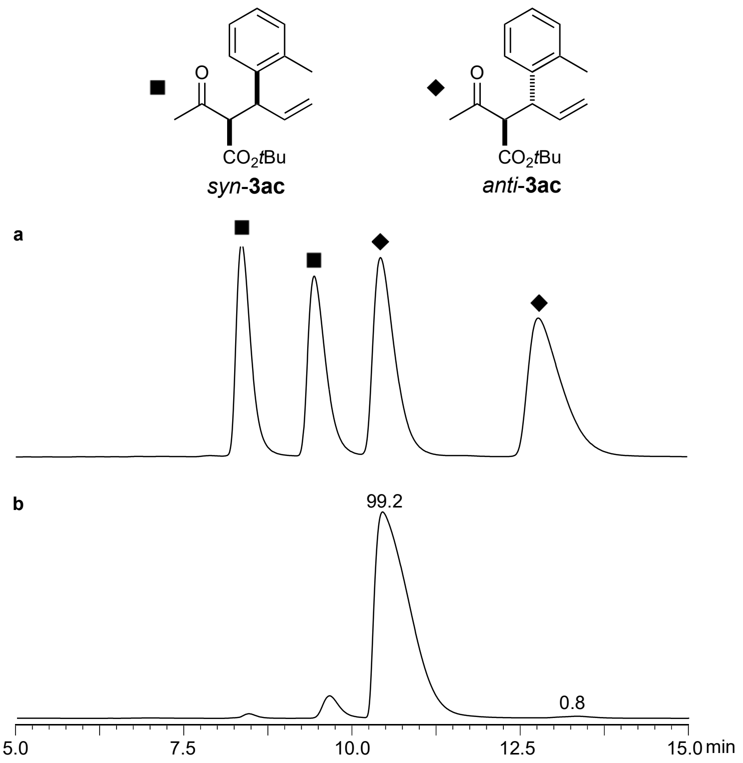

Supplementary Figure 107. HPLC charts of racemic **3ac** (*syn*/*anti* mixture) (**a**) and synthetic (**b**) *tert*-butyl (2*R*,3*R*)-2-acetyl-3-(2-methylphenyl)pent-4-enoate (*anti*-**3ac**) (**Table 3**, entry 6). Conditions: column, CHIRALPAK ID-3; eluent, 1.0:99.0 2-PrOH–Hex; flow rate, 1.00 mL/min; detection, 220-nm light.

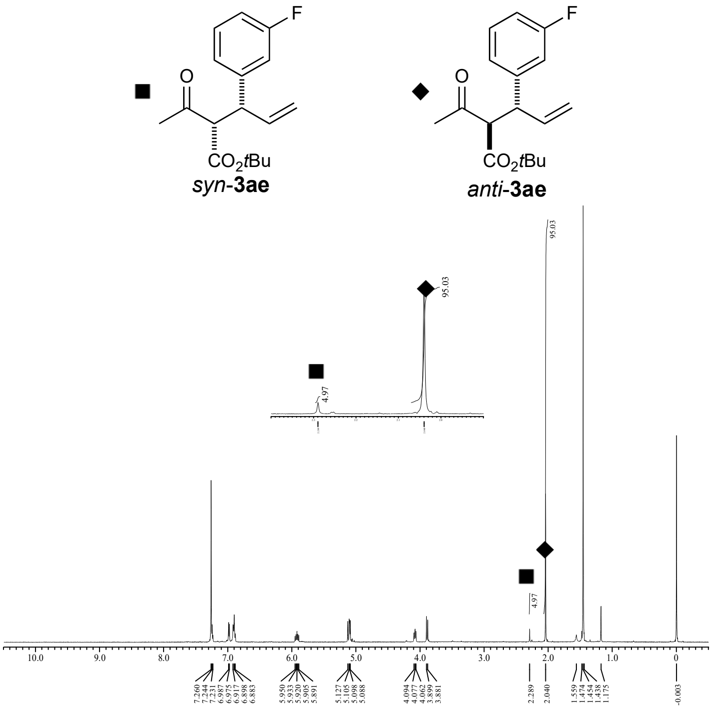

Supplementary Figure 108. ^{1}H-NMR spectrum of 0.500-mmol scale reaction mixture for synthesis of *anti*-**3ae** in $CDCl_3$. Repetition time: 10 sec.

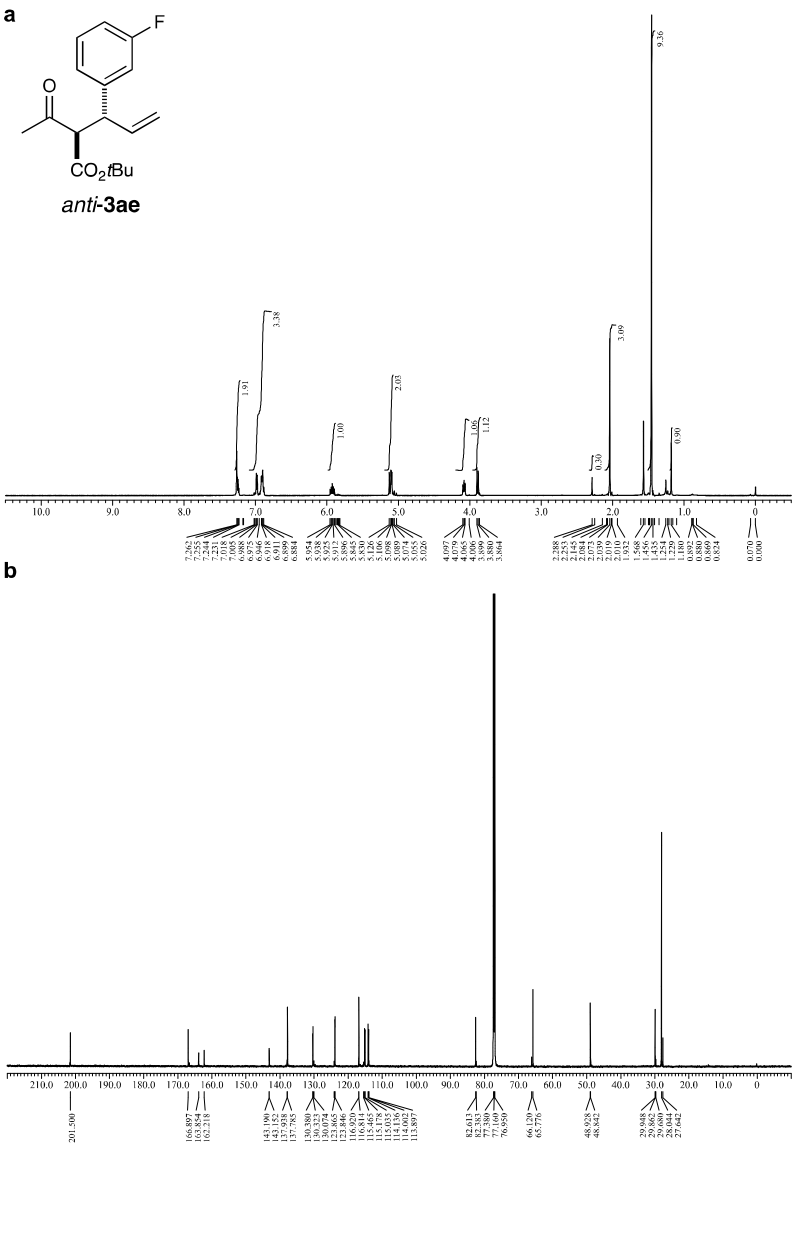

Supplementary Figure 109. ^{1}H-NMR (**a**) and ^{13}C-NMR (**b**) spectra of *tert*-butyl (2*R*,3*R*)-2-acetyl-3-(3-fluorophenyl)pent-4-enoate (*anti*-**3ae**) in $CDCl_3$ (**Table 3**, entry 7).

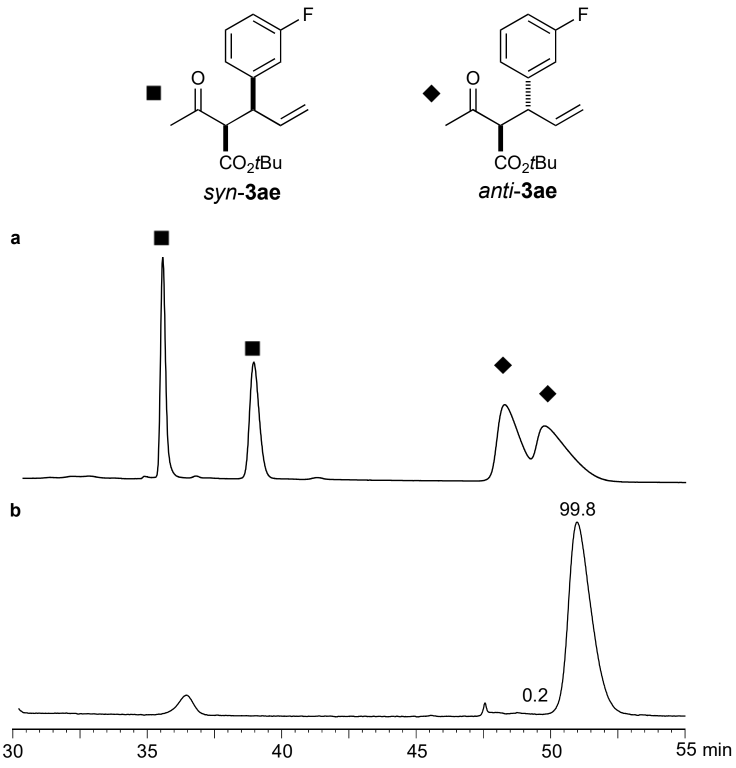

Supplementary Figure 110. HPLC charts of racemic **3ae** (*syn*/*anti* mixture) (**a**) and synthetic (**b**) *tert*-butyl (2*R*,3*R*)-2-acetyl-3-(3-fluorophenyl)pent-4-enoate (*anti*-**3ae**) (**Table 3**, entry 7). Conditions: column, CHIRALPAK IA and CHIRALPAK IA-3; eluent, 0.5:99.5 2-PrOH–Hex; flow rate, 0.50 mL/min; detection, 220-nm light.

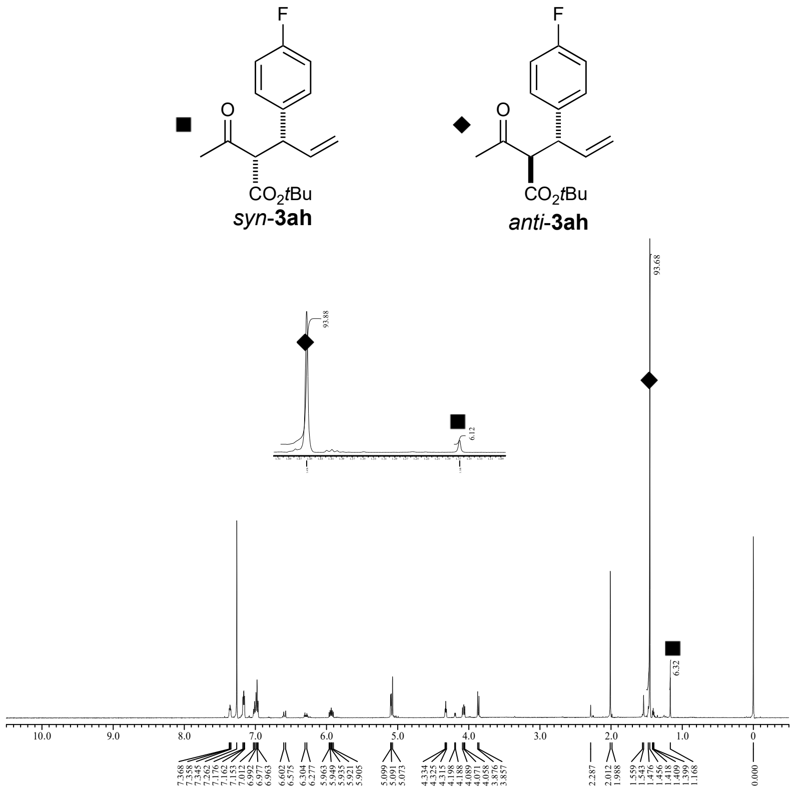

Supplementary Figure 111. ^{1}H-NMR spectrum of 0.500-mmol scale reaction mixture for synthesis of *anti*-**3ah** in $CDCl_3$. Repetition time: 10 sec.

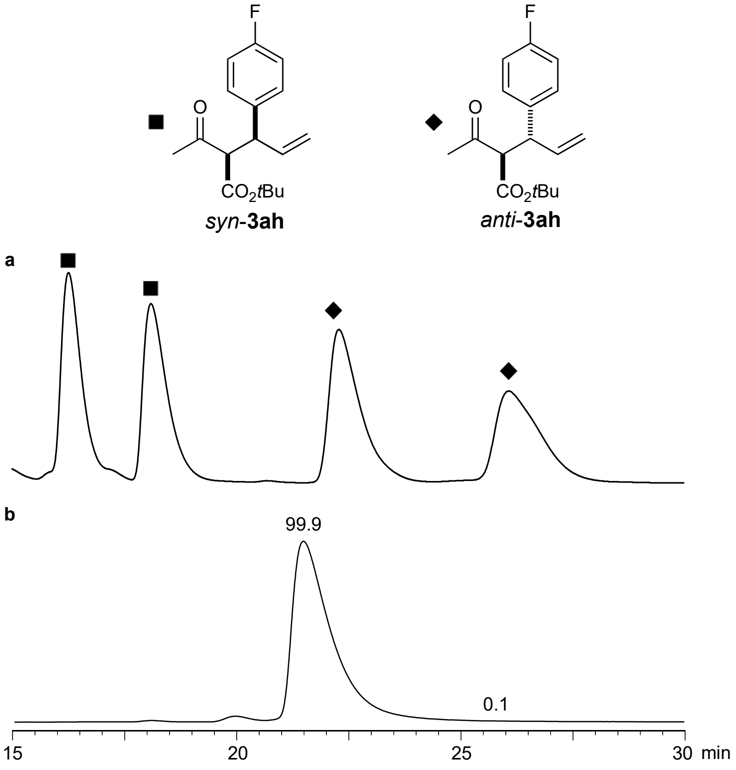

Supplementary Figure 112. HPLC charts of racemic **3ah** (*syn*/*anti* mixture) (**a**) and synthetic (**b**) *tert*-butyl (2*R*,3*R*)-2-acetyl-3-(4-fluorophenyl)pent-4-enoate (*anti*-**3ah**) (**Table 3**, entry 8). Conditions: column, CHIRALPAK ID-3; eluent, 1.0:99.0 2-PrOH–Hex; flow rate, 0.50 mL/min; detection, 220-nm light.

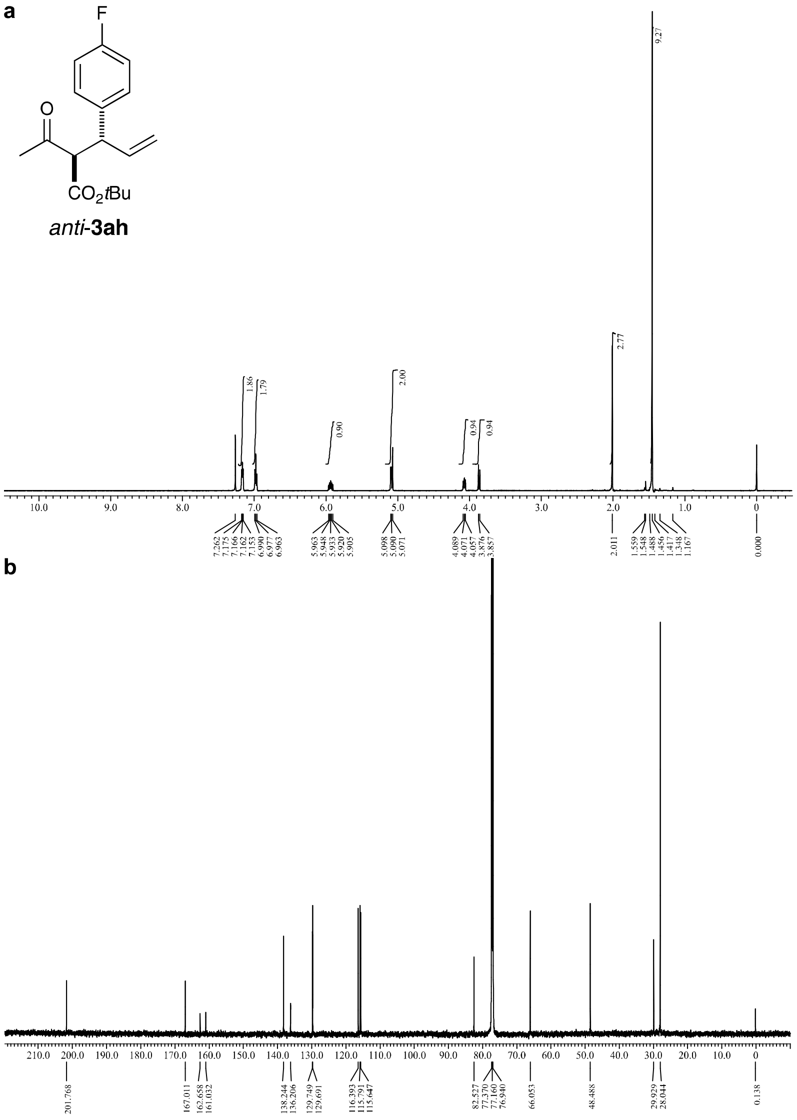

Supplementary Figure 113. ^{1}H-NMR (**a**) and ^{13}C-NMR (**b**) spectra of *tert*-butyl (2*R*,3*R*)-2-acetyl-3-(4-fluorophenyl)pent-4-enoate (*anti*-**3ah**) in $CDCl_3$ (**Table 3**, entry 8).

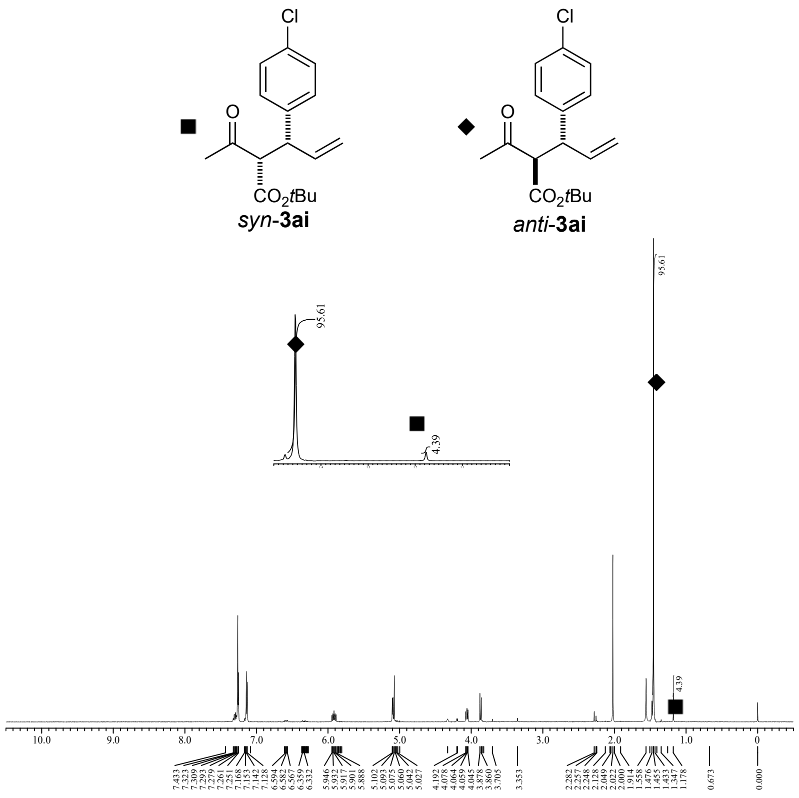

Supplementary Figure 114. ^{1}H-NMR spectrum of 0.500-mmol scale reaction mixture for synthesis of *anti*-**3ai** in $CDCl_3$. Repetition time: 10 sec.

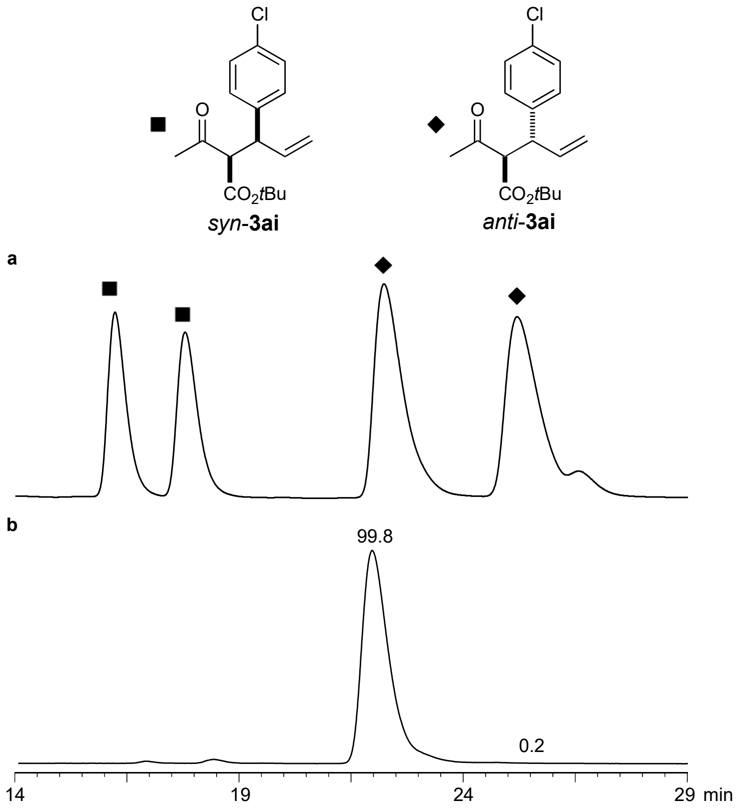

Supplementary Figure 115. HPLC charts of racemic **3ai** (*syn*/*anti* mixture) (**a**) and synthetic (**b**) *tert*-butyl (2*R*,3*R*)-2-acetyl-3-(4-chlorophenyl)pent-4-enoate (*anti*-**3ai**) (**Table 3**, entry 9). Conditions: column, CHIRALPAK ID-3; eluent, 1.0:99;0 2-PrOH–Hex; flow rate, 0.50 mL/min; detection, 220-nm light.

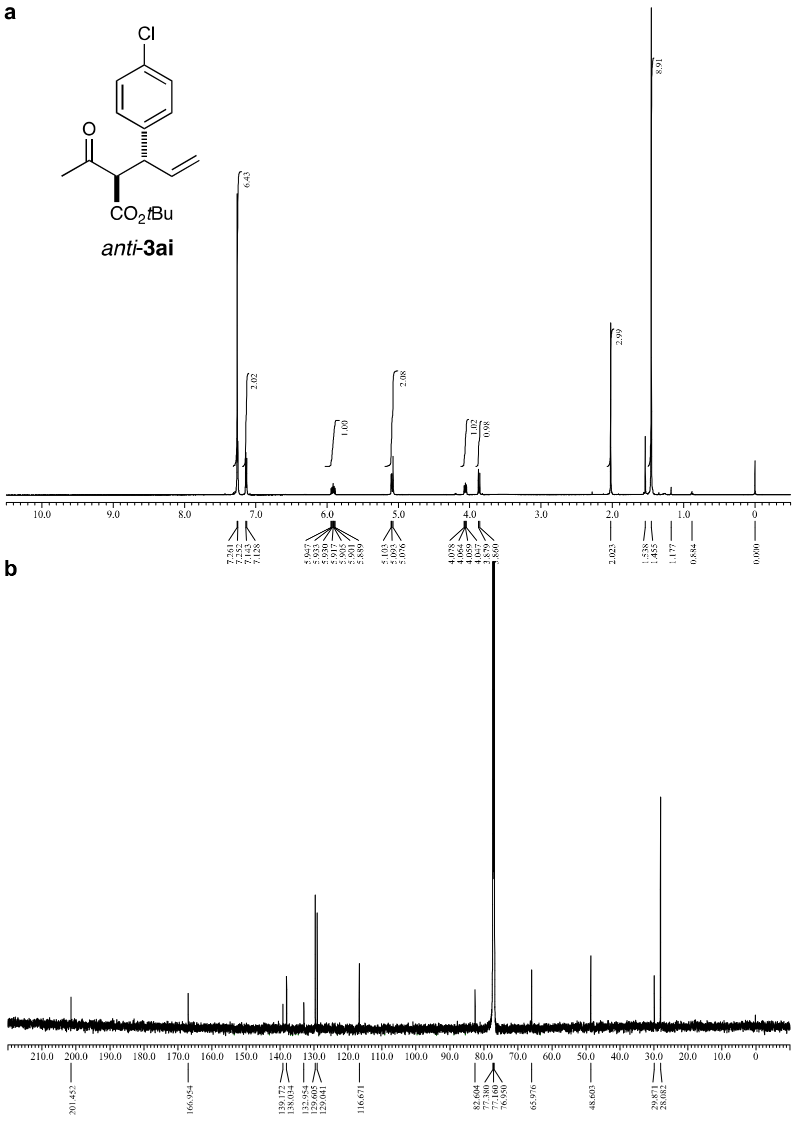

Supplementary Figure 116. ^{1}H-NMR (**a**) and ^{13}C-NMR (**b**) spectra of *tert*-butyl (2*R*,3*R*)-2-acetyl-3-(4-chlorophenyl)pent-4-enoate (*anti*-**3ai**) in $CDCl_3$ (**Table 3**, entry 9).

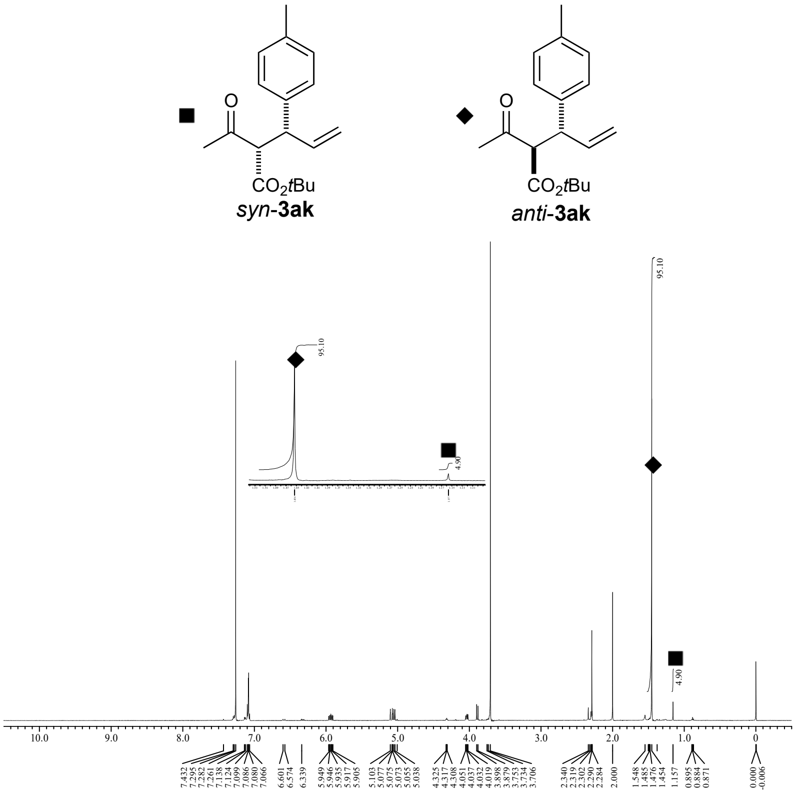

Supplementary Figure 117. ^{1}H-NMR spectrum of 0.500-mmol scale reaction mixture for synthesis of *anti*-**3ak** in $CDCl_3$. Repetition time: 10 sec.

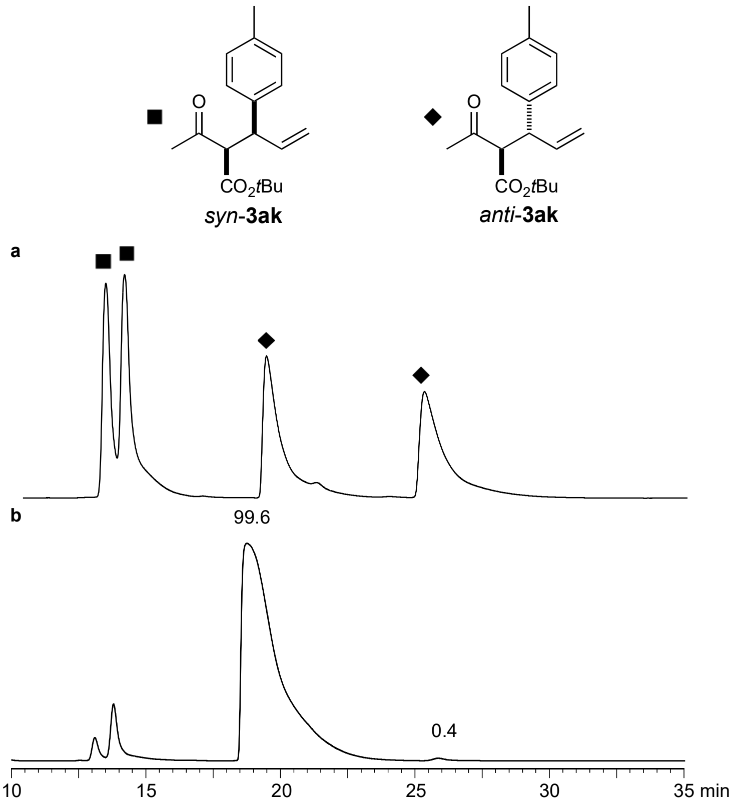

Supplementary Figure 118. HPLC charts of racemic **3ak** (*syn*/*anti* mixture) (**a**) and synthetic (**b**) *tert*-butyl (2*R*,3*R*)-2-acetyl-3-(4-methylphenyl)pent-4-enoate (*anti*-**3ak**) (**Table 3**, entry 10). Conditions: column, CHIRALPAK IA-3; eluent, 1.0:99.0 2-PrOH–Hex; flow rate, 0.50 mL/min; detection, 220-nm light.

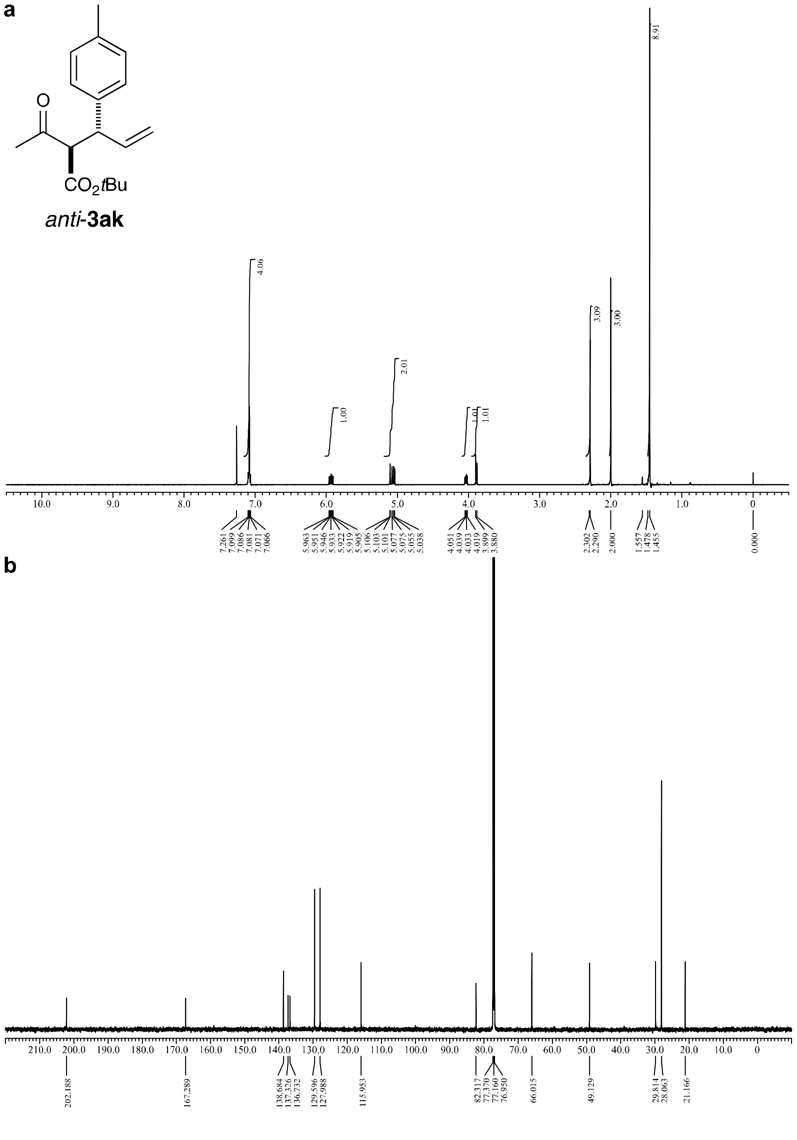

Supplementary Figure 119. ^{1}H-NMR (**a**) and ^{13}C-NMR (**b**) spectra of *tert*-butyl (2*R*,3*R*)-2-acetyl-3-(4-methylphenyl)pent-4-enoate (*anti*-**3ak**) in $CDCl_3$ (**Table 3**, entry 10).

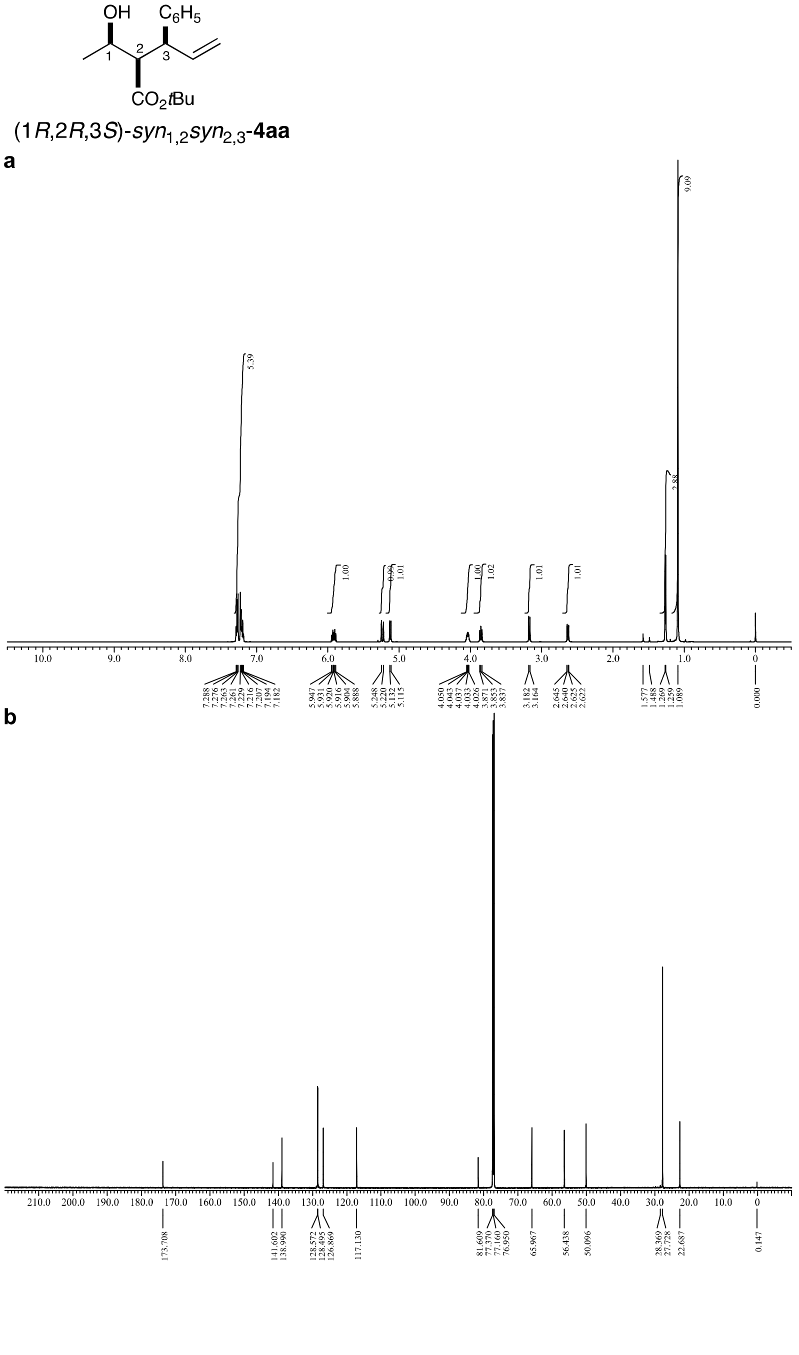

Supplementary Figure 120. ^{1}H-NMR (**a**) and ^{13}C-NMR (**b**) spectra of *tert*-butyl (2*R*,3*S*)-2-((*R*)-1-hydroxyethyl)-3-phenylpent-4-enoate ((1*R*,2*R*,3*S*)-*syn*$_{1,2}$*syn*$_{2,3}$-**4aa**) in $CDCl_3$ (**Figure 4**).

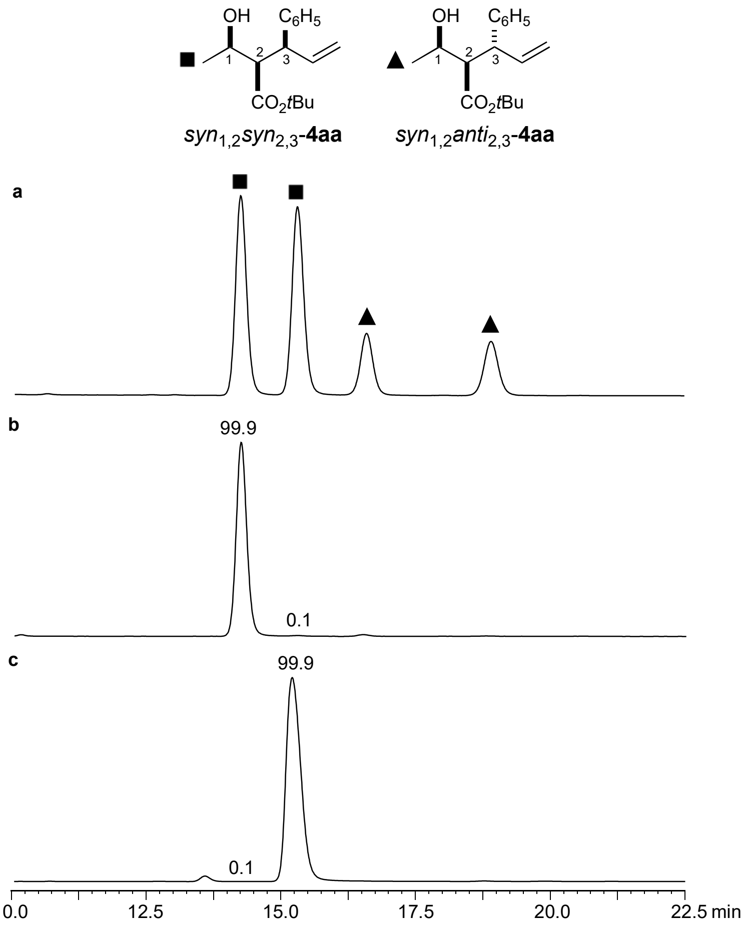

Supplementary Figure 121. HPLC charts of racemic (**a**), synthetic (**b**) *tert*-butyl (2*R*,3*S*)-2-((*R*)-1-hydroxyethyl)-3-phenylpent-4-enoate ((1*R*,2*R*,3*S*)-*syn*$_{1,2}$*syn*$_{2,3}$-**4aa**) and synthetic (**c**) *tert*-butyl (2*S*,3*R*)-2-((*S*)-1-hydroxyethyl)-3-phenylpent-4-enoate ((1*S*,2*S*,3*R*)-*syn*$_{1,2}$*syn*$_{2,3}$-**4aa**) (**Figure 4**). Conditions: column, CHIRALPAK IA-3; eluent, 2.0:98.0 2-PrOH–Hex; flow rate, 0.50 mL/min; detection, 220-nm light.

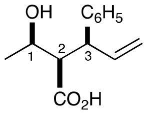

(1*R*,2*R*,3*S*)-*syn*$_{1,2}$*syn*$_{2,3}$-**11aa**

a

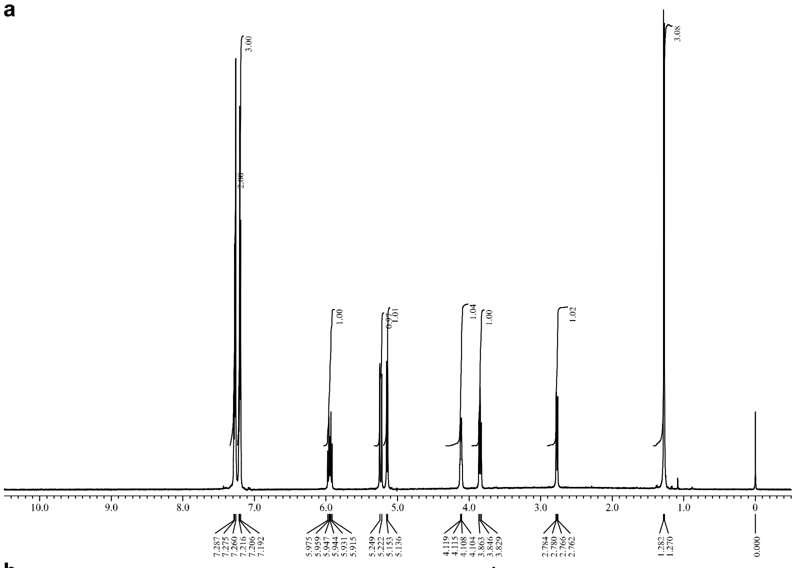

b

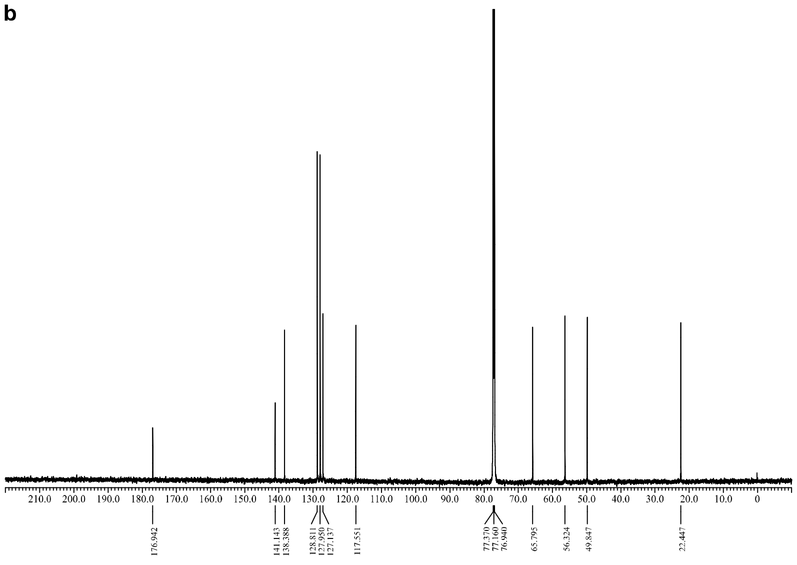

Supplementary Figure 122. ^{1}H-NMR (**a**) and ^{13}C-NMR (**b**) spectra of (2*R*,3*S*)-2-((*R*)-1-hydroxyethyl)-3-phenylpent-4-enoic acid ((1*R*,2*R*,3*S*)-*syn*$_{1,2}$*syn*$_{2,3}$-**11aa**) in $CDCl_3$.

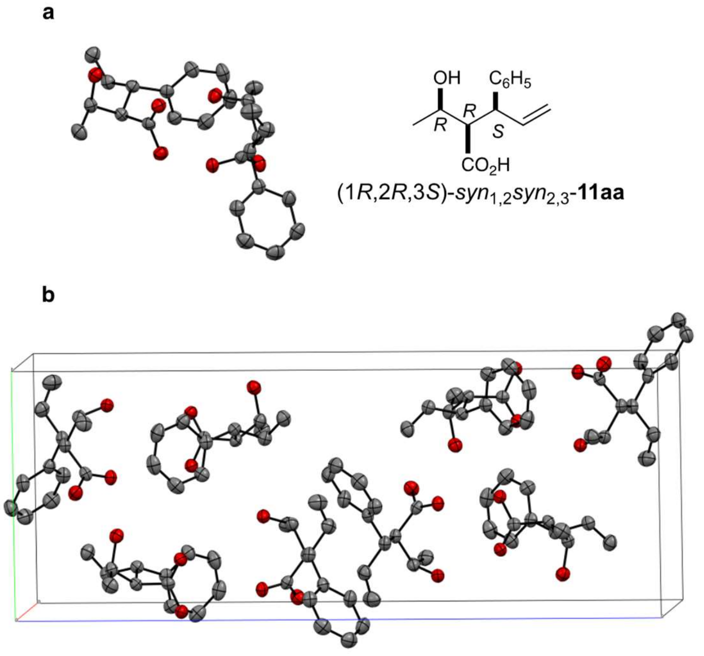

Supplementary Figure 123. ORTEP drawing (**a**) and packing diagram (**b**) of (2*R*,3*S*)-2-((*R*)-1-hydroxyethyl)-3-phenylpent-4-enoic acid ((1*R*,2*R*,3*S*)-*syn*$_{1,2}$*syn*$_{2,3}$-**11aa**).

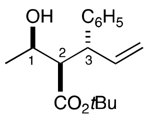

(1*R*,2*R*,3*R*)-*syn*$_{1,2}$*anti*$_{2,3}$-**4aa**

a

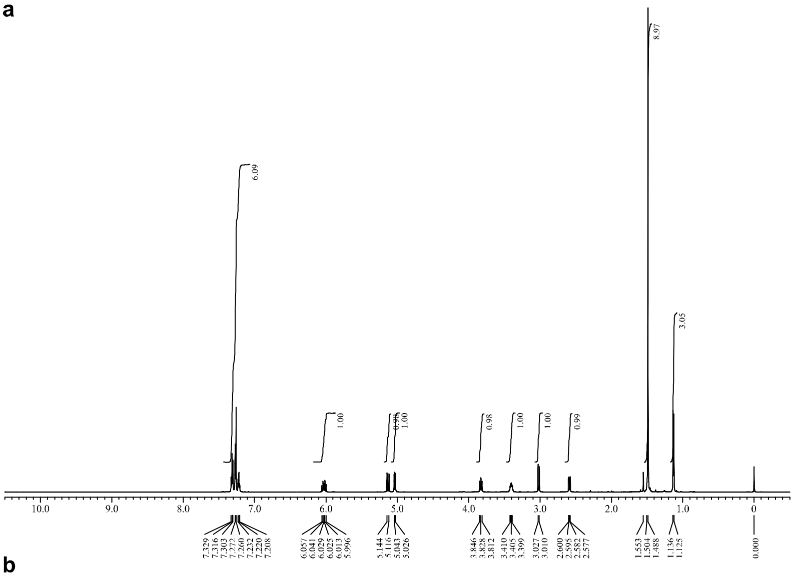

b

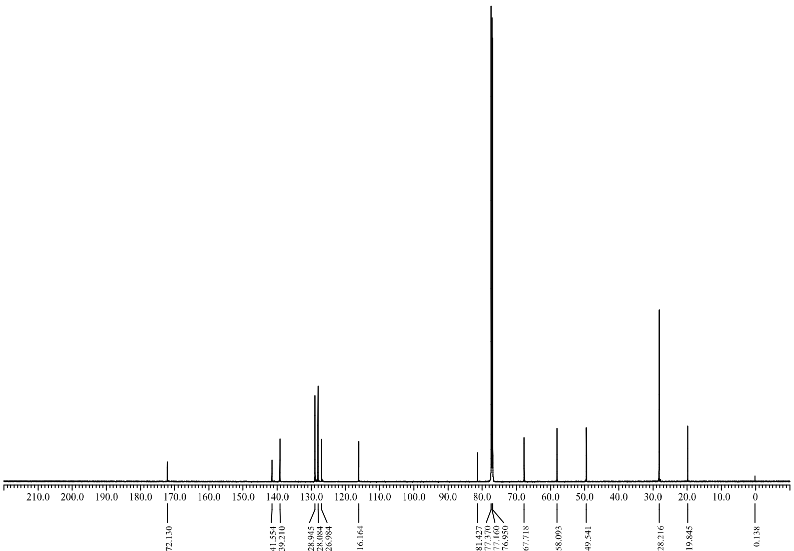

Supplementary Figure 124. ^{1}H-NMR (**a**) and ^{13}C-NMR (**b**) spectra of *tert*-butyl (2*R*,3*R*)-2-((*R*)-1-hydroxyethyl)-3-phenylpent-4-enoate ((1*R*,2*R*,3*R*)-*syn*$_{1,2}$*syn*$_{2,3}$-**4aa**) in $CDCl_3$ (**Figure 4**).

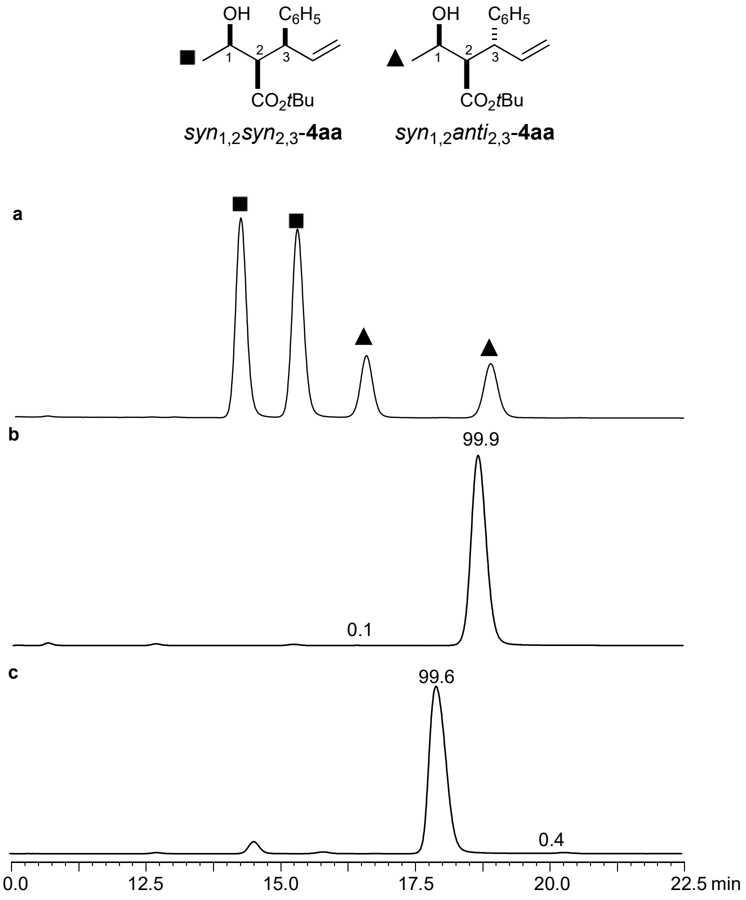

Supplementary Figure 125. HPLC charts of racemic (**a**), synthetic (**b**) *tert*-butyl (2*R*,3*R*)-2-((*R*)-1-hydroxyethyl)-3-phenylpent-4-enoate ((1*R*,2*R*,3*R*)-$syn_{1,2}anti_{2,3}$-**4aa**) and synthetic (**c**) *tert*-butyl (2*S*,3*S*)-2-((*S*)-1-hydroxyethyl)-3-phenylpent-4-enoate ((1*S*,2*S*,3*S*)- $syn_{1,2}anti_{2,3}$-**4aa**) (**Figure 4**). Conditions: column, CHIRALPAK IA-3; eluent, 2.0:98.0 2-PrOH–Hex; flow rate, 0.50 mL/min; detection, 220-nm light.

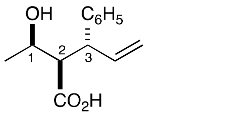

(1*R*,2*R*,3*R*)-*syn*$_{1,2}$*anti*$_{2,3}$-**11aa**

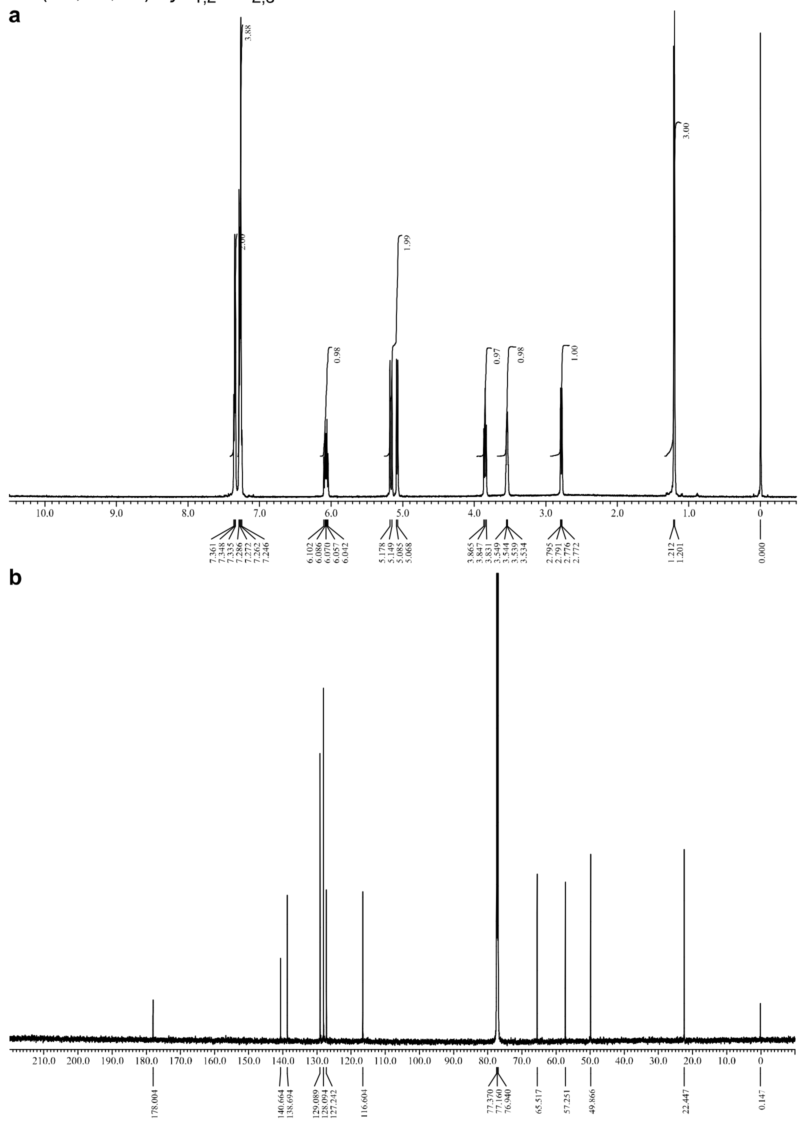

Supplementary Figure 126. ^{1}H-NMR (**a**) and ^{13}C-NMR (**b**) spectra of (2*R*,3*R*)-2-((*R*)-1-hydroxyethyl)-3-phenylpent-4-enoic acid ((1*R*,2*R*,3*R*)-*syn*$_{1,2}$*anti*$_{2,3}$-**11aa**) in $CDCl_3$.

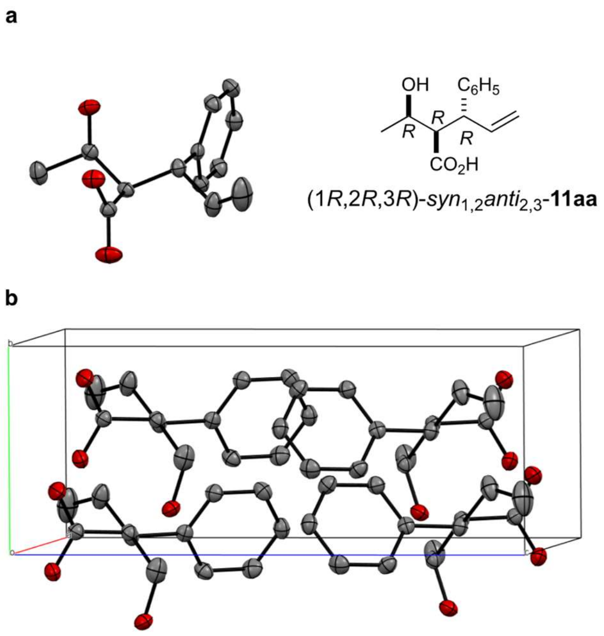

Supplementary Figure 127. ORTEP drawing (**a**) and packing diagram (**b**) of (2*R*,3*R*)-2-((*R*)-1-hydroxyethyl)-3-phenylpent-4-enoic acid ((1*R*,2*R*,3*R*)-*syn*$_{1,2}$*anti*$_{2,3}$-**11aa**).

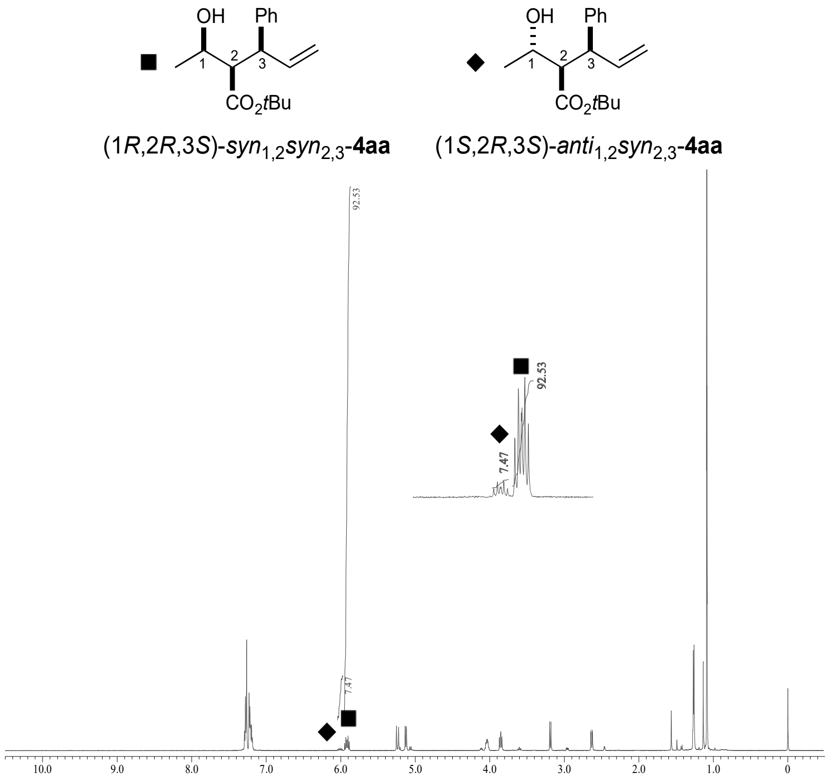

Supplementary Figure 128. ^{1}H-NMR spectrum of the reaction mixture after concentration for the reduction of *syn*-**3aa** using in Luche's condition. Solvent: $CDCl_3$. Repetition time: 10 sec.

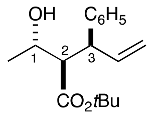

(1*S*,2*R*,3*S*)-*anti*$_{1,2}$*syn*$_{2,3}$-**4aa**

a

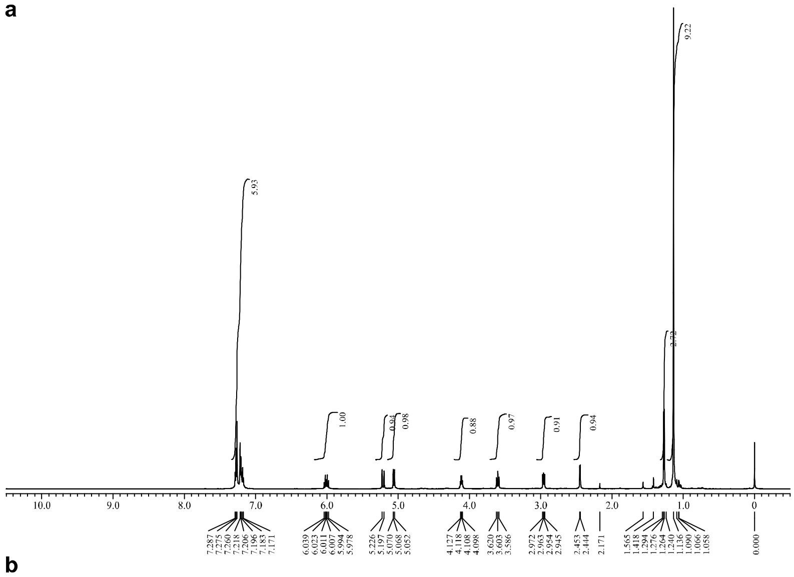

b

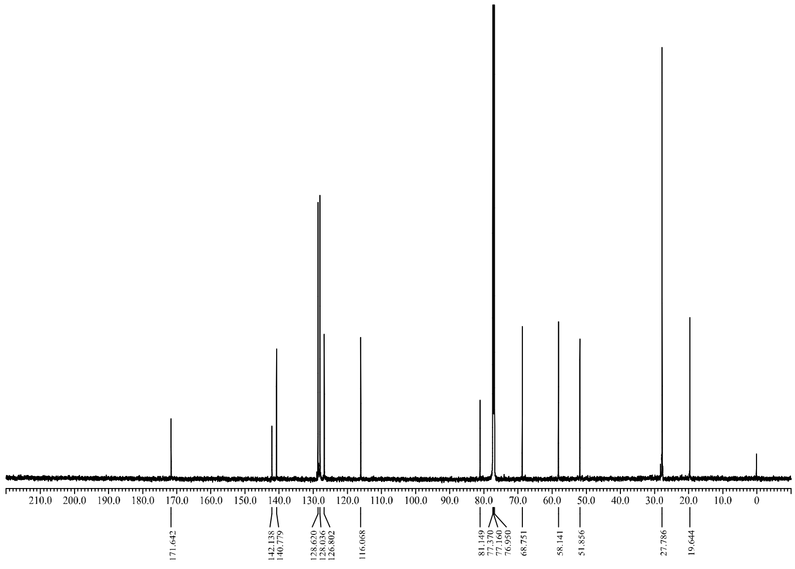

Supplementary Figure 129. ^{1}H-NMR (**a**) and ^{13}C-NMR (**b**) spectra of *tert*-butyl (2*R*,3*S*)-2-((*S*)-1-hydroxyethyl)-3-phenylpent-4-enoate ((1*S*,2*R*,3*S*)-*anti*$_{1,2}$*syn*$_{2,3}$-**4aa**) in $CDCl_3$ (**Figure 4**).

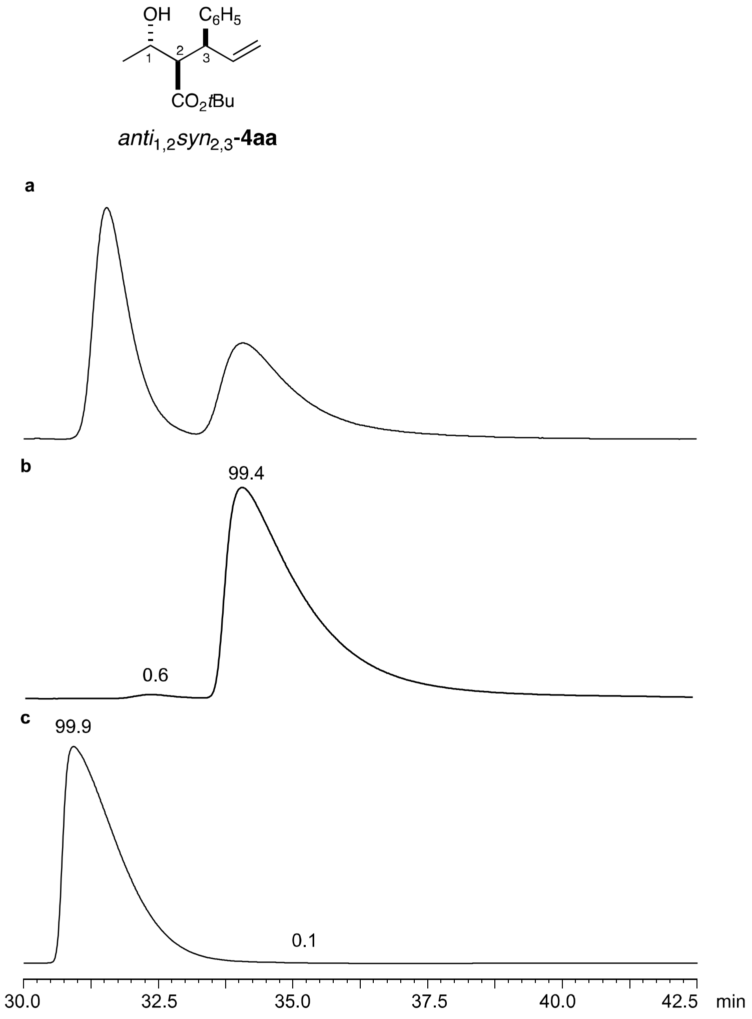

Supplementary Figure 130. HPLC charts of racemic (**a**), synthetic (**b**) *tert*-butyl (2*R*,3*S*)-2-((*S*)-1-hydroxyethyl)-3-phenylpent-4-enoate ((1*S*,2*R*,3*S*)-*anti*$_{1,2}$*syn*$_{2,3}$-**4aa**) and synthetic (**c**) *tert*-butyl (2*S*,3*R*)-2-((*S*)-1-hydroxyethyl)-3-phenylpent-4-enoate ((1*R*,2*S*,3*R*)-*anti*$_{1,2}$*syn*$_{2,3}$-**4aa**) (**Figure 4**). Conditions: column, CHIRALPAK ID-3; eluent, 1.0:99.0 2-PrOH–Hex; flow rate, 0.50 mL/min; detection, 220-nm light.

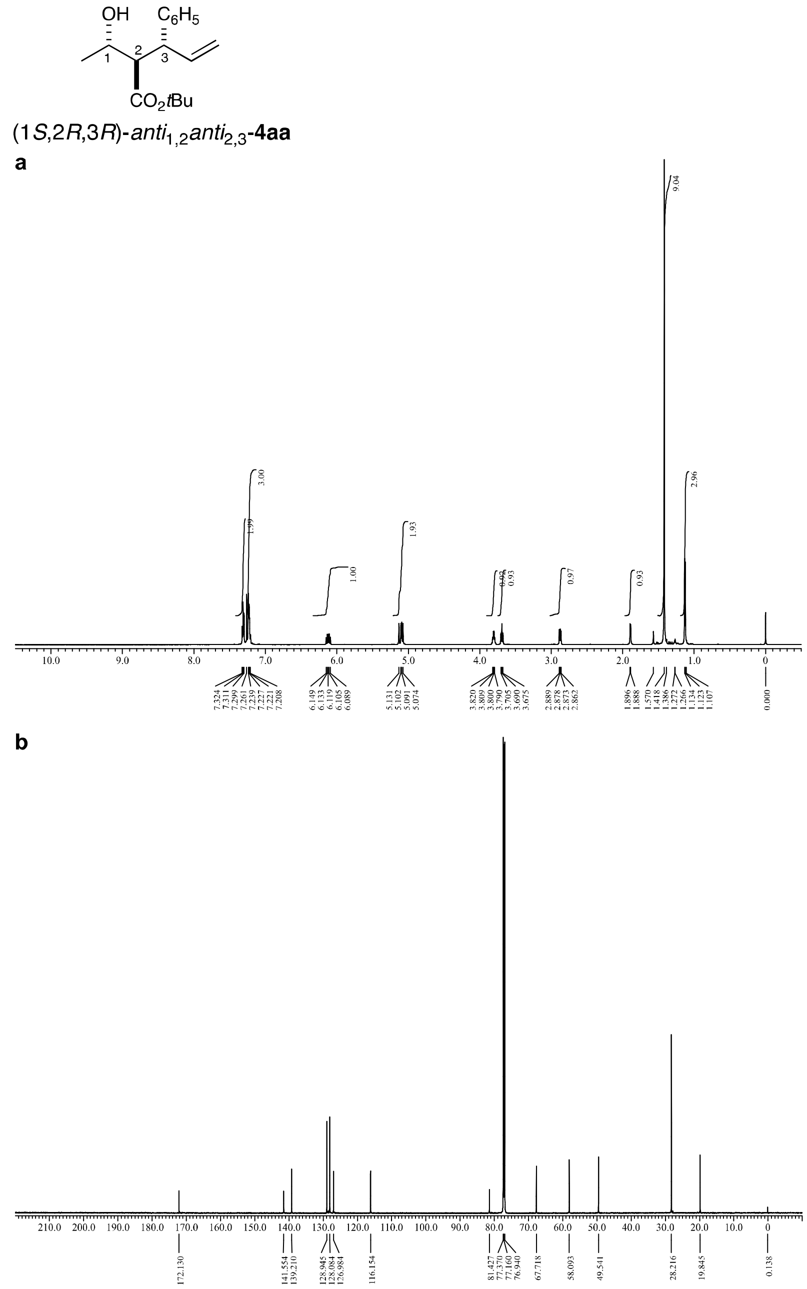

Supplementary Figure 131. ^{1}H-NMR (**a**) and ^{13}C-NMR (**b**) spectra of *tert*-butyl (2*R*,3*R*)-2-((*S*)-1-hydroxyethyl)-3-phenylpent-4-enoate ((1*S*,2*R*,3*R*)-*anti*$_{1,2}$*syn*$_{2,3}$-**4aa**) in $CDCl_3$ (**Figure 4**).

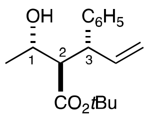

anti$_{1,2}$*anti*$_{2,3}$-**4aa**

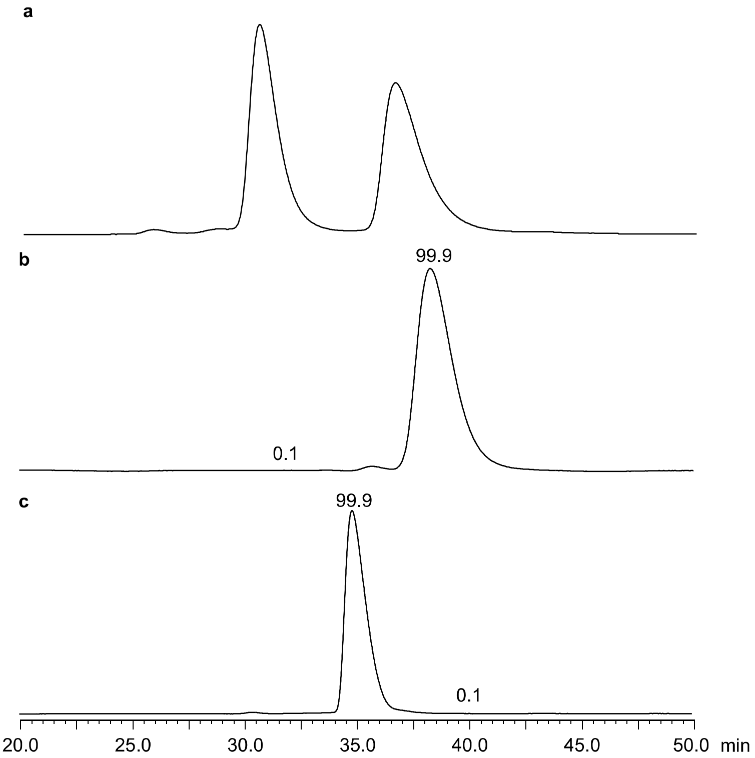

Supplementary Figure 132. HPLC charts of racemic (**a**), synthetic (**b**) *tert*-butyl (2*R*,3*R*)-2-((*S*)-1-hydroxyethyl)-3-phenylpent-4-enoate ((1*S*,2*R*,3*R*)-*anti*$_{1,2}$*anti*$_{2,3}$-**4aa**) and synthetic (**c**) *tert*-butyl (2*S*,3*S*)-2-((*R*)-1-hydroxyethyl)-3-phenylpent-4-enoate ((1*R*,2*S*,3*S*)-*anti*$_{1,2}$*anti*$_{2,3}$-**4aa**) (**Figure 4**). Conditions: column, CHIRALPAK ID-3; eluent, 1.0:99.0 2-PrOH–Hex; flow rate, 0.50 mL/min; detection, 220-nm light.

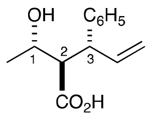

(1*S*,2*R*,3*R*)-*anti*$_{1,2}$*anti*$_{2,3}$-**11aa**

a

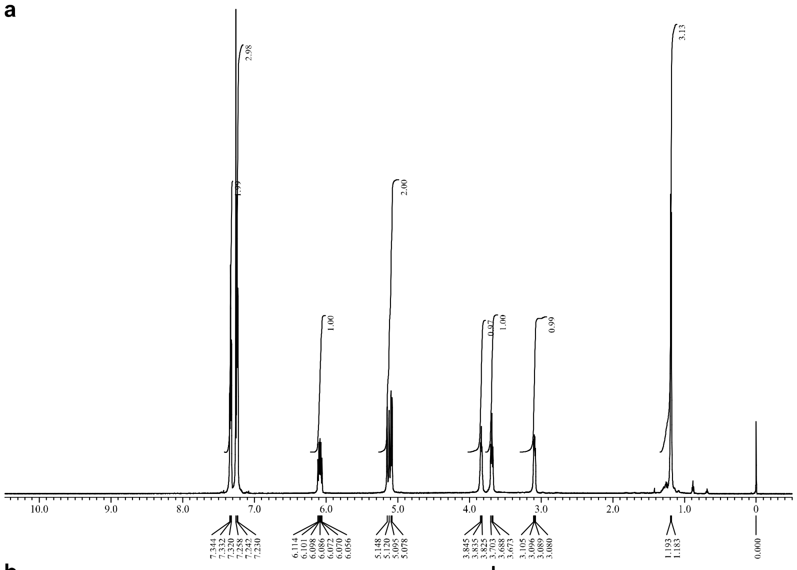

b

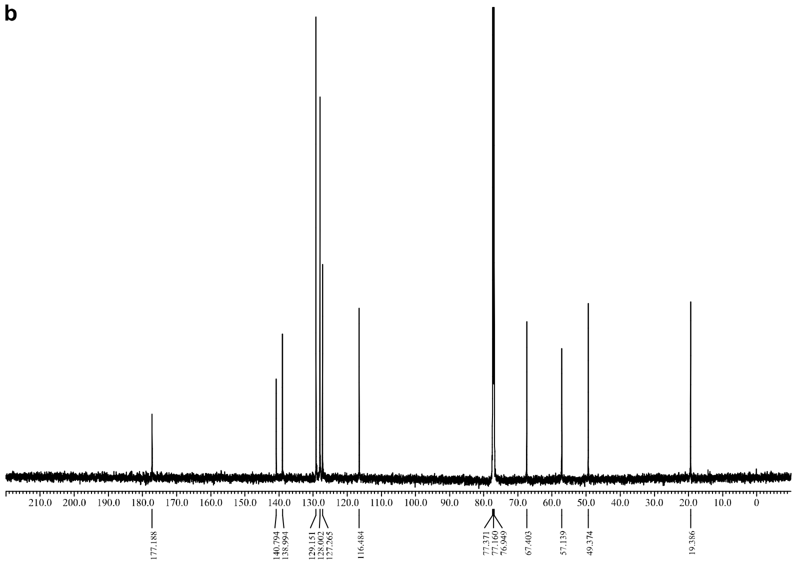

Supplementary Figure 133. ^{1}H-NMR (**a**) and ^{13}C-NMR (**b**) spectra of (2*R*,3*R*)-2-((*S*)-1-hydroxyethyl)-3-phenylpent-4-enoic acid ((1*S*,2*R*,3*R*)-*anti*$_{1,2}$*anti*$_{2,3}$-**11aa**) in $CDCl_3$.

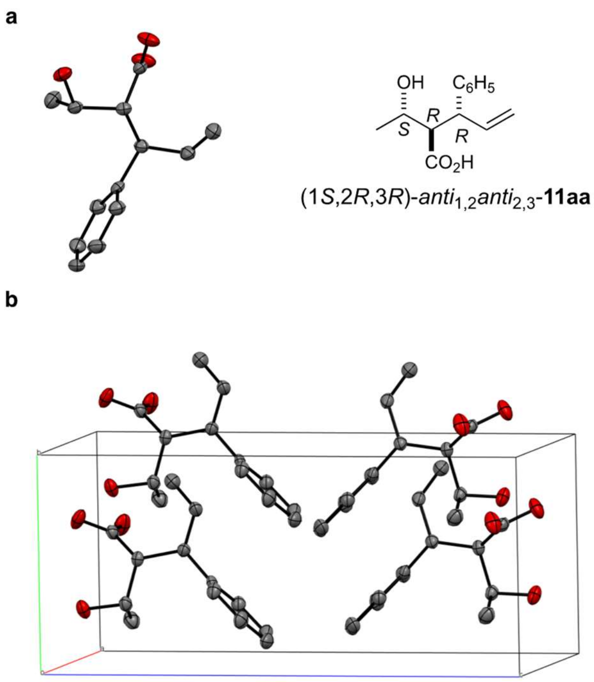

Supplementary Figure 134. ORTEP drawing (**a**) and packing diagram (**b**) of (2*R*,3*R*)-2-((*S*)-1-hydroxyethyl)-3-phenylpent-4-enoic acid ((1*S*,2*R*,3*R*)-*syn*$_{1,2}$*anti*$_{2,3}$-**11aa**).

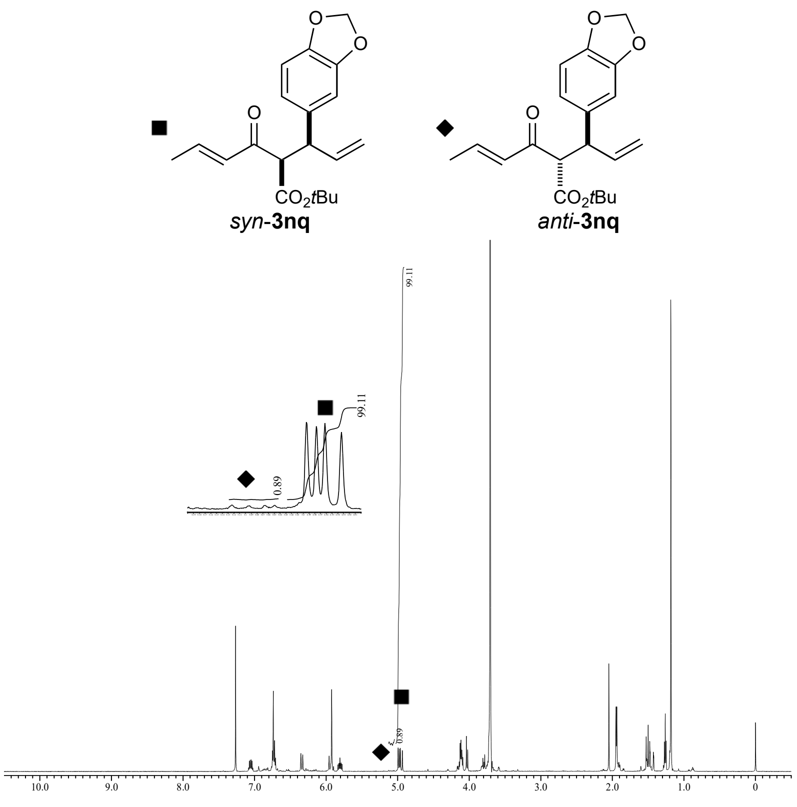

Supplementary Figure 135. ^{1}H-NMR spectrum of 20.0-mmol scale reaction mixture for synthesis of *syn*-**3nq** in $CDCl_3$. Repetition time: 10 sec.

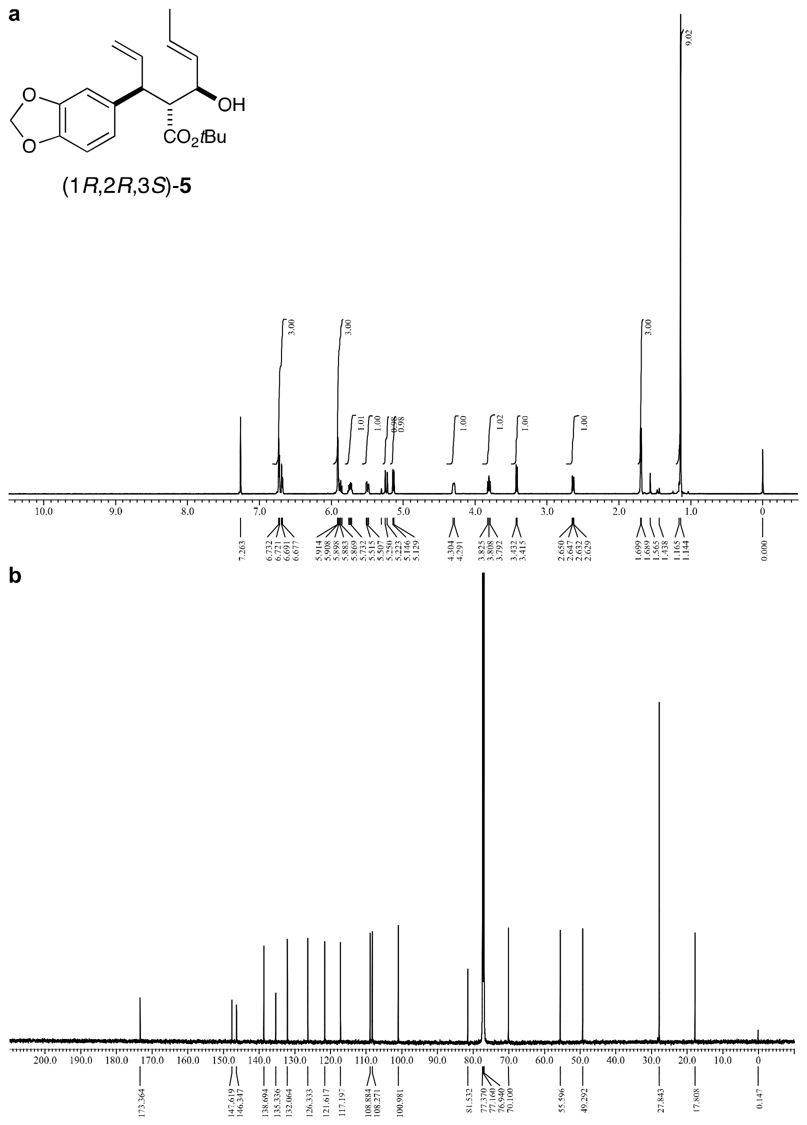

Supplementary Figure 136. ^{1}H-NMR (**a**) and ^{13}C-NMR (**b**) spectra of *tert*-butyl (2*R*,3*R*,*E*)-2-((*S*)-1-(benzo[*d*][1,3]dioxol-5-yl)allyl)-3-hydroxyhex-4-enoate ((1*R*,2*R*,3*S*)-**5**) in $CDCl_3$.

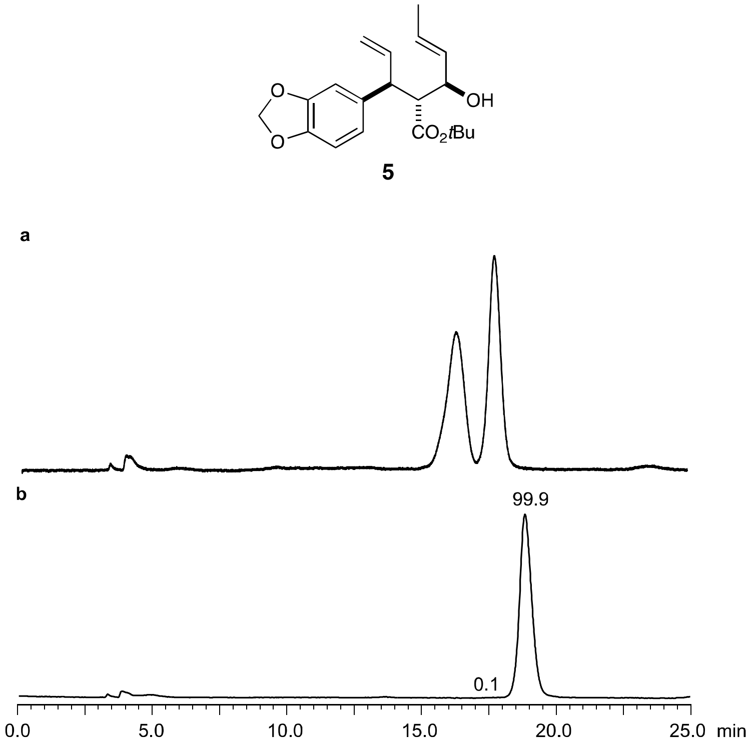

5

Supplementary Figure 137. HPLC charts of racemic (**a**), and synthetic (**b**) *tert*-butyl (2*R*,3*R*,*E*)-2-((*S*)-1-(benzo[*d*][1,3]dioxol-5-yl)allyl)-3-hydroxyhex-4-enoate ((1*R*,2*R*,3*S*)-**5**) (**Figure 5**). Conditions: column, CHIRALPAK IE-3; eluent, 2.0:98.0 2-PrOH–Hex; flow rate, 1.00 mL/min; detection, 220-nm light.

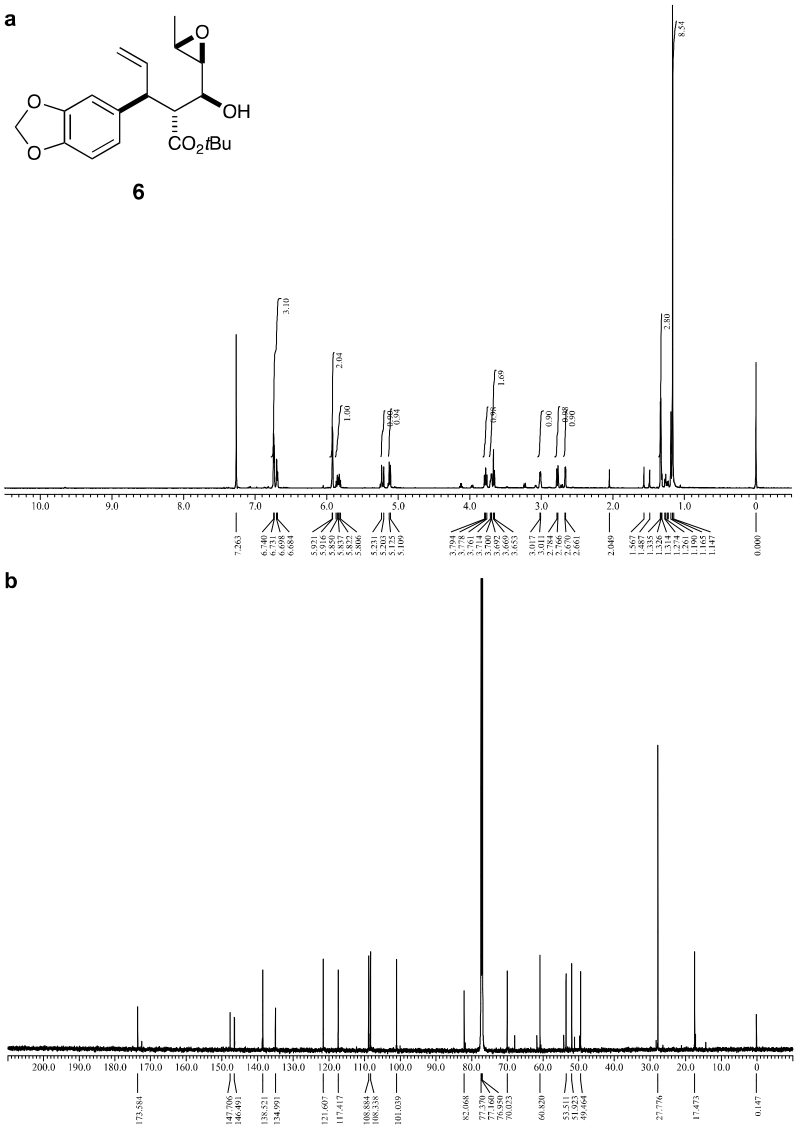

Supplementary Figure 138. ^{1}H-NMR (**a**) and ^{13}C-NMR (**b**) spectra of *tert*-butyl (2*R*,3*S*)-3-(benzo[*d*][1,3]dioxol-5-yl)-2-((*S*)-hydroxy((2*S*,3*S*)-3-methyloxiran-2-yl)methyl)pent-4-enoate (**6**) in $CDCl_3$.

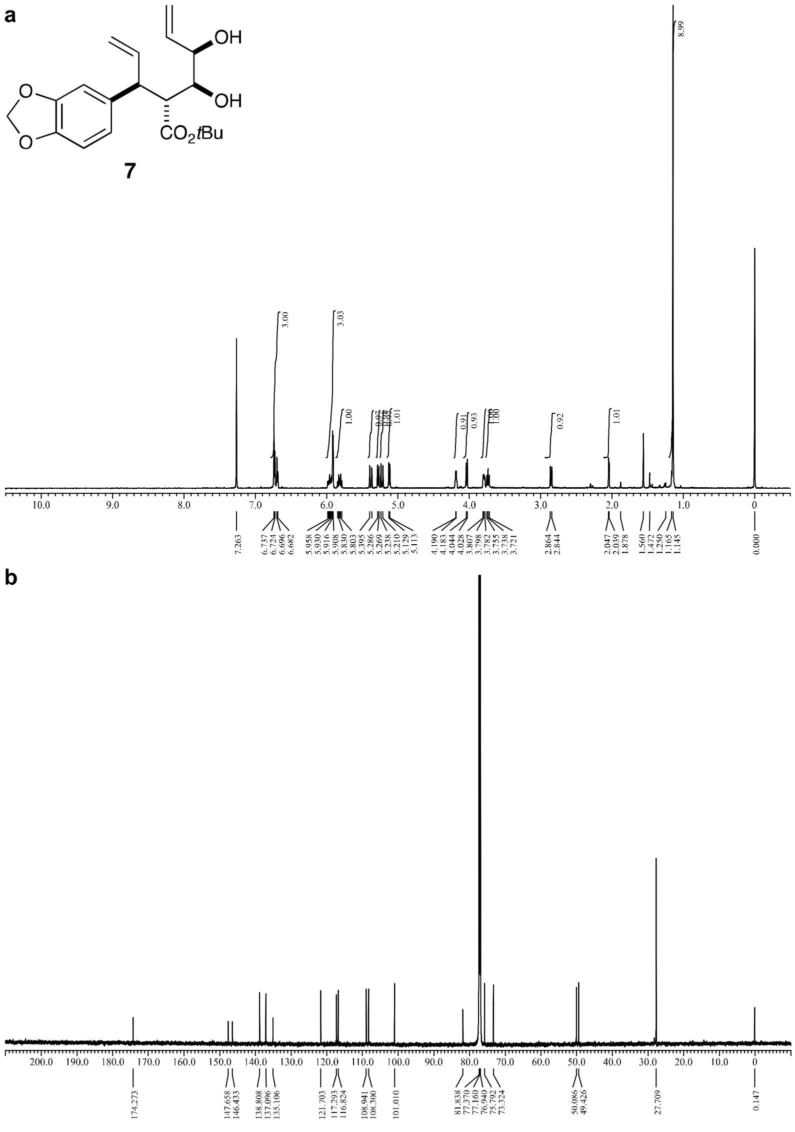

Supplementary Figure 139. ^{1}H-NMR (**a**) and ^{13}C-NMR (**b**) spectra of *tert*-butyl (2*R*,3*S*,4*R*)-2-((*S*)-1-(benzo[*d*][1,3]dioxol-5-yl)allyl)-3,4-dihydroxyhex-5-enoate (**7**) in $CDCl_3$.

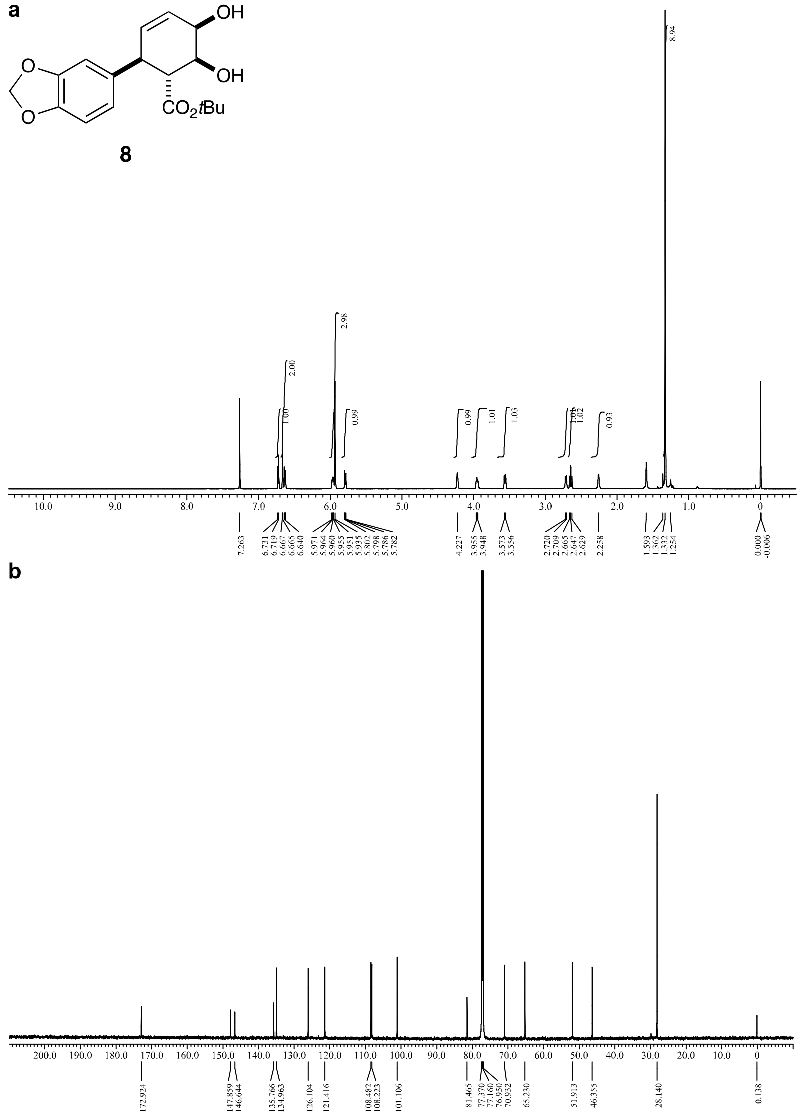

Supplementary Figure 140. ^{1}H-NMR (**a**) and ^{13}C-NMR (**b**) spectra of *tert*-butyl (1*R*,2*S*,5*R*,6*S*)-2-(benzo[*d*][1,3]dioxol-5-yl)-5,6-dihydroxycyclohex-3-ene-1-carboxylate (**8**) in $CDCl_3$.

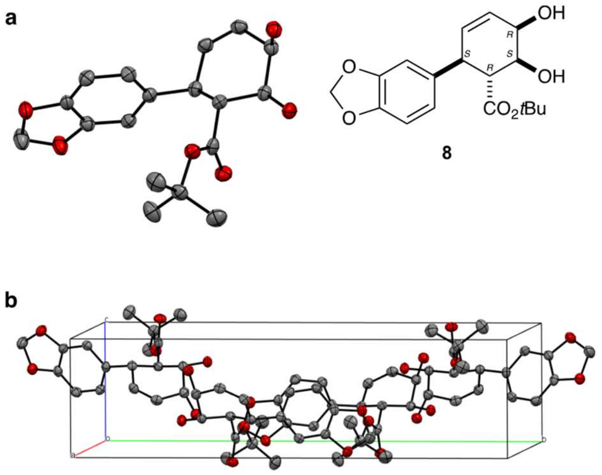

Supplementary Figure 141. ORTEP drawing (**a**) and packing diagram (**b**) of *tert*-butyl (1*R*,2*S*,5*R*,6*S*)-2-(benzo[*d*][1,3]dioxol-5-yl)-5,6-dihydroxycyclohex-3-ene-1-carboxylate ((1*R*,2*S*,5*R*,6*S*)-**8**).

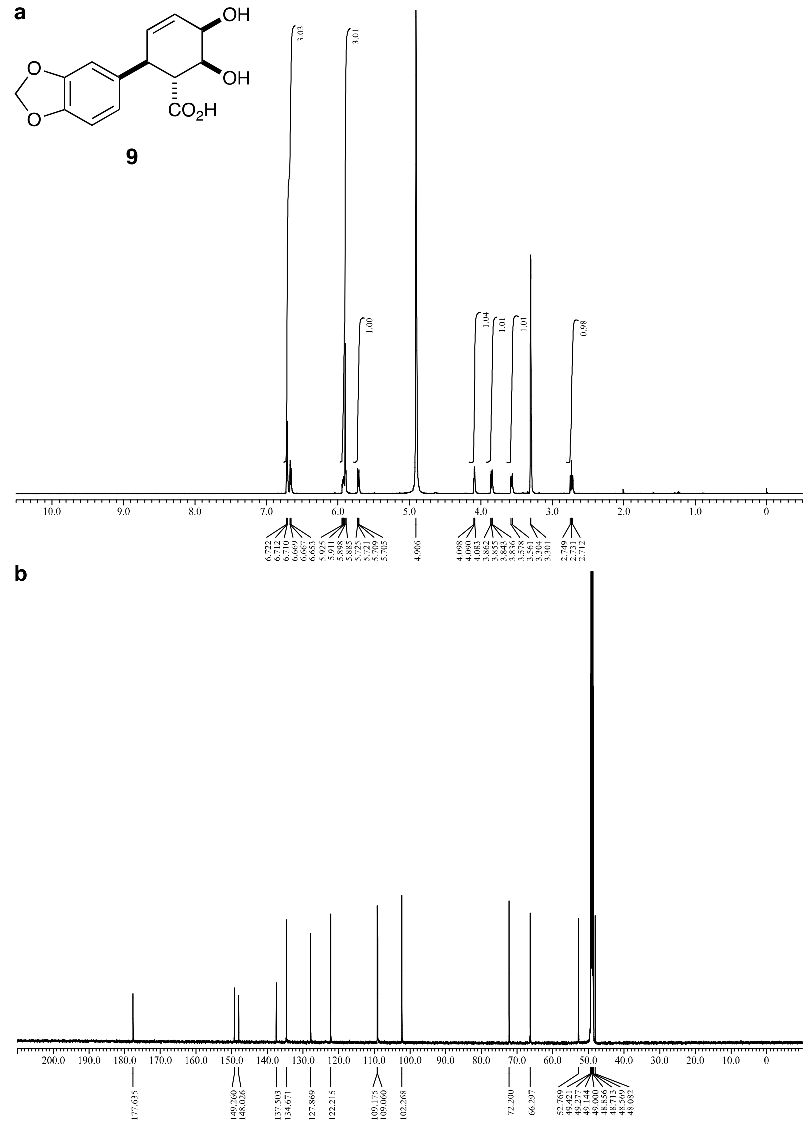

Supplementary Figure 142. ^{1}H-NMR (**a**) and ^{13}C-NMR (**b**) spectra of (1*R*,2*S*,5*R*,6*S*)-2-(benzo[*d*][1,3]dioxol-5-yl)-5,6-dihydroxycyclohex-3-ene-1-carboxylic acid (**9**) in CD_3OD.

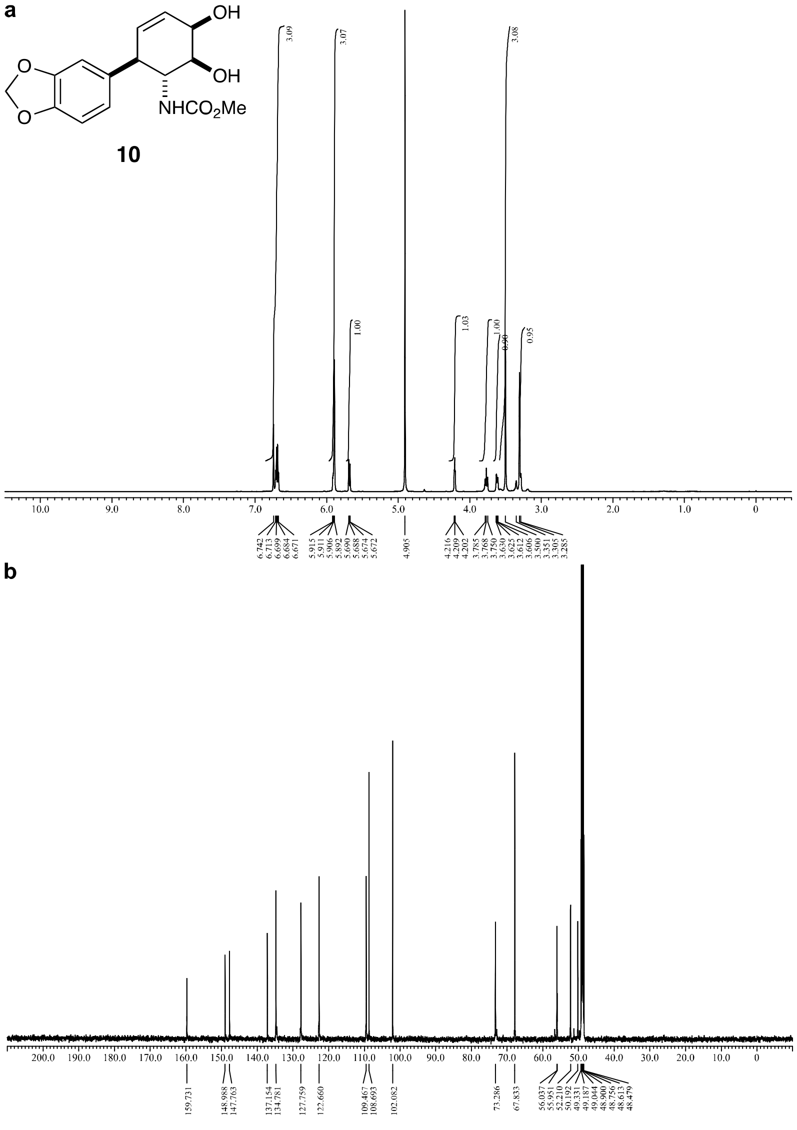

Supplementary Figure 143. ^{1}H-NMR (**a**) and ^{13}C-NMR (**b**) spectra of methyl ((1*R*,2*R*,5*R*,6*S*)-2-(benzo[*d*][1,3]dioxol-5-yl)-5,6-dihydroxycyclohex-3-en-1-yl)carbamate (**10**) in CD_3OD.

Supplementary Table 1: Crystallographic data and parameters for *tert*-butyl (2*R*,3*S*)-2-acetyl-3-phenylpent-4-enoate ((2*R*,3*S*)-*syn*-**3aa**).

mol formula	$C_{17}H_{22}O_3$
mol wt	274.36
crystal color, habit	colorless, block
crystal size, mm^3	0.300 x 0.300 x 0.100
crystal system	orthorhombic
lattice type	primitive
space group	$P2_12_12_1$ (#19)
cell dimension	
a, Å	5.6046(1)
b, Å	16.6443(3)
c, Å	17.0141(3)
vol, Å^3	1587.15(5)
Z	4
ρ calcd, g cm^{-3}	1.148
μ (Cu $K\alpha$), cm^{-1}	6.200
diffractometer	R-AXIS, RAPID
radiation	Cu $K\alpha$ (λ = 1.54187 Å)<br>graphite monochromated
$2\theta_{max}$, deg	136.4
no. of reflections measured	total: 7404
corrections	Lorentz-polarization
structure solution	SHELXT 2018/2 (Sheldrick, 2018)
function minimized by	$\Sigma\omega(F_0^2-F_c^2)^2$
refinement	full-matrix least-squares on F^2
no. of observations	2478
no. of variables	246
R	0.0358
wR^2	0.0886
goodness-of-fit indicator	1.0141
Flack parameter	0.09(6)

Supplementary Table 2: Crystallographic data and parameters for 2*R*,3*S*)-2-(hydroxymethyl)-3-phenylpent-4-enoic acid ((2*R*,3*S*)-*syn*-**11ba**).

mol formula	$C_{12}H_{14}O_3$
mol wt	206.23
crystal color, habit	colorless, chip
crystal size, mm^3	0.500 x 0.200 x 0.100
crystal system	orthorhombic
lattice type	primitive
space group	$P2_12_12_1$ (#19)
cell dimension	
a, Å	6.0023(2)
b, Å	12.6334(4)
c, Å	14.7797(4)
vol, Å^3	1120.74(6)
Z	4
ρ calcd, g cm^{-3}	1.222
μ (Cu $K\alpha$), cm^{-1}	7.14
diffractometer	R-AXIS, RAPID
radiation	Cu $K\alpha$ (λ = 1.54187 Å)
	graphite monochromated
$2\theta_{max}$, deg	136.4
no. of reflections measured	total: 13044
corrections	Lorentz-polarization
structure solution	SHELXT 2018/2 (Sheldrick, 2018)
function minimized by	$\Sigma\omega(F_0^2 - F_c^2)^2$
refinement	full-matrix least-squares on F^2
no. of observations	2015
no. of variables	177
R	0.0296
wR^2	0.0796
goodness-of-fit indicator	1.066
Flack parameter	0.05(5)

Supplementary Table 3: Crystallographic data and parameters for *tert*-butyl (2*R*,3*S*)-2-(4-chlorobenzoyl)-3-phenylpent-4-enoate ((2*R*,3*S*)-*syn*-**3ia**).

mol formula	$C_{22}H_{27}ClO_3$
mol wt	374.91
crystal color, habit	colorless, prism
crystal size, mm^3	0.500 x 0.100 x 0.100
crystal system	orthorhombic
lattice type	primitive
space group	$P2_12_12_1$ (#19)
cell dimension	
a, Å	5.68500(10)
b, Å	17.6663(3)
c, Å	19.3753(3)
vol, $Å^3$	1945.92(6)
Z	4
ρ calcd, g cm^{-3}	1.266
μ (Cu $K\alpha$), cm^{-1}	18.80
diffractometer	R-AXIS, RAPID
radiation	Cu $K\alpha$ (λ = 1.54187 Å)<br>graphite monochromated
$2\theta_{max}$, deg	136.4
no. of reflections measured	total: 21173
corrections	Lorentz-polarization
structure solution	SHELXT 2018/2 (Sheldrick, 2018)
function minimized by	$\Sigma\omega(F_0^2-F_c^2)^2$
refinement	full-matrix least-squares on F^2
no. of observations	3545
no. of variables	443
R	0.0349
wR^2	0.0776
goodness-of-fit indicator	0.973
Flack parameter	0.060(8)

Supplementary Table 4: Crystallographic data and parameters for (2*R*,3*S*)-3-(2-fluorophenyl)-2-((*R*)-1-hydroxyethyl)pent-4-enoic acid ((1*R*,2*R*,3*S*)-$syn_{1,2}syn_{2,3}$-**11ab**).

mol formula	$C_{13}H_{15}FO_3$
mol wt	238.25
crystal color, habit	colorless, block
crystal size, mm^3	0.300 x 0.200 x 0.100
crystal system	orthorhombic
lattice type	primitive
space group	$P2_12_12_1$ (#19)
cell dimension	
a, Å	8.5824(2)
b, Å	10.5729(2)
c, Å	27.6062(5)
vol, Å^3	2505.01(9)
Z	8
ρ calcd, g cm^{-3}	1.263
μ (Cu $K\alpha$), cm^{-1}	8.27
diffractometer	R-AXIS, RAPID
radiation	Cu $K\alpha$ (λ = 1.54187 Å)<br>graphite monochromated
$2\theta_{max}$, deg	136.4
no. of reflections measured	total: 29450
corrections	Lorentz-polarization
structure solution	SHELXT 2018/2 (Sheldrick, 2018)
function minimized by	$\Sigma\omega(F_0^2-F_c^2)^2$
refinement	full-matrix least-squares on F^2
no. of observations	4573
no. of variables	308
R	0.0664
wR^2	0.2087
goodness-of-fit indicator	1.076
Flack parameter	0.03(3)

Supplementary Table 5: Crystallographic data and parameters for (2*R*,3*S*)-3-(3-fluorophenyl)-2-((*R*)-1-hydroxyethyl)pent-4-enoic acid ((1*R*,2*R*,3*S*)-*syn*$_{1,2}$*syn*$_{2,3}$-**11ae**).

mol formula	$C_{13}H_{15}FO_3$
mol wt	238.25
crystal color, habit	colorless, prism
crystal size, mm^3	0.100 x 0.100 x 0.100
crystal system	orthorhombic
lattice type	primitive
space group	$P2_12_12_1$ (#19)
cell dimension	
a, Å	8.3327(3)
b, Å	10.5051(3)
c, Å	27.7393(8)
vol, Å^3	2428.18(13)
Z	8
ρ calcd, g cm^{-3}	1.303
μ (Cu $K\alpha$), cm^{-1}	8.53
diffractometer	R-AXIS, RAPID
radiation	Cu $K\alpha$ (λ = 1.54187 Å)<br>graphite monochromated
$2\theta_{max}$, deg	136.5
no. of reflections measured	total: 26361
corrections	Lorentz-polarization
structure solution	SHELXT 2018/2 (Sheldrick, 2018)
function minimized by	$\Sigma\omega(F_0^2-F_c^2)^2$
refinement	full-matrix least-squares on F^2
no. of observations	4417
no. of variables	384
R	0.0358
wR^2	0.0706
goodness-of-fit indicator	0.929
Flack parameter	-0.01(7)

Supplementary Table 6: Crystallographic data and parameters for (2*R*,3*S*)-3-(4-fluorophenyl)-2-((*R*)-1-hydroxyethyl)pent-4-enoic acid ((1*R*,2*R*,3*S*)-*syn*$_{1,2}$*syn*$_{2,3}$-**11ah**).

mol formula	$C_{13}H_{15}FO_3$
mol wt	238.25
crystal color, habit	colorless, block
crystal size, mm^3	0.500 x 0.400 x 0.400
crystal system	orthorhombic
lattice type	primitive
space group	$P2_12_12_1$ (#19)
cell dimension	
a, Å	8.2453(2)
b, Å	10.8251(2)
c, Å	28.2204(5)
vol, Å^3	2518.85(9)
Z	8
ρ calcd, g cm^{-3}	1.257
μ (Cu $K\alpha$), cm^{-1}	8.22
diffractometer	R-AXIS, RAPID
radiation	Cu $K\alpha$ (λ = 1.54187 Å)
	graphite monochromated
$2\theta_{max}$, deg	136.3
no. of reflections measured	total: 24449
corrections	Lorentz-polarization
structure solution	SHELXT 2018/2 (Sheldrick, 2018)
function minimized by	$\Sigma\omega(F_0^2-F_c^2)^2$
refinement	full-matrix least-squares on F^2
no. of observations	4560
no. of variables	332
R	0.0841
wR^2	0.2426
goodness-of-fit indicator	1.083
Flack parameter	0.06(18)

Supplementary Table 7: Crystallographic data and parameters for (2*R*,3*S*)-2-((*R*)-1-hydroxy ethyl)-3-(naphthalen-2-yl)pent-4-enoic acid ((1*R*,2*R*,3*S*)-*syn*$_{1,2}$*syn*$_{2,3}$-**11am**).

mol formula	$C_{17}H_{18}O_3$
mol wt	270.31
crystal color, habit	colorless, block
crystal size, mm^3	0.500 x 0.200 x 0.200
crystal system	orthorhombic
lattice type	primitive
space group	$P2_12_12_1$ (#19)
cell dimension	
a, Å	10.0226(2)
b, Å	10.6133(2)
c, Å	27.3413(5)
vol, Å^3	2908.37(10)
Z	8
ρ_{calcd}, g cm^{-3}	1.235
μ (Cu $K\alpha$), cm^{-1}	6.74
diffractometer	R-AXIS, RAPID
radiation	Cu $K\alpha$ (λ = 1.54187 Å)<br>graphite monochromated
$2\theta_{max}$, deg	136.4
no. of reflections measured	total: 31608
corrections	Lorentz-polarization
structure solution	SHELXT 2018/2 (Sheldrick, 2018)
function minimized by	$\Sigma\omega(F_0^2 - F_c^2)^2$
refinement	full-matrix least-squares on F^2
no. of observations	5241
no. of variables	494
R	0.0385
wR^2	0.1098
goodness-of-fit indicator	1.066
Flack parameter	0.03(5)

Supplementary Table 8: Crystallographic data and parameters for *tert*-butyl 3-((3*S*,4*R*)-4-(*tert*-butoxycarbonyl)-5-oxohex-1-en-3-yl)-1*H*-indole-1-carboxylate ((*R*,*S*)-*syn*-**3ao**).

mol formula	$C_{24}H_{31}NO_5$
mol wt	413.50
crystal color, habit	colorless, block
crystal size, mm^3	0.500 x 0.400 x 0.300
crystal system	orthorhombic
lattice type	primitive
space group	$P2_12_12_1$ (#19)
cell dimension	
a, Å	10.0901(2)
b, Å	11.2770(2)
c, Å	20.1031(4)
vol, Å^3	2287.45(8)
Z	4
ρ_{calcd}, g cm^{-3}	1.201
μ (Cu $K\alpha$), cm^{-1}	6.77
diffractometer	R-AXIS, RAPID
radiation	Cu $K\alpha$ (λ = 1.54187 Å)
	graphite monochromated
$2\theta_{max}$, deg	136.5
no. of reflections measured	total: 20861
corrections	Lorentz-polarization
structure solution	SHELXT 2018/2 (Sheldrick, 2018)
function minimized by	$\Sigma\omega(F_o^2-F_c^2)^2$
refinement	full-matrix least-squares on F^2
no. of observations	3942
no. of variables	372
R	0.0316
wR^2	0.0624
goodness-of-fit indicator	0.754
Flack parameter	0.15(8)

Supplementary Table 9: Crystallographic data and parameters for *tert*-butyl (2*R*,3*R*)-2-acetyl-3-phenylpent-4-enoate ((*R*,*R*)-*anti*-**3aa**).

mol formula	$C_{17}H_{22}O_3$
mol wt	274.34
crystal color, habit	colorless, prism
crystal size, mm^3	0.400 x 0.300 x 0.200
crystal system	orthorhombic
lattice type	primitive
space group	$P2_12_12_1$ (#19)
cell dimension	
a, Å	8.7220(2)
b, Å	10.3044(2)
c, Å	18.2870(4)
vol, Å^3	1643.54(6)
Z	4
ρ_{calcd}, g cm^{-3}	1.109
μ (Cu $K\alpha$), cm^{-1}	5.97
diffractometer	R-AXIS, RAPID
radiation	Cu $K\alpha$ (λ = 1.54187 Å)<br>graphite monochromated
$2\theta_{max}$, deg	136.5
no. of reflections measured	total: 10214
corrections	Lorentz-polarization
structure solution	SHELXT 2018/2 (Sheldrick, 2018)
function minimized by	$\Sigma\omega(F_0^2-F_c^2)^2$
refinement	full-matrix least-squares on F^2
no. of observations	2683
no. of variables	230
R	0.0365
wR^2	0.0950
goodness-of-fit indicator	1.009
Flack parameter	0.07(9)

Supplementary Table 10: Crystallographic data and parameters for (2*R*,3*S*)-3-phenyl-2-((*R*)-1-hydroxyethyl)pent-4-enoic acid ((1*R*,2*R*,3*S*)-$syn_{1,2}syn_{2,3}$-**11aa**).

mol formula	$C_{13}H_{16}O_3$
mol wt	220.27
crystal color, habit	colorless, block
crystal size, mm^3	0.400 x 0.400 x 0.300
crystal system	orthorhombic
lattice type	primitive
space group	$P2_12_12_1$ (#19)
cell dimension	
a, Å	8.3042(2)
b, Å	10.6012(2)
c, Å	27.5177(5)
vol, $Å^3$	2422.51(9)
Z	8
ρ calcd, g cm^{-3}	1.208
μ (Cu $K\alpha$), cm^{-1}	6.91
diffractometer	R-AXIS, RAPID
radiation	Cu $K\alpha$ (λ = 1.54187 Å)<br>graphite monochromated
$2\theta_{max}$, deg	136.4
no. of reflections measured	total: 27456
corrections	Lorentz-polarization
structure solution	SHELXT 2018/2 (Sheldrick, 2018)
function minimized by	$\Sigma\omega(F_0^2-F_c^2)^2$
refinement	full-matrix least-squares on F^2
no. of observations	4382
no. of variables	318
R	0.0266
wR^2	0.0681
goodness-of-fit indicator	1.019
Flack parameter	0.11(4)

Supplementary Table 11: Crystallographic data and parameters for (2*R*,3*R*)-3-phenyl-2-((*R*)-1-hydroxyethyl)pent-4-enoic acid ((1*R*,2*R*,3*R*)-*syn*$_{1,2}$*anti*$_{2,3}$-**11aa**).

mol formula	$C_{13}H_{16}O_3$
mol wt	220.26
crystal color, habit	colorless, prism
crystal size, mm^3	0.500 x 0.300 x 0.200
crystal system	monoclinic
lattice type	C-centered
space group	C2 (#5)
cell dimension	
a, Å	15.2729(4)
b, Å	5.6683(2)
c, Å	14.3661(4)
β, deg	97.701(7)
vol, Å^3	1232.48(7)
Z	4
ρ calcd, g cm^{-3}	1.187
μ (Cu $K\alpha$), cm^{-1}	6.79
diffractometer	R-AXIS, RAPID
radiation	Cu $K\alpha$ (λ = 1.54187 Å) graphite monochromated
$2\theta_{max}$, deg	136.4
no. of reflections measured	total: 6.638
corrections	Lorentz-polarization
structure solution	SHELXT 2018/2 (Sheldrick, 2018)
function minimized by	$\Sigma\omega(F_0^2 - F_c^2)^2$
refinement	full-matrix least-squares on F^2
no. of observations	1951
no. of variables	198
R	0.0394
wR^2	0.1156
goodness-of-fit indicator	1.113
Flack parameter	0.07(7)

Supplementary Table 12: Crystallographic data and parameters for (2*R*,3*R*)-3-phenyl-2-((*R*)-1-hydroxyethyl)pent-4-enoic acid ((1*R*,2*R*,3*R*)-$syn_{1,2}anti_{2,3}$-**11aa**).

mol formula	$C_{13}H_{16}O_3$
mol wt	220.26
crystal color, habit	colorless, prism
crystal size, mm^3	0.500 x 0.300 x 0.200
crystal system	monoclinic
lattice type	C-centered
space group	C2 (#5)
cell dimension	
a, Å	10.0234(5)
b, Å	7.2000(4)
c, Å	16.2871(8)
β, deg	94.908(7)
vol, Å^3	1171.11(11)
Z	4
ρ calcd, g cm^{-3}	1.249
μ (Cu $K\alpha$), cm^{-1}	7.14
diffractometer	R-AXIS, RAPID
radiation	Cu $K\alpha$ (λ = 1.54187 Å) graphite monochromated
$2\theta_{max}$, deg	136.3
no. of reflections measured	total: 6.011
corrections	Lorentz-polarization
structure solution	SHELXT 2018/2 (Sheldrick, 2018)
function minimized by	$\Sigma\omega(F_0^2-F_c^2)^2$
refinement	full-matrix least-squares on F^2
no. of observations	1856
no. of variables	194
R	0.0348
wR^2	0.0802
goodness-of-fit indicator	0.992
Flack parameter	0.11(15)

Supplementary Table 13: Screening of solvent and temperature[a]

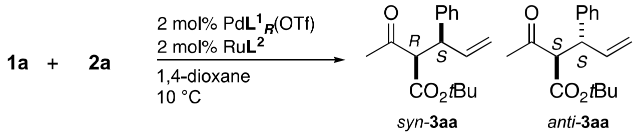

entry	Ru**L**^{2}	solvent	temp. (°C)	time (h)	NMR yield (%)	dr	er (major)
1	Ru**L**^2_S	THF	25	12	quant.	94.4:5.6	n.d.
2	Ru**L**^2_R	THF	10	12	quant.	99.3:0.7	99.8:0.2
3	Ru**L**^2_R	THF	10	48	80.1	28.6:71.4	n.d.
4	Ru**L**^2_R	THF	25	24	quant.	19.2:80.8	99.3:0.7
5	Ru**L**^2_R	$CHCl_3$	25	24	42.8	35.7:64.3	n.d.
6	Ru**L**^2_R	CH_2Cl_2	25	24	46.7	34.5:65.5	n.d.
7	Ru**L**^2_R	CPME	25	24	quant.	32.3:67.7	n.d.
8	Ru**L**^2_R	TBME	25	24	quant.	33.3:66.7	n.d.
9	Ru**L**^2_R	Toluene	25	24	quant.	34.5:65.5	n.d.
10	Ru**L**^2_R	1,4-dioxane	25	24	quant.	13.9:86.1	n.d.
11[b]	Ru**L**^2_R	1,4-dioxane	25	48	quant.	7.5:92.5	n.d.
12[b,c]	Ru**L**^2_R	1,4-dioxane	10	72	quant.	6.2:93.8	n.d.
13[b,d]	Ru**L**^2_R	1,4-dioxane	10	72	quant.	3.9:96.1	99.2:0.8
14	Ru**L**^2_S	1,4-dioxane	10	12	quant.	99.5:0.5	99.7:0.3

[a]Conditions unless otherwise specified: [**1a**] = 500 mM, [**2a**] = 600 mM;[Pd**L**^1_R(OTf)] = [Ru**L**^2_S] = 10.0 mM; 1,4-dioxane 1.00 mL. [b]1 mol% of Pd**L**^1_R(OTf) was used. [c][**1a**] = 250 mM, [**2a**] = 300 mM. [d][**1a**] = 125 mM, [**2a**] = 150 mM

Supplementary Table 14: Crystallographic data and parameters for *tert*-butyl (1*R*,2*S*,5*R*,6*S*)-2-(benzo[*d*][1,3]dioxol-5-yl)-5,6-dihydroxycyclohex-3-ene-1-carboxylate ((1*R*,2*S*,5*R*,6*S*)-**8**).

mol formula	$C_{18}H_{22}O_6$
mol wt	334.35
crystal color, habit	colorless, prism
crystal size, mm^3	0.500 x 0.100 x 0.100
crystal system	orthorhombic
lattice type	C-centered
space group	$P2_12_12_1$ (#18)
cell dimension	
a, Å	13.5170(3)
b, Å	21.5528(4)
c, Å	5.81610(10)
vol, Å^3	1694.40(6)
Z	4
ρ calcd, g cm^{-3}	1.311
μ (Cu $K\alpha$), cm^{-1}	8.16
diffractometer	R-AXIS, RAPID
radiation	Cu $K\alpha$ (λ = 1.54187 Å)
	graphite monochromated
$2\theta_{max}$, deg	136.4
no. of reflections measured	total: 6.011
corrections	Lorentz-polarization
structure solution	direct methods (SIR92)
function minimized by	$\Sigma\omega(F_0^2 - F_c^2)^2$
refinement	full-matrix least-squares on F^2
no. of observations	3077
no. of variables	416
R	0.0320
wR^2	0.0642
goodness-of-fit indicator	0.894
Flack parameter	0.10(8)

Supplementary References.

(1) Nakata, T.; Tani, Y.; Hatozaki, M.; Oishi, T. *Chem. Pharm. Bull.* **1984,** *32,* 1411–1415.
(2) Buschbeck, L.; Christoffers, J. *J. Org. Chem.* **2018,** *83,* 4002–4014.
(3) Meyer, W. L.; Brannon, M. J.; Burgos, C. da G.; Goodwin, T. E.; Howard, R. W. *J. Org. Chem.* **1985**, *50*, 438–447.
(4) Evans, S. D.; Houghton, R. P. *J. Mol. Cat. A: Chem.* **2000**, *164*, 157–164.
(5) Angelov, P.; Chau, Y. K. S.; Fryer, P. J.; Moloney, M. G.; Thompson, A. L.; Trippier, P. C. *Org. Biomol. Chem.* **2012**, *10*, 3472–3485.
(6) Agura, K.; Hayashi, Y.; Wada, M.; Nakatake, D.; Mashima, K.; Ohshima, T. *Chem. Asian J.* **2016**, *11*, 1548–1554.
(7) Peixoto, P. A.; Boulange, A.; Ball, M.; Naudin, B.; Alle, T.; Cosette, P.; Karuso, P.; Franck, X. *J. Am. Chem. Soc.* **2014**, *136*, 15248–15256.
(8) Erhardt, H.; Mohr, F.; Kirsch, S. F. *Eur. J. Org. Chem.* **2016**, 5629–5632.
(9) Lee, S. I.; Hwang, G.-S.; Ryu, D. H. *J. Am. Chem. Soc.* **2013**, *135*, 7126–7129.
(10) Qian, J.; Yi, W.; Huang, X.; Miao, Y.; Zhang, J.; Cai, C.; Zhang, W. *Org. Lett.* **2015**, *17*, 1090–1093.
(11) Takagi, R.; Fujii, E.; Kondo, H. *J. Org. Chem.* **2018**, *83*, 11191–11203.
(12) Yang, B.; Wang, Z.-X. *Org. Lett.* **2019**, *21*, 7965–7969.
(13) Kreibich, M.; Petrović, D.; Brückner, R. *J. Org. Chem.* **2018**, *83*, 1116–1133.
(14) He, J.; Jia, Z.; Tan, H.; Luo, X.; Qiu, D.; Shi, J.; Xu, H.; Li, Y. *Angew. Chem. Int. Ed.* **2019**, *58*, 18513–18518.
(15) Yang, B.; Wang, Z.-X. *Org. Lett.* **2019**, *21*, 7965–7969.
(16) Beaud, R.; Michelet, B.; Reviriot, Y.; Martin-Mingot, A.; Rodriguez, J.; Bonne, D.; Thbaideau, S. *Angew. Chem. Int. Ed.* **2020**, *59*, 1279–1285.
(17) He, J.; Jia, Z.; Tan, H.; Luo, X.; Qiu, D.; Shi, J.; Xu, H.; Li, Y. *Angew. Chem. Int. Ed.* **2019**, *58*, 18513–18518.
(18) Liu, X.; Liu, S.; Wang, Q.; Zhou, G.; Yao, L.; Ouyang, Q.; Jiang, R.; Lan, Y.; Chen, W. *Org. Lett.* **2020**, *22*, 3149–3154.
(19) Podunavac, M.; Lacharity, J. J.; Jones, K. E.; Zakarian, A. *Org. Lett.* **2018**, *20*, 4867–4870.
(20) Yang, M. H.; Orsi, D. L.; Altman, R. A. *Angew. Chem. Int. Ed.* **2015**, *54*, 2361–2365.
(21) Miyata, K.; Kutsuna, H.; Kawakami, S.; Kitamura, M. *Angew. Chem. Int. Ed.* **2011**, *50*, 4649–4653.
(22) Suzuki, S.; Kitamura, Y.; Lectard, S.; Hamashima, Y.; Sodeoka, M. *Angew. Chem. Int. Ed.* **2012**, *51,* 4581–4585.
(23) Hudlicky, T.; Tian, X.; Königsberger, K.; Maurya, R.; Rouden, J.; Fan, B. *J. Am. Chem. Soc.* **1996**, *118*, 10752–10765.
